# Explore the Optimal Treatment Regimen Across Combinations of Variate Protein Sources and Exercise Modalities and Its Associated Factors in Older Adults: A Network Meta-Analysis and Meta-Regression of Randomized Controlled Trials

**DOI:** 10.3390/nu18091409

**Published:** 2026-04-29

**Authors:** Che-Li Lin, Shih-Wei Huang, Hung-Chou Chen, Mao-Hua Huang, Tsan-Hon Liou, Chun-De Liao

**Affiliations:** 1 Department of Orthopedic Surgery, Shuang Ho Hospital, Taipei Medical University, New Taipei City 23561, Taiwan; 11010@s.tmu.edu.tw; 2 Department of Orthopedics, School of Medicine, College of Medicine, Taipei Medical University, Taipei 11031, Taiwan; 3 Department of Physical Medicine and Rehabilitation, Wan Fang Hospital, Taipei Medical University, Taipei 116079, Taiwan; 113128@w.tmu.edu.tw (S.-W.H.); peter_liou@s.tmu.edu.tw (T.-H.L.); 4 Department of Physical Medicine and Rehabilitation, School of Medicine, College of Medicine, Taipei Medical University, Taipei 110301, Taiwan; 10462@s.tmu.edu.tw; 5 Department of Physical Medicine and Rehabilitation, Shuang Ho Hospital, Taipei Medical University, New Taipei City 235041, Taiwan; 6 Department of Biochemistry, University of Washington, Seattle, WA 98015, USA; 7 International Ph.D. Program in Gerontology and Long-Term Care, College of Nursing, Taipei Medical University, Taipei 110301, Taiwan

**Keywords:** sarcopenia, whey protein, exercise, lean mass, strength, mobility

## Abstract

**Background/Objectives:** Aging is closely associated with sarcopenia, which has a significant impact on muscle mass and its function. Protein supplementation (PS) brings benefits such as lean mass and strength gains during exercise training. This paper determined the optimal regimen among the composites of variate protein sources and training modalities for older individuals. **Methods:** We comprehensively searched the electronic databases, namely MEDLINE Complete, PEDro, the Cochrane Library, Google Scholar, EMBASE, and the China National Knowledge Infrastructure, from its inception until December 2025. We included randomized controlled trials (RCTs) that examined the effectiveness of any type of PS combined with one of three exercise types—resistance, aerobic, or multicomponent training—in untrained older adults. The main outcomes used to identify sarcopenia were assessed, including lean mass, handgrip and leg strength, and physical mobility measures. Network meta-analysis (NMA) was performed by a frequentist method using random-effects models. The estimated treatment effect was expressed as the standard mean difference (SMD) with a 95% confidence interval (CI). Any potential factor moderating the treatment effect was determined by the meta-regression analyses, including participant characteristics and methodological factors. Certainty of evidence (CoE) was assessed by the GRADE framework. **Results**: In total, we included 235 RCTs (20,980 participants) for analyses. A total of 10 protein sources (whey, soy, casein, milk, and the others) were identified, corresponding to 24 monotherapy and combined regimens of PS and exercise. Among the treatment arms, whey plus resistance training was ranked as the most effective treatment for muscle mass (large SMD, 1.29; CoE, moderate) and leg strength (large SMD, 1.16; CoE, moderate); additionally, whey plus multicomponent exercise training achieved the most promising effects on such sarcopenia-related physical indicators such as chair rise (large effect, SMD = 1.09; CoE: high), timed up and go (medium SMD, 0.70; CoE, high), and global mobility score (large SMD, 1.02; CoE, high). **Conclusions**: The treatment efficacy appears to be moderated by the participant’s conditions, PS resource, and PS dose, particularly the outcome of muscle mass and strength. The present NMA results indicate that whey protein incorporated with resistance training is the optimal program to help combat sarcopenia in older adults.

## 1. Introduction

Sarcopenia has been recognized as a skeletal muscle disease commonly derived from aging-induced muscle degeneration, including the loss of muscle mass and morphological changes [1,2]. Such muscle attenuations can further contribute to strength deterioration and physical mobility decline, which in turn result in high risks of frailty and disability in the elderly [3,4,5]. Based on the rapidly growing rate of the aging population worldwide, sarcopenia is becoming prevalent among older people, especially those with chronic or acute conditions [6]. Sarcopenia has an estimated prevalence of 8% to 13% in healthy community-dwelling older adults across the world, dependent on the measured methods of muscle mass [7,8], and it increases to 31–51% among institutionalized elder residents. Since sarcopenia comes with numerous adverse effects that place a significant burden on the healthcare system [9,10,11], there is an urgent need to explore effective approaches aimed at preserving or restoring muscle mass in elderly people.

The main factor behind the development and progression of sarcopenia is thought to be a reduced muscle protein synthesis response (i.e., myogenesis) and a negative muscle balance in older adults [12,13]. In addition, sarcopenia is commonly recognized as a disease with multiple causes, stemming from various physical, psychological, and social factors. Among these, malnutrition and physical inactivity are major contributors that greatly impact muscle protein turnover in older adults [14]. As a result, numerous authors have emphasized the need for a comprehensive tool that could assist with the diagnosis or the assessment of sarcopenia risk factors [15,16,17]. The main goals for the management of sarcopenia is to optimize muscle health by enhancing myoprotein catabolism and increasing muscle function. Accordingly, an effective approach to managing sarcopenia involves a composite of strategies with proper nutrition and regular physical activity being the most commonly recommended [18,19,20].

The exercise therapy commonly employed for enhancing physical activity includes resistance-based exercise training (RET), aerobic exercise training (AET), and its composite regimen (i.e., multi-composited exercise training, MET), which enable elder individuals to counteract sarcopenia [21]. Among the variety of training modalities, resistance exercise with moderate to high intensity exerts promising increases in lean mass and muscular strength [22,23], despite the low compliance for most older individuals, especially the frail elderly. Therefore, other non-resistance exercise with low-load training may be used as alternatives to enhance physical performance in elderly [24].

Aside from regular physical activity, targeted nutritional strategies like specific diets and nutrient supplements aim to help prevent and delay the onset of sarcopenia-related conditions in older adults [25,26,27]. A protein-rich diet or targeted protein supplements are believed to provide additional benefits that could enhance the results of exercise training for healthy older adults [28,29,30,31], as well as for those at higher risk of sarcopenia [26,29,30,31,32,33,34,35,36,37]. Sufficient protein intake plays a key role in triggering the maximum rate of muscle protein synthesis, which promotes anabolism and the build-up of new muscle proteins during RET [38,39]. Over time, this accumulation of muscle protein leads to gains in muscle mass and noticeable hypertrophy. As sarcopenia becomes increasingly common in older adults, it is crucial to figure out the optimal approach for older individuals who are at high risk of muscle atrophy and physical decline.

Muscle turnover depends on the rate of protein synthesis after ingestion, which is likely influenced by both the intake amount of protein and the type of protein source [40]. Plenty of protein nutrients are available from animal-based sources like milk (whey, casein) or meat (beef, chicken, fish, etc.) and plant-based sources like soy, oat, or rice, most of which effectively facilitate post-exercise myofibrillar protein synthesis [41,42,43,44]. Since different protein supplements vary in leucine content and digestion rate, the muscle protein synthesis rates after consuming such protein supplements can differ following exercise training. Previous research has indicated that whey supplements boost post-meal muscle protein synthesis rates more than soy [45] and casein [45,46], especially in older individuals [47,48]. In addition, soy intake after RET stimulates muscle protein synthesis more than casein [45], while milk consumption has a greater effect compared to beef protein [49] and soy supplements [50], respectively. It is reasonable to expect that these short-term benefits from the protein supplementation could lead to positive long-term adaptations in skeletal muscle from training. However, it remains unclear whether the different protein supplements produce distinct long-term effects on muscle health after exercise intervention. Comparing relative effective among different protein supplements can help clinicians make better decisions and set timely intervention strategies.

A number of systematic reviews and network meta-analysis (NMA) studies have investigated the comparative effects between protein sources for young and older people [30,48,51,52,53,54,55,56], and results generally show comparable effectiveness for lean mass or strength gains while comparing whey with soy [51,52,53,56], milk [30,56], and beef [54,56] during an exercise intervention; similar results are observed when comparing soy with blended proteins [51,56]. However, some other protein supplies have not yet been fully compared with former protein supplements in an NMA study, such as collagen, rice, and oat as well as high-protein meals enriched with blended animal and plant protein in daily diets. Therefore, the purpose of this NMA study was to (1) investigate the comparative treatment effectiveness across all combined regimens composed of different types of protein supply and training modality (i.e., RET, AET, and MET); (2) compare the substantial moderators that influence effectiveness; and (3) determine the optimal treatment regimen by ranking the probability of all cross-combined treatments for older adults.

## 2. Materials and Methods

### 2.1. Study Protocol and Registration

This study followed the PRISMA Extension Statement for Network Meta-Analyses, making adjustments as necessary [57]. The protocol of this systematic review has been submitted to the PROSPERO registry (registration number: CRD42023472942).

### 2.2. Literature Search Strategy

An elaborate search for eligible articles was carried out using a variety of online databases—namely MEDLINE Complete, Physiotherapy Evidence Database (the PEDro), the Cochrane Library, Google Scholar, EMBASE, and the China National Knowledge Infrastructure—from its inception until December 2025. Manual searches were also carried out in relevant systematic reviews to identify eligible references. To reduce publication and language biases, there were no restrictions on publication year or language.

The inclusion criteria, based on the PICOS (Participants, Interventions, Comparisons, Outcomes, and Study Design) framework, were outlined in Table 1. The study population was specified using the keywords as follows: (“older adult” OR “elder individual” OR “middle aged adult”) AND (“untrained” OR “sedentary” OR “frailty” OR “sarcopenia”). The protein supplementation and exercise intervention were specified using the keywords as follows: (“resistance exercise training” OR “strengthening exercise”) AND (“protein supplementation” OR “high-protein diet”). The detailed search formulas for each database are presented in Supplementary Table S1.

We used an online platform (the Covidence electronic workflow platform [58]) to screen and assess the identified studies. Eligibility was assessed independently by four reviewers (CDL, HCC, SWH, and CLL) with disagreements resolved by consensus. Initially, titles and abstracts were evaluated in accordance with the PICOS eligibility. The full texts of all eligible articles were retrieved and further examined independently by all reviewers to ensure their inclusion in the study.

### 2.3. Study Selection Criteria

Trials were included if they met the following criteria (Table 1): (1) the trial design was a randomized control or crossover trial (RCTs); (2) participants were community-dwelled, institutionalized or hospitalized older adults who were untrained, suffered acute onsets, or had chronic conditions; (3) at least one study group received protein supplementation plus exercise which was employed as the primary treatment; (4) the comparator group only received protein supply or exercise (with placebo supplementation or not); (5) the control group, receiving placebo supplementation alone or regular care (RC) not related to any protein supply or exercise, was defined as the reference arm in this NMA; and (5) the trial reported at least one of the main outcome measures refer to sarcopenia indices (defined in Section 2.4).

### 2.4. Main Outcomes Refer to Treatment

The main treatment outcomes in relation to protein supplementation and exercise interventions were measurements of muscle mass, strength, and physical mobility (Table 1); all were recognized as indicators of sarcopenia by the Asian [1] and the European [2] recommendations for managing sarcopenia in elder individuals. The primary measures referring to muscle mass were as follows: fat-free mass, whole body lean mass, appendicular lean mass, muscle cross-sectional area, muscle volume, and muscle thickness. For muscle strength of arm, handgrip strength was prioritized for assessment [2]. The leg strength results were gathered as follows: peak torque or power followed by maximum voluntary isometric contraction of the leg muscles. Physical mobility was assessed by gait speed, chair stand, timed up-and-go task (TUG), as well as the Short Physical Performance Battery (SPPB) score. All of the negative scores that indicated improved changes were transformed to positive scores (i.e., a higher changed score was considered more favorable).

### 2.5. Data Extraction

The fields captured from each included trial were as follows: (1) the author’s last name, publication year, country, study arm, study sample, participants’ mean age, body mass index (BMI), and sex distribution; (2) intervention protocol of the exercise and PS; (3) follow-up time frames; and (4) measures of the treatment outcomes. Two reviewers (CDL and CLL) independently extracted details from the included trials, which were confirmed by the third reviewer (SWH). Any disagreements were resolved through the consensus procedure.

If the trial reported different intensities or involved a bilateral side related to the hands or legs, the treatment effects of both sides were combined into a single overall effect as recommended by the Cochrane Handbook [59]. The follow-up time frame was categorized as short term (≤3 months), medium term (>3 months and ≤6 months), or long term (>6 months) for subgroup analysis. If multiple time points fell within the same category, the longest one was used for analysis. For example, if muscle strength was measured at 6, 9, and 12 months, the 12-month data were considered the long-term result. Treatment feasibility and safety were evaluated by examining compliance and any adverse events reported in the included studies.

### 2.6. Quality and Risk of Bias Assessment of Included Studies

Quality assessment was conducted by four reviewers (CDL, HCC, SWH, and CLL) who independently evaluated risk of bias within the included studies by applying the Cochrane Risk of Bias 2 (RoB2) tool [60]. Any disagreements among reviewers’ assessments were discussed at a consensus meeting until an agreement was reached.

The RoB2 tool assesses the risk of bias according to the following six domains alongside seven judgement items related to biased estimates of intervention effects [60]: (1) selection bias (random sequence generation, allocation concealment); (2) performance bias (blinding of participants and personnel); (3) detection bias (blinding of outcome assessors); (4) attrition bias (incomplete outcome data); (5) reporting bias (selective reporting); and (6) other bias referring to other sources of potential bias such as funding or agenda bias [61]. Each item was judged as low risk, unclear risk (i.e., some concerns) or high risk according to criteria specified by Cochrane.

Based on the rated scores across all six domains, the overall bias of each trial was determined to be low (all seven items were rated as low risk), moderate (any one high-risk item with or without unclear-risk judged items), or high risk (two or more high-risk items) [60]. In addition, the individual bias score of each trial was incorporated into the analyses by computing the weighted bias score for each direct estimate in this NMA [62], which was further used for the bias account of a network estimate (described in Section 2.8). Review Manager (RevMan) version 5.4.1 software was used to summarize the results of the risk-of-bias assessment.

Publication bias was assessed using funnel plots and the Begg–Mazumdar rank correlation test [63].

### 2.7. Data Synthesis and Analysis

Due to differences in the tools used to measure treatment outcomes across the trials, standard mean differences (SMDs) with 95% confidence intervals (CIs) were calculated to assess the treatment effect sizes for all outcome measures.

The SMD is essentially an overall estimate of the average difference between the change scores of two study groups. Mean change scores between the post-test and pretest, along with their standard deviations (SD), were recorded directly. If the variance of the paired difference could not be determined, a conservative estimate was made using a within-participant correlation of 0.9 for muscle mass and 0.7 for strength and mobility, as suggested by Rosenthal [64], based on baseline and posttest data. When SD was not reported, it was calculated from p-values or 95% CIs. For data given as the median and interquartile range, the mean was estimated from the median, and the SD was obtained by dividing the interquartile range by 1.35 [65]. The magnitude of the SMD was classified using Cohen’s criteria [66]: small (*d* < 0.5), medium (0.50 ≤ *d* < 0.80), and large (*d* ≥ 0.80).

Direct and indirect estimates among treatment regimens were computed by running a random-effects NMA model within a frequentist framework. Network heterogeneity and inconsistency were assessed using the I^2^ and Q statistics alongside τ^2^ values to estimate the variance across the studies. The consistency between direct and indirect comparisons was assessed using the node-splitting method [67]. Additionally, a full design-by-treatment interaction random-effects model was established to test global inconsistency across all treatment arms [68]. Ranking probabilities of effect estimation among treatments per outcome were expressed using the surface under the cumulative ranking (SUCRA) score [69].

Network meta-regression (NMR) analyses were performed to determine any relevant moderators that might create heterogeneity across the studies. For each outcome, the NMR model was adjusted by including an individual moderator as a covariate [70]. Potential moderators were identified on the basis of (1) participant characteristics, namely age range, BMI, sex (i.e., proportion of women in the sample), population area, and health status (i.e., relatively healthy versus subhealth); (2) the study methodology, comprising study quality, and follow-up period; and (3) the intervention design, including protein source (i.e., animal, plant, and mixed), training type (i.e., RET, AET, MET), and treatment time period. The NMR results were expressed as *β* alongside 95% credible interval (CrI).

Furthermore, compliance and adverse effects were measured in terms of the occurrence rate of all-cause withdrawal and adverse events, respectively. The results of the analyses were expressed as odds ratios (ORs) alongside 95% CIs.

NMA was conducted using R statistical software (version 4.2.3, R Foundation for Statistical Computing, Vienna, Austria) [70,71]. *p* values < 0.05 were statistically significant.

### 2.8. Certainty of Evidence

The Grading of Recommendation Assessment, Development, and Evaluation (GRADE) framework was used to determine the quality of evidence contributing to network estimates per main outcome [72]. The GRADE profile allows the reviewers to rate the evidence as high, moderate, low, or very low quality. The assessment procedures were carried out independently by the reviewers (CDL, HCC, THL, CLL, SWH).

Additionally, we assessed the weighted contribution of direct comparisons to the NMA estimates for each outcome using the contributions matrix [62,73,74]. Firstly, the risk-of-bias assessment for each direct pairwise comparison was performed by the weighted computation of the individual bias score of each included trials [73]. In the following, the contributions matrix was used to calculate the percentage contribution of all direct comparisons to the NMA estimates, indicating the contribution of direct evidence to the entire network [62,73,74]. Finally, the downgrading level for the study limitation was based on the amounts of contribution of each direct estimate and its corresponding risk-of-bias score to the NMA estimates [74].

## 3. Results

### 3.1. Trial Selection Flowchart

**Figure 1:**
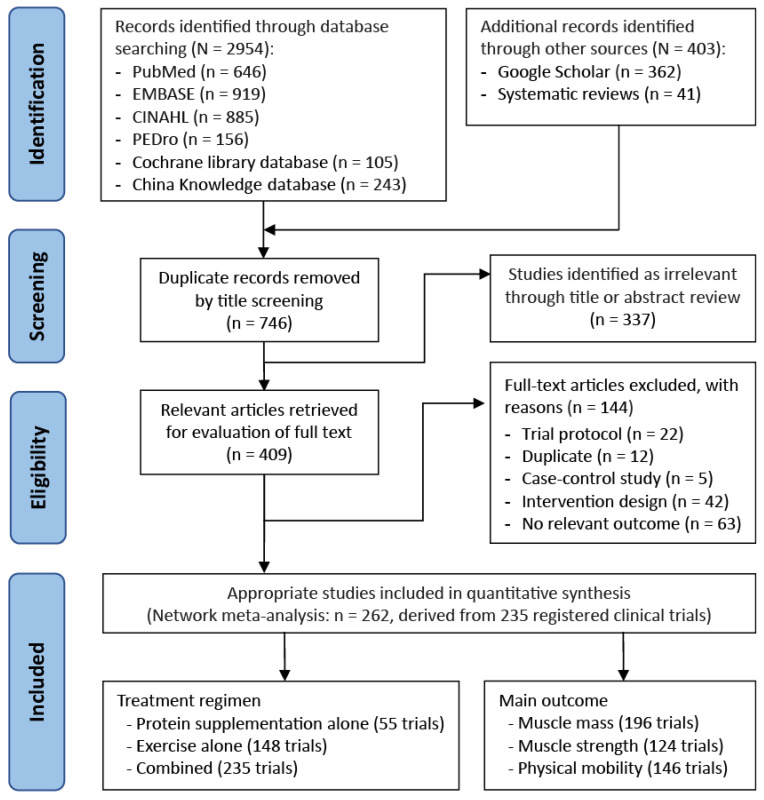
Preferred Reporting Items for Systematic Reviews and Meta-analysis flow diagram for literature search and study screening.

Figure 1 shows a flowchart of the trial selection process. Initially, a total of 2954 articles were identified through an online and manual literature search. Once duplicates were removed, we reviewed the titles and abstracts of 746 articles to assess their eligibility; in the following, 409 were further assessed by full-text review (Figure 1). Finally, we included 235 registered clinical RCTs, corresponding with 262 articles published between 1994 and 2023.

### 3.2. Study Characteristics

Table 2 summarizes the participant demographic data and study characteristics of the included RCTs. The detailed information are presented in the Supplementary Table S2. A total of 20,980 participants were enrolled in all of the included RCTs. In overall, the sample had a mean age of 73.2 (range: 50.0–89.2) years and a mean BMI of 25.6 (range: 16.5–34.9) kg/m^2^. Among the 235 included RCTs (Table S2), 161 enrolled both men with an average proportion of female participants being 57.9% (range: 10.0–95.7%) and the others had a sex-specific study design that only enrolled men (37 trials) or women (37 trials).

With respective to participants’ physical conditions, 52 out of 235 RCTs enrolled older adults who were physically independent, whereas others recruited individuals who were identified as frail (54 trials), experiencing sarcopenia (76 trials), experiencing dynapenia (15 trials), and sedentary or inactive (38 trials). Additionally, the majority of the participants (74.9% in 176 RCTs) were medically stable, whereas other trials enrolled older people who suffered acute or chronic conditions.

Moreover, most of the study population (71.9%) was enrolled in Asia (89 trials) and Europe (80 trials) followed by the America (52 trials) and Oceania areas (14 trials; Table 2 and Table S2).

Summarizing the intervention designs across the included trials (Table 2), a total of 12,224 participants received PS with exercise (50.2%, 235 RCTs) or without (8.0%, 55 RCTs), whereas 21.3% of the participants (4462 in 148 RCTs) received exercise alone, and the other 20.5% (4294 in 95 RCTs) received RC without any PS or exercise interventions. In addition, nearly 95% of the trials employed an intervention period of 1–36 weeks, and 14 RCTs conducted a long-term intervention of 12–36 months. Regarding the follow-up duration for outcome assessment, 169 out of the 235 included RCTs conducted a short-term follow-up duration of ≤12 weeks, 89 RCTs had a medium-term follow-up duration of 13–36 weeks, and 19 RCTs investigated a long-term outcome over a period of 12–36 months (Table S2).

### 3.3. Protein Supplementation Characteristics

The prescriptions for the PS of each trials are presented in Supplementary Table S2. In the current NMA, a total of 10 types of protein-based supplement or diet were employed by the trials (Table 1), namely whey protein (WP, 111 RCTs); milk protein (MP, 56 RCTs); soy protein (SP, 28 RCTs); collagen (6 RCTs); casein (10 RCTs); meat (9 RCTs); rice protein (4 RCTs); insect protein (1 RCT); oat (1 RCT); and protein-enriched meals (50 RCTs). There was a variety of PS protocol employed among trials; in summary, 43% (101 RCTs) of the included trials prescribed supplementation before or following exercise on the training days by a mean dosage of 25 g (range 10–60 g); and the other RCTs prescribed daily supplementation by a mean ingestion amount of 28.5 g (range 10–85 g) as well as protein-enriched meals (0.8-1.8 g per body weight in kg) during an exercise intervention.

The detailed information indicating dosage for each protein source is presented in Table S2. In summary, PS was prescribed by an average amount of 25.8 g (range 4–75 g) for whey protein, 22 g (range 4–43 g) for milk protein; 23 g (range 7–40 g) for soy protein, 19 g (range 9–40 g) for collagen; 20 g (range 10–40 g) for casein; 31 g (range 5–45 g) for meat, and 13 g (range 3–40 g) for rice protein.

### 3.4. Exercise Training Protocol

The summarized protocol of exercise in each RCT is presented in Table S2. Among the included trials, 130 (55.3%) employed RET, another 21 (8.9%) conducted AET, and the others (84 RCTs, 35.8%) used a combined regimen (i.e., multicomponent exercise training, MET) comprising two or more of the following exercise modalities: RET, AET, balance, or function mobility training.

The detailed information indicating dosage for each exercise is presented in Table S2. In summary, the majority of the included trials (155 RCTs) employed RET by a moderate to high training load (≥70%), one-repetition maximum (range 70–85%) or a perceived exertion of 6–9 out of a 10-point rating. In addition, 43 RCTs employed AET by a moderate to high intensity ≥ 70% (70–90%) maximal heart rate or peak oxygen intake, as well as a perceived exertion of 6–8 out of a 10-point rating. A total of 90 RCTs prescribed low intensity for RET, AET, or MET. Regarding the training duration, overall, 149 out of 235 RCTs conducted a short-term or medium-term training period ≤ 12 weeks (range 2–12 weeks) with a total of 6–84 training sessions (frequency, 2–7 sessions per week); the other 86 RCTs had an intervention period longer than 3 months (range 4–24 months) with a total of 25–1008 training sessions (frequency, 3–14 sessions per week).

### 3.5. Assessment of Transitivity Across Treatment Arms

In this paper, a total of 36 treatment options derived from 10 protein sources and 3 exercise types were identified for our NMA. Each of the identified treatment regimens and its abbreviation is listed in Table 3.

The assessment of transitivity indicated that the distribution of relevant variables of baseline clinical condition (healthy versus relatively healthy individuals), setting (community-dwelling versus institutionalized), and main intervention characteristics were generally balanced across treatment comparisons (Table 4). The baseline clinical conditions were comparably distributed, with relatively healthy older adults in approximately 75–85% of the trials across all major treatment arms. Regarding the clinical settings, community-dwelling populations accounted for approximately 80% of the trials across treatment arms.

Intervention characteristics, including protein dosage, exercise intensity, and intervention duration showed no systematic variation that would preclude cross-treatment comparisons (Table 4). These findings suggest that the transitivity assumption was likely satisfied, allowing for valid indirect comparisons within the network.

### 3.6. Risk of Bias in Included Trials

**Figure 2:**
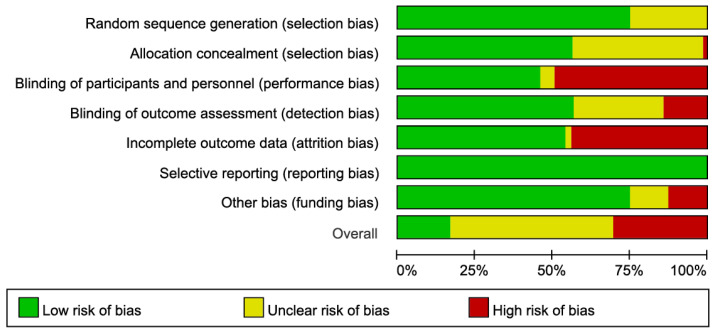
Risk of bias graph crossing trials.

Figure 2 presents a summary of ranking score for each risk-of-bias item and an overall rank crossing the included RCTs. The details of all ranked risk-of-bias items within each trial are presented in the Supplementary Figure S1. Using the Cochrane risk-of-bias tool, 70 out of 235 (29.8%) trials were ranked as having an overall high risk of bias, whereas it determined 39 RCTs (16.6%) had low risk of bias. Over half of the included trials (126 RCTs) with an overall moderate risk of bias were susceptible to some bias but probably not sufficient to invalidate the results.

Several biases commonly contributed to an overall high risk of bias ranked across all RCTs, which included at least one of the following: performance, detection, attrition and other (i.e., agenda or sponsor) biases (Figure 2). In brief, 115 (48.9%) and 32 (13.6%) out of 235 RCTs were judged having a high risk of performance and detection biases, respectively, due to the unmasked participants or assessors. Additionally, 102 RCTs (43.4%) which did not perform intention-to-treat analyses were scored as having a high risk of attrition bias.

Regarding the domain of other bias, we assumed that the use of supplement products which are supported by any grants or provided by financial sponsors may indicate a potential funding bias; accordingly, the information related to financial resources as well as declarations of conflicts of interest were assessed. There was a potentially high risk of funding bias in 29 RCTs (12.3%) which reported financial sources without any conflict-of-interest disclosures, and 30 RCTs (12.8%) that had neither information of any funding source nor interest disclosure were considered as having an unclear risk of financial bias (Figure S1).

### 3.7. Effectiveness of Treatment for Muscle Mass

**Figure 3:**
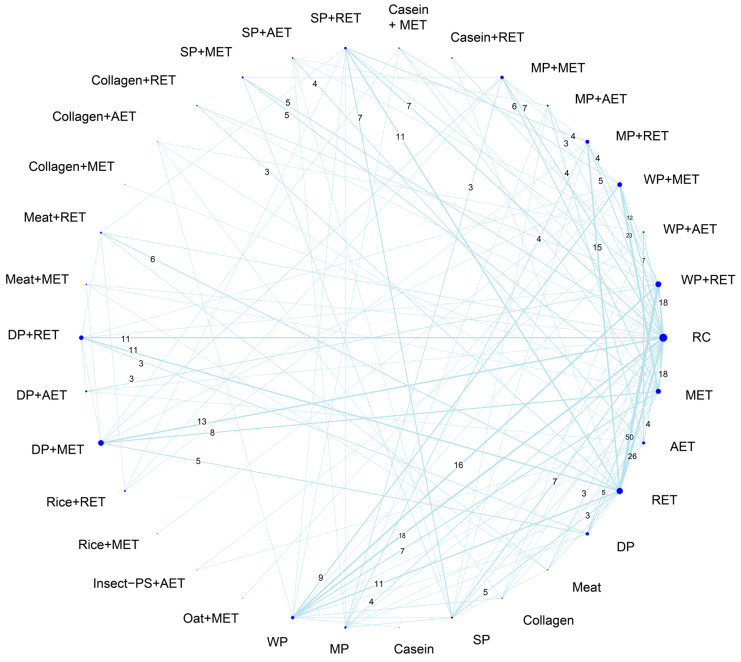
Network evidence geometry for muscle mass outcome. The numbers along the lines indicate the total direct comparisons, displaying only those with three or more. The width of each line indicates the weighted edge proportional to the number of studies. The size of each dot indicates the sample size proportional to the number of the participants. AET, aerobic exercise training; DP, dietary protein; Insect-PS, insect protein supplement; MET, multicomponent exercise training; MP, milk protein; RET, resistance exercise training; SP, soy protein; WP, whey protein; RC, regular care.

All of the outcome measures across trials are presented in Supplementary Table S3. The muscle mass was mostly measured by dual-energy X-ray absorptiometry (153 RCTs) or bioelectrical impedance analysis (33 RCTs), which were expressed as absolute whole-body lean mass, appendicular lean mass, or fat-free mass as well as its indices. In addition, muscle volume was expressed as cross-sectional area measured by magnetic resonance imaging (30 RCTs), muscle thickness measured by ultrasonography (19 RCTs), and the muscle girth (14 RCTs) of arms or legs. When the trial reported multiple measures, priority for analysis was given to the following: lean body mass, fat-free mass, appendicular lean mass, cross-sectional area, muscle thickness, and girth. In total, muscle mass measures were employed by 211 RCTs along with 34 treatment arms and 494 pairwise comparisons in our NMA. The eligible comparisons among all treatment options are presented in the network geometry shown in Figure 3.

#### 3.7.1. Pairwise Meta-Analysis for Muscle Mass 

Direct compar isons of pairwise meta-analyses indicated that PS plus RET achieved favorable effects on increasing muscle mass compared with RC, particularly WP, MP, SP, Collagen, and DP (SMD = 0.89–2.08, all *p* < 0.05; Supplementary Table S4). Similar results were observed in WP+MET (SMD = 0.85, 95% CI: 0.48–1.26), SP+MET (SMD = 0.65, 95% CI: 0.03–1.26), and MP+AET (SMD = 1.26, 95% CI: 0.33–2.19). In addition, the combined treatments WP+RET (SMD = 0.67) as well as MP+RET (SMD = 0.60) and SP+RET (SMD = 0.54) exhibited greater effects on muscle mass gain than the monotherapy of RET did (all *p* < 0.05).

#### 3.7.2. Global Effects in NMA for Muscle Mass

**Figure 4:**
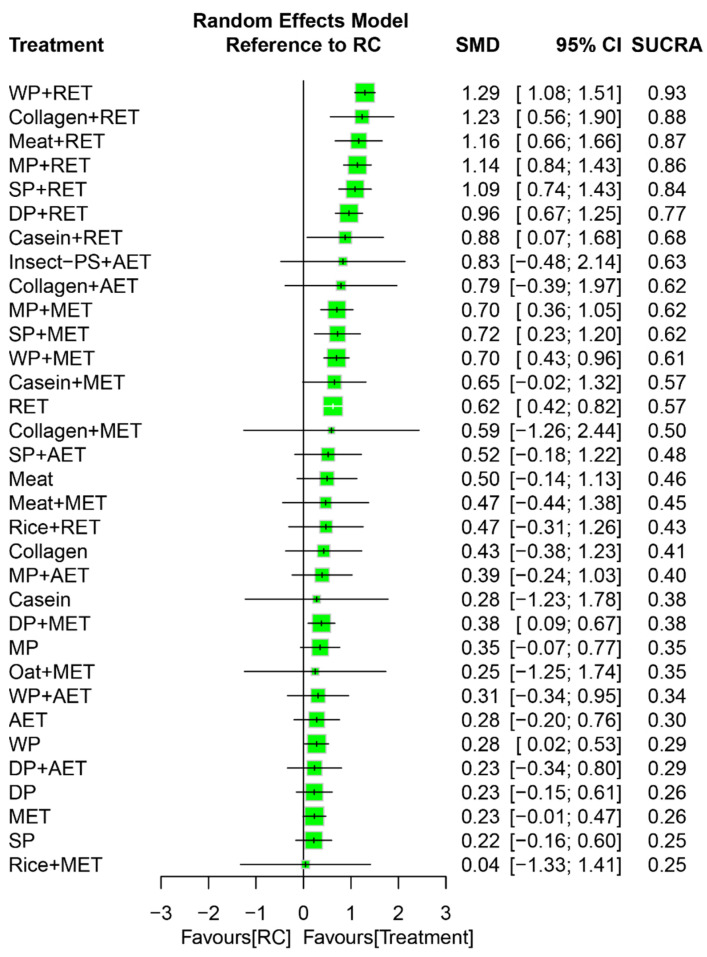
Network effects for muscle mass gain during an overall follow-up duration. The size of each square represents the weight of the effect size proportional to the number of analyzed studies and participants. AET, aerobic exercise training; DP, dietary protein; Insect-PS, insect protein supplement; MET, multicomponent exercise training; MP, milk protein; RET, resistance exercise training; SP, soy protein; WP, whey protein; RC, regular care.

Counting together by the direct and indirect estimates in our NMA, using RC as the common reference, PS combined with RET (SMD = 0.88–1.29) generally achieved superior efficacy on muscle mass gain compared with the combined treatment of MET (SMD = 0.38–0.70) or AET (SMD = 0.31–0.83; Figure 4) regardless of follow-up duration.

Pooling all treatment effects in the NMA, WP+RET was ranked as the most effective (SUCRA = 0.93) by a large effect (SMD = 1.29) among all treatment arms for muscle mass gain followed by regular Collagen+RET (SUCRA = 0.88; SMD = 1.23) and Meat+RET (SUCRA = 0.87; SMD = 1.16; Figure 4). The global heterogeneity of the NMA model for muscle mass was significant across treatment arms (*τ*^2^ = 0.39, *I*^2^ = 78.6%, *p* < 0.0001). Q statistic results assessing the total consistency between designs in the NMA were shown to be significant under the assumption of a full design-by-treatment random-effects model (*τ*^2^ = 0.27, *p* < 0.001). The node-splitting results showed significant inconsistencies in the treatment arms of WP+AET and MP+AET, compared with RC, by differences between the direct and indirect estimates of −3.60 (*p* < 0.0001) and 1.59 (*p* < 0.05), respectively (Supplementary Figure S2).

#### 3.7.3. Subgroup Analysis of Follow-Up Duration for Muscle Mass

The combined treatment WP+RET was ranked as the optimal treatment for muscle mass over the short-term (SMD = 1.24, SUCRA = 0.91), medium-term (SMD = 1.26, SUCRA = 0.91) and long-term (SMD = 0.81, SUCRA = 0.85) follow-up durations (Supplementary Figure S3).

### 3.8. Effectiveness of Treatment for Muscle Strength

**Figure 5:**
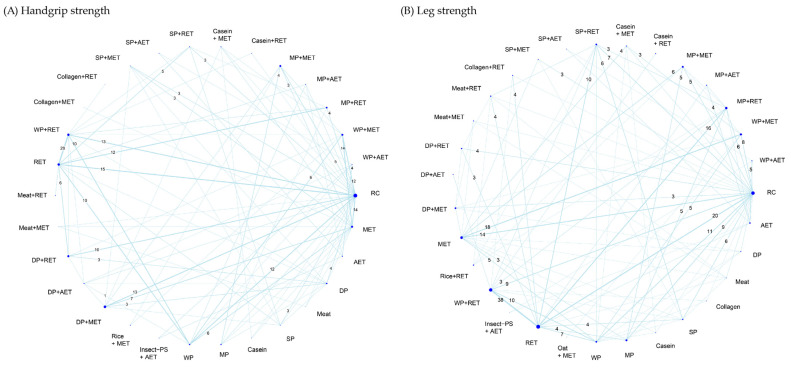
Network evidence geometry for (**A**) handgrip strength and (**B**) leg strength. The numbers along the lines indicate the total direct comparisons, displaying only those with three or more. The width of each line indicates the weighted edge proportional to the number of studies. The size of each dot indicates the sample size proportional to the number of the participants. AET, aerobic exercise training; DP, dietary protein; Insect-PS, insect protein supplement; MET, multicomponent exercise training; MP, milk protein; RET, resistance exercise training; SP, soy protein; WP, whey protein; RC, regular care.

Muscle strength outcomes were assessed in terms of handgrip strength and leg strength. Handgrip strength as well as leg strength was mostly measured by a handled dynamometer and expressed as maximal voluntary isometric contraction; other leg-strength measures included leg press (1, 5, 10 repetition maximum) and isokinetic knee extension (Table S3). A total of 133 RCTs reported treatment outcome of handgrip strength along with 30 treatment options and 308 pairwise comparisons in the NMA (Figure 5A). The treatment effects on leg strength were investigated by 129 RCTs along with 31 treatment options and 351 pairwise comparisons in the NMA (Figure 5B).

#### 3.8.1. Pairwise Meta-Analysis for Muscle Strength

Direct comparisons of pairwise meta-analyses indicated that PS plus RET achieved favorable effects on handgrip strength gain compared with RC (Supplementary Table S5), particularly WP, SP, and DP (SMD = 0.62–1.07, all *p* < 0.05); similar results were observed in WP+MET (SMD = 0.62, 95% CI: 0.25–0.99) as well as DP+AET (SMD = 1.37, 95% CI: 0.22–2.51). In addition, the combined treatment SP+RET yielded greater changes in handgrip strength compared with the monotherapy of SP alone (SMD = 1.43, 95% CI: 0.59–2.28) or RET alone (SMD = 0.51, 95% CI: 0.01–1.01); similar results were observed for the comparison between WP+RET and WP alone (SMD = 0.73, 95% CI: 0.38–1.08; Table S5).

In leg strength gain, PS plus RET achieved favorable effects compared with RC (Supplementary Table S6), particularly WP, MP, SP, and DP (SMD = 0.76–1.54, all *p* < 0.05). Additionally, the combined treatments WP plus RET as well as MET yielded greater strength gains than the monotherapy of WP (SMD = 1.18 and 1.53, respectively) or exercise (SMD = 0.44 and 0.63, respectively); similar results were observed in SP+RET when it was compared with SP alone (SMD = 1.58, 95% CI: 0.96–2.19; Table S6).

#### 3.8.2. Global Effects of NMA for Muscle Strength

**Figure 6:**
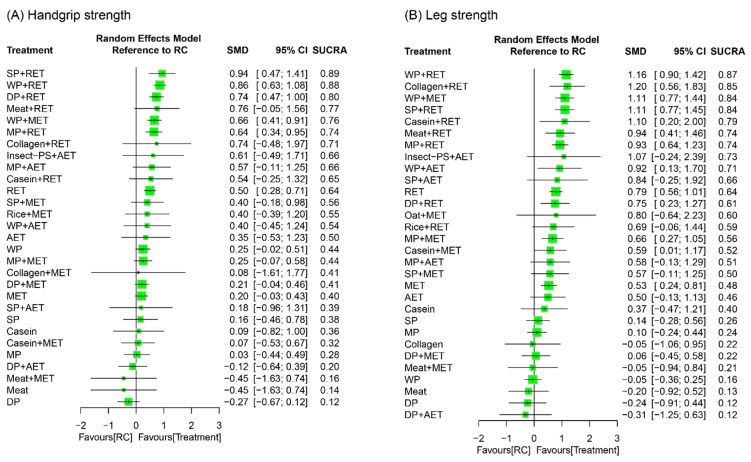
Network effects of treatment regimens on (**A**) handgrip strength and (**B**) leg strength. The size of each square represents the weight of the effect size proportional to the number of analyzed studies and participants. AET, aerobic exercise training; DP, dietary protein; Insect-PS, insect protein supplement; MET, multicomponent exercise training; MP, milk protein; RET, resistance exercise training; SP, soy protein; WP, whey protein; RC, regular care.

The NMA results for handgrip strength showed that PS plus RET as well as MET exerted significant effects on strength gain relative to RC during the overall follow-up duration (Figure 6A)—particularly the protein sources derived from whey, soy, and milk (SMD = 0.57–0.94, *p* < 0.05). Among all treatment options, SP+RET was ranked the most effective (SUCRA = 0.86) for muscle strength—followed by WP+RET (SUCRA = 0.85) and DP+RET (SUCRA = 0.76)—during the overall follow-up duration (Figure 6A). The global heterogeneity of the NMA model for handgrip strength was significant (*τ*^2^ = 0.25, *I*^2^ = 76.8%, *p* < 0.0001). When assuming a full design-by-treatment random-effects model, the overall between-design inconsistency was shown to be significant (*τ*^2^ = 0.15, *p* < 0.0001). The node-splitting results showed significant inconsistencies in the treatment arms of DP+AET and MET, compared with RC, by differences between the direct and indirect estimates of 2.18 (*p* < 0.0001) and 0.49 (*p* < 0.05), respectively (Supplementary Figure S4).

Regarding strength gain in the lower extremity (Figure 6B), the NMA results showed that most of the PS treatments combined with RET as well as MET obtained favorable effects relative to RC, including protein sources of WP, SP, MP, casein, meat, collagen, and DP (SMD = 0.64–1.23) during an overall follow-up duration. In addition, WP+RET as well as WP+MET was ranked the most effective (both SUCRA = 0.86) for leg strength gain followed by SP+RET (SUCRA = 0.85; Figure 6B). The global heterogeneity of the NMA model for leg strength was significant (*τ*^2^ = 0.33, *I*^2^ = 70.1%, *p* < 0.0001). Under the assumption of a full design-by-treatment interaction random-effects model, there was significant inconsistency between designs in our NMA (*τ*^2^ = 0.16, *p* < 0.0001). The node-splitting results showed significant inconsistencies in the composite SP+RET, compared with RC, by a difference between the direct and indirect estimates of 0.79 (*p* < 0.05; Supplementary Figure S5).

#### 3.8.3. Subgroup Analysis of Follow-Up Duration for Muscle Strength

In handgrip strength, SP as well as MP in combination with RET achieved the highest treatment efficacy over a short-term (SMD = 1.29, SUCRA = 0.92) and medium-term (SMD = 0.35, SUCRA = 0.89) follow-up period, respectively (Supplementary Figure S6). Within the long-term time frame, WP+MET exhibited the most effective among whole treatment regimens in our NMA (SMD = 0.97, SUCRA = 0.95).

For leg strength gain, the combined regimen SP+RET was ranked as the optimal treatment option during the short-term (SMD = 1.17, SUCRA = 0.85) as well as the long-term (SMD = 1.34, SUCRA = 0.93) follow-up period, whereas MP+RT was ranked the most effective among whole treatment regimens during the medium-term (SMD = 1.09, SUCRA = 0.83) follow-up time frames (Supplementary Figure S7).

### 3.9. Effectiveness of Treatment for Physical Mobility

**Figure 7:**
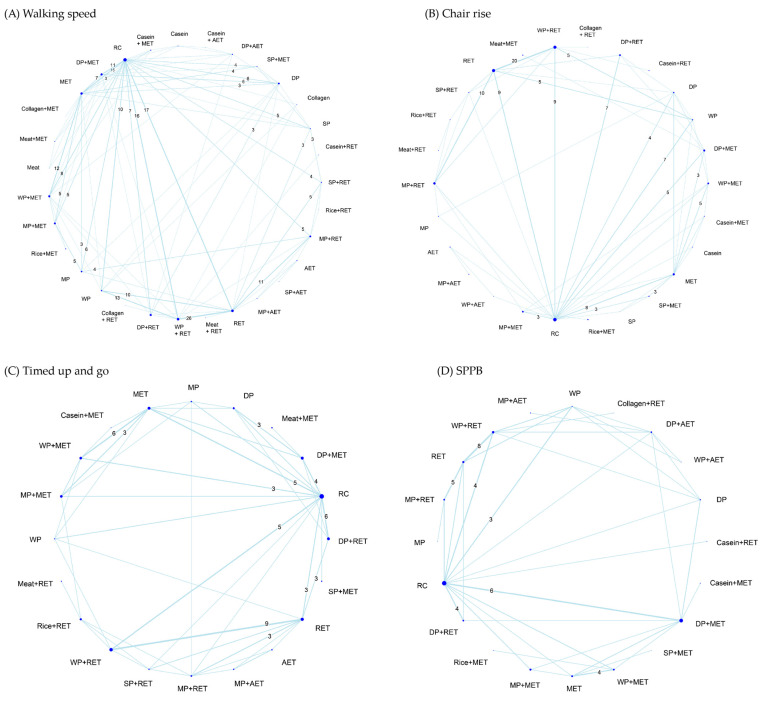
Network evidence geometry for (**A**) walking speed, (**B**) chair rise, (**C**) timed up and go, and (**D**) Short Physical Performance Battery scores. The numbers along the lines indicate the total direct comparisons, displaying only those with three or more. The width of each line indicates the weighted edge proportional to the number of studies. The size of each dot indicates the sample size proportional to the number of the participants. AET, aerobic exercise training; DP, dietary protein; MET, multicomponent exercise training; MP, milk protein; RET, resistance exercise training; SP, soy protein; SPPB, Short Physical Performance Battery; WP, whey protein; RC, regular care.

Treatment efficacy on physical mobility was assessed by walking speed (126 RCTs), chair rise (68 RCTs), TUG (47 RCTs), and global function in terms of SPPB score (49 RCTs; Table S3). In walking speed, a total of 31 treatment options were identified along with 312 pairwise comparisons in the NMA (Figure 7A). In chair rise (Figure 7B), a total of 26 treatment options (157 pairwise comparisons) were identified in the NMA. In TUG (Figure 7C), a total of 20 treatment options (96 pairwise comparisons) were identified in the NMA. For SPPB, 20 treatment options were identified along with 74 pairwise comparisons in the NMA (Figure 7D).

#### 3.9.1. Pairwise Meta-Analysis for Physical Mobility

Direct comparisons of pairwise meta-analyses indicated that PS plus RET as well as MET generally achieved favorable effects on walking speed (SMD = 0.44–1.23; Supplementary Table S7), chair rise (SMD = 0.54–1.09; Supplementary Table S8), TUG (SMD = 0.81; Supplementary Table S9), and SPPB score (SMD = 0.77–1.04; Supplementary Table S10) compared to RC, particularly the protein sources derived from WP, MP, SP, and DP. Additionally, the protein sources of WP, SP, and DP plus RET achieved greater effects on chair rise (SMD = 1.03), walking speed (SMD = 0.77), and SPPB (SMD = 1.36), respectively, than PS monotherapy (all *p* < 0.05). Furthermore, the combined treatment WP+MET exhibited greater effects on walking speed (SMD = 0.41) and chair rise (SMD = 0.98) than MET monotherapy; similar results were observed in MP+MET for CR (SMD = 0.50).

#### 3.9.2. Global Effects of NMA for Physical Mobility

**Figure 8:**
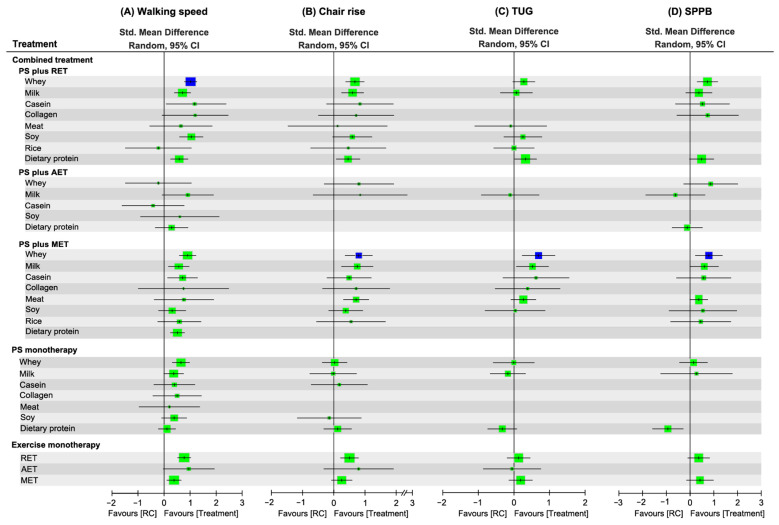
Network effects of treatment regimens on changes in (**A**) walking speed; (**B**) chair rise; (**C**) TUG; and (**D**) SPPB scores. The size of each square represents the weight of the effect size proportional to the number of analyzed studies and participants. The blue-colored point estimate indicates the highest rank of probability. AET, aerobic exercise training; Std., standardized; 95% CI, 95% confidence interval; DP, dietary protein; MET, multicomponent exercise training; MP, milk protein; PS, protein supplementation; RET, resistance exercise training; SP, soy protein; SPPB, Short Physical Performance Battery; TUG, timed up and go; WP, whey protein; RC, regular care.

Figure 8 summarizes the network comparative results among treatment options (using RC as reference) during an overall follow-up duration for all mobility outcomes; and the details are presented in Supplementary Figures S8–S11. Overall, PS in combination with MET as well as RET achieved superior effects on mobility outcomes compared with its composite of AET, especially the protein derived from whey (in chair rise and SPPB), casein (in walking speed), milk (in all mobility measures), soy (in walking speed), and DP (in walking speed and SPPB).

Among all treatment options, WP+RET was ranked the most effective treatment to increase walking speed (SMD = 1.08; SUCRA = 0.85; Figure S8). In addition, WP+MET yielded the highest probability of treatment efficacy on chair rise (SMD = 1.09; SUCRA = 0.87; Figure S9), TUG (SMD = 0.70; SUCRA = 0.90; Figure S10), and SPPB (SMD = 1.02; SUCRA = 0.86; Figure S11).

The global heterogeneity of the NMA model was significant in walking speed (*τ*^2^ = 0.28, *I*^2^ = 75.7%, *p* < 0.0001), chair rise (*τ*^2^ = 0.23, *I*^2^ = 72.9%, *p* < 0.0001), TUG (*τ*^2^ = 0.14, *I*^2^ = 67.9%, *p* < 0.0001), and SPPB (*τ*^2^ = 0.26, *I*^2^ = 80.9%, *p* < 0.001). Using a full design-by-treatment random-effects model, we found an overall between-design inconsistency in the NMA for walking speed (*τ*^2^ = 0.17, *p* < 0.01), but this was not statistically significant for the other mobility outcomes (all *p* > 0.05). For all mobility measures, the node-splitting results for NMA inconsistency showed no inconsistencies between direct and indirect evidence (Supplementary Figures S12–S15).

#### 3.9.3. Subgroup Analysis of Follow-Up Duration for Physical Mobility

During the short-term follow-up duration, WP+RET was ranked as the most effective treatment option to increase walking speed (SMD = 1.16, SUCRA = 0.80; Figure S8), while WP+MET was the most effective for saving time during the chair-rise (SMD = 1.52, SUCRA = 0.97; Figure S9) and TUG (SMD = 0.83, SUCRA = 0.90; Figure S10) tasks, as well as increasing the global SPPB score (SMD = 0.64, SUCRA = 0.74; Figure S11). Regarding the medium-term and long-term outcome, PS plus MET as well as RET was mostly ranked as the optimal treatment for mobility, especially WP, MP, SP, and DP (Figures S8–S11).

### 3.10. Network Meta-Regression Results for Moderators of Treatment Efficacy

The results of network meta-regression analyses are shown in Supplementary Table S11. In muscle mass, there was a potential gender effect on relative treatment efficacy. Older age (*ꞵ* = –0.39; 95% CrI: −0.69, −0.09) as well as female sex (*ꞵ* = −0.33; 95% CrI: −0.60, −0.05) predicted minor effects on muscle mass gain; in addition, those with acute or chronic conditions were more likely to achieve lean mass gain than their healthy peers (*ꞵ* = 0.28; 95% CrI: 0.02, 0.55); furthermore, animal-derived protein was associated with greater muscle mass gain (*ꞵ* = 0.27; 95% CrI: 0.03, 0.50); finally, a greater amount of PS dose may correspond with greater lean mass gains (*ꞵ* = 0.28; 95% CrI: 0.001, 0.57). In muscle strength, BMI (*ꞵ* = −0.26; 95% CrI: −0.52, −0.001) and age (*ꞵ* = −0.42; 95% CrI: −0.76, −0.06) may exert negative effects on relative treatment efficacy for handgrip and leg strength, respectively. In addition, a higher dosage of PS (*ꞵ* = 0.36; 95% CrI: 0.09, 0.64) as well as a longer period of treatment (*ꞵ* = 0.24; 95% CrI: 0.02, 0.45) and follow-up duration (*ꞵ* = 0.22; 95% CrI: 0.01, 0.45) may achieve greater changes in handgrip strength. For function outcome, younger age (*ꞵ* = 0.28; 95% CrI: 0.04, 0.52) and female sex (*ꞵ* = 0.61; 95% CrI: 0.23, 1.18) may have effects on increasing walking speed and chair rise, respectively. No moderator was identified to have an influence on the treatment efficacy regarding mobility tasks of walking speed, chair rise, and timed up-and-go.

### 3.11. Attrition and Side Effects

**Figure 9:**
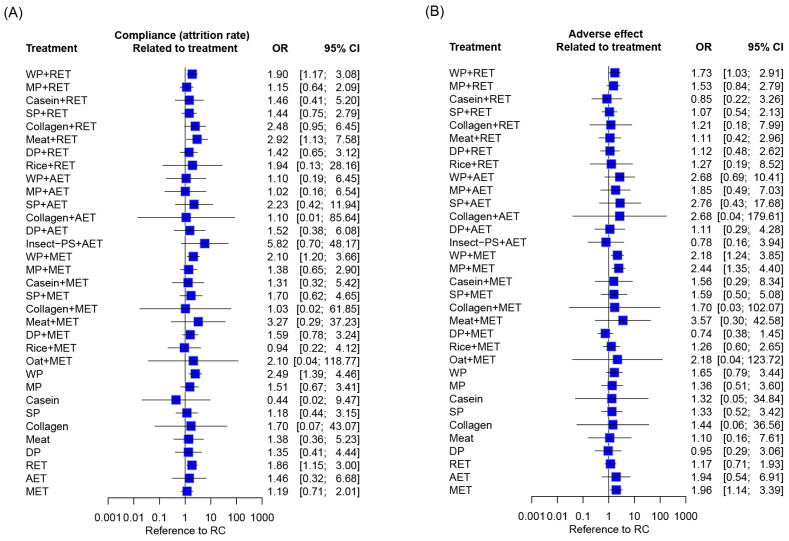
Compliance and adverse effects of treatment regimens. Data are presented as (**A**) all-cause drop-out rate and (**B**) treatment-related adverse events. Each point estimate, shown as a square, represents the combined effect (OR), with the horizontal line indicating the 95% CI. OR, odds ratio; CI, confidence interval; AET, aerobic exercise training; DP, dietary protein; Insect-PS, insect protein supplement; MET, multicomponent exercise training; MP, milk protein; RET, resistance exercise training; SP, soy protein; WP, whey protein; RC, regular care.

In overall, a mean attrition rate of 12.5% (range 0–78.6%) was reported during follow-up on the basis of the analyzed RCTs, among which 2.1% (range 0–45.2%) of participants withdrew because of treatment noncompliance (Table S12). A total of 40 RCTs reported withdrawal of participants due to the noncompliance of PS by an attrition rate of 1.6–39.5%. The noncompliance of exercise training was reported by 50 RCTs with an attrition rate of 1.0–31.3%. The NMA results revealed no difference in treatment-related attrition rate across all the treatment regimens with reference to RC (Figure 9A). Comparing to the common reference of RC, the treatment options WP+RET, Meat+RET, WP+MET, WP, and RET are likely to have higher withdrawal rates (OR: 1.86–2.92).

In total, 32 and 23 out of the 235 analyzed RCTs reported mild adverse events in response to PS and exercise training, respectively (Table S12). There was no serious adverse event reported in relation to PS plus exercise or its monotherapy. Among the reported adverse events, the most common were training-induced joint pain and muscle soreness of short duration during training sessions and gastrointestinal symptoms such as diarrhea, bloating, and constipation after the consumption of PS. Compared with RC, WP+RET, WP+MET, MP+RET, and MET yielded significant risks of adverse effects (OR: 1.73–2.44; Figure 9B).

### 3.12. Publication Bias

The funnel plots indicating publication bias for all outcome domains are presented in Supplementary Figure S16. Based on Begg–Mazumdar test results, there was significant reporting bias for muscle mass (*p* < 0.01; Figure S16A) and leg muscle strength (*p* < 0.01; Figure S16C).

### 3.13. Certainty of the Evidence

Table 5 summarizes the GRADE certainty rating for all main outcomes and the details of judgments in each domain of the GRADE framework are presented in Supplementary Tables S13–S19. Overall, the combined composites MP+RET, WP+RET, and WP+MET obtain high certainty of the evidence for strength and mobility outcomes, particularly handgrip strength, walking speed, and chair rise. (Table 5). In muscle mass, the certainty of the evidence ranged from low to moderate among combined treatment regimen. The most common reasons for downgrading the certainty of evidence were related to major concerns about the study limitations, imprecision, and publication bias.

## 4. Discussion

This paper aimed to identify the comparative efficacy of different protein sources in combination with exercise modalities on sarcopenia-related outcomes in middle-aged and older adults. The main NMA results are presented as follows: (1) the combined composite PS plus RET as well as MET exerted favorable effects on all primary outcomes relative to RC; (2) based on the SUCRA ranking results, WP combined with RET yielded the highest probability in muscle mass and leg strength gain, whereas WP plus MET was determined as the optimal strategy for physical recovery; (3) a number of potential moderators of relative treatment efficacy were identified by a series of NMR analyses, including age, BMI, gender, races, intervention period, and follow-up time; (4) in addition, WP, MP, RET, MET and its combined regimens may exhibit lower compliance and significant minor adverse effects compared to RC despite no serious events occurring in relation to treatments.

This NMA was a meticulous evaluation of the transitivity assumption across a large pool of 235 trials. Although our inclusion criteria were intentionally broad to capture a comprehensive evidence base, our analysis confirmed that the distribution of key effect modifiers was consistent across treatment nodes. Notably, the vast majority of included trials were conducted among community-dwelling individuals, which minimizes potential confounding related to the care setting (e.g., institutionalization vs. independent living). Furthermore, the baseline age and clinical condition of participants was well-balanced across all comparison groups, ensuring that the observed differences in treatment effects are likely attributable to the interventions themselves rather than discrepancies in participant demographics. By demonstrating that clinical characteristics such as setting and age are comparable across nodes, we have addressed the risk of intransitivity, thereby strengthening the validity of our indirect comparisons.

A number of pairwise comparisons for efficacy between two PS types have been performed in young or older people during exercise training [30,48,51,52,53,54,55], and there were no significant differences between any two of the protein sources, including whey, milk, soy or other mixed sources such as protein-rich meals. In two previous NMA [56,75], full comparisons among numerous protein sources were further investigated in individuals who were undergoing RET. Liao et al. indicated that whey protein augmented training efficacy the most in elderly with high sarcopenia risk [56]. In healthy individuals, Drummond et al. indicated that collagen and whey are the optimal supplements to enhance muscle mass and strength gains during RET interventions [75]. The above previous systematic reviews only focused on PS plus RET, despite other training modalities also being employed for older individuals, such as MET, which is composed of AET, balance training, function training or physical activity. In the current NMA, we made full comparisons across 10 types of PS, three exercise modalities (i.e., RET, AET, MET), and its combinations; the overall results showed that whey as well as collagen in combination with RET exhibited superior effects than other PS did on muscle mass and strength. In addition, our NMA further investigated treatment effects for physical indicators of sarcopenia; WP+RET as well as WP+MET was identified as the most optimal option to enhance performances in walking speed, chair rise, TUG, and SPPB.

In this paper, all types of PS combined with RET generally exhibited superior effects to other exercise, especially in muscle mass and strength. Such findings were in agreement with previous studies indicating more benefits derived from RET for skeletal lean mass and strength in older people [76,77,78]. Among the diverse exercise training modalities, RET was deemed to have the most promising effects on increments in muscle mass or volume since it significantly elucidates myofibril proliferation [79,80], muscle protein synthesis [81,82], and the ultimate hypertrophy [83,84]. Based on the current and previous results, RET is likely to play an important role among exercise regimens, and such findings are parallel with the recommendations that RET ought to be the first-line treatment to improve sarcopenia features [85], especially muscle mass and strength gains [86,87]. Additionally, it was noted that WP+MET yielded optimal effects on most physical indicators (i.e., CR, TUG, SPPB) despite WP+RET being the most effective regimen for walking speed (Figure 8); these results were supported by the previous studies exploring the optimal exercise intervention subtype for older individuals [87,88]. Our findings imply that rather than strength training alone, incorporating RET with other exercise modes such as aerobic training, balance training, and task-oriented function training may further promote performances in physical mobility which are more dependent on cardiopulmonary fitness, balance control, and motor coordination.

Supplementary protein supply is generally believed to enhance training efficacy for individuals undergoing exercise interventions. The comparative efficiency among various protein sources has been widely investigated; however, conflicting results remain regarding which protein type is more efficient among the animal and plant-derived sources [48,89]. Accordingly, these inconsistent results bring challenges in clinical decision making to set up an optimal treatment strategy, especially for older individuals with high sarcopenia risk. Some researchers reported that animal-based protein (mostly the whey supplements) achieves superior treatment efficacy to plant sources such as soya [45,53,90], whereas other authors have drawn conflicting conclusions [48,51,91,92]. In our NMA, varieties of animal and plant proteins were pooled together and fully compared. The analyzed results indicated that whey mostly achieved superior treatment efficacy to either other animal proteins or plant sources. Based on the current results, whey can be effectively employed prior to other supplements with or without exercise training.

In our NMA, whey supplements were found to be the most effective supplement to augment RET-induced muscle mass gain followed by collagen, meat, milk, and soy (Figure 4); additionally, these findings were in line with the present meta-regression analyses results indicating that animal protein is associated with greater effects on lean mass gain (Table S11). Accordingly, our NMA results revealed that animal-derived protein is likely to be more beneficial for lean mass than plant-based protein. These findings were supported by previous studies [51,53,89,93]; some possible reasons for this difference are further addressed below. First, animal protein, especially whey [94], is generally thought to have a higher leucine content compared with plant-based protein for increasing muscle mass [48,95,96,97]. Accordingly, whey intake leads to higher postprandial muscle protein synthesis rates than soy in older individuals [47,48]. Additionally, numerous previous results indicate that whey is generally superior to collagen [98], casein [46,99], milk [100], and soy [101] in the activation of myofibrillar protein synthesis after RET. Secondly, the superior effects of WP on myogenesis are attributed to its high leucine content and rapid digestion kinetics (i.e., aminoacidemia) [102], which becomes more evident while combining with RET [48,103]. Notably, such whey-induced acute increases in muscle protein synthesis rate may subsequently enhances skeletal muscle adaptations (i.e., muscle hypertrophy) in response to prolonged training [104]. Accounting for these previous results and our findings together, it appears that supplementation by animal protein may hold more promise in the prevention of sarcopenia than plant protein, especially whey intake. Finally, due to the heterogeneity across studies in this NMA, the present findings should be cautiously interpreted. The current NMA results showed that the superior efficacy of whey in lean mass was cosigned with strength gain and physical recovery. These findings are consistent with earlier meta-analyses showing that PS accelerate increases in muscle mass, strength gains, and the recovery of physical mobility in older adults participating in exercise training [105,106,107,108]. Considering that lean mass gains from PS play a key role in recovering strength and walking ability during exercise training [88], and that greater leg strength is linked to faster walking speeds after PS plus RET [109] in older adults with sarcopenia and frailty risks, the greater impact of whey supplements on muscle mass could work synergistically to enhance their benefits for strength and mobility compared to other protein sources in this NMA. With respect to the nutritional supplementation during rehabilitation, optimizing certain nutrients like L-carnitine may enhance functional recovery and physical performance in older adults [110]. In such a manner, incorporating PS into exercise training could offer a more comprehensive nutritional approach for sarcopenia management.

This NMA identified several substantial factors that significantly influenced the relative effectiveness among treatment regimens. First, greater gains in muscle mass were more likely observed in those who were male and aged younger than 65, as well as those with acute conditions (such as hospitalization) or chronic comorbidities (such as obesity, sarcopenia, frailty, or limited mobility), compared to the relatively healthy individuals who were female and aged older than ≥85 (Table S11). These findings are consistent with earlier reviews showing that people with sarcopenia or at risk of frailty often experience greater lean mass increases from PS plus exercise training than the healthier peers [111,112]. Some reasons can be addressed to explain our findings as follows: (1) the elder adults who suffered frailty, sarcopenia, or other chronic comorbidities have experienced a minor habitual intake of protein and lower physical activity level [113,114,115,116], which impacts myofibrillar protein synthetic rates after PS and exercise training [38,39,116]. Interestingly, those who suffered from sarcopenia and were older than 80 experienced higher post-prandial myofibrillar synthesis rates after whey ingestion compared with the healthy and younger (<70 years old) control [117]; (2) sex-specific differences in the effectiveness of PS combined with exercise have been observed among older adults, especially in relation to muscle strength [118,119,120] and chair stand performance [118]. Synthesizing these previous results together, older male adults with age-related conditions are more likely to have lean mass gains from PS during exercise training compared to their relatively younger, healthier female counterparts. Secondly, the protein source has a significant effect on lean mass gain following PS plus exercise training. Animal protein appears to achieve greater changes in muscle mass than plant protein, which may be due to the different leucine content between the two protein sources, since myogenesis is associated with leucine quality [43]. Thirdly, we observed that a greater amount of PS corresponds with greater changes in lean mass and muscle strength. Several previous studies have shown that post-exercise increases in myofibrillar fractional synthesis rates in response to PS stimuli are greatly elevated by higher protein intake in both trained young and older adults [121,122,123]. Accordingly, these short-term, dose-dependent effects on muscle growth can eventually result in lasting neuromuscular adaptations after consistent exercise training. Finally, the current NMR results revealed a negative relationship between BMI and handgrip strength gain, suggesting that a higher BMI was associated with smaller improvements in strength. Conversely, greater changes in handgrip strength may be observed over a longer intervention or follow-up length up to 12 months. These findings suggest that physical recovery is more noticeable at a 6-month follow-up or beyond compared to less than 3 months, particularly in normal-weight older adults. This suggests that regaining physical mobility through PS and exercise depends on time, particularly for individuals with lower BMIs.

The practical implications of PS plus exercise, including adherence and feasibility, are needed to be considered in older populations. Participant compliance with PS was similarly consistent across protocols with different protein sources and exercise types in the current NMA. The findings showed that older individuals tolerated all protein products from various sources well despite higher withdrawal rates for WP and its combination with RET. Additionally, a relatively higher occurrence of minor adverse events was observed with WP (OR = 2.18) and WP plus RET (OR = 1.73). After consuming whey supplements, most reported adverse events involved gastrointestinal issues such as nausea, diarrhea, bloating, or gastroesophageal reflux. Based on the results (Table S12), we believe that most adverse events linked to WP are not serious and can be effectively managed despite their higher frequency.

Some limitations in the following are needed to be counted while interpreting the findings of this paper. First, this NMA is limited by the varying methodological quality of the studies with only 39 out of 235 RCTs (16.6%) rated as having a low risk of bias. This weakens the strength of the results and could lead to overestimating the relative effects of different treatment regimens. Secondly, because of the wide variation in PS (protein source, dosage, intake time) and exercise protocols (training type, duration, and volume) across the included RCTs, we were not able to draw a definite conclusion about the treatment effectiveness of any specific protocol within the regimens. Thirdly, notable publication biases in treatment effects were observed for muscle mass and leg strength, reducing the certainty of the evidence. As a result, the conclusions for these outcomes should be cautiously interpreted. Furthermore, the transitivity assumption as well as heterogeneity of NMA is concerned based on the broad inclusion criteria. However, the assessment of transitivity showed that factors like setting and age were consistent across treatment arms; thus, the risk of intransitivity may be reduced, and the indirect comparisons were likely more reliable. Finally, some of the included RCTs used placebo supplementation either with exercise or without. The placebo effects were not excluded when assessing the treatment effects of training alone or RC groups. As a result, the relative effects of all treatment options might be underestimated when using the RC groups as a common reference.

## 5. Conclusions

This NMA determined the superiority of treatment efficacy among different regimens composed of varying sources of PS and exercise modality for older individuals. Overall, the synergistic use of WP with RET was likely the optimal approach for muscle mass and strength gains, with a moderate to high certainty of evidence, despite its relatively high risk of minor side effects. While combining with MET, the WP seems to have more favorable effects on recovering physical mobility.

The effectiveness of the treatment may depend on substantial factors including age, health status (whether acute or chronic conditions), sex, body weight, and protein source. It is also likely influenced by the PS dose and follow-up timing, especially when it comes to lean mass gains and regaining strength.

Given the substantial heterogeneity, the significant inconsistency reported in some comparisons, and the overall quality limitations of the evidence in this NMA, the findings in this paper are based on uncertain results and should be cautiously interpreted.

## Figures and Tables

**Table 1:** PICOS criteria for study selection.

PICOS item		Eligibility criteria
Participant (P)		Older adults aged 50 years or older. No limitation to living status (community dwelling, institutionalized or hospitalized resident), physical condition (active or independent, inactive or sedentary, sarcopenia, dynapenia, prefrail/frail), health status (untrained healthy, overweight/obese, acute onsets such as recent fracture and CVA, chronic comorbidities such as DM, COPD, CVD, osteopenia, osteoarthritis, etc.).
Intervention (I)		Protein nutrient intervention in combination with an exercise therapy
A. Protein nutrient intervention		
	The specified protein supplements were included and defined as follows (right side column).	(a) Animal-based sources like milk (whey, casein), meat (beef, chicken, fish etc.), collagen, and insect.
		(b) Plant-based sources like soy (pea, peanut, etc.), rice, and oat.
		(c) Protein-enriched meal composed of rich blended animal and plant protein in daily diets. Numerically, daily protein consumption exceeded 0.8 g per kilogram of body weight.
B. Exercise therapy		
	The specified exercise therapy was included and defined as follows (right side column).	(a) Resistance-based exercise training (RET): any exercise involving muscles resisting or opposing an external force. Resistance force can come from free weights (e.g., dumbbells, sandbags or barbells), elastic bands or tubing, body weight, or any other object that challenges the muscles, such as WBV, NMES, and WB-EMS.
		(b) Aerobic exercise training (AET): an exercise modality referring to fitness and cardiovascular reconditioning like walking, running, swimming, and bicycling.
		(c) Multi-composited exercise regimen (MET): an exercise program that combines two or more types of training, such as resistance, aerobic, balance, or functional training.
Comparison (C)		
	The comparator includes the following (right side column).	(a) Different protein sources of supplementation (b) Different training modalities (c) Regular (or standard) care with a placebo supplementation (d) Regular (or standard) care without any protein supplementation or exercise therapy
Outcome (O)		
Sarcopenia indices		A. Muscle growth or hypertrophy measures (a) Muscle mass (index): fat-free mass, whole-body lean mass, appendicular lean mass (b) Muscle volume: cross-sectional area, muscle girth, muscle thickness
		B. Muscle strength (a) Handgrip strength (b) Leg strength or power measures: 1-RM leg press; knee extensor peak power, isokinetic peak torque (0/60/120/180 degree per second), or MVIC; leg muscle quality
		C. Sarcopenia physical indices (a) Walk speed: 3/4/6/10/400 m level walk time (usual or fast), six-minute walk distance, etc. (b) Chair-rise performance: five-time chair rise, 30 s sit-to-stand task, etc. (c) 3 m (8 feet) timed up-and-go task (d) SPPB
Study design (S)		Randomized controlled trial; randomized crossover trial

1-RM, one repetition maximum; COPD, chronic obstructive pulmonary disease; CVA, cerebrovascular accident; CVD, cardiovascular disease; DM, diabetes mellitus; MVIC, maximum voluntary isometric contraction; NMES, neuromuscular electrical stimulation; SPPB, Short Physical Performance Battery; WB-EMS, whole-body electromyostimulation; WBV, whole body vibration.

**Table 2:** Study summary.

Study Group	PS Plus Exercise					Exercise (with or Without Placebo Supplement)					PS Alone					Control (Placebo Supplement or Regular Care)			
	Trials (*n*)	Study Arm (*n*)	Patients (*n*)	Mean (Range)		Trials (*n*)	Study Arm (*n*)	Patients (*n*)	Mean (Range)		Trials (n)	Study Arm (*n*)	Patients (*n*)	Mean (Range)		Trials (n)	Study Arm (*n*)	Patients (*n*)	Mean (Range)
Age, year, total **^a^**	235	287	10,539	72.8 (51.5–87.6)		148	155	4462	71.3 (51.0–86.2)		55	59	1685	73.7 (50.0–85.7)		95	96	4294	75.8 (54.1–89.2)
<65	51	64	1792	58.5 (51.5–64.9)		36	38	1020	59.0 (51.0–64.8)		12	14	239	60.1 (50.0–64.9)		10	10	180	60.1 (54.1–63.8)
65~74.9	106	133	4008	70.5 (65.1–74.9)		66	69	1803	70.4 (65.0–74.6)		20	22	627	73.1 (65.6–74.7)		43	44	1447	71.2 (65.6–74.5)
75~84.9	69	80	4293	79.5 (75.0–84.7)		43	45	1519	79.7 (75.0–84.7)		22	22	795	80.2 (76.5–84.6)		37	37	2455	78.8 (75.0–83.9)
≥85	9	10	446	86.2 (85.0–87.6)		3	3	120	85.8 (85.5–86.2)		1	1	24	85.7		5	5	212	86.1 (85.1–89.2)
BMI, kg/m^2^	215	263	9839	25.8 (17.1–34.9)		142	150	4325	25.8 (16.5–35.2)		49	52	1559	24.4 (18.6–32.1)		85	86	3968	25.5 (18.5–34.3)
Sex, n, %																			
Sex-specific trial ^a^	74	90	2135			55	60	1127			19	21	410			25	25	703	
Men	37	46	996	100		27	29	460	100		6	6	134	100		13	13	451	100
Women	37	44	1139	100		28	31	667	100		13	15	276	100		12	12	252	100
Sex-mixed trial ^a^	161	197	8404			93	95	3335			36	38	1275			70	71	3591	
Men ^b^	149	183	3251	43.1 (4.4–88.2)		83	85	1248	41.1 (9.8–84.6)		32	33	509	39.8 (12.4–86.9)		63	64	1303	41.7 (7.1–90.0)
Women ^b^	149	183	4766	56.9 (11.8–95.7)		83	85	1793	58.9 (15.4–90.2)		32	33	722	60.2 (13.0–80.0)		63	64	2146	58.3 (10.0–92.9)
Population (area)																			
America	52	68	1406			35	38	769			9	11	180			11	11	242	
Asia	89	100	3580			53	53	1570			28	29	968			43	44	1630	
Europe	80	100	4436			52	56	1747			16	17	467			35	35	2172	
Oceania	14	19	1117			8	8	376			2	2	70			6	6	250	
Physical condition																			
Physically independent	52	65	1138			41	44	1168			9	12	265			12	12	236	
Frailty	54	66	3247			31	34	1010			15	16	449			32	33	1656	
Sarcopenia	76	87	3833			38	38	1236			20	20	724			34	34	1961	
Dynapenia	15	18	489			11	11	315			4	4	123			6	6	189	
Sedentary/inactive	38	51	1832			27	28	733			7	7	124			11	11	252	
Living status																			
Institutionalized	47	55	1985			27	29	885			10	10	222			24	24	861	
Noninstitutionalized	188	232	8554			121	126	3577			45	49	1463			71	72	3433	
Acute or chronic conditions																			
Medically stable ^c^	176	219	8216			108	113	3087			46	50	1481			76	77	3679	
Overweight/obesity	19	23	1053			16	17	611			2	2	54			5	5	147	
Type-2 DM	9	11	351			4	4	196			1	1	11			2	2	91	
Osteoarthritis	5	5	155			4	4	111			0					1	1	43	
Osteopenia	5	5	138			2	2	94			3	3	87			3	3	94	
Fracture	7	9	348			5	6	174			1	1	25			3	3	153	
CVD	3	3	45			2	2	36			0					1	1	5	
COPD	4	5	73			2	2	32			0					2	2	42	
CVA	7	7	160			5	5	121			2	2	27			2	2	40	
Protein source (type)																			
Whey	111	116	3656								23	23	535						
Milk protein	56	59	2195								10	10	346						
Soy	28	28	637								10	10	156						
Collagen	6	6	99								1	1	50						
Meat	9	9	270								3	3	54						
Protein-enriched meal	50	53	3188								11	11	505						
Casein	10	10	252								1	1	39						
Insect protein	1	1	16								0								
Rice	4	4	214								0								
Oat	1	1	12								0								
Exercise type																			
Resistance training	130	164	4972			88	92	2306											
Aerobic training	21	26	787			12	12	398											
Multicomponent	84	97	4780			48	51	1758											
Intervention compliance (%) ^b^																			
PS	108	135	5550	86.5 (43.4–100)							29	31	771	82.8 (48–100)					
Exercise	112	138	5498	83.3 (41.6–100)		76	78	2411	82.5 (44.0–100)										
Placebo supplement						45	50	1559	88.1 (32.0–100)							9	9	263	80.1 (32.0–100)
Muscle mass (baseline) ^b^																			
TLM, kg	84	108	3898	42.5 (16.8–63.2)		59	59	1798	42.9 (16.2–64.0)		13	15	389	40.1 (21.7–56.6)		23	23	599	40.0 (17.8–57.5)
ALM, kg	62	77	3927	18.7 (7.9–30.8)		44	46	1549	20.1 (8.0–32.5)		13	14	553	16.8 (13.2–31.4)		22	22	1388	17.0 (12.3–30.9)
TLMI, kg/m^2^	95	119	4308	13.1 (4.5–21.1)		65	65	2074	13.5 (5.2–21.4)		20	22	523	12.8 (5.3–18.6)		30	30	851	12.7 (5.6–18.8)
ALMI, kg/m^2^	83	102	4028	6.67 (1.16–14.98)		52	54	1750	7.13 (1.08–14.31)		16	16	632	6.47 (5.07–12.97)		30	30	1001	6.35 (4.99–12.86)
Handgrip strength (baseline, kg) ^b^																			
Men	18	21	734	26.7 (15.3–46.3)		12	13	211	31.6 (14.6–42.2)		4	4	60	28.9 (19.1–36.5)		8	9	494	24.4 (15.3–41.8)
Women	19	22	1236	20.4 (11.6–28.3)		17	20	461	20.9 (12.2–27.9)		6	6	122	19.7 (15.9–23.0)		7	8	721	17.6 (16.3–23.9)
Pooled	120	154	6222	23.6 (8.9–57.3)		74	84	2436	24.8 (11.0–42.3)		29	31	937	20.7 (7.8–36.5)		51	56	2593	20.9 (10.4–41.8)
Physical mobility (baseline) ^b^																			
SPPB	39	46	2586	7.7 (2.7–11.8)		20	20	668	8.7 (4.3–11.7)		5	5	109	8.3 (6.2–11.4)		14	14	1312	7.2 (4.8–11.7)
5-time CR, s	56	71	2696	14.2 (4.6–33.8)		41	42	1384	13.9 (4.7–28.6)		9	10	280	13.7 (7.5–25.0)		21	21	686	15.2 (7.5–39.5)
GS, m/s	100	121	5496	0.98 (0.24–2.29)		64	66	2165	1.08 (0.29–2.01)		31	33	1148	1.01 (0.38–2.14)		45	46	2362	0.92 (0.36–2.05)
TUG, s	41	52	2217	9.8 (4.2–28.2)		22	24	908	9.8 (4.8–24.0)		8	8	216	12.8 (9.9–16.1)		16	16	605	10.2 (4.3–25.3)

^a^ Total sum of the indicated item. ^b^ All total sum values were calculated based on the available values reported in the analyzed studies. ^c^ Participants were identified as relatively healthy or medically stable if they were free from major chronic medical conditions; respiratory or pulmonary diseases; neuromuscular or orthopedic disorders; metabolic or cardiovascular diseases; and mental and physical impairments. ALM, appendicular lean mass; ALMI, appendicular lean mass index; BMI, body mass index; COPD, chronic obstructive pulmonary disease; CVA, cerebrovascular accident; CVD, cardiovascular disease; CR, chair rise; GS, gait speed; DM, diabetes mellitus; SPPB, Short Physical Performance Battery; TLM, total lean mass; TLMI, total lean mass index; TUG, timed up and go.

**Table 3:** Identified treatment options among the included studies.

Type of intervention Main regimen and its abbreviation								
A. Monotherapy								
	Primary exercise training	Abbreviation		Protein source for supplementation	Abbreviation		Reference	Abbreviation
	Resistance exercise training	RET		Whey protein	WP		Regular care	RC
				Milk protein	MP			
	Aerobic exercise training	AET		Soy protein	SP			
				Dietary protein	DP			
	Multicomponent exercise training	MET		Insect protein	Insect-PS			
				Casein				
				Collagen				
				Meat				
				Oat				
B. Combined treatment								
	Resistance exercise training			Aerobic exercise training			Multicomponent exercise training	
	Combined protein source	Abbreviation		Combined protein source	Abbreviation		Combined protein source	Abbreviation
	Whey protein	WP+RET		Whey protein	WP+AET		Whey protein	WP+MET
	Milk protein	MP+RET		Milk protein	MP+AET		Milk protein	MP+MET
	Soy protein	SP+RET		Soy protein	SP+AET		Soy protein	SP+MET
	Collagen	Collagen+RET		Collagen	Collagen+AET		Collagen	Collagen+MET
	Meat	Meat+RET		Dietary protein	DP+AET		Meat	Meat+MET
	Dietary protein	DP+RET		Casein	Casein+AET		Dietary protein	DP+MET
	Casein	Casein+RET		Insect protein	Insect-PS+AET		Casein	Casein+MET
	Rice	Rice+RET					Rice	Rice+MET
							Oat	Oat+MET

**Table 4:** Distribution of relevant variables and intervention characteristics across treatment arms.

Variable	Treatment Arms			
	PS Plus Exercise	Exercise	PS Alone	Control
No. of Trials	235	148	55	95
Age, mean (range), total	72.8 (51.5–87.6)	71.3 (51.0–86.2)	73.7 (50.0–85.7)	75.8 (54.1–89.2)
<65, *n* (%)	51 (21.7)	36 (24.3)	12 (21.8)	10 (10.5)
65~74.9, *n* (%)	106 (45.1)	66 (44.6)	20 (36.4)	43 (45.3)
75~84.9, *n* (%)	69 (29.4)	43 (29.1)	22 (40.0)	37 (38.9)
≥85, *n* (%)	9 (3.8)	3 (2.0)	1 (1.8)	5 (5.3)
Community-dwelling, *n* (%)	188 (80.0)	121 (81.8)	45 (81.8)	71 (74.7)
Physical condition, *n* (%)				
Physically independent	52 (22.1)	41 (27.7)	9 (16.4)	12 (12.6)
Frailty	54 (23.0)	31 (20.9)	15 (27.3)	32 (33.7)
Sarcopenia	76 (32.3)	38 (25.7)	20 (36.4)	34 (35.8)
Dynapenia	15 (6.4)	11 (7.4)	4 (7.3)	6 (6.3)
Sedentary or inactive	38 (16.2)	27 (18.2)	7 (12.7)	11 (11.6)
Healthy status, n%				
Medically stable ^c^	176 (74.9)	108 (73.0)	46 (83.6)	76 (80.0)
Acute or chronic conditions				
Overweight or obesity	19 (8.1)	16 (10.8)	2 (3.6)	5 (5.3)
Type-2 DM	9 (3.8)	4 (2.7)	1 (1.8)	2 (2.1)
Osteoarthritis	5 (2.1)	4 (2.7)	0	1 (1.1)
Osteopenia	5 (2.1)	2 (1.4)	3 (5.5)	3 (3.2)
Fracture	7 (3.0)	5 (3.4)	1 (1.8)	3 (3.2)
CVD	3 (1.3)	2 (1.4)	0	1 (1.1)
COPD	4 (1.7)	2 (1.4)	0	2 (2.1)
CVA	7 (3.0)	5 (3.4)	2 (3.6)	2 (2.1)
Protein source (type), n%				
Animal-based protein	193 (82.1)		38 (64.4)	
Whey	111 (47.2)		23 (41.8)	
Milk protein	56 (23.8)		10 (18.2)	
Collagen	6 (2.6)		1 (1.8)	
Meat	9 (3.8)		3 (5.5)	
Casein	10 (4.3)		1 (1.8)	
Insect-PS	1 (0.4)		0	
Plant-based protein	33 (14)		10 (18.2)	
Soy	28 (11.9)		10 (18.2)	
Rice	4 (1.7)		0	
Oat	1 (0.4)		0	
Mixed (protein-enriched meal)	50 (21.3)		11 (20.0)	
Mean Protein Dose (range)				
Animal-based protein, g/day	27.4 (4.0–64.5)		24.5 (4.0–60.0)	
≤20.0 g/day, n%	50 (21.3)		10 (18.2)	
>20.0 g/day, n%	143 (60.9)		28 (50.9)	
Plant-based protein, g/day	23.9 (3.2–50.0)		24.1 (6.8–40.0)	
≤20.0 g/day, %	15 (6.4)		4 (7.3)	
>20.0 g/day, %	18 (7.7)		6 (10.9)	
Mixed (protein-enriched meal), g/kg/day	1.17 (0.80–1.35)		1.17 (0.80–1.35)	
≤1.0 g/kg/day, %	7 (3.0)		3 (5.5)	
>1.0 g/kg/day, %	43 (18.3)		8 (14.5)	
Exercise type				
Resistance training, *n* (%)	151 (64.2)	88 (59.5)		
Duration (week), median (range)	12 (1–52)	14 (1–52)		
Intensity, *n* (%) ^a^				
Low	26 (17.2)	10 (11.4)		
Moderate	80 (52.9)	52 (59.1)		
High	45 (29.8)	26 (29.5)		
Aerobic training, n (%)	21 (8.9)	12 (8.1)		
Duration (week), median (range)	12 (1–36)	12 (2–24)		
Intensity, *n* (%) ^b^				
Low	3 (14.3)	1 (8.3)		
Moderate	15 (71.4)	8 (66.7)		
High	3 (14.3)	3 (25.0)		
Multicomponent, *n* (%)	88 (37.5)	51 (34.5)		
Duration (week), median (range)	12 (2–144)	12 (2–52)		
Intensity, *n* (%) ^c^				
Low	18 (20.5)	11 (21.6)		
Moderate	49 (55.7)	28 (54.9)		
High	21 (23.9)	12 (23.5)		

^a^ Training intensity is classified as Low (<50% one repetition maximum, 1-RM); Moderate (50–80% 1-RM); and High (>80% 1-RM). ^b^ Training intensity is classified as Low (<50% maximum heart rate, (Hmax) or maximum oxygen consumption ($\dot{V}$O_2_ max)); Moderate (50–80% Hmax or $\dot{V}$O_2_ max); and High (>80% Hmax or $\dot{V}$O_2_ max). ^c^ Exercise intensity is classified based on the rank of resistance and aerobic training load.

**Table 5:** GRADE certainty rating of treatment options in all main outcomes.

Treatment (Common Comparator: RC)	GRADE Certainty of Evidence ^a^								
	Muscle Mass		Muscle Strength			Physical Mobility			
			Handgrip	Leg Strength		GS	CR	TUG	SPPB
A. Combined therapy									
(a) Protein supplementation plus RET									
Casein+RET	⨁⨁⨁⊝ ^e^		⨁⨁⨁⊝ ^d^	⨁⨁⨁⊝ ^e^		⨁⨁⨁⨁	⨁⨁⨁⊝ ^d^		⨁⨁⨁⊝ ^d^
Collagen+RET	⨁⨁⨁⊝^e^		⨁⨁⨁⊝ ^d^	⨁⨁⨁⊝ ^e^		⨁⨁⨁⊝ ^d^	⨁⨁⨁⊝ ^d^		⨁⨁⨁⊝ ^d^
DP+RET	⨁⨁⊝⊝ ^b,e^		⨁⨁⨁⊝ ^c^	⨁⨁⨁⊝ ^e^		⨁⨁⨁⨁	⨁⨁⨁⨁	⨁⨁⨁⨁	⨁⨁⨁⊝ ^b^
Meat+RET	⨁⨁⨁⊝ ^e^		⨁⨁⨁⊝ ^d^	⨁⨁⨁⊝ ^e^		⨁⨁⨁⊝ ^d^	⨁⨁⨁⊝ ^d^	⨁⨁⨁⊝ ^d^	
MP+RET	⨁⨁⨁⊝ ^e^		⨁⨁⨁⨁	⨁⨁⨁⊝ ^e^		⨁⨁⨁⨁	⨁⨁⨁⨁	⨁⨁⨁⊝ ^d^	⨁⨁⊝⊝ ^b,d^
Rice+RET	⨁⨁⊝⊝ ^d,e^			⨁⨁⊝⊝ ^d,e^		⨁⨁⨁⊝ ^d^	⨁⨁⨁⊝ ^d^	⨁⨁⨁⊝ ^d^	
SP+RET	⨁⨁⨁⊝ ^e^		⨁⨁⨁⨁	⨁⨁⊝⊝ ^c,e^		⨁⨁⨁⨁	⨁⨁⨁⊝ ^d^	⨁⨁⨁⊝ ^d^	
WP+RET	⨁⨁⨁⊝ ^e^		⨁⨁⨁⨁	⨁⨁⨁⊝ ^e^		⨁⨁⨁⨁	⨁⨁⨁⨁	⨁⨁⨁⊝ ^d^	⨁⨁⨁⊝ ^b^
(b) Protein supplementation plus AET									
Casein+AET						⨁⨁⨁⊝ ^d^			
Collagen+AET	⨁⨁⊝⊝ ^d,e^		⨁⨁⊝⊝ ^c,d^						
DP+AET	⨁⨁⊝⊝ ^d,e^		⨁⨁⨁⊝ ^d^	⨁⨁⊝⊝ ^d,e^		⨁⨁⊝⊝ ^b,d^			⨁⨁⨁⊝ ^d^
Insect-PS+AET	⨁⨁⊝⊝ ^d,e^		⨁⨁⨁⊝ ^d^	⨁⨁⊝⊝ ^d,e^					
MP+AET	⨁⊝⊝⊝ ^c,d,e^		⨁⨁⨁⊝ ^d^	⨁⨁⊝⊝ ^d,e^		⨁⨁⨁⊝ ^d^	⨁⨁⨁⊝ ^d^	⨁⨁⨁⊝ ^d^	⨁⨁⨁⊝ ^d^
SP+AET	⨁⨁⊝⊝ ^d,e^		⨁⨁⨁⊝ ^d^	⨁⨁⊝⊝ ^d,e^		⨁⨁⨁⊝ ^d^			
WP+AET	⨁⊝⊝⊝ ^c,d,e^		⨁⨁⊝⊝ ^c,d^	⨁⨁⨁⊝ ^e^			⨁⨁⨁⊝ ^d^		⨁⨁⨁⊝ ^d^
(c) Protein supplementation plus MET									
Casein+MET	⨁⨁⊝⊝ ^d,e^		⨁⨁⨁⊝ ^d^	⨁⨁⨁⊝ ^e^		⨁⨁⨁⨁	⨁⨁⨁⊝ ^d^	⨁⨁⨁⊝ ^d^	⨁⨁⊝⊝ ^b,d^
Collegen+MET	⨁⨁⊝⊝ ^d,e^		⨁⨁⨁⊝ ^d^			⨁⨁⊝⊝ ^b,d^			
DP+MET	⨁⨁⨁⊝ ^e^		⨁⨁⨁⊝ ^d^	⨁⨁⊝⊝ ^d,e^		⨁⨁⨁⊝ ^b^	⨁⨁⨁⊝ ^b^	⨁⨁⊝⊝ ^b,d^	⨁⨁⨁⊝ ^d^
Meat+MET	⨁⨁⊝⊝ ^d,e^		⨁⨁⨁⊝ ^d^	⨁⨁⊝⊝ ^d,e^		⨁⨁⨁⊝ ^d^	⨁⨁⊝⊝ ^b,d^	⨁⨁⊝⊝ ^b,d^	
MP+MET	⨁⨁⨁⊝ ^e^		⨁⨁⨁⊝ ^d^	⨁⨁⨁⊝ ^e^		⨁⨁⨁⨁	⨁⨁⨁⨁	⨁⨁⨁⊝ ^d^	⨁⨁⨁⨁
Oat+MET	⨁⨁⊝⊝ ^d,e^			⨁⨁⨁⊝ ^e^					
Rice+MET	⨁⨁⊝⊝ ^d,e^		⨁⨁⨁⊝ ^d^			⨁⨁⨁⊝ ^d^	⨁⨁⨁⊝ ^d^		⨁⨁⨁⊝ ^d^
SP+MET	⨁⨁⨁⊝ ^e^		⨁⨁⨁⊝ ^d^	⨁⨁⊝⊝ ^d,e^		⨁⨁⨁⊝ ^d^	⨁⨁⨁⊝ ^d^	⨁⨁⨁⊝ ^d^	⨁⨁⨁⊝ ^d^
WP+MET	⨁⨁⨁⊝ ^e^		⨁⨁⨁⨁	⨁⨁⨁⊝ ^e^		⨁⨁⨁⨁	⨁⨁⨁⨁	⨁⨁⨁⨁	⨁⨁⨁⨁
B. Monotherapy									
(a) Exercise training alone									
RET	⨁⨁⨁⊝ ^e^		⨁⨁⨁⨁	⨁⨁⨁⊝ ^e^		⨁⨁⨁⨁	⨁⨁⨁⨁	⨁⨁⨁⊝ ^d^	⨁⨁⨁⊝ ^d^
AET	⨁⨁⊝⊝ ^d,e^		⨁⨁⨁⊝ ^d^	⨁⨁⊝⊝ ^d,e^		⨁⨁⨁⊝ ^d^	⨁⨁⨁⊝ ^d^	⨁⨁⨁⊝ ^d^	
MET	⨁⨁⊝⊝ ^d,e^		⨁⨁⊝⊝ ^c,d^	⨁⨁⨁⊝ ^e^		⨁⨁⨁⨁	⨁⨁⨁⊝ ^d^	⨁⨁⊝⊝ ^c,d^	⨁⨁⨁⊝ ^d^
(b) Protein supplementation alone									
Casein			⨁⨁⨁⊝^d^	⨁⨁⊝⊝ ^d,e^		⨁⨁⨁⊝ ^d^	⨁⨁⨁⊝ ^d^		
Collagen	⨁⨁⊝⊝ ^d,e^			⨁⨁⊝⊝ ^d,e^		⨁⨁⨁⊝ ^d^			
DP	⨁⨁⊝⊝ ^d,e^		⨁⨁⨁⊝ ^d^	⨁⨁⊝⊝ ^d,e^		⨁⨁⨁⊝ ^d^	⨁⨁⊝⊝ ^b,d^	⨁⨁⊝⊝ ^c,d^	⨁⨁⨁⨁
Meat	⨁⊝⊝⊝ ^b,d,e^		⨁⨁⨁⊝ ^d^	⨁⨁⊝⊝ ^d,e^		⨁⨁⨁⊝ ^d^			
MP	⨁⨁⊝⊝ ^d,e^		⨁⨁⨁⊝ ^d^	⨁⨁⊝⊝ ^d,e^		⨁⨁⨁⨁	⨁⨁⨁⊝ ^d^	⨁⨁⨁⊝ ^d^	⨁⨁⨁⊝ ^d^
SP	⨁⨁⊝⊝ ^d,e^		⨁⨁⨁⊝ ^d^	⨁⨁⊝⊝ ^d,e^		⨁⨁⊝⊝ ^b,d^	⨁⨁⨁⊝ ^d^		
WP	⨁⨁⊝⊝ ^d,e^		⨁⨁⨁⊝ ^d^	⨁⨁⊝⊝ ^d,e^		⨁⨁⨁⨁	⨁⨁⨁⊝ ^d^	⨁⨁⨁⊝ ^d^	⨁⨁⨁⊝ ^d^

^a^ Certainty of evidence is graded as follows: High: ⨁⨁⨁⨁; Moderate: ⨁⨁⨁⊝; Low: ⨁⨁⊝⊝; Very low: ⨁⊝⊝⊝. ^b^ Network contribution of high risk of bias ≧50%. ^c^ There is significant difference between direct and indirect estimates. ^d^ 95% confidence interval is wide and imprecise. ^e^ Test for publication bias is statistically significant. AET, aerobic exercise training; GRADE,^,^ Grading of Recommendations^,^ Assessment^,^ Development and Evaluations; GS, gait speed; CR, chair rise; Insect-PS, insect protein supplement; MET, multicomponent exercise training; MP, milk protein; RET, resistance exercise training; SP, soy protein; SPPB, Short Physical Performance Battery; TUG, timed up and go; WP, whey protein; RC, regular care.

## Data Availability

Data are presented within the Supplementary Materials.

## Supplementary Materials

The following supporting information can be downloaded at https://www.mdpi.com/article/10.3390/nu18091409/s1, Table S1. Database search formulas; Table S2. Summary of included study characteristics [38,109,118,119,120,124,125,126,127,128,129,130,131,132,133,134,135,136,137,138,139,140,141,142,143,144,145,146,147,148,149,150,151,152,153,154,155,156,157,158,159,160,161,162,163,164,165,166,167,168,169,170,171,172,173,174,175,176,177,178,179,180,181,182,183,184,185,186,187,188,189,190,191,192,193,194,195,196,197,198,199,200,201,202,203,204,205,206,207,208,209,210,211,212,213,214,215,216,217,218,219,220,221,222,223,224,225,226,227,228,229,230,231,232,233,234,235,236,237,238,239,240,241,242,243,244,245,246,247,248,249,250,251,252,253,254,255,256,257,258,259,260,261,262,263,264,265,266,267,268,269,270,271,272,273,274,275,276,277,278,279,280,281,282,283,284,285,286,287,288,289,290,291,292,293,294,295,296,297,298,299,300,301,302,303,304,305,306,307,308,309,310,311,312,313,314,315,316,317,318,319,320,321,322,323,324,325,326,327,328,329,330,331,332,333,334,335,336,337,338,339,340,341,342,343,344,345,346,347,348,349,350,351,352,353,354,355,356,357,358,359,360,361,362,363,364,365,366,367,368,369,370,371,372,373,374,375,376,377,378,379,380]; Table S3. Outcome measures identified among the included trials; Table S4. Direct and network estimates for muscle mass; Table S5. Direct and network estimates for handgrip strength; Table S6. Direct and network estimates for leg strength; Table S7. Direct and network estimates for walking speed; Table S8. Direct and network estimates for chair stand; Table S9. Direct and network estimates for timed up-and-go performance; Table S10. Direct and network estimates for global physical mobility (SPPB); Table S11. Summary for meta-regression results; Table S12. Assessment for treatment safety; Table S13. GRADE certainty rating of treatment efficacy for muscle mass gain; Table S14. GRADE certainty rating of treatment efficacy for handgrip strength outcome; Table S15. GRADE certainty rating of treatment efficacy for leg strength outcome; Table S16. GRADE certainty rating of treatment efficacy for walking speed outcome; Table S17. GRADE certainty rating of treatment efficacy for chair rise outcome; Table S18. GRADE certainty rating of treatment efficacy for timed up-and-go outcome; Table S19. GRADE certainty rating of treatment efficacy for global mobility (SPPB) outcome; Figure S1. Judgements of risk-of-bias items within each trial; Figure S2. Inconsistency assessment results for muscle mass; Figure S3. Treatment effects for muscle mass gain within time frames; Figure S4. Inconsistency assessment results for handgrip strength; Figure S5. Inconsistency assessment results for leg strength; Figure S6. Treatment effects for handgrip strength within time frames; Figure S7. Treatment effects for leg strength within time frames; Figure S8. Treatment effects for walking speed within time frames; Figure S9. Treatment effects for chair stand within time frames; Figure S10. Treatment effects for timed up-and-go performance within time frames; Figure S11. Treatment effects for global mobility (SPPB) within time frames; Figure S12. Inconsistency assessment results for walking speed; Figure S13. Inconsistency assessment results for chair rise; Figure S14. Inconsistency assessment results for timed up-and-go performance; Figure S15. Inconsistency assessment results for global mobility (SPPB); Figure S16. Funnel plots for treatment outcomes.
