# Supplementary material for: Explore the Optimal Treatment Regimen Across Combinations of Variate Protein Sources and Exercise Modalities and Its Associated Factors in Older Adults: A Network Meta-Analysis and Meta-Regression of Randomized Controlled Trials
Source: Nutrients. 2026 Apr 29;18(9):1409. doi: 10.3390/nu18091409 (PMC13165454; doi:10.3390/nu18091409)

Supplementary figures

Explore the Optimal Treatment Regimen across Combinations of Variate Protein Sources and Exercise Modalities and Its Associated Factors in Older Adults: A Network Meta-Analysis and Meta-Regression of Randomized Controlled Trials

Che-Li Lin [1,2], Shih-Wei Huang [3,4], Hung-Chou Chen [4,5], Mao-Hua Huang [6], Tsan-Hon Liou [3,4] and Chun-De Liao [5,7*]

[1] Department of Orthopedic Surgery, Shuang Ho Hospital, Taipei Medical University, New Taipei City 23561, Taiwan; 11010@s.tmu.edu.tw (C.-L. L.)
[2] Department of Orthopedics, School of Medicine, College of Medicine, Taipei Medical University, Taipei 11031, Taiwan
[3] Department of Physical Medicine and Rehabilitation, Wan Fang Hospital, Taipei Medical University, Taipei 116079, Taiwan; 113128@w.tmu.edu.tw (S.-W. H.); peter_liou@s.tmu.edu.tw (T.-H. L.)
[4] Department of Physical Medicine and Rehabilitation, School of Medicine, College of Medicine, Taipei Medical University, Taipei 110301, Taiwan; 10462@s.tmu.edu.tw (H.-C.C.)
[5] Department of Physical Medicine and Rehabilitation, Shuang Ho Hospital, Taipei Medical University, New Taipei City 235041, Taiwan
[6] Department of Biochemistry, University of Washington, Seattle, Washington, 98015, USA; huangkevin507@gmail.com
[7] International Ph.D. Program in Gerontology and Long-Term Care, College of Nursing, Taipei Medical University, Taipei 110301, Taiwan

[*] **Correspondence** Chun-De Liao, PT, PhD Tel: 886-2-2249-0088 ext. 1600 Fax: 886-2-2248-0577 Email: 08415@s.tmu.edu.tw

Title of figure	Page
Figure S1. Judgements of risk-of-bias items within each trial.	1
Figure S2. Inconsistency assessment results for muscle mass.	2
Figure S3. Treatment effects for muscle mass gain within time frames.	3
Figure S4. Inconsistency assessment results for handgrip strength.	4
Figure S5. Inconsistency assessment results for leg strength.	5
Figure S6. Treatment effects for handgrip strength within time frames.	6
Figure S7. Treatment effects for leg strength within time frames.	7
Figure S8. Treatment effects for walking speed within time frames.	8
Figure S9. Treatment effects for chair stand within time frames.	9
Figure S10. Treatment effects for timed up-and-go performance within time frames.	10
Figure S11. Treatment effects for global mobility (SPPB) within time frames.	11
Figure S12. Inconsistency assessment results for walking speed.	12
Figure S13. Inconsistency assessment results for chair rise.	13
Figure S14. Inconsistency assessment results for timed up-and-go performance.	14
Figure S15. Inconsistency assessment results for global mobility (SPPB).	15
Figure S16. Funnel plots for treatment outcomes.	16

Supplementary figure S1. Judgements of risk-of-bias items within each trial.

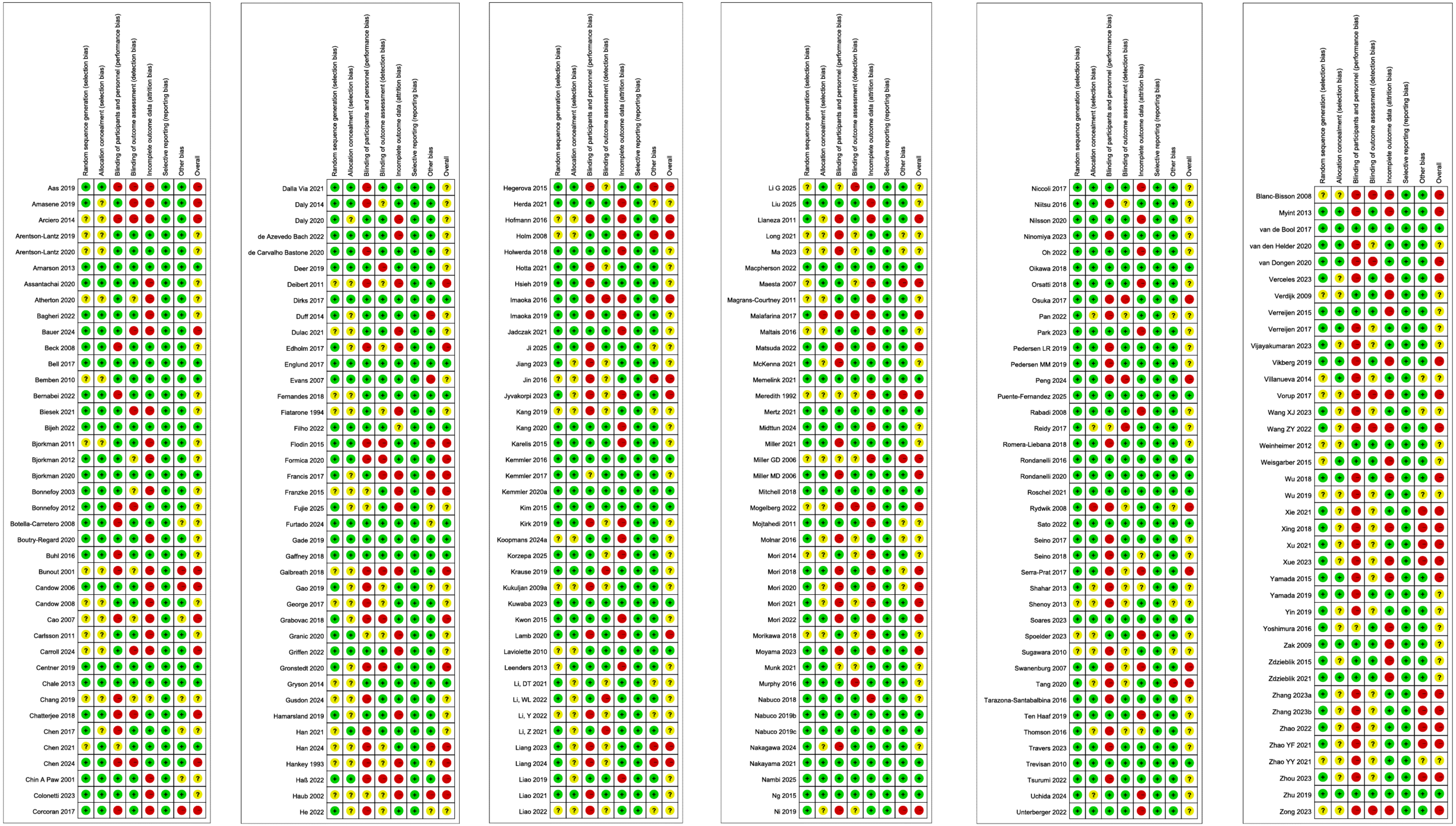

Supplementary figure S2. Inconsistency assessment results for muscle mass.

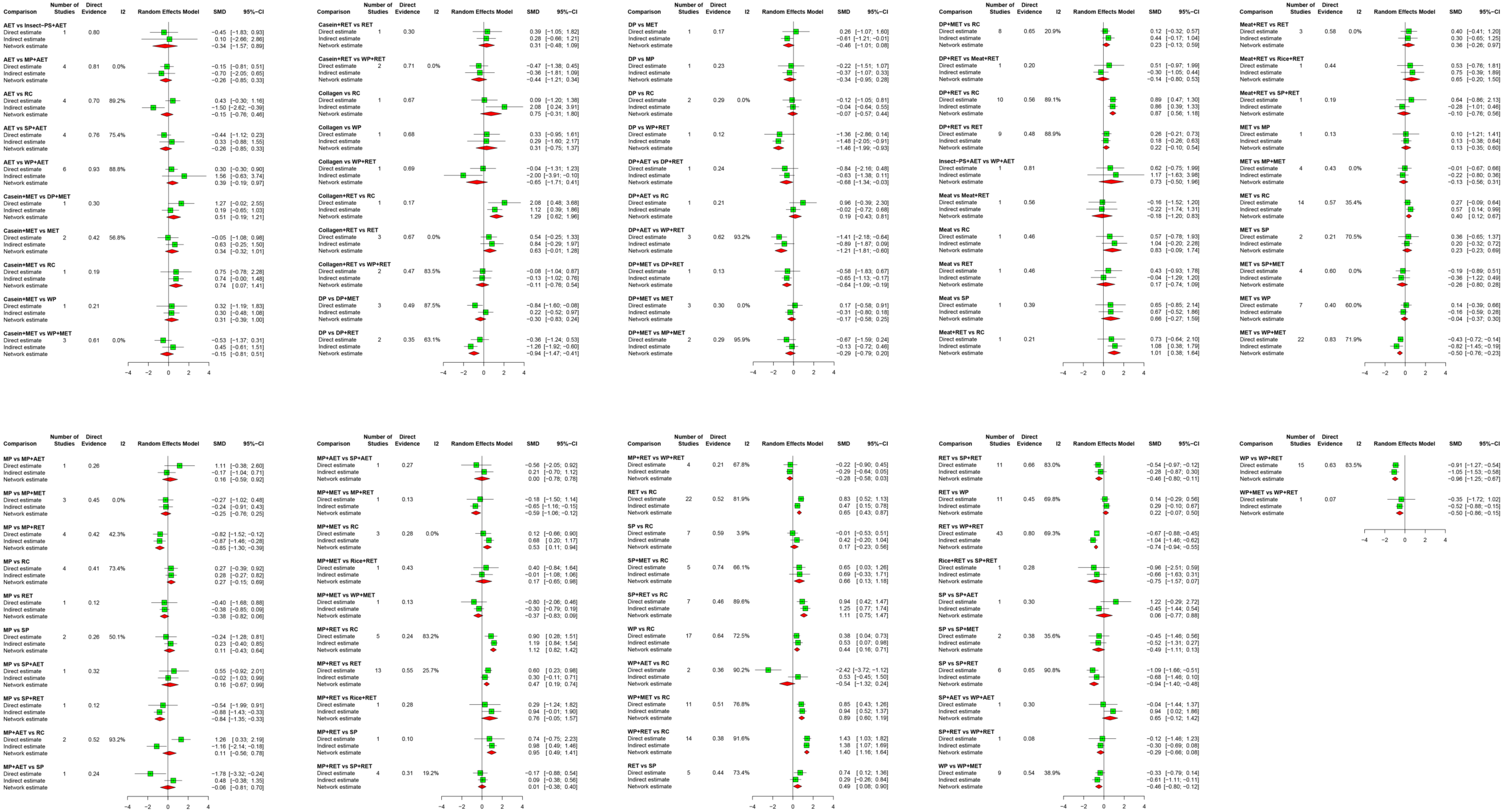

Supplementary figure S3. Treatment effects for muscle mass gain within time frames.

Follow up ≤3 months

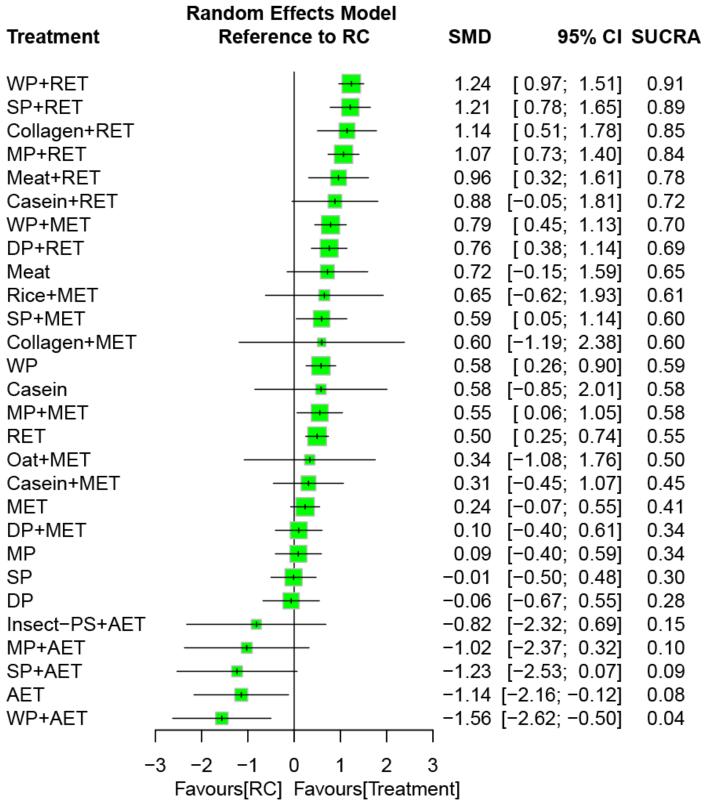

Follow up >3 months, ≤6 months

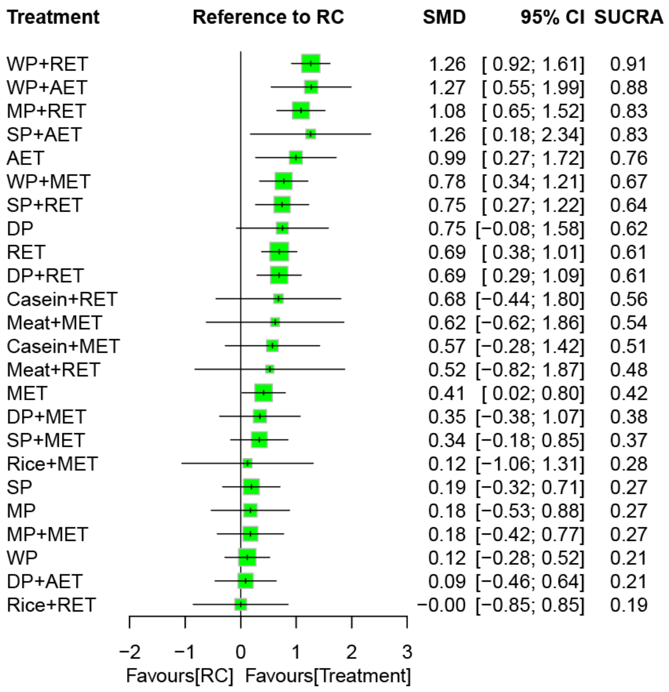

Follow up >6 months

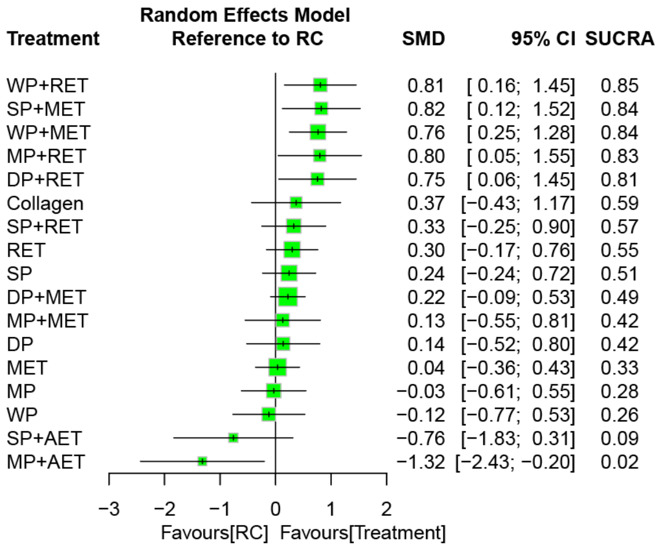

Supplementary figure S4. Inconsistency assessment results for handgrip strength.

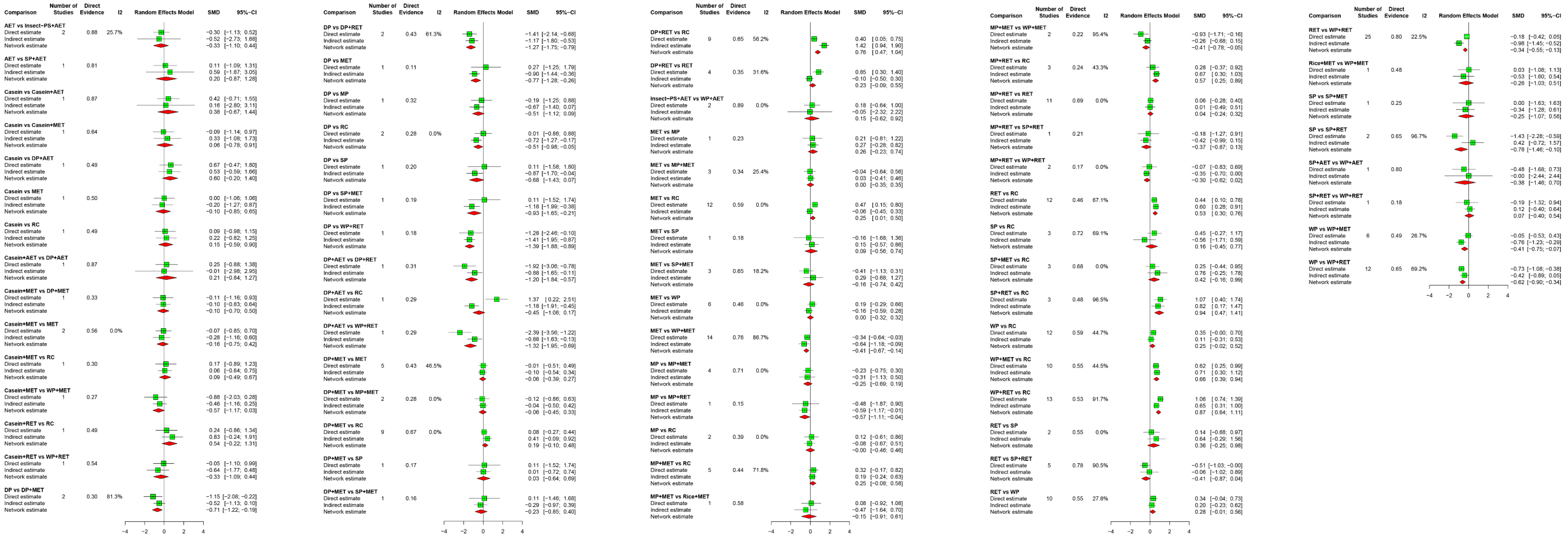

Supplementary figure S5. Inconsistency assessment results for leg strength.

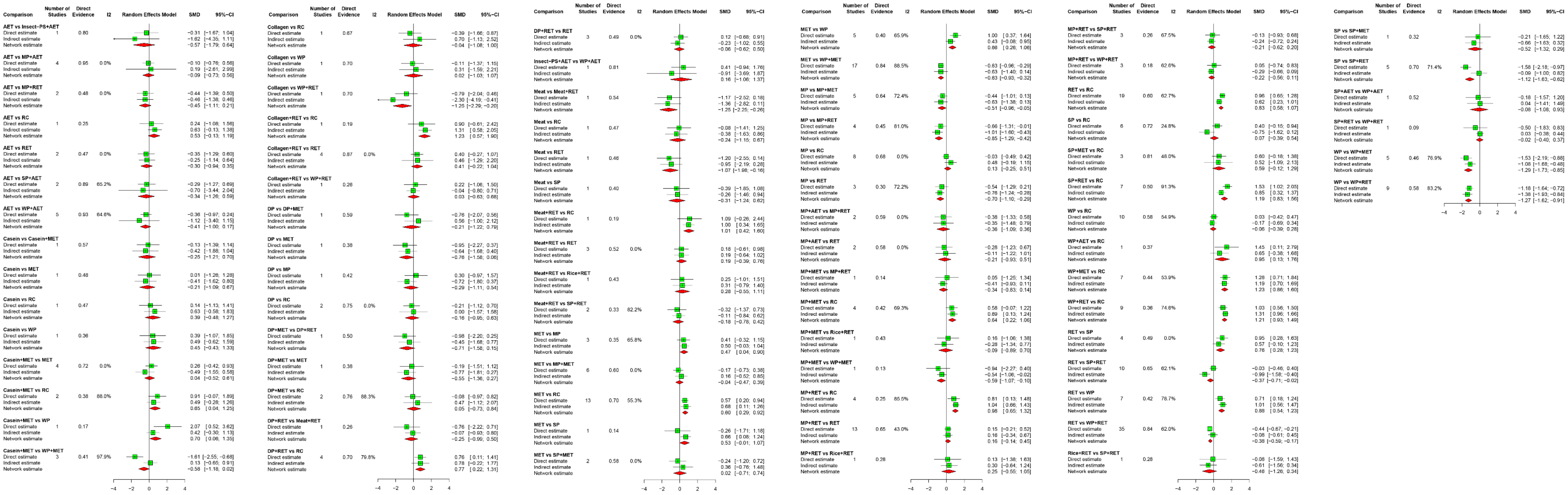

Supplementary figure S6. Treatment effects for handgrip strength within time frames.

Follow up ≤3 months

Random Effects Model
Reference to RC

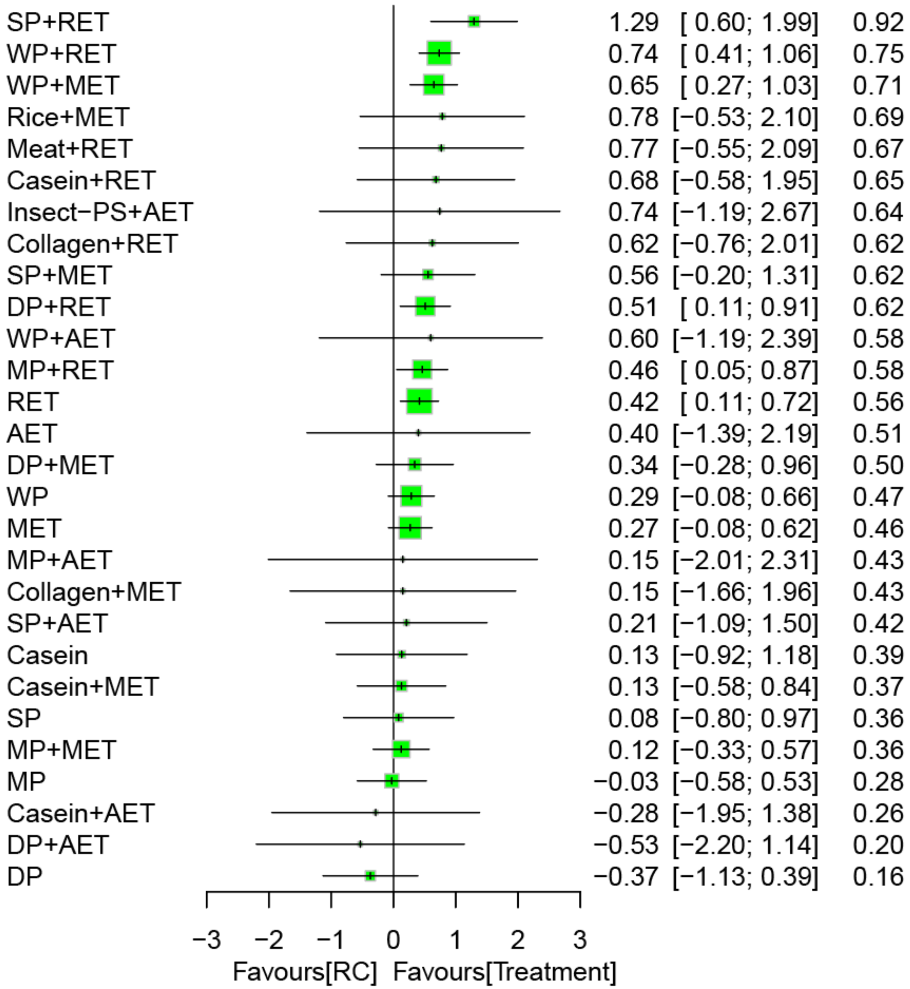

Treatment	SMD	95% CI	SUCRA
SP+RET	1.29	[0.60; 1.99]	0.92
WP+RET	0.74	[0.41; 1.06]	0.75
WP+MET	0.65	[0.27; 1.03]	0.71
Rice+MET	0.78	[−0.53; 2.10]	0.69
Meat+RET	0.77	[−0.55; 2.09]	0.67
Casein+RET	0.68	[−0.58; 1.95]	0.65
Insect−PS+AET	0.74	[−1.19; 2.67]	0.64
Collagen+RET	0.62	[−0.76; 2.01]	0.62
SP+MET	0.56	[−0.20; 1.31]	0.62
DP+RET	0.51	[0.11; 0.91]	0.62
WP+AET	0.60	[−1.19; 2.39]	0.58
MP+RET	0.46	[0.05; 0.87]	0.58
RET	0.42	[0.11; 0.72]	0.56
AET	0.40	[−1.39; 2.19]	0.51
DP+MET	0.34	[−0.28; 0.96]	0.50
WP	0.29	[−0.08; 0.66]	0.47
MET	0.27	[−0.08; 0.62]	0.46
MP+AET	0.15	[−2.01; 2.31]	0.43
Collagen+MET	0.15	[−1.66; 1.96]	0.43
SP+AET	0.21	[−1.09; 1.50]	0.42
Casein	0.13	[−0.92; 1.18]	0.39
Casein+MET	0.13	[−0.58; 0.84]	0.37
SP	0.08	[−0.80; 0.97]	0.36
MP+MET	0.12	[−0.33; 0.57]	0.36
MP	−0.03	[−0.58; 0.53]	0.28
Casein+AET	−0.28	[−1.95; 1.38]	0.26
DP+AET	−0.53	[−2.20; 1.14]	0.20
DP	−0.37	[−1.13; 0.39]	0.16

Follow up >3 months, ≤6 months

Random Effects Model
Reference to RC

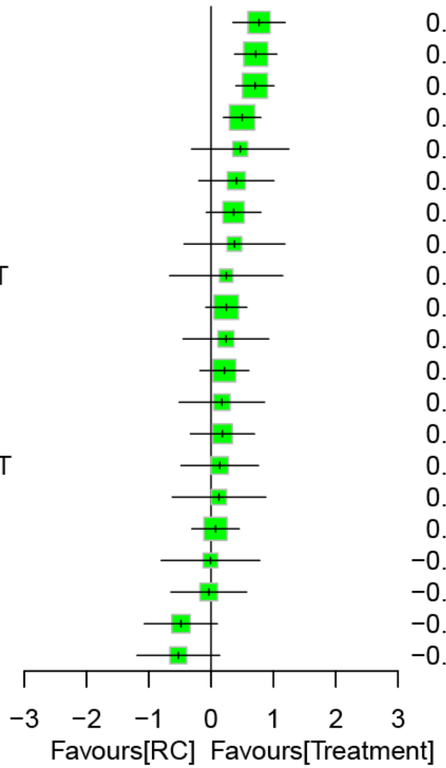

Treatment	SMD	95% CI	SUCRA
MP+RET	0.77	[0.35; 1.19]	0.89
DP+RET	0.72	[0.37; 1.06]	0.88
WP+RET	0.70	[0.40; 1.01]	0.88
RET	0.50	[0.20; 0.80]	0.73
AET	0.47	[−0.31; 1.25]	0.66
SP+RET	0.41	[−0.20; 1.01]	0.62
WP+MET	0.36	[−0.08; 0.80]	0.61
WP+AET	0.37	[−0.44; 1.19]	0.60
Casein+RET	0.24	[−0.67; 1.15]	0.51
MET	0.24	[−0.09; 0.57]	0.51
Rice+MET	0.24	[−0.45; 0.93]	0.50
MP+MET	0.21	[−0.18; 0.61]	0.48
MP	0.17	[−0.51; 0.86]	0.45
DP+MET	0.18	[−0.33; 0.70]	0.45
Casein+MET	0.14	[−0.48; 0.76]	0.42
Casein	0.13	[−0.63; 0.88]	0.41
WP	0.07	[−0.31; 0.45]	0.35
SP	−0.01	[−0.80; 0.78]	0.33
SP+MET	−0.04	[−0.65; 0.57]	0.30
DP+AET	−0.49	[−1.07; 0.10]	0.07
DP	−0.53	[−1.19; 0.14]	0.07

Follow up >6 months

Random Effects Model
Reference to RC

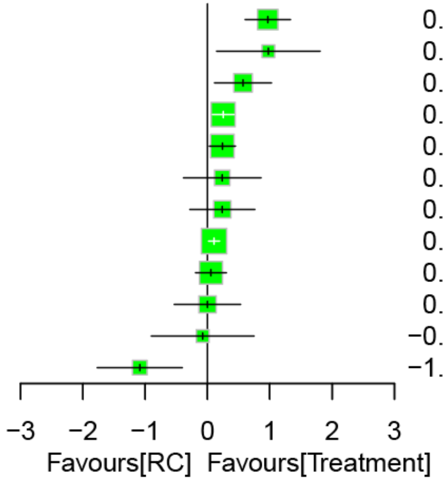

Treatment	SMD	95% CI	SUCRA
WP+MET	0.97	[0.60; 1.33]	0.95
WP+RET	0.98	[0.15; 1.81]	0.93
DP+RET	0.57	[0.11; 1.03]	0.80
MET	0.25	[0.08; 0.43]	0.57
MP+MET	0.24	[0.03; 0.45]	0.55
RET	0.24	[−0.39; 0.86]	0.55
SP+RET	0.24	[−0.29; 0.76]	0.54
DP+MET	0.11	[0.02; 0.20]	0.40
MP	0.06	[−0.19; 0.30]	0.34
SP	0.00	[−0.53; 0.53]	0.30
WP	−0.08	[−0.90; 0.75]	0.30
DP	−1.09	[−1.77; −0.40]	0.00

Supplementary figure S7. Treatment effects for leg strength within time frames.

Follow up ≤3 months

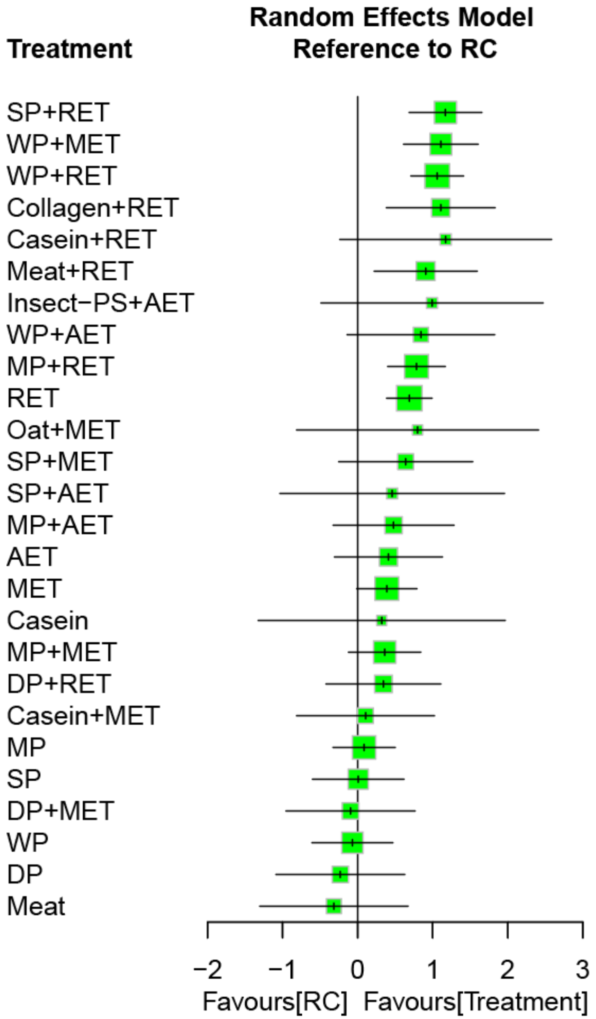

Treatment	SMD	95% CI	SUCRA
SP+RET	1.17	[0.68; 1.65]	0.85
WP+MET	1.11	[0.61; 1.60]	0.83
WP+RET	1.06	[0.71; 1.41]	0.81
Collagen+RET	1.11	[0.38; 1.83]	0.80
Casein+RET	1.17	[−0.24; 2.58]	0.77
Meat+RET	0.91	[0.22; 1.59]	0.72
Insect−PS+AET	0.99	[−0.49; 2.47]	0.72
WP+AET	0.84	[−0.14; 1.83]	0.68
MP+RET	0.78	[0.40; 1.17]	0.67
RET	0.69	[0.39; 0.99]	0.61
Oat+MET	0.80	[−0.81; 2.41]	0.60
SP+MET	0.64	[−0.25; 1.53]	0.58
SP+AET	0.46	[−1.04; 1.96]	0.50
MP+AET	0.48	[−0.33; 1.29]	0.50
AET	0.41	[−0.31; 1.13]	0.45
MET	0.39	[−0.01; 0.79]	0.44
Casein	0.32	[−1.33; 1.97]	0.43
MP+MET	0.36	[−0.12; 0.84]	0.42
DP+RET	0.34	[−0.42; 1.11]	0.41
Casein+MET	0.10	[−0.81; 1.02]	0.29
MP	0.09	[−0.33; 0.50]	0.26
SP	0.01	[−0.60; 0.62]	0.23
DP+MET	−0.10	[−0.96; 0.77]	0.22
WP	−0.07	[−0.61; 0.47]	0.18
DP	−0.23	[−1.09; 0.63]	0.16
Meat	−0.32	[−1.31; 0.67]	0.14

Follow up >3 months, ≤6 months

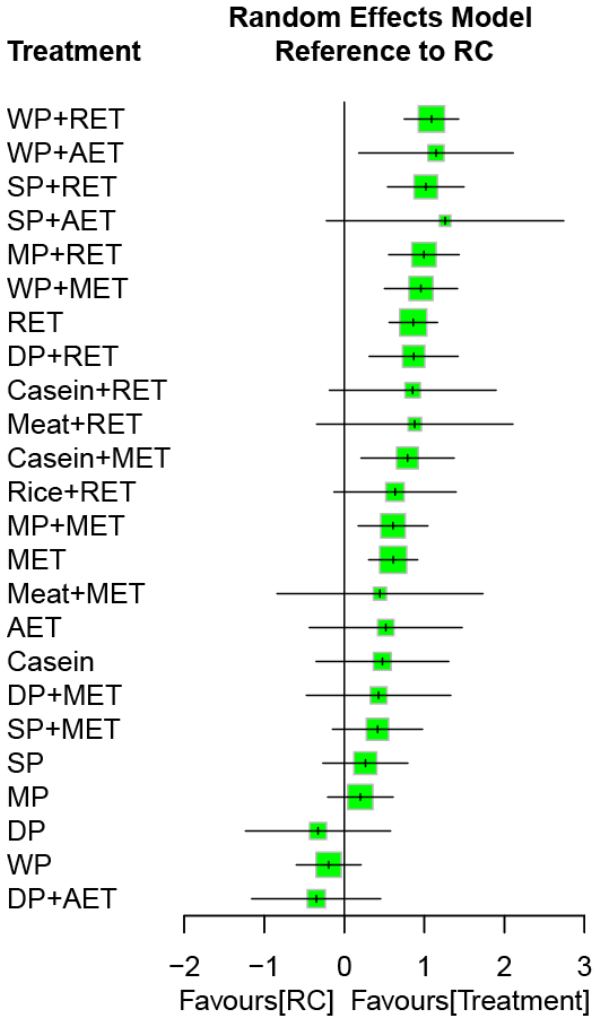

Treatment	SMD	95% CI	SUCRA
WP+RET	1.09	[0.75; 1.43]	0.83
WP+AET	1.15	[0.18; 2.11]	0.79
SP+RET	1.02	[0.54; 1.50]	0.78
SP+AET	1.26	[−0.22; 2.74]	0.77
MP+RET	1.00	[0.55; 1.44]	0.76
WP+MET	0.96	[0.50; 1.41]	0.74
RET	0.86	[0.56; 1.16]	0.67
DP+RET	0.87	[0.31; 1.42]	0.67
Casein+RET	0.85	[−0.19; 1.90]	0.65
Meat+RET	0.88	[−0.35; 2.11]	0.63
Casein+MET	0.79	[0.21; 1.37]	0.62
Rice+RET	0.63	[−0.13; 1.40]	0.52
MP+MET	0.61	[0.17; 1.04]	0.49
MET	0.61	[0.30; 0.92]	0.49
Meat+MET	0.45	[−0.84; 1.73]	0.45
AET	0.52	[−0.44; 1.47]	0.44
Casein	0.48	[−0.35; 1.30]	0.43
DP+MET	0.43	[−0.47; 1.33]	0.42
SP+MET	0.41	[−0.15; 0.98]	0.38
SP	0.26	[−0.27; 0.79]	0.29
MP	0.20	[−0.21; 0.61]	0.26
DP	−0.33	[−1.23; 0.58]	0.09
WP	−0.19	[−0.60; 0.21]	0.09
DP+AET	−0.35	[−1.16; 0.46]	0.08

Follow up >6 months

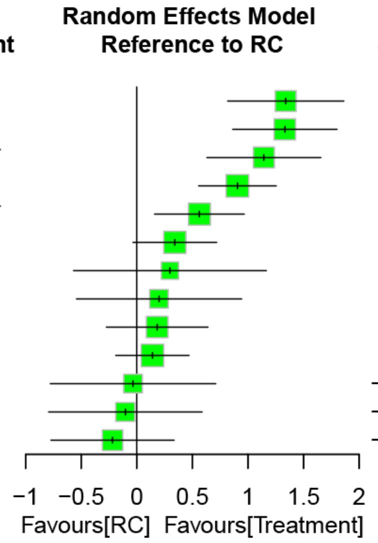

Treatment	SMD	95% CI	SUCRA
SP+RET	1.34	[0.82; 1.86]	0.93
MP+RET	1.33	[0.86; 1.80]	0.93
WP+RET	1.14	[0.63; 1.66]	0.87
RET	0.91	[0.56; 1.26]	0.78
MP+MET	0.56	[0.16; 0.97]	0.63
MET	0.34	[−0.03; 0.72]	0.50
SP+MET	0.30	[−0.57; 1.17]	0.45
DP+MET	0.20	[−0.54; 0.94]	0.39
SP	0.18	[−0.27; 0.64]	0.38
MP	0.14	[−0.19; 0.47]	0.36
DP	−0.03	[−0.78; 0.71]	0.23
Collagen	−0.10	[−0.79; 0.59]	0.20
WP	−0.22	[−0.77; 0.34]	0.12

Supplementary figure S8. Treatment effects for walking speed within time frames.

Overall follow-up duration

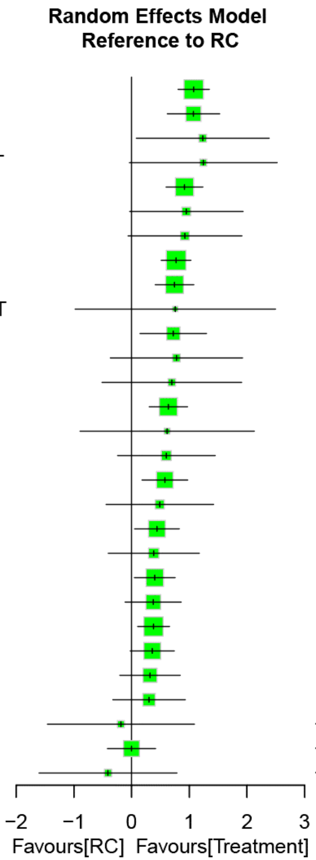

Treatment	SMD	95% CI	SUCRA
WP+RET	1.08	[0.81; 1.35]	0.85
SP+RET	1.07	[0.62; 1.53]	0.83
Casein+RET	1.23	[0.08; 2.38]	0.80
Collagen+RET	1.24	[−0.04; 2.52]	0.80
WP+MET	0.92	[0.60; 1.24]	0.75
AET	0.95	[−0.03; 1.93]	0.71
MP+AET	0.92	[−0.06; 1.91]	0.68
RET	0.77	[0.51; 1.03]	0.66
MP+RET	0.74	[0.41; 1.08]	0.63
Collagen+MET	0.76	[−0.98; 2.49]	0.61
Casein+MET	0.72	[0.15; 1.30]	0.60
Meat+MET	0.78	[−0.37; 1.92]	0.60
Meat+RET	0.70	[−0.51; 1.91]	0.58
WP	0.64	[0.31; 0.97]	0.55
SP+AET	0.62	[−0.89; 2.13]	0.53
Rice+MET	0.60	[−0.24; 1.45]	0.53
MP+MET	0.58	[0.18; 0.97]	0.50
Collagen	0.49	[−0.44; 1.42]	0.46
DP+RET	0.44	[0.05; 0.83]	0.40
Casein	0.38	[−0.40; 1.17]	0.37
DP+MET	0.40	[0.05; 0.75]	0.37
SP	0.37	[−0.11; 0.86]	0.35
MET	0.38	[0.11; 0.66]	0.35
MP	0.36	[−0.02; 0.74]	0.34
SP+MET	0.32	[−0.20; 0.84]	0.32
DP+AET	0.30	[−0.33; 0.93]	0.31
Rice+RET	−0.19	[−1.46; 1.09]	0.17
DP	−0.00	[−0.42; 0.42]	0.13
Casein+AET	−0.41	[−1.60; 0.78]	0.10

Follow up ≤3 months

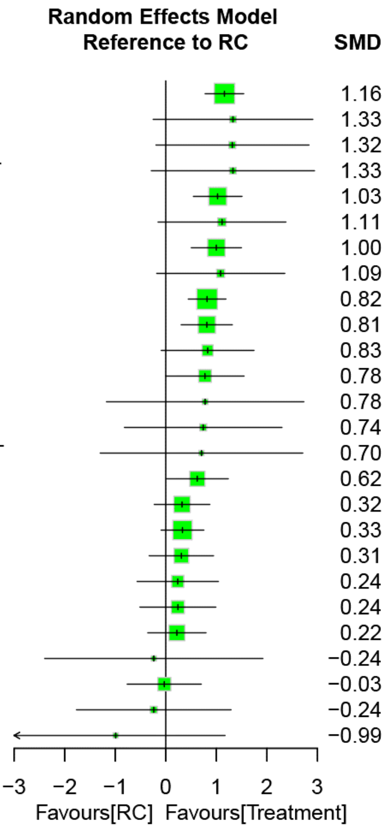

Treatment	SMD	95% CI	SUCRA
WP+RET	1.16	[0.78; 1.54]	0.80
Rice+MET	1.33	[−0.25; 2.91]	0.78
Casein+RET	1.32	[−0.20; 2.83]	0.76
Collagen+RET	1.33	[−0.29; 2.94]	0.76
MP+RET	1.03	[0.55; 1.50]	0.73
AET	1.11	[−0.15; 2.37]	0.72
WP+MET	1.00	[0.50; 1.50]	0.72
MP+AET	1.09	[−0.18; 2.35]	0.71
RET	0.82	[0.44; 1.19]	0.63
WP	0.81	[0.31; 1.32]	0.62
Casein+MET	0.83	[−0.09; 1.74]	0.61
SP+RET	0.78	[0.00; 1.55]	0.59
SP+AET	0.78	[−1.17; 2.73]	0.57
Meat+RET	0.74	[−0.82; 2.30]	0.55
Collagen+MET	0.70	[−1.30; 2.71]	0.54
MP+MET	0.62	[0.01; 1.24]	0.51
MP	0.32	[−0.23; 0.87]	0.35
MET	0.33	[−0.09; 0.75]	0.35
DP+RET	0.31	[−0.33; 0.95]	0.34
SP	0.24	[−0.57; 1.04]	0.32
SP+MET	0.24	[−0.51; 0.99]	0.32
DP+MET	0.22	[−0.36; 0.79]	0.31
Casein	−0.24	[−2.40; 1.92]	0.25
DP	−0.03	[−0.76; 0.70]	0.21
DP+AET	−0.24	[−1.76; 1.29]	0.18
Casein+AET	−0.99	[−3.15; 1.17]	0.10

Follow up >3 months, ≤6 months

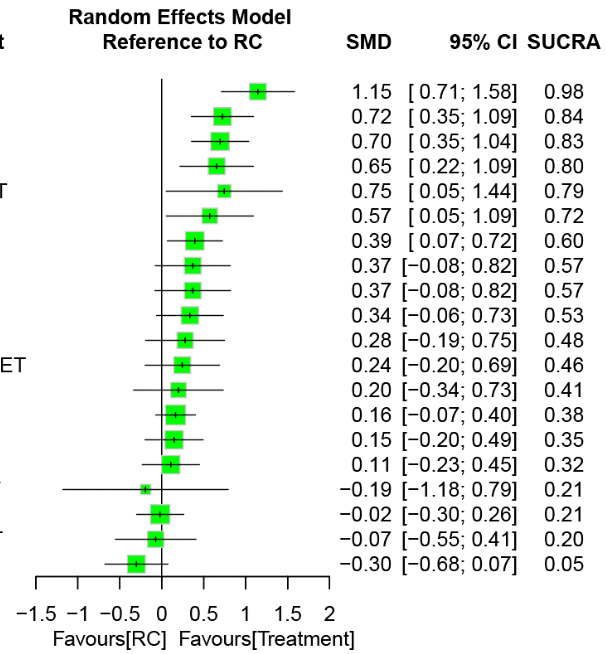

Treatment	SMD	95% CI	SUCRA
SP+RET	1.15	[0.71; 1.58]	0.98
WP+RET	0.72	[0.35; 1.09]	0.84
RET	0.70	[0.35; 1.04]	0.83
MP+RET	0.65	[0.22; 1.09]	0.80
Meat+MET	0.75	[0.05; 1.44]	0.79
DP+AET	0.57	[0.05; 1.09]	0.72
DP+RET	0.39	[0.07; 0.72]	0.60
DP+MET	0.37	[−0.08; 0.82]	0.57
SP	0.37	[−0.08; 0.82]	0.57
SP+MET	0.34	[−0.06; 0.73]	0.53
WP	0.28	[−0.19; 0.75]	0.48
Casein+MET	0.24	[−0.20; 0.69]	0.46
Casein	0.20	[−0.34; 0.73]	0.41
MET	0.16	[−0.07; 0.40]	0.38
MP	0.15	[−0.20; 0.49]	0.35
WP+MET	0.11	[−0.23; 0.45]	0.32
Rice+RET	−0.19	[−1.18; 0.79]	0.21
MP+MET	−0.02	[−0.30; 0.26]	0.21
Rice+MET	−0.07	[−0.55; 0.41]	0.20
DP	−0.30	[−0.68; 0.07]	0.05

Follow up >6 months

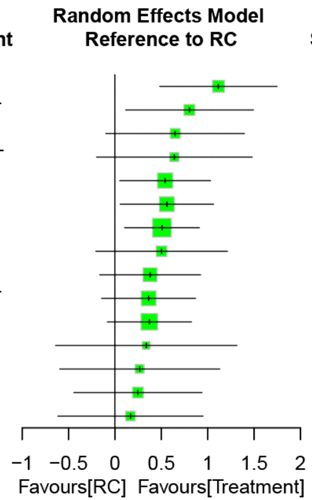

Treatment	SMD	95% CI	SUCRA
SP+RET	1.12	[0.49; 1.75]	0.93
WP+RET	0.80	[0.12; 1.49]	0.77
DP+RET	0.65	[−0.10; 1.40]	0.64
WP+MET	0.64	[−0.20; 1.48]	0.63
RET	0.54	[0.05; 1.03]	0.60
DP+MET	0.56	[0.06; 1.06]	0.60
MET	0.51	[0.11; 0.91]	0.56
DP	0.50	[−0.20; 1.21]	0.53
SP	0.38	[−0.16; 0.92]	0.43
MP+MET	0.36	[−0.14; 0.87]	0.42
MP	0.37	[−0.08; 0.82]	0.41
SP+MET	0.34	[−0.64; 1.32]	0.40
Collagen	0.27	[−0.59; 1.13]	0.37
WP	0.25	[−0.44; 0.94]	0.33
MP+RET	0.17	[−0.61; 0.95]	0.28

Supplementary figure S9. Treatment effects for chair stand within time frames.

Overall follow-up duration

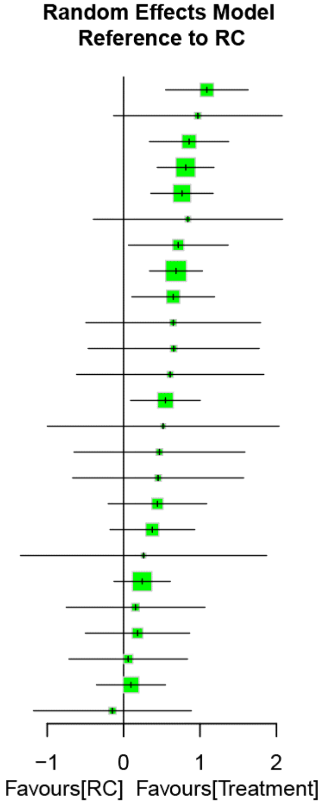

Treatment	SMD	95% CI	SUCRA
WP+MET	1.09	[0.55; 1.63]	0.86
Casein+RET	0.97	[−0.13; 2.07]	0.74
MP+MET	0.86	[0.34; 1.38]	0.73
WP+RET	0.81	[0.44; 1.18]	0.73
MP+RET	0.76	[0.36; 1.17]	0.69
Collagen+RET	0.84	[−0.39; 2.08]	0.67
SP+RET	0.72	[0.06; 1.37]	0.65
RET	0.69	[0.34; 1.03]	0.63
DP+MET	0.65	[0.11; 1.19]	0.61
Meat+MET	0.65	[−0.49; 1.79]	0.59
Rice+MET	0.66	[−0.46; 1.77]	0.57
Rice+RET	0.61	[−0.61; 1.83]	0.55
DP+RET	0.55	[0.09; 1.00]	0.53
MP+AET	0.52	[−1.00; 2.03]	0.53
AET	0.47	[−0.65; 1.59]	0.48
WP+AET	0.45	[−0.67; 1.57]	0.47
Casein+MET	0.44	[−0.20; 1.09]	0.46
SP+MET	0.38	[−0.18; 0.93]	0.43
Meat+RET	0.26	[−1.35; 1.87]	0.38
MET	0.24	[−0.13; 0.61]	0.31
Casein	0.16	[−0.75; 1.06]	0.30
DP	0.18	[−0.50; 0.87]	0.28
MP	0.06	[−0.71; 0.84]	0.24
WP	0.10	[−0.35; 0.55]	0.22
SP	−0.15	[−1.18; 0.89]	0.19

Follow up ≤3 months

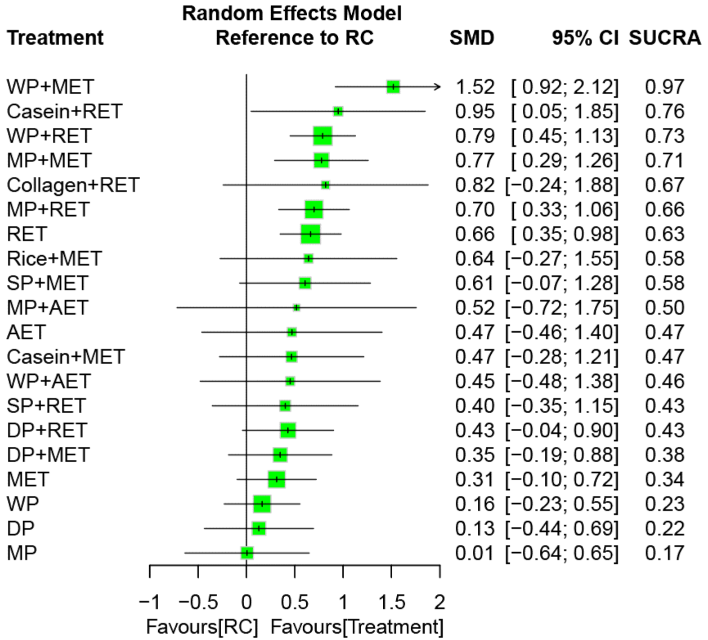

Treatment	SMD	95% CI	SUCRA
WP+MET	1.52	[0.92; 2.12]	0.97
Casein+RET	0.95	[0.05; 1.85]	0.76
WP+RET	0.79	[0.45; 1.13]	0.73
MP+MET	0.77	[0.29; 1.26]	0.71
Collagen+RET	0.82	[−0.24; 1.88]	0.67
MP+RET	0.70	[0.33; 1.06]	0.66
RET	0.66	[0.35; 0.98]	0.63
Rice+MET	0.64	[−0.27; 1.55]	0.58
SP+MET	0.61	[−0.07; 1.28]	0.58
MP+AET	0.52	[−0.72; 1.75]	0.50
AET	0.47	[−0.46; 1.40]	0.47
Casein+MET	0.47	[−0.28; 1.21]	0.47
WP+AET	0.45	[−0.48; 1.38]	0.46
SP+RET	0.40	[−0.35; 1.15]	0.43
DP+RET	0.43	[−0.04; 0.90]	0.43
DP+MET	0.35	[−0.19; 0.88]	0.38
MET	0.31	[−0.10; 0.72]	0.34
WP	0.16	[−0.23; 0.55]	0.23
DP	0.13	[−0.44; 0.69]	0.22
MP	0.01	[−0.64; 0.65]	0.17

Follow up >3 months, ≤6 months

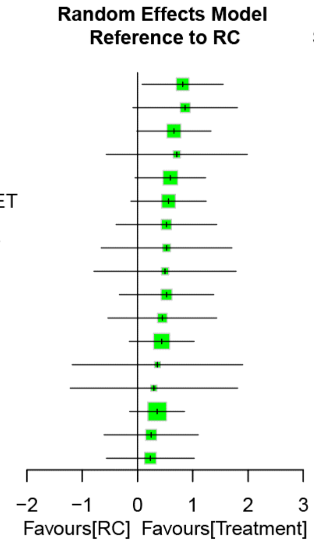

Treatment	SMD	95% CI	SUCRA
MP+RET	0.82	[0.08; 1.55]	0.74
SP+RET	0.86	[−0.08; 1.81]	0.74
WP+RET	0.66	[−0.01; 1.33]	0.64
Rice+RET	0.71	[−0.56; 1.99]	0.62
RET	0.59	[−0.04; 1.23]	0.58
Casein+MET	0.56	[−0.12; 1.24]	0.58
DP+RET	0.52	[−0.39; 1.43]	0.53
Meat+MET	0.53	[−0.66; 1.71]	0.53
MP+MET	0.50	[−0.79; 1.78]	0.51
DP+MET	0.53	[−0.33; 1.38]	0.50
WP+MET	0.45	[−0.53; 1.43]	0.47
SP+MET	0.44	[−0.15; 1.02]	0.46
Meat+RET	0.36	[−1.18; 1.90]	0.44
Rice+MET	0.30	[−1.22; 1.81]	0.41
MET	0.36	[−0.14; 0.85]	0.41
DP	0.25	[−0.60; 1.10]	0.35
Casein	0.23	[−0.56; 1.03]	0.34

Follow up >6 months

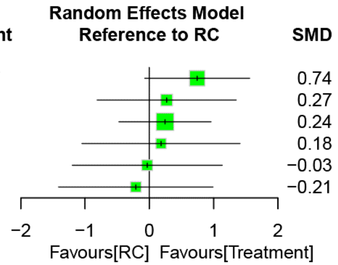

Treatment	SMD	95% CI	SUCRA
DP+MET	0.74	[−0.07; 1.56]	0.85
DP	0.27	[−0.81; 1.35]	0.57
MET	0.24	[−0.47; 0.96]	0.56
SP+MET	0.18	[−1.05; 1.41]	0.51
DP+RET	−0.03	[−1.20; 1.13]	0.38
SP	−0.21	[−1.41; 0.99]	0.28

Supplementary figure S10. Treatment effects for timed up-and-go performance within time frames.

Overall follow-up duration

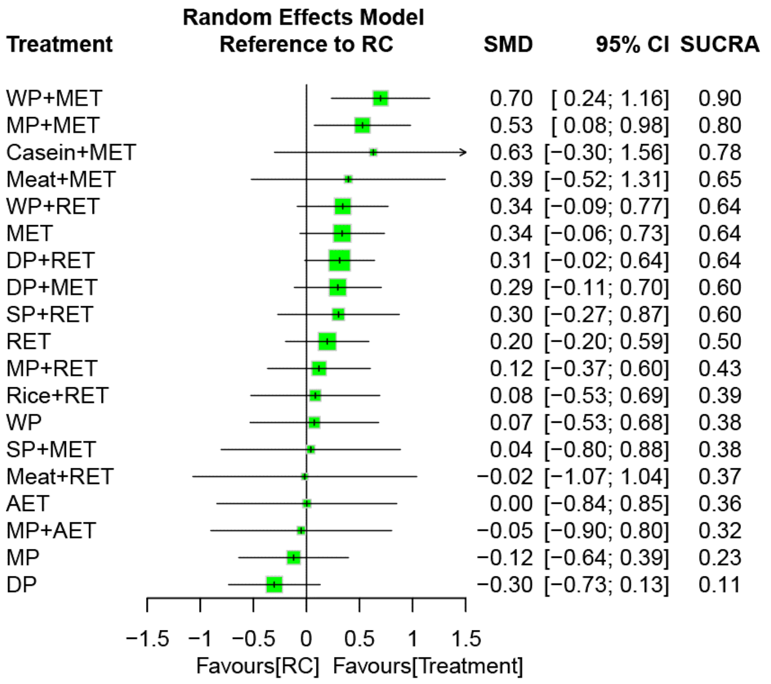

Follow up ≤3 months

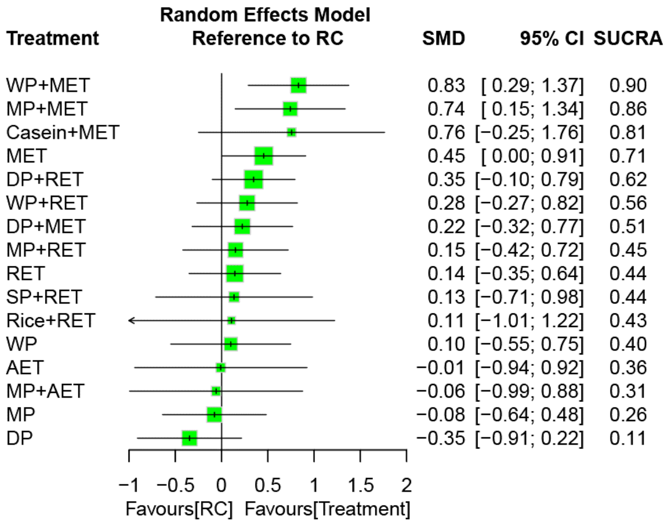

Follow up >3 months, ≤6 months

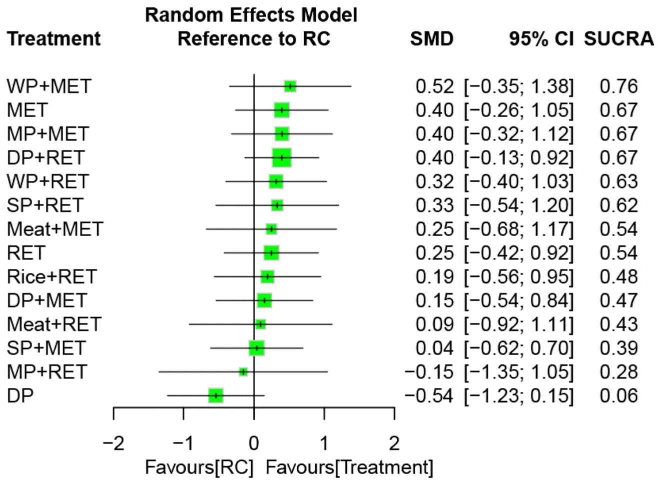

Follow up >6 months

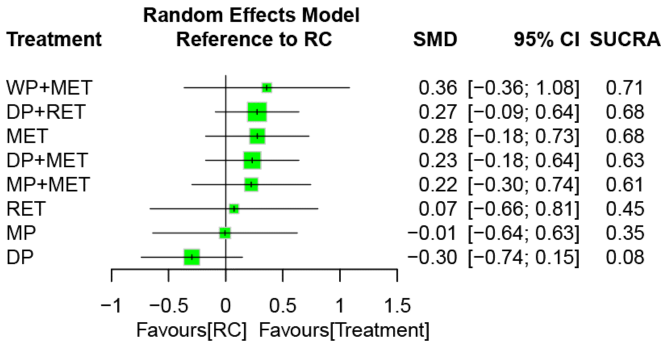

Supplementary figure S11. Treatment effects for global mobility (SPPB) within time frames.

Overall follow-up duration

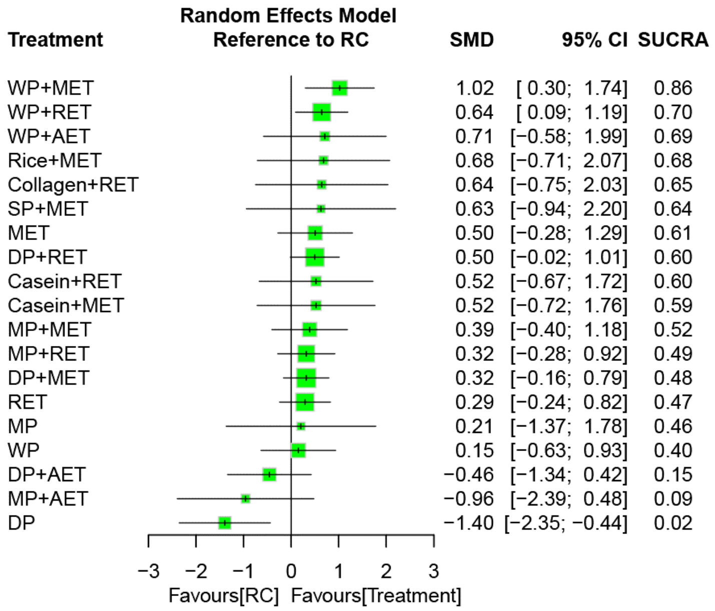

Follow up ≤3 months

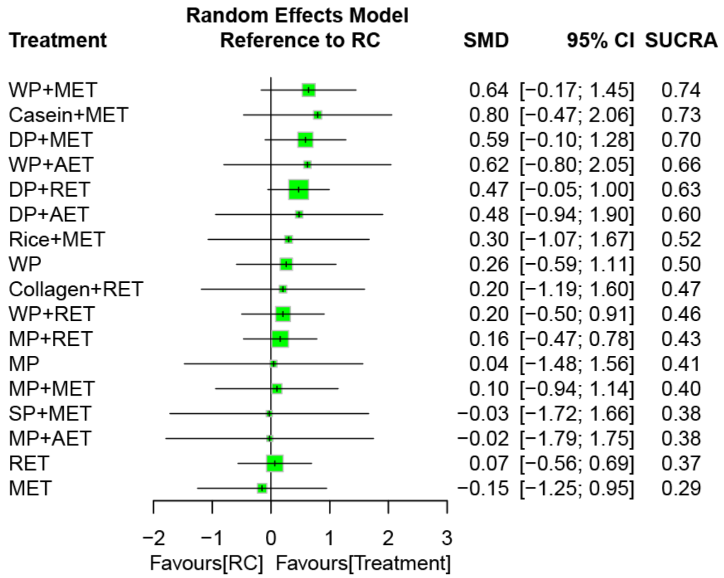

Follow up >3 months, ≤6 months

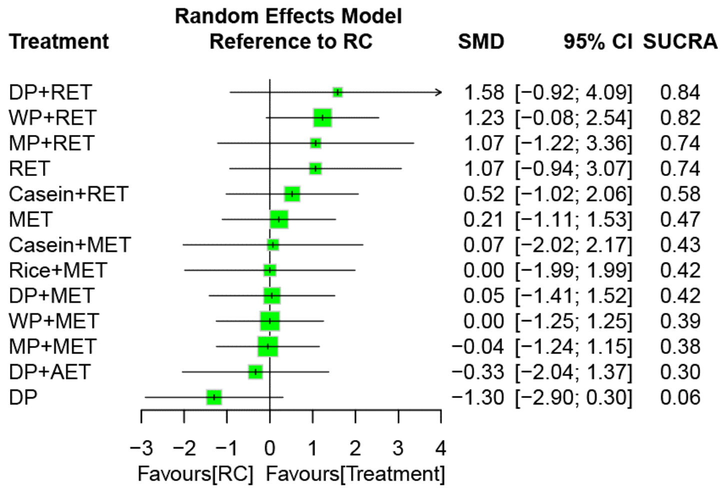

Follow up >6 months

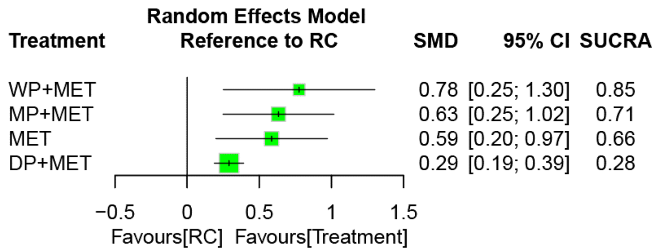

Supplementary figure S12. Inconsistency assessment results for walking speed.

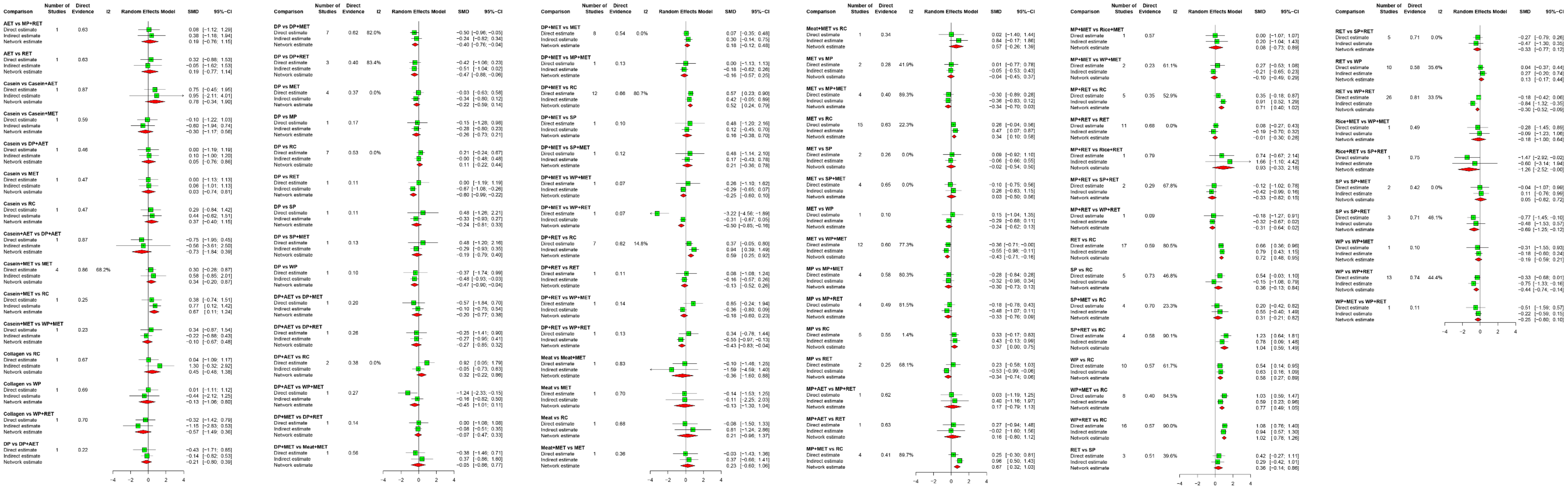

Supplementary figure S13. Inconsistency assessment results for chair rise.

Comparison	Number of Studies	Direct Evidence	I2	SMD	95%-CI
**Casein vs Casein+MET**					
Direct estimate	1	0.86		−0.25	[−1.29; 0.79]
Indirect estimate				−0.70	[−3.25; 1.86]
Network estimate				−0.31	[−1.28; 0.65]
**Casein vs MET**					
Direct estimate	1	0.74		−0.11	[−1.15; 0.93]
Indirect estimate				−0.00	[−1.74; 1.74]
Network estimate				−0.08	[−0.97; 0.81]
**Casein vs RC**					
Direct estimate	1	0.74		0.14	[−0.90; 1.18]
Indirect estimate				0.28	[−1.49; 2.04]
Network estimate				0.18	[−0.72; 1.08]
**Casein+MET vs MET**					
Direct estimate	2	0.78	0.0%	0.08	[−0.70; 0.86]
Indirect estimate				0.77	[−0.70; 2.25]
Network estimate				0.23	[−0.45; 0.92]
**Casein+MET vs RC**					
Direct estimate	1	0.46		0.39	[−0.65; 1.43]
Indirect estimate				0.57	[−0.39; 1.54]
Network estimate				0.49	[−0.22; 1.20]
**Casein+MET vs WP+MET**					
Direct estimate	1	0.40		0.15	[−1.02; 1.32]
Indirect estimate				−0.61	[−1.56; 0.34]
Network estimate				−0.31	[−1.05; 0.43]
**DP vs DP+MET**					
Direct estimate	2	0.52	0.0%	−0.33	[−1.07; 0.42]
Indirect estimate				−0.88	[−1.66; −0.10]
Network estimate				−0.59	[−1.13; −0.05]
**DP vs DP+RET**					
Direct estimate	1	0.26		0.08	[−1.01; 1.18]
Indirect estimate				−0.47	[−1.11; 0.17]
Network estimate				−0.33	[−0.88; 0.22]
**DP vs MET**					
Direct estimate	2	0.45	0.0%	−0.14	[−0.89; 0.61]
Indirect estimate				−0.13	[−0.81; 0.56]
Network estimate				−0.13	[−0.64; 0.37]
**DP vs MP**					
Direct estimate	1	0.57		0.22	[−0.83; 1.27]
Indirect estimate				0.04	[−1.15; 1.24]
Network estimate				0.14	[−0.64; 0.93]
**DP vs RC**					
Direct estimate	4	0.69	0.0%	0.05	[−0.48; 0.58]
Indirect estimate				0.29	[−0.51; 1.09]
Network estimate				0.12	[−0.32; 0.57]
**DP vs RET**					
Direct estimate	1	0.20		0.25	[−0.86; 1.36]
Indirect estimate				−0.54	[−1.10; 0.02]
Network estimate				−0.38	[−0.88; 0.12]
**DP vs WP**					
Direct estimate	1	0.18		−0.85	[−2.16; 0.47]
Indirect estimate				0.30	[−0.31; 0.91]
Network estimate				0.10	[−0.46; 0.65]
**DP+MET vs DP+RET**					
Direct estimate	1	0.28		−0.23	[−1.22; 0.76]
Indirect estimate				0.45	[−0.16; 1.06]
Network estimate				0.26	[−0.26; 0.78]
**DP+MET vs MET**					
Direct estimate	3	0.56	52.3%	0.38	[−0.22; 0.99]
Indirect estimate				0.56	[−0.13; 1.24]
Network estimate				0.46	[0.01; 0.91]
**DP+MET vs RC**					
Direct estimate	5	0.76	88.5%	0.84	[0.37; 1.30]
Indirect estimate				0.33	[−0.51; 1.16]
Network estimate				0.71	[0.31; 1.12]

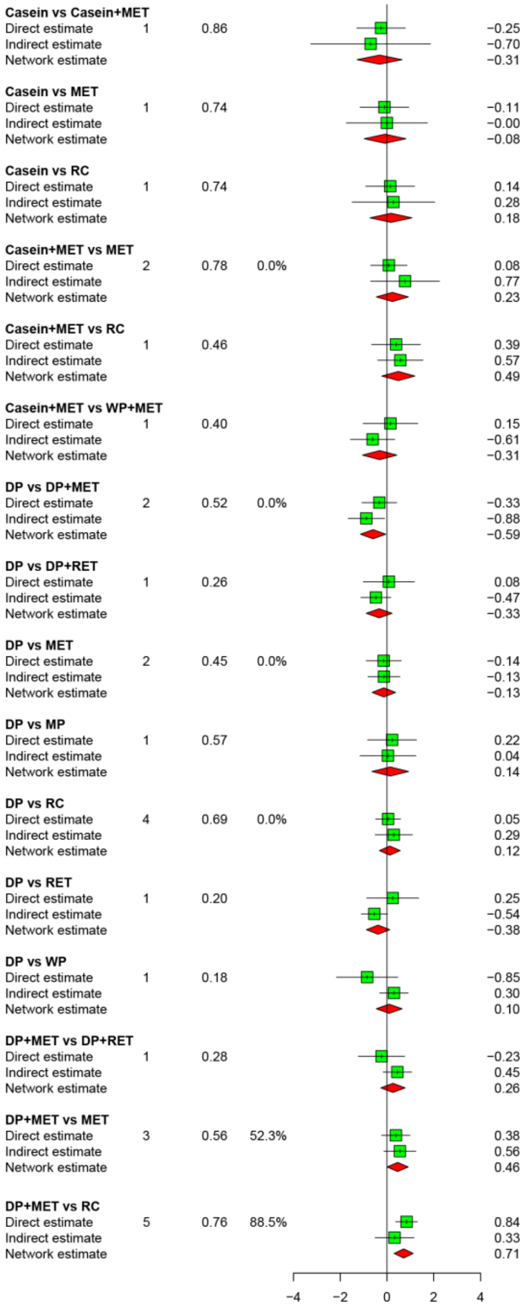

Comparison	Number of Studies	Direct Evidence	I2	SMD	95%-CI
**DP+MET vs WP+MET**					
Direct estimate	1	0.17		−0.22	[−1.51; 1.07]
Indirect estimate				−0.06	[−0.64; 0.53]
Network estimate				−0.08	[−0.62; 0.45]
**DP+RET vs RC**					
Direct estimate	7	0.88	75.7%	0.43	[0.03; 0.83]
Indirect estimate				0.64	[−0.43; 1.72]
Network estimate				0.45	[0.08; 0.83]
**DP+RET vs RET**					
Direct estimate	2	0.32	0.0%	0.12	[−0.67; 0.90]
Indirect estimate				−0.13	[−0.67; 0.42]
Network estimate				−0.05	[−0.49; 0.40]
**MET vs MP+MET**					
Direct estimate	1	0.22		−0.08	[−1.26; 1.11]
Indirect estimate				−0.61	[−1.24; 0.02]
Network estimate				−0.49	[−1.05; 0.06]
**MET vs RC**					
Direct estimate	8	0.73	29.2%	0.39	[0.00; 0.78]
Indirect estimate				−0.11	[−0.74; 0.53]
Network estimate				0.26	[−0.07; 0.59]
**MET vs SP**					
Direct estimate	1	0.68		0.24	[−1.01; 1.48]
Indirect estimate				0.73	[−1.08; 2.53]
Network estimate				0.40	[−0.63; 1.42]
**MET vs SP+MET**					
Direct estimate	3	0.66	0.0%	−0.25	[−0.93; 0.43]
Indirect estimate				0.11	[−0.84; 1.07]
Network estimate				−0.13	[−0.68; 0.43]
**MET vs WP**					
Direct estimate	1	0.19		−0.07	[−1.18; 1.05]
Indirect estimate				0.30	[−0.24; 0.84]
Network estimate				0.23	[−0.26; 0.72]
**MET vs WP+MET**					
Direct estimate	5	0.68	94.3%	−0.66	[−1.16; −0.16]
Indirect estimate				−0.30	[−1.02; 0.43]
Network estimate				−0.54	[−0.96; −0.13]
**MP vs MP+RET**					
Direct estimate	1	0.42		−0.34	[−1.52; 0.84]
Indirect estimate				−0.82	[−1.81; 0.18]
Network estimate				−0.62	[−1.38; 0.14]
**MP vs RC**					
Direct estimate	1	0.51		−0.27	[−1.32; 0.77]
Indirect estimate				0.24	[−0.82; 1.31]
Network estimate				−0.02	[−0.77; 0.73]
**MP+MET vs MP+RET**					
Direct estimate	1	0.27		0.67	[−0.41; 1.76]
Indirect estimate				−0.05	[−0.72; 0.62]
Network estimate				0.15	[−0.42; 0.72]
**MP+MET vs RC**					
Direct estimate	3	0.56	0.0%	0.62	[−0.05; 1.30]
Indirect estimate				0.91	[0.15; 1.67]
Network estimate				0.75	[0.24; 1.25]
**MP+MET vs WP+MET**					
Direct estimate	1	0.34		0.01	[−0.99; 1.01]
Indirect estimate				−0.08	[−0.79; 0.64]
Network estimate				−0.05	[−0.63; 0.53]
**MP+RET vs RC**					
Direct estimate	2	0.19	0.0%	0.51	[−0.29; 1.32]
Indirect estimate				0.62	[0.23; 1.01]
Network estimate				0.60	[0.25; 0.95]
**MP+RET vs RET**					
Direct estimate	10	0.68	79.8%	0.16	[−0.19; 0.50]
Indirect estimate				−0.03	[−0.54; 0.48]
Network estimate				0.10	[−0.19; 0.38]
**MP+RET vs Rice+RET**					
Direct estimate	1	0.79		−0.16	[−1.48; 1.17]
Indirect estimate				1.27	[−1.33; 3.86]
Network estimate				0.14	[−1.04; 1.32]

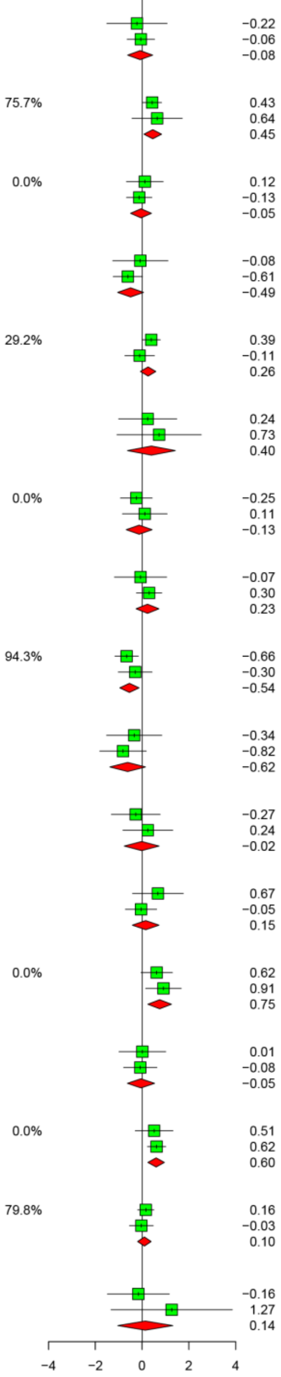

Comparison	Number of Studies	Direct Evidence	I2	SMD	95%-CI
**MP+RET vs SP+RET**					
Direct estimate	1	0.22		−0.58	[−1.95; 0.79]
Indirect estimate				0.18	[−0.54; 0.90]
Network estimate				0.02	[−0.62; 0.65]
**MP+RET vs WP+RET**					
Direct estimate	2	0.19	0.0%	0.27	[−0.49; 1.03]
Indirect estimate				−0.16	[−0.53; 0.21]
Network estimate				−0.08	[−0.41; 0.26]
**RET vs RC**					
Direct estimate	9	0.54	84.8%	0.77	[0.38; 1.17]
Indirect estimate				0.19	[−0.24; 0.61]
Network estimate				0.50	[0.21; 0.79]
**SP vs RC**					
Direct estimate	1	0.70		−0.41	[−1.63; 0.81]
Indirect estimate				0.49	[−1.37; 2.36]
Network estimate				−0.14	[−1.16; 0.88]
**SP+MET vs RC**					
Direct estimate	3	0.74	78.3%	0.33	[−0.30; 0.97]
Indirect estimate				0.52	[−0.56; 1.60]
Network estimate				0.38	[−0.16; 0.93]
**SP+RET vs RC**					
Direct estimate	1	0.37		0.30	[−0.74; 1.34]
Indirect estimate				0.75	[−0.04; 1.54]
Network estimate				0.58	[−0.05; 1.21]
**WP vs RC**					
Direct estimate	7	0.73	57.0%	−0.04	[−0.51; 0.42]
Indirect estimate				0.22	[−0.55; 0.99]
Network estimate				0.03	[−0.37; 0.42]
**WP+MET vs RC**					
Direct estimate	2	0.30	0.0%	0.56	[−0.24; 1.35]
Indirect estimate				0.90	[0.38; 1.43]
Network estimate				0.80	[0.36; 1.24]
**WP+RET vs RC**					
Direct estimate	9	0.60	76.3%	0.82	[0.44; 1.20]
Indirect estimate				0.45	[−0.02; 0.93]
Network estimate				0.67	[0.38; 0.97]
**RET vs SP+RET**					
Direct estimate	2	0.63	0.0%	0.09	[−0.68; 0.85]
Indirect estimate				−0.36	[−1.36; 0.63]
Network estimate				−0.08	[−0.69; 0.52]
**RET vs WP**					
Direct estimate	5	0.56	92.6%	0.72	[0.16; 1.29]
Indirect estimate				0.17	[−0.46; 0.80]
Network estimate				0.48	[0.05; 0.90]
**RET vs WP+RET**					
Direct estimate	20	0.84	51.5%	−0.19	[−0.44; 0.06]
Indirect estimate				−0.09	[−0.65; 0.48]
Network estimate				−0.17	[−0.40; 0.06]
**Rice+RET vs SP+RET**					
Direct estimate	1	0.79		−0.42	[−1.75; 0.91]
Indirect estimate				1.00	[−1.58; 3.57]
Network estimate				−0.12	[−1.30; 1.06]
**SP vs SP+MET**					
Direct estimate	1	0.73		−0.39	[−1.62; 0.84]
Indirect estimate				−0.88	[−2.91; 1.16]
Network estimate				−0.52	[−1.58; 0.53]
**SP+RET vs WP+RET**					
Direct estimate	1	0.31		0.00	[−1.11; 1.11]
Indirect estimate				−0.13	[−0.88; 0.61]
Network estimate				−0.09	[−0.71; 0.53]
**WP vs WP+MET**					
Direct estimate	1	0.23		−0.31	[−1.48; 0.85]
Indirect estimate				−0.91	[−1.55; −0.27]
Network estimate				−0.77	[−1.33; −0.21]
**WP vs WP+RET**					
Direct estimate	5	0.57	88.5%	−1.02	[−1.59; −0.46]
Indirect estimate				−0.15	[−0.80; 0.50]
Network estimate				−0.65	[−1.07; −0.22]

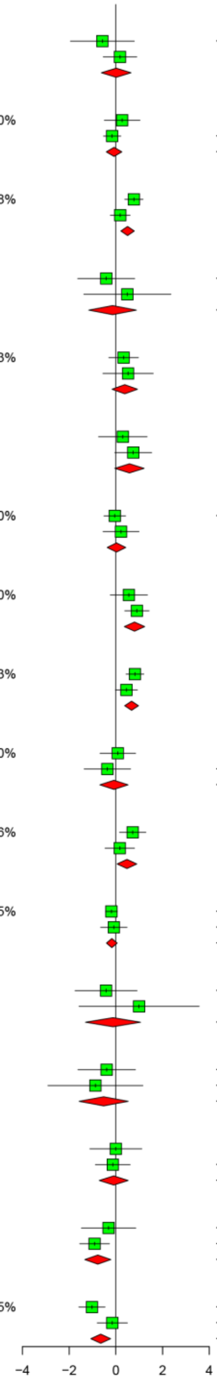

Supplementary figure S14. Inconsistency assessment results for timed up-and-go performance.

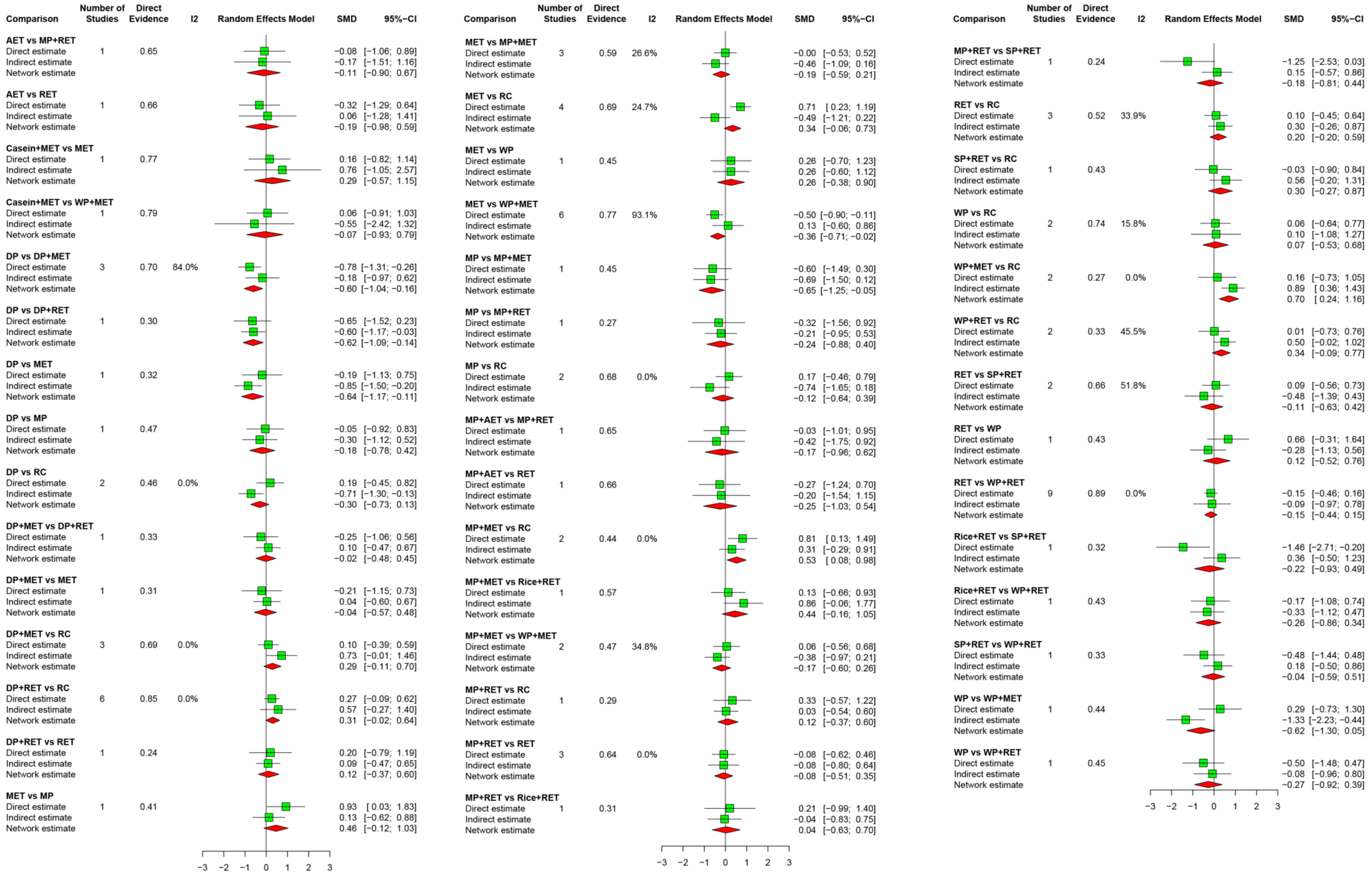

Supplementary figure S15. Inconsistency assessment results for global mobility (SPPB).

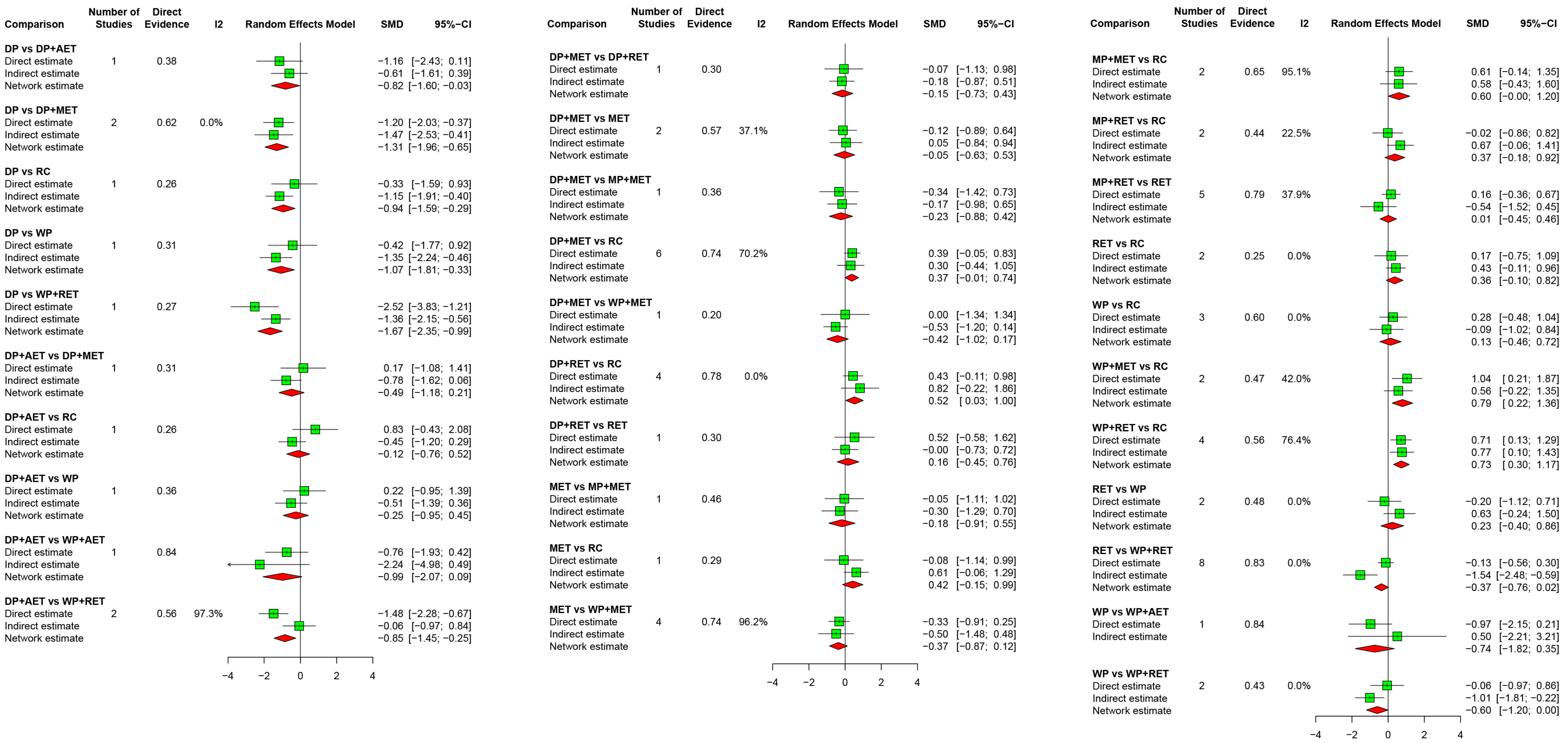

Supplementary figure S16. Funnel plots for treatment outcomes of (A) muscle mass, (B) handgrip strength, (C) leg muscle strength, (D) walk speed, (E) chair rise ability, (F) timed up and go, and (G) SPPB.

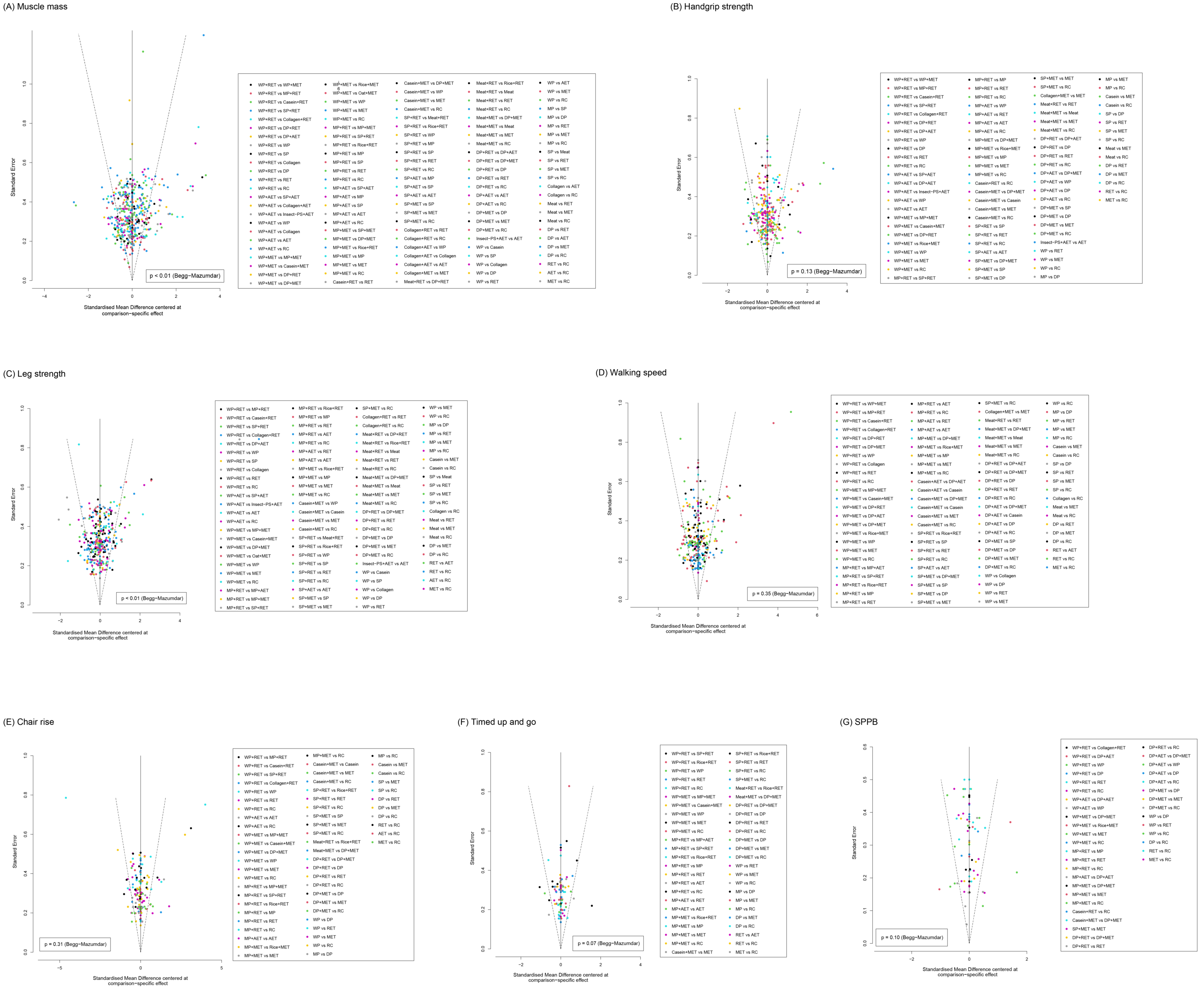

Supplement: Supplementary file 1 [file nutrients-18-01409-s001.zip › nutrients-4191078-supplementary figures S1- S16_title page merged.pdf]
