# Supplementary material for: Explore the Optimal Treatment Regimen Across Combinations of Variate Protein Sources and Exercise Modalities and Its Associated Factors in Older Adults: A Network Meta-Analysis and Meta-Regression of Randomized Controlled Trials
Source: Nutrients. 2026 Apr 29;18(9):1409. doi: 10.3390/nu18091409 (PMC13165454; doi:10.3390/nu18091409)
Supplement: Supplementary file 1 [file nutrients-18-01409-s001.zip › nutrients-4191078 -supplementary tables S1- S19_titled (ref 380).pdf]

Supplementary tables

Explore the Optimal Treatment Regimen across Combinations of Variate Protein Sources and Exercise Modalities and Its Associated Factors in Older Adults: A Network Meta-Analysis and Meta-Regression of Randomized Controlled Trials

Che-Li Lin [1,2], Shih-Wei Huang [3,4], Hung-Chou Chen [4,5], Mao-Hua Huang [6], Tsan-Hon Liou [3,4] and Chun-De Liao [5,7*]

[1] Department of Orthopedic Surgery, Shuang Ho Hospital, Taipei Medical University, New Taipei City 23561, Taiwan; 11010@s.tmu.edu.tw (C.-L. L.)
[2] Department of Orthopedics, School of Medicine, College of Medicine, Taipei Medical University, Taipei 11031, Taiwan
[3] Department of Physical Medicine and Rehabilitation, Wan Fang Hospital, Taipei Medical University, Taipei 116079, Taiwan; 113128@w.tmu.edu.tw (S.-W. H.); peter_liou@s.tmu.edu.tw (T.-H. L.)
[4] Department of Physical Medicine and Rehabilitation, School of Medicine, College of Medicine, Taipei Medical University, Taipei 110301, Taiwan; 10462@s.tmu.edu.tw (H.-C.C.)
[5] Department of Physical Medicine and Rehabilitation, Shuang Ho Hospital, Taipei Medical University, New Taipei City 235041, Taiwan
[6] Department of Biochemistry, University of Washington, Seattle, Washington, 98015, USA; huangkevin507@gmail.com
[7] International Ph.D. Program in Gerontology and Long-Term Care, College of Nursing, Taipei Medical University, Taipei 110301, Taiwan

[*] **Correspondence** Chun-De Liao, PT, PhD Tel: 886-2-2249-0088 ext. 1600 Fax: 886-2-2248-0577 Email: 08415@s.tmu.edu.tw

Title of table	Page
Table S1. Database search formulas.	1
Table S2. Summary of included study characteristics.	4
Table S3. Outcome measures identified among the included trials.	21
Table S4. Direct and network estimates for muscle mass.	28
Table S5. Direct and network estimates for handgrip strength.	31
Table S6. Direct and network estimates for leg strength.	34
Table S7. Direct and network estimates for walking speed.	37
Table S8. Direct and network estimates for chair stand.	40
Table S9. L Direct and network estimates for timed up-and-go performance.	43
Table S10. Direct and network estimates for global physical mobility (SPPB).	45
Table S11. Summary for meta-regression results.	47
Table S12. Assessment for treatment safety.	49
Table S13. GRADE certainty rating of treatment efficacy for muscle mass gain.	66
Table S14. GRADE certainty rating of treatment efficacy for handgrip strength outcome.	67
Table S15. GRADE certainty rating of treatment efficacy for leg strength outcome.	68
Table S16. GRADE certainty rating of treatment efficacy for walking speed outcome.	69
Table S17. GRADE certainty rating of treatment efficacy for chair rise outcome.	70
Table S18. GRADE certainty rating of treatment efficacy for timed up-and-go outcome.	71
Table S19. GRADE certainty rating of treatment efficacy for global mobility (SPPB) outcome.	72

Supplementary table S1. Database search formulas.

Data base	Search terms for query
**Pubmed**	
#1	((elder adults) OR (elderly)) OR older adults
#2	((untrained) OR (sedentary)) OR (inactivity)
#3	((((community dwelling) OR (nursing home resident)) OR (facility resident)) OR (institutionalized resident)) OR (hospitalized inpatient)
#4	((((((sarcopenia) OR (frailty)) OR (frail)) OR (prefrail)) OR (dynapenia)) OR (dynapaenia)) OR (physically dependent)
#5	((((protein supplement) OR (protein supplementation)) OR (dietary protein)) OR (high protein diet)) OR (protein enriched foods)
#6	(((((((((((((animal protein) OR (plant protein)) OR (whey protein)) OR (casein)) OR (soy protein)) OR (pea protein)) OR (peanut)) OR (nut)) OR (seed)) OR (rice)) OR (oat)) OR (collagen)) OR (beef)) OR (meat)) OR (milk protein)
#7	(((((((exercise training) OR (resistance training)) OR (aerobic training)) OR (physical activity)) OR (physical therapy)) OR (rehabilitation)) OR (strength training)) OR (strengthening exercise) AND (clinicaltrial[Filter] OR randomized controlled trial[Filter])
#8	(((#2) OR #3) OR #4) AND #1 AND (clinicaltrial[Filter] OR randomized controlled trial [Filter])
#9	(#5 OR #6) AND (clinicaltrial[Filter] OR randomized controlled trial [Filter])
#10	(((#8) AND #9) AND #7
**Physiotherapy Evidence Database (PEDro)**	
	Method: clinical trial
	Abstract & Title:
#1	order adults
#2	sarcopenia
#3	frailty
#4	exercise training
#5	physical activity
#6	strength training
#7	protein supplementation
#8	high-protein diet
#9	dietary protein
**China knowledge resource integrated database**	
#1	order adults

#2	exercise training
#3	protein supplementation
#4	randomized controlled trial
#5	#1 AND #2 AND #3 AND #4

(continued)

Table S1. (continued)

Data base	Search terms for query
**Excerpta Medica dataBASE (EMBASE)**	
#1	order adults
#2	sarcopenia
#3	frailty
#4	dynapenia
#5	sedentary/untrained
#6	exercise training
#7	physical activity
#8	strength training
#9	protein supplementation
#10	dietary protein
#11	#1 OR #2 OR #3 OR #4 OR #5
#12	#6 OR #7 OR #8
#13	#9 OR #10
#14	#14 AND #15 AND #16 AND ([systematic review]/lim OR [meta analysis]/lim OR [randomized controlled trial]/lim) AND [humans]/lim
**Cochrane Library Database**	
#1	order adults
#2	sarcopenia
#3	frailty
#4	sedentary/untrained
#5	exercise training
#6	physical activity
#7	strength training
#8	protein supplementation
#9	dietary protein
#10	randomized controlled trial
#11	#1 OR #2 OR #3 OR #4
#12	#5 OR #6 OR #7
#13	#8 AND #9
#14	#11 AND #12 AND #13
Google Scholar	
#1	allintitle: elderly OR older adults
#2	allintitle: untrained OR sarcopenia OR frailty
#3	allintitle: exercise training OR physical activity
#4	allintitle: “protein supplementation” OR “dietary protein”
#5	allintitle: randomized controlled trial

Table S2. Summary of included study characteristics.

Study (year) [Reference No.][a]	Country (area)	Study arm	Age (years) Mean	BMI (kg/m^2) Mean	Sex (n) Women	Sex (n) Men	Study sample (n)	Health status (medical condition)	Living status[d]	Protein supplementation: Frequency (day /week)	Protein supplementation: Intake amount (g/d or g/session)	Protein supplementation: Compliance (%)	Exercise intervention: Modality	Exercise intervention: Intensity (% 1-RM/RPE/HR_{max})	Exercise intervention: Frequency (session /week)	Exercise intervention: Duration (week)	Exercise intervention: Compliance (%)	Outcome measures[e] Muscle mass/volume: Whole body lean	Muscle mass/volume: ALM	Muscle mass/volume: Muscle volume	Strength: Handgrip strength	Strength: Leg strength	Mobility: Walk speed	Mobility: Chair rise	Mobility: TUG	Mobility: SPPB	Follow-up time point (week)
Aas 2020 [109]	Norway	MP+RET	86.6	26.1	7	4	11	Mobility-limited	1	7	34	NR	RET	80–90% 1-RM	3	10	95.8		V	V		V	V	V			0, 12
	(Europe)	RC	82.6	26.3	8	3	11	older adults																			
Amasene 2021 [124]	Spain	WP+RET	82.9	27.4	12	9	21	Post-Hospitalized	2	2	20	NR	RET	70% 1-RM	2	12	NR	V	V	V	V		V	V		V	0, 12
	(Europe)	PLA+RET	81.2	31.1	10	10	20	sarcopenic older adults																			
Arciero 2014 [125]	USA	WP+MET	52.0	28.0	8	9	17	Inactive middle-aged	1	4	60	75	MET (RET, AET, StreE),	RPE: 6/10 for AET;	4	16	85	V									0, 4
	(America)	WP+RET	47.0	29.0	17	5	22	adults					supervised, 45~60 min, whole-body ex	7-9/10 for RET (10-12 RM)			85										
		WP	50.0	28.0	11	7	18						RET, supervised, 60 min, UE/LE ex														
Arentson-Lantz 2019 [127]	USA	WP+MET	69.0	27.4	5	5	10	Immobilized older adults	2	7	16~20[f]	NR	MET (RET, BalaT, StreE), supervised,	NR	7	2	NR	V	V			V	V	V		V	0, 1, 2
	(America)	DP+MET	68.0	25.2	3	7	10				0.97~1.17 g・kg^{-1}・ d^{-1}		45 min, structured rehabilitation														
Arentson-Lantz 2020 [126]	USA	WP+MET	68.0	25.2	3	7	10	Immobilized older adults	2	7	14.6 (0.18 g・kg^{-1}・ d^{-1})	NR	MET (RET, BalaT, StreE), supervised,	NR	7	2	NR	V	V			V	V	V		V	0, 1, 2
	(America)	PLA+MET	68.0	28.0	3	7	10				13.2 (0.18 g・kg^{-1}・ d^{-1})		45 min, structured rehabilitation														
Arnarson 2013 [128]	Iceland	WP+RET	73.3	28.1	94[c]	67[c]	83	Healthy community-	1	3	20	NR	RET	75-80% 1-RM	3	12	NR	V	V			V	V		V		0, 6
	(Europe)	PLA+RET	74.6	29.4			78	dwelling elderly																			
Assantachai 2020 [129]	Thailand	Meat+MET	81.5	19.7	22[c]	13[c]	10	Community-dwelling	1	7	4.5	95.2	High-level physical activity	NR	7	24	NR		V		V	V	V				0, 24
	(Asia)	PLA+MET	81.5	19.5			8	older adults with																			
		Meat	81.5	19.7			10	sarcopenia.				95.2															
		PLA	81.5	19.5			7																				
Atherton 2020 [130]	Ireland	WP+RET	70.5[c]	NR	0	10	10	Untrained, recreationally	1	3	40	100	RET	70% 1-RM	3	10	NR	V			V	V					0, 10
	(Europe)	PLA+RET			0	9	9	active older adults				100															
Bagheri 2022 [131]	Iran	WP+RET	67.3	21.2	0	14	14	Untrained healthy older adults	1	7	18	>90	RET	60–80% 1-RM	3	8	>90	V				V					0, 8
	(Asia)	PLA+RET	68.4	21.9	0	14	14					>90															
Bauer 2024 [132]	Netherlands	WP+RET	62.7	32.9	34	29	63	Overweight and	1	7	20~40	91	RET: 60 min, supervised, whole-body Ex	AET: 85~95% HR_{max};	3	13	76.9	V	V		V		V				0, 13
	(Europe)	DP+RET	62.0	32.0	34	21	55	obese older adults		7	1.3 g・kg^{-1}・ d^{-1}	87	MET (AET, RET): 60 min, supervised,	RET: 80% 1-RM	3		70.9										
		WP+MET	65.7	33.3	41	73	114			7		90	UE/LE ex	(12 RM), 3 set x 12 rep	3		81.2										
Beck 2016 [133]	Denmark	MP+MET	87.0	23.4	42	20	62	Physically dependent	2	7	9	100	moderate intensity (3~4/CR-10); 80	moderate intensity	2	11	100				V			V			0, 11, 27
	(Europe)	RC	86.0	23.4	46	13	59	nursing-home residents						(3~4/CR-10); 80													
Bell 2017 [134]	Canada	WP+MET	71.0	28.9	0	25	25	Untrained healthy	1	7	60	87	MET (AET, RET),	80% 1-RM;	3	12	95	V	V			V					0, 6, 12
	(America)	PLA+MET	74.0	28.1	0	24	24	older adults				92	supervised, 60 min, UE/LE ex	90% HR_{max} for AET			94										
Bemben 2010 [135];	USA	WP+Cre+RET	57.2	28.7	0	11	11	Untrained healthy	1	3	35	100	RET	80% 1-RM	3	14	100	V	V	V		V					0, 14
Eliot 2008 [176]	(America)	WP+RET	58.2	28.6	0	11	11	middle-aged and																			
		Cre+RET	56.1	29.1	0	10	10	older adults																			
		PLA+RET	56.1	31.3	0	10	10																				
Bernabei 2022 [136]	Italy	DP+MET	79.1	28.6	547	212	759	Elderly with physical	1	7	1.0-1.2 g・kg^{-1}・ d^{-1}	78.6	MET (AET, RET)	Moderate, RPE 13~16/20	3~6	144	67~73.5		V		V					V	0, 104, 144
	(Europe)	RC	78.8	28.6	541	218	759	frailty and sarcopenia						(15-point Borg scale)			65.9										
Biesek 2021 [137]	Brazil	WP+MET	71.7	30.3	18	0	18	Prefrail Community-	1	3	21	50.1-74.9	MET (RET, BalaT)	10~15% body weight	2	12	NR	V	V	V	V	V					0, 12
	(Europe)	PLA+MET	69.7	29.3	18	0	18	dwelling older adults				50.1-74.9															
		MET	71.2	30.1	18	0	18																				
		WP	73.1	28.1	18	0	18					50.1-74.9															
		RC	70.4	27.1	18	0	18																				

To be continued.

Table S2. Continued.

Study (year) [Reference No.][a]	Country (area)	Study arm	Age (years) Mean	BMI (kg/m²) Mean	Sex (n) Women	Sex (n) Men	Study sample (n)	Health status (medical condition)	Living status[d]	Protein supplementation: Frequency (day /week)	Protein supplementation: Intake amount (g/d or g/session)	Protein supplementation: Compliance (%)	Exercise intervention: Modality	Exercise intervention: Intensity (% 1-RM/RPE/HR_{max})	Exercise intervention: Frequency (session /week)	Exercise intervention: Duration (week)	Exercise intervention: Compliance (%)	Outcome measures[e] Muscle mass/volume: Whole body lean	Muscle mass/volume: ALM	Muscle mass/volume: Muscle volume	Strength: Handgrip strength	Strength: Leg strength	Mobility: Walk speed	Mobility: Chair rise	Mobility: TUG	Mobility: SPPB	Follow-up time point (week)
Bijeh 2022 [138]	Iran	SP+RET	65.6[c]	NR	0	15	15	Sedentary, healthy,	1	7	6.75	100	RET	60% 1-RM	3	12	100	V			V	V					0, 12
	(Asia)	RET			0	15	15	physically independent			(1.2~1.35 g・kg^{-1}・d^{-1})[f]						100										
		SP			0	15	15	older adults			6.75	100															
		RC			0	15	15				(1.2~1.35 g・kg^{-1}・d^{-1})[f]																
Bjorkman 2011 [140]	Finland	WP+RET	69.9	28.8	20	3	23	Older adults with	1	7	28	84.2	RET	Low intensity resistance	2	8	90	V	V		V	V	V	V			0, 8
	(Europe)	Casein+RET	69.1	27.1	22	2	24	substantial sarcopenia			13.2	84.4		for ergo training			55										
Bjorkman 2012 [139]	Finland	WP+AET	84.1	24.8	37	12	49	Nursing home residents at	2	7	20	84.2	AET (ergo ex)	Low intensity	2~3	24	90	V			V	V					0, 12, 24
	(Europe)	PLA+AET	83.0	24.0	43	14	57	frailty and sarcopenia risk				84.4					55										
Bjorkman 2020 [141]	Finland	MP+MET	83.6	25.3	51	22	73	Older people	1	7	40	58	MET (walking, chair stand, step),	Low intensity	7	52	45	V			V					V	0, 24, 52
	(Europe)	DP+MET	83.7	26.3	51	21	72	with sarcopenia			1.0~1.2 g・kg^{-1}・ d^{-1}	61[f]	home-based, 10~30 minutes/session,		(14		45										
		PLA+MET	84.0	26.8	46	27	73				7.5	64	2 session/day.		sessions)		45										
Bonnefoy 2003 [144]	France	SP+MET	83.0	27.4	12	3	15	Institutionalized	2	7	30	54~61[c]	MET (RET, BalaT)	Moderate intensity	3	36	63~70	V				V	V	V			0, 12, 36
	(Europe)	PLA+MET	83.0	27.5	13	0	13	frail elderly									63~70										
		SP	83.5	27.0	12	3	15																				
		PLA	83.5	27.1	13	1	14																				
Bonnefoy 2012 [143]	France	SP+MET	86.5	24.1	49	4	53	Pre-frail Community-	1	7	20	56	NR	NR	7	16	48	V					V	V	V		0, 16
	(Europe)	RC	85.1	25.8	39	10	49	dwelling older adults																			
Botella-Carretero 2008 [145]	Spain	SP+MET	83.1	24.2	27	3	30	Hospitalized elder patients	2	7	36 (1.03 g・kg^{-1}・ d^{-1})	41.1	MET(inpatient rehabilitation)	NR	7	2	NR			V							0, 2
	(Europe)	MP+MET	84.6	23.7	21	9	30	with hip fracture			37.6 (1.1 g・kg^{-1}・ d^{-1})	51.4															
		MET	83.7	23.6	23	7	30																				
Boutry-Regard 2020 [146]	Japan	ω3FA+WP+RET	76.0	22.5	8	2	10	Free-living elderly adults	1	7	20	97.4	RET (NMES), 20 min, co-contractions	Maximum intensity	2	12	NR	V		V		V	V				0, 12
	(Asia)	WP+RET	78.0	21.3	12	3	15	with limited mobility				96.8	in the upper & lower extremity	as tolerated													
		PLA+RET	78.0	20.8	10	2	12					97.2	muscle groups														
Buhl 2016 [147]	Denmark	MP+RET	73.3	27.0	12	2	14	Acutely ill	2	7	18.8	70.2~73.2	RET	8-12 RM	3	12	55	V	V		V			V			0, 12
	(Europe)	RC	72.4	30.4	7	8	15	old medical patients																			
Bunout 2004 [148]	Chile	SP+RET	74.0	27.3	21	10	31	Untrained,	1	7	15	48	RET	10-15 RM;	2	52	56	V		V	V	V	V				0, 24, 52, 72
	(America)	RET	74.4	27.1	12	4	16	medically stable,						Borg RPE: ≤8/10-point													
		SP	74.7	26.2	16	12	28	free-living elders						Light or very light													
		RC	73.7	28.9	17	16	33																				
Candow 2006 [149]	Canada	WP+RET	64.9	28.3	0	19	19	Untrained older adults	1	3	25 (0.3 g・kg^{-1}・ d^{-1})	NR	RET	70% 1-RM	3	12	NR	V		V		V					0, 12
	(America)	PLA+RET	64.6	29.1	0	10	10																				
Candow 2008 [150]	Canada	WP+Cre+RET	67.3	26.3	0	10	10	Untrained healthy	1	3	25 (0.3 g・kg^{-1}・ d^{-1})	94	RET	70% 1-RM	3	10	NR	V		V		V					0, 10
	(America)	Cre+RET	65.5	28.1	0	13	13	older adults				93															
		PLA+RET	64.1	26.1	0	12	12					95															
Cao 2007 [151]	Japan	DP+MET	63.5	23.2	40	0	40	Postmenopausal women	1	7	65	NR	20~30 RM	20~30 RM	4	36	NR	V			V		V	V			0, 36
	(Asia)	MET	63.9	23.4	48	0	48	with high fracture risk																			
		RC	68.0	23.0	38	0	38																				

To be continued.

Table S2. Continued.

Study (year) [Reference No.][a]	Country (area)	Study arm	Age (years)	BMI (kg/m^2)	Sex (n)		Study sample (n)	Health status (medical condition)	Living status[d]	Protein supplementation			Exercise intervention					Outcome measures[e]									Follow-up time point (week)
																		Muscle mass/volume			Strength		Mobility				
			Mean	Mean	Women	Men				Frequency (day /week)	Intake amount (g/d or g/session)	Compliance (%)	Modality	Intensity (% 1-RM/RPE/HR$_{max}$)	Frequency (session /week)	Duration (week)	Compliance (%)	Whole body lean	ALM	Muscle volume	Handgrip strength	Leg strength	Walk speed	Chair rise	TUG	SPPB	
Carlsson 2011 [152];	Sweden	MP+MET	85.0	24.9	36	10	46	ADL dependent,	2	2~3	7.4	82~84	MET (RET, BalaT, MobT),	13-15 RM	2~3	13	79	V				V	V	V			0, 13, 24
Rosendahl 2006 [319]	(Europe)	PLA+MET	85.5	24.8	31	14	45	institutionalized			0.2	79~80	supervised, 45 min,	(high intensity)	(29		72										
		MP	82.9	24.9	35	15	50	older adults				84	whole-body ex		sessions)												
		PLA	85.6	24.5	37	13	50					79															
Carroll 2024 [153]	USA	Beef+RET	67.0	31.0	9	0	9	Untrained, sedentary	1	7	1.4 g・kg^{-1}・ d^{-1}	91-93	RET	70% 1-RM	3	12	91			V		V					0, 12
	(America)	DP+RET	66.0	27.0	14	0	14	older adults			1.4 g・kg^{-1}・ d^{-1}	93-96					92										
		RET	65.0	27.0	11	0	11				0.8 g・kg^{-1}・ d^{-1}	96					93										
Centner 2019 [154]	Australia	Collagen+RET	61.7	27.0	0	11	11	Untrained, healthy,	1	7	15	NR	RET	20~30% 1-RM	3	8	NR			V		V					0, 8
	(Oceania)	PLA+RET	56.6	26.8	0	11	11	sedentary older adults						(low-intensity BFR)													
		RC	62.5	26.4	0	8	8																				
Chale 2013 [155]	USA	WP+RET	78.0	27.0	25	17	42	Mobility-limited,	1	7	40	72.1	RET	80% 1-RM	3	24	79.9	V		V		V	V	V		V	0, 24
	(America)	PLA+RET	77.3	26.9	22	16	38	older adults				82.3					80.2										
Chang 2019 [156]	China	Casein+RET	73.5	19.8	6	22	28	Sarcopenic elderly	1	7	20	NR	RET	70% 1-RM	3	24	NR				V					V	0, 24
	(Asia)	RC	76.5	19.1	6	22	28	people																			
Chatterjee 2018 [157]	India	DP+AET	75.7	NR	9	13	22	Prefrail older adults	1	7	1.2 g・kg^{-1}・ d^{-1}	100	AET	NR	3	12	100				V		V			V	0, 12
	(Asia)	WP+AET	77.4		4	18	22				8~10	100	(Nordic walking training)														
		WP	76.7		11	11	22				8~10	100															
Chen 2017 [160]	China	WP+RET	73.5	19.8	4	16	20	Elderly people	2	7	25.1	NR	RET	70% 1-RM	6	24	NR				V					V	0, 24
	(Asia)	DP	76.5	19.1	4	16	20	with sarcopenia			NR																
Chen 2021 [159];	Taiwan	WP+AET	58.6	27.3	22	1	23	Obese middle-aged and	1	7	0.4 g・kg^{-1}・ d^{-1}	93.75	AET, HIIT, 60 min	70%~90% Hrmax; RPE:	3	12	94	V	V			V		V			0, 12
Hsu 2021 [215]	(Asia)	AET	57.1	28.1	20	3	23	older adults						12~15/20 Borg			90										
		RC	59.0	27.6	21	2	23							15-point scale													
Chen 2024 [158]	China	Casein+MET	86.7	23.1	18	23	41	Nursing-home elderly	2	7	19.6	72.7	MET (AET, RET, BalaT)	Low (50~65 % HRmax) and	7	12	85.7	V	V		V		V			V	0, 6, 12
	(Asia)	DP+MET	82.9	23.7	28	30	58	resident with malnutrition			1.1 g・kg^{-1}・ d^{-1}	100		medium (65~75 % HRmax)			83.6										
Chin A Paw 2001 [161];	Netherlands	Casein+MET	78.9	25.0	31	11	42	Frail, Independently	1	7	40[f]	85	MET (AET, RET, MobT)	RPE 6-8/10	2	17	81.8	V			V	V	V	V			0, 17
de Jong 2000 [169]	(Europe)	PLA+MET	76.2	24.4	28	11	39	Living Elderly				32					81.8										
		Casein	79.2	24.5	28	11	39					85															
		PLA	78.6	24.1	25	12	37					32															
Colonetti 2023 [162]	Brazil	WP+RET	73.0	30.4	1	7	8	Institutionalized	2	3	30	NR	RET	70% 1-RM	3	24	NR	V	V								0, 6, 24
	(America)	PLA+RET	75.4	29.9	3	7	10	older adults																			
		RC	74.1	26.8	5	3	8																				
Corcoran 2017 [163]	USA	MP+MET	82.3	28.2	56	11	67	Mobility-limited older	2	3	20	87	MET (AET, RET, BalaT)	10 RM; RPE: "somewhat hard"	3	24	68				V		V			V	0, 12, 24
	(America)	RC	81.2	28.5	45	9	54	facility residents						to "moderately hard"			81										
Dalla Via 2021 [164]	Australia	WP+MET	71.4	28.4	0	34	34	Older adults with	1	7	25	77	MET (AET, RET, BalaT, MobT);	55– 80% HRmax for AET;	1~2	52	56	V	V	V	V	V	V	V	V		0, 24, 52
	(Oceania)	RC	71.1	29.2	0	36	36	prostate cancer					supervised, home-based sessions	RPE 4-6/10 for RET													
Daly 2014 [166]	Australia	Meat+RET	72.1	27.7	53	0	53	Sedentary older adults	2	7	45 (1.3 g・kg^{-1}・ d^{-1})	81	RET	RPE 14-16/20	2	16	75	V		V		V		V	V		0, 16
	(Oceania)	PLA (rice)+RET	73.6	27.6	47	0	47			7	≥6.8	100					72										

To be continued.

Table S2. Continued.

Study (year) [Reference No.][a]	Country (area)	Study arm	Age (years) Mean	BMI (kg/m²) Mean	Sex (n) Women	Sex (n) Men	Study sample (n)	Health status (medical condition)	Living status[d]	Protein supplementation: Frequency (day /week)	Protein supplementation: Intake amount (g/d or g/session)	Protein supplementation: Compliance (%)	Exercise intervention: Modality	Exercise intervention: Intensity (% 1-RM/RPE/HR_{max})	Exercise intervention: Frequency (session /week)	Exercise intervention: Duration (week)	Exercise intervention: Compliance (%)	Outcome measures[e] Muscle mass/volume: Whole body lean	Muscle mass/volume: ALM	Muscle mass/volume: Muscle volume	Strength: Handgrip strength	Strength: Leg strength	Mobility: Walk speed	Mobility: Chair rise	Mobility: TUG	Mobility: SPPB	Follow-up time point (week)
Daly 2020 [165]	Australia	MP+MET	55.0	29.3	123	0	123	Untrained, sedentary	1	7	18.2	90	MET (RET, BalaT, MobT, StreE)	10-15 RM	2	16	79	V	V	V	V	V	V	V	V		0, 16
	(Oceania)	PLA (rice)+MET	56.0	28.9	121	0	121	older adults			3.2	92					78										
de Azevedo Bach 2022 [167]	Brazil	WP+RET	66.9	26.3	NR	NR	16	Untrained, sedentary	1	7	30	97.2	RET	75-85% 1-RM (6-8 RM)	2	12	100	V	V			V		V	V		0, 12
	(America)	PLA+RET	65.8	25.4			15	older adults				97.1					99										
de Carvalho Bastone	Brazil	WP+RET	76.9	25.7	16	4	20	Older adults with	1	7	21	NR	RET	80% 1-RM	3	12	98.5	V			V		V	V	V		0, 12
2020 [168]	(America)	RET	77.6	26.0	12	8	20	dynapenia									97.2										
		WP	76.5	25.9	14	6	20																				
		RC	72.5	26.2	15	5	20																				
Deer 2019 [170]	USA	WP+RET	80.0	26.1	14	6	20	Hospitalized older adults	2	7	40	70.7	RET, home rehabilitation	NR (moderate intensity)	3	4	73.7	V	V				V	V		V	0, 4
	(America)	PLA+RET	77.6	27.4	14	7	21					79.9	training program				80.8										
		WP	80.0	28.9	14	6	20					70.7															
		PLA	75.7	29.0	14	6	20					79															
Deibert 2011 [171]	Germany	SP+RET	55.9	28.4	0	13	13	Untrained, sedentary	1	7	26.7	NR	RET	10 RM	2	12	90	V				V					0, 12
	(Europe)	RET	55.5	27.7	0	13	13	middle-aged and				NR					90										
		RC	55.8	27.2	0	9	9	older adults																			
Dirks 2017 [172];	Netherlands	MP+RET	78.0	28.7	20	11	31	Frail, untrained, sedentary	1	7	30	≥98	RET, supervised, whole-body ex	75% 1-RM (3~4 set x 8~10 rep)	4	24	84	V	V	V	V	V	V	V		V	0, 12, 24
Tieland 2012 [339]	(Europe)	PLA (CHO)+RET	79.0	28.2	21	10	31	elderly people							(96 sessions)												
Duff 2014 [173]	Canada	MP+RET	61.8	26.9	12	7	19	Untrained, medically stable	1	7	38	97	RET	8-12 RM	3	8	86	V		V		V					0, 8
	(America)	WP+RET	57.5	25.9	13	8	21	older adults				88					84										
Dulac 2021 [174]	Canada	WP+RET	68.3	26.7	0	21	21	Sedentary, independent	1	7	30	NR	RET	80% 1-RM	3	12	NR	V	V	V	V	V	V	V	V		0, 12
	(America)	Casein+RET	69.0	26.0	0	20	20	older adults						(RPE 8-10/10)													
		PLA+RET	70.7	25.4	0	19	19																				
Edholm 2017 [175];	Sweden	DP+RET	67.2	24.3	20	0	20	Untrained, independent,	1	7	$1.3\ g \cdot kg^{-1} \cdot d^{-1}$	NR	RET, SupV, LE	75-85% 1-RM	2	24	91	V	V			V		V	V		0, 24
Strandberg 2015 [329]	(Europe)	RET	67.9	24.6	17	0	17	recreationally active,									87										
		RC	67.5	24.8	18	0	18	older adults																			
Englund 2017 [177];	Sweden	WP+MET	78.1	27.9	34	40	74	Older adults with	1	7	20	88	MET (AET, RET, BalaT, StreE)	RPE (Borg): 13-14/20	3	24	75	V	V	V		V	V			V	0, 24
Fielding 2017 [181]	(Europe)	PLA+MET	76.9	28.4	35	40	75	mobility limitation				86		for AET; 15-17/20 for RET			72										
Evans 2007 [178]	USA	MP+AET	59.7	26.8	10	0	10	Relatively healthy or	1	7	25.6	NR	AET	Moderate intensity	3	36	NR	V									0, 36
	(America)	SP+AET	62.5	25.2	11	0	11	medically stable		7	25.6			(75% to 80% of VO_{2peak})													
		MP	62.8	27.7	12	0	12	postmenopausal women																			
		SP	63.5	25.9	10	0	10																				
Fernandes 2018 [179];	Brazil	WP+RET	67.3	25.9	16	0	16	Pre-conditioned,	1	3	35	NR	RET, 45~50 min, supervised,	8-12 RM	3	12	>85	V	V			V					0, 12
Sugihara Junior 2018 [333]	(Europe)	PLA (CHO)+RET	67.8	25.4	16	0	16	older adult					whole body training														
Fiatarone 1994 [180]	USA	SP+RET	87.2	24.5	16	9	25	Institutionalized,	2	7	40	99	RET	80% 1-RM	3	10	97	V		V		V	V				0, 10
	(America)	PLA+RET	86.2	24.9	16	9	25	medically stable				100					97										
		SP	85.7	25.4	17	7	24	elderly people				99					100										
		PLA	89.2	25.8	14	12	26					100					100										

To be continued.

Table S2. Continued.

Study (year) [Reference No.][a]	Country (area)	Study arm	Age (years) Mean	BMI (kg/m²) Mean	Sex (n) Women	Sex (n) Men	Study sample (n)	Health status (medical condition)	Living status[d]	Protein supplementation: Frequency (day /week)	Protein supplementation: Intake amount (g/d or g/session)	Protein supplementation: Compliance (%)	Exercise intervention: Modality	Exercise intervention: Intensity (% 1-RM/RPE/HR_{max})	Exercise intervention: Frequency (session /week)	Exercise intervention: Duration (week)	Exercise intervention: Compliance (%)	Outcome measures[e] Muscle mass/volume: Whole body lean	Muscle mass/volume: ALM	Muscle mass/volume: Muscle volume	Strength: Handgrip strength	Strength: Leg strength	Mobility: Walk speed	Mobility: Chair rise	Mobility: TUG	Mobility: SPPB	Follow-up time point (week)
Filho 2022 [182]	Brazil	WP+MET	71.7	30.4	18	0	18	Institutionalized pre-frail,	2	7	21	50.1-74.9	MET (RET, BalaT)	10-15 RM	2	12	NR			V	V	V	V	V	V		0, 12
	(Europe)	PLA+MET	69.7	29.4	18	0	18	medically stable				50.1-74.9															
		MET	71.2	29.6	18	0	18	older adults																			
		WP	73.1	28.4	18	0	18					50.1-74.9															
		RC	70.4	28.2	18	0	18																				
Flodin 2015 [183]	Sweden	MP+MET	81	22.7	19	7	26	Independent living,	1	7	40	69.4[f]	MET	NR		52	NR	V	V		V						0, 24, 52
	(Europe)	Vitamin-D+MET	78	22.4	19	6	25	elderly with hip fracture		7		69.4[f]	(post-surgery														
		Bisphosphonates+MET	80	24	18	10	28	medically stable		1		69.4[f]	conventional rehabilitation)														
Formica 2020 [184]	Australia	Meat+RET	71.2	27.8	48	29	77	Community-dwelling	1	3	45 (1.3 g・kg⁻¹・ d⁻¹)	NR	RET	RPE: 5-8/10 Borg	3	24	77.9	V	V	V		V	V	V	V		0, 24
	(Oceania)	RET	70.3	27.9	48	29	77	elderly									78.6										
Francis 2017 [185]	UK	MP+RET	60.4	24.7	29	0	29	Untrained free-living	1	7	24 (0.33 g・kg⁻¹・ d⁻¹)	86	RET	8-15 RM	3	12	NR	V	V			V	V	V			0, 12
	(Europe)	MP	61.8	26.1	28	0	28	older adults				82															
Franzke 2015a [186]; 2015b [187]	Austria	WP+RET	82.5	NR	84[c]	13[c]	29	Institutionalized elderly	2	7	20.7	NR	RET	15 RM	2	24	NR				V		V	V			0, 12, 24
	(Europe)	RET	82.8				35																				
		RC	83.5				33																				
Fujie 2025 [188]	Janan	Meat+RET	67.2	28.5	18	0	18	Postmenopausal elderly	1	3	22.5	≥90[c]	RET (weight-stack machine),	70% 1-RM	3	12	≥90[c]			V		V					0, 12
	(Asia)	RET	66.9	27.1	20	0	20	women					supervised; lower-body training	(moderate to high													
		Meat	67.0	27.6	22	0	22							-intensity;													
		RC	67.6	28.3	21	0	21							3 set x 10rep)													
Furtado 2024 [189]	Brazil	WP+RET	68.0	30.3	12	7	19	Older adults with	1	2	27	NR	RET,	70% 1-RM	2	12	NR	V	V	V	V	V					0, 12
	(Europe)	PLA (MP)+RET	66.6	30.7	12	8	20	type 2 DM			8.3		whole body training														
Gade 2019 [190]	Denmark	WP+RET	85.3	25.1	51	22	73	Hospitalized	2	7	27.5	63	RET (supervised inpatient training;	Low-intensity (8-12 RM)	4~7	12	37.2-51.4	V	V		V		V	V			0, 1, 12
	(Europe)	PLA (MP)+RET	84.2	25.8	49	26	75	older adults			1.5	57	home-based outpatient training)				32.4-50.8										
Gaffney 2018 [191]	UK	WP+MET	53.5	29.6	0	12	12	Older adults with	1	4~5	40	NR	MET (AET, RET)	70%–90% V̇O₂ maxium for AET;	4~5	10	NR			V		V					0, 10
	(Europe)	PLA(Oat)+MET	57.8	30.1	0	12	12	type 2 DM		4~5	10			25% 1-RM for RET													
Galbreath 2018 [192]	USA	DP+MET	65.5	31.3	17	0	17	Sedentary	1	7	1.2 g・kg⁻¹・ d⁻¹	95.8[f]	MET (AET, RET, StreE),	80% HRmax (65% V̇O₂ maxium)	3	14	≥ 70	V				V	V				0, 10, 14
	(America)	REHC+MET	63.3	30.3	18	0	18	overweight/obese					30 min, supervised circuit Ex	for AET; 61–82% 1-RM for RET													
		MET	66.0	29.9	19	0	19	older adults																			
Gao 2019 [193]	China	DP+RET	71.6	22.1	37[c]	39[c]	38	Hospitalized elderly	2	7	1.0~1.5 g・kg⁻¹・ d⁻¹	63.2	RET, home-based	10 RM	7	52	65.8	V			V		V	V	V		0, 52
	(Asia)	RC	72.3	21.9			38	with sarcopenia				44.7					42.1										
George 2017 [194]	USA	WP+MET	84.0	23.6	3	3	6	Community-dwelling	1	6	1.5 g・kg⁻¹・ d⁻¹	NR	MET (AET, RET),	Light exercise program	6	24	NR				V	V	V		V		0, 24
	(America)	RC	75.0	26.8	3	2	5	elderly with heart failure					20 min/session, home-based														
Grabovac 2018 [195]; Haider 2017 [201]; Kapan 2017 [226]	Austria	DP+RET	83.0	27.1	33	6	39	Malnourished frail	1	7	0.8 g・kg⁻¹・ d⁻¹	75	RET, supervised,	15 RM	2	24	75	V	V		V	V	V	V		V	0, 12, 24
	(Europe)	RC	82.5	27.6	34	7	41	elderly				58	whole-body ex, home-visit				58										
Granic 2020 [196]	UK	MP (whole)+RET	72.0	24.9	4	6	10	Community-dwelling,	1	2	17	97.1	RET, supervised	>70% 1-RM (8-10 RM); RPE:	2	6	97.2		V		V		V	V		V	0, 6
	(Europe)	MP (skimmed)+RET	72.2	25.5	4	6	10	medically stable older		2	18	98.3		"somewhat hard" to "hard"			98.3										
		PLA (CHO)+RET	70.8	27.0	4	6	10	adults with sarcopenia				95		(35~50/100 Borg CR scale)			95.8										

To be continued.

Table S2. Continued.

Study (year) [Reference No.][a]	Country (area)	Study arm	Age (years) Mean	BMI (kg/m²) Mean	Sex (n) Women	Sex (n) Men	Study sample (n)	Health status (medical condition)	Living status[d]	Protein supplementation: Frequency (day /week)	Protein supplementation: Intake amount (g/d or g/session)	Protein supplementation: Compliance (%)	Exercise intervention: Modality	Exercise intervention: Intensity (% 1-RM/RPE/HR_{max})	Exercise intervention: Frequency (session /week)	Exercise intervention: Duration (week)	Exercise intervention: Compliance (%)	Outcome measures[e] Muscle mass/volume: Whole body lean	Muscle mass/volume: ALM	Muscle mass/volume: Muscle volume	Strength: Handgrip strength	Strength: Leg strength	Mobility: Walk speed	Mobility: Chair rise	Mobility: TUG	Mobility: SPPB	Follow-up time point (week)
Griffen 2022 [197]	UK	WP+RET	68.0	26.6	0	9	9	Untrained, medically stable	1	7	50	96.1	RET, supervised,	80% 1-RM	2	12	98.2	V	V		V	V	V			V	0, 12
	(Europe)	PLA+RET	67.0	25.1	0	9	9	community-dwelling				96.1	whole-body ex				98.2										
		WP	66.0	25.0	0	9	9	older adults				96.8															
		PLA	67.0	25.1	0	9	9					94.1															
Gronstedt 2020 [198]	Sweden	MP+RET	85.8	25.3	34	18	52	Institutionalized,	2	7	36	64	RET	NR	7	12	44	V					V	V			0, 12
	(Europe)	RET	85.9	25.3	29	21	50	medically stable elderly									44										
Gryson 2014 [199]	France	WP+MET	60.9	26.8	0	8	8	Sedentary,	1	7	10	NR	MET (AET, RET),	80% HR_{max} for AET;	3	16	NR	V	V			V					0, 16
	(Europe)	Casein+MET	60.9	26.8	0	9	9	medically stable			10		45-60 min/session, supervised	80% 1-RM for RET													
		PLA+MET	60.9	26.5	0	9	9	older adults			4																
		WP	60.5	25.9	0	10	10				10																
		PLA	60.5	26.5	0	9	9				4																
Gusdon 2024 [200]	USA	WP+RET	60.2	NR	7	5	12	Hospitalized	2	7	81 (1.75 g・kg^{-1}・ d^{-1})	NR	RET	NR	7	12	NR			V							0, 12
	(America)	DP	55.6		9	3	12	older adults with CVA			1.2~1.4 g・kg^{-1}・ d^{-1}																
Haß 2022 [118]	Germany	WP+Omega-3+RET	70.4	27.8	11	10	21	Community-dwelling,	1	7	27	96	RET	NR	3	8	NR	V			V	V	V	V			0, 8
	(Europe)	WP+RET	71.5	28.2	11	9	20	medically stable				98															
		RET	69.9	26.9	10	10	20	older adults																			
Hamarsland 2019 [202]	Norway	MP+RET	74.3	NR	6	9	15	Untrained, medically	1	7	40	99	RET	6-12 RM	3	11	98.5	V	V	V		V		V			0, 11
	(Europe)	WP+RET	72.9		6	9	15	stable older adults				99					100										
Han 2021 [203]	Australia	DP+MET	82.4	25.1	58	28	86	Older adults with	1	7	30[f]	NR	MET(AET, RET, BalaT), 50 min, supervised,	NR	3	52	NR	V			V	V	V				0, 24, 52
	(Oceania)	RC	83.0	24.8	77	12	89	proximal femoral fracture					lower-body exercise														
Han 2024 [204]	China	WP+RET	74.2	NR	24	26	50	Older adults with	1	7	60	NR	RET,	8-12 RM	3	4	NR		V		V		V				0, 4
	(Asia)	RC	73.5		22	28	50	sarcopenia					supervised, 30~45 min														
Hankey 1993 [205]	Netherlands	MP+MET	81.0	NR	11[b]	3[b]	7	Hospitalized frail elderly	2	7	40[f]	NR	MET	NR	7	8	NR			V							0, 8
	(Europe)	MET	81.0				7	with malnutrion					(inpatient continuing care)														
Haub 2002 [207];	USA	Meat+RET	63.0	28.1	0	10	10	Community-dwelling	1	7	50 (0.6 g・kg^{-1}・ d^{-1})	NR	RET	80% 1-RM	2	12	94-100	V		V		V					0, 12
2005 [206]	(America)	SP+RET	67.0	28.3	0	11	11	older adults																			
He 2022 [208]	China	WP+RET	82.4	19.4	0	75	75	Older adults	1	7	25	NR	RET	NR	7	4	NR				V						0, 4
	(Asia)	RC	82.4	19.4	0	75	75	with sarcopenia																			
Hegerova 2015 [209]	Czech Republic	WP+MET	83.6	26.4	NR	NR	100	Hospitalized acutely	2	7	20	83.3	MET (AET, BalaT, MobT)	Low intensity (maximal 15-beat HR beats increase)	6	2	NR	V									0, 12, 24,
	(Europe)	MET	83.2	27.8			100	ill older patients					MET (BalaT, MobT)	NR	5												36, 52
Herda 2021 [119]	USA	WP+MET	62.3	25.0	28	18	46	Untrained healthy	1	7	40	> 70	MET (AET, RET),	8-12 RM	3	12	≥ 70	V			V	V	V		V		0, 12
	(America)	PLA+MET	61.8	24.8	39	16	55	community-dwelling elder				> 70	supervised, whole-body Ex														
Hofmann 2016 [210];	Austria	WP+RET	81.8	29.8	31	5	36	Institutionalized elderly	2	7	20.7~41.4	NR	RET, supervised, 60 min	RPE <7/10	2	24	71	V		V	V	V	V	V			0, 12, 24
Oesen 2015 [302];	(Europe)	RET	83.0	28.9	37	4	41	(retirement care facilities)						(15 RM)			71										
Strasser 2022 [330]		RC	83.4	28.9	35	5	40																				
Holm 2008 [211]	Denmark	WP+RET	55.0	24.0	13	0	13	Postmenopausal women	1	2-3	10	NR	RET	8-10 RM	2~3	24	81-91.7	V		V		V					0, 12, 24
	(Europe)	PLA+RET	55.0	27.0	16	0	16				(1.15 g・kg^{-1}・ d^{-1})[f]						81-91.7										

To be continued.

Table S2. Continued.

Study (year) [Reference No.][a]	Country (area)	Study arm	Age (years) Mean	BMI (kg/m²) Mean	Sex (n) Women	Sex (n) Men	Study sample (n)	Health status (medical condition)	Living status[d]	Protein supplementation: Frequency (day /week)	Protein supplementation: Intake amount (g/d or g/session)	Protein supplementation: Compliance (%)	Exercise intervention: Modality	Exercise intervention: Intensity (% 1-RM/RPE/HR_{max})	Exercise intervention: Frequency (session /week)	Exercise intervention: Duration (week)	Exercise intervention: Compliance (%)	Outcome measures[e] Muscle mass/volume: Whole body lean	ALM	Muscle volume	Strength: Handgrip strength	Leg strength	Mobility: Walk speed	Chair rise	TUG	SPPB	Follow-up time point (week)
Holwerda 2018 [212]	Netherlands	WP+RET	69.0	25.5	0	21	21	Untrained, medically stable	1	7	21	99.6	RET	80% 1-RM	3	12	95	V	V			V	V	V		V	0, 12
	(Europe)	PLA+RET	71.0	25.1	0	20	20	older adults				99.6															
Hotta 2021 [213]	Janan	WP+MET	75.3	21.5	5	5	10	Middle-aged & older adults	1	7	15	NR	MET (AET, RET, cardiac rehabilitation),	Low intensity	3	12	NR	V	V		V	V	V				0, 12
	(Asia)	MET	71.1	21.5	5	5	10	with chronic heart failure					supervised/home ex														
Hsieh 2019 [214]	Taiwan	MP+MET	71.6	24.4	27	50	77	Pre-frail or frail	1	7	25	NR	MET (AET, RET, BalaT),	NR (ACSM adapted)	3~7	24	NR				V	V	V				0, 4, 12, 24
	(Asia)	MET	72.0	25.1	33	46	79	older adults					home-based, 5~60 min,														
		MP	70.4	25.5	38	45	83						low-body ex														
		RC	72.5	25.1	29	51	80																				
Imaoka 2016 [216]	Janan	WP+MET	87.6	20.4	18	5	23	Institutionalized	2	7	4	NR	MET (AET, RET, BalaT, MobT)	Low intensity	2	12	NR	V			V						0, 12
	(Asia)	MET	82.6	20.5	16	6	22	frail elderly																			
		WP	84.6	20.4	20	3	23																				
		RC	82.5	20.6	15	8	23																				
Imaoka 2019 [217]	Janan	SP+AET	74.4	22.8	31	6	37	Independent, Community-	1	2	8.4	90	AET, 45 min, supervised	NR	1	12	NR	V			V		V				0, 12
	(Asia)	AET	76.3	23.6	30	7	37	dwelling elderly																			
Jadczak 2021 [218]	Australia	WP+MET	73.5	NR	24	10	34	Community dwelling	1	7	40	91.3	MET (AET, RET, BalaT), 30~60 min,	RPE: 12-14/20 for AET;	5	24	100	V	V		V		V		V	V	0, 12, 24
	(Oceania)	Rice-PS+MET	73.2		23	13	36	pre-frail or frail elder			40	100	level walking, whole-body ex	15-16/20 for RET			100										
Ji 2025a [220];	Korea	MP+MET	77.9	24.9	11	10	21	Community-dwelling	1	7	26	NR	MET (AET, RET), supervised,	50~70% VO_2 max for AET;	2	12	89.3	V			V		V			V	0, 12
2025b [219]	(Asia)	RC	78.2	26.6	11	10	21	elderly with sarcopenia					60 min, whole-body ex	40%~80% 1-RM for RET													
Jiang 2023 [221]	China	WP+MET	78.2	21.3	31	21	52	Community dwelling	1	7	36	91.6	MET (AET, RET), 40 min	Mod to high load	3	12	NR	V			V		V				0, 12
	(Asia)	RC	79.0	20.9	29	23	52	elderly with sarcopenia																			
Jin 2016 [222]	China	DP+AET	> 60[c]	NR	23[c]	27[c]	25	Hospitalized elderly	2	7	$1.2\ g \cdot kg^{-1} \cdot d^{-1}$	NR	AET, 30 min	70% $\dot{V}O_2$ peak	3	24	NR		V		V		V				0, 24
	(Asia)	RC					25	with sarcopenia																			
Jyvakorpi 2023 [223]	Finland	MP+RET	75.0	28.3	44	0	44	Older adults	1	7	23	92.1	RET, home-based, 60 min,	10-15 RM	7	12	81.8				V		V	V		V	0, 12
	(Europe)	RET	76.0	28.3	50	0	50	with sarcopenia					lower-body ex				70.7										
Kang 2019 [224]	China	WP+RET	76.8	21.0	41	25	66	Community-dwelling	1	7	32.4	NR	RET, supervised, 30 min,	NR	7	12	NR				V		V	V		V	0, 4, 8, 12
	(Asia)	RET	78.0	22.7	30	19	49	frail older people					home-based														
Kang 2020 [225]	Korea	MP+RET	61.2	23.7	41	19	60	Untrained, medically	1	7	40	>80	RET, 20 min	Light	7	12	NR	V	V	V	V	V				V	0, 12
	(Asia)	PLA+RET	58.4	23.6	46	14	60	stable older adults				>80															
Karelis 2015 [227]	Canada	WP+RET	69.9	24.9	26	8	34	Sedentary, medically	1	7	20	97	RET	80% 1-RM	3	19	90	V	V			V					0, 19
	(America)	Casein+RET	71.0	25.4	26	7	33	stable, non-frail elderly				96					91										
Kemmler 2016 [230]	Germany	WP+RET(NMES)	76.4	25.2	25	0	25	Community-dwelling	1	7	40 ($1.2\ g \cdot kg^{-1} \cdot d^{-1}$)	100	RET, supervised, 20 min,	RPE (Borg CR 10 scale):	1	26	88		V		V		V				0, 26
	(Europe)	RET(NMES)	77.3	25.0	25	0	25	older adults with					whole-body NMES	5-6/10 (ie, hard to hard+)			89										
		RC	77.4	24.7	25	0	25	sarcopenic obesity																			
Kemmler 2017 [231]	Germany	WP+RET(NMES)	77.1	26.1	0	33	33	Community-dwelling	1	7	80 ($1.7\text{~}1.8\ g \cdot kg^{-1} \cdot d^{-1}$)	91.2[f]	RET, supervised, 50 min,	RPE (Borg CR 10 scale):	1.5	16	91		V		V						0, 16
	(Europe)	WP	78.1	26.3	0	33	33	older adults with				87.5[f]	whole-body NMES	6-7/10 (ie, hard+	2 sessions												
		RC	76.9	26.1	0	34	34	sarcopenic obesity						to very hard)	per 2 wk												
Kemmler 2020a [228];	Germany	WP+RET	77.8	25.0	0	21	21	Untrained elderly	1	7	80 ($1.6\ g \cdot kg^{-1} \cdot d^{-1}$)	NR (high)	RET	80% 1-RM	2-3	52	95	V			V	V	V				0, 52, 72
2020b [229]	(Europe)	WP	79.2	24.5	0	22	22	with osteosarcopenia																			

To be continued.

Table S2. Continued.

Study (year) [Reference No.][a]	Country (area)	Study arm	Age (years)	BMI (kg/m²)	Sex (n)		Study sample (n)	Health status (medical condition)	Living status[d]	Protein supplementation			Exercise intervention					Outcome measures[e]									Follow-up time point (week)
																		Muscle mass/volume			Strength		Mobility				
			Mean	Mean	Women	Men				Frequency (day /week)	Intake amount (g/d or g/session)	Compliance (%)	Modality	Intensity (% 1-RM/RPE/HR_{max})	Frequency (session /week)	Duration (week)	Compliance (%)	Whole body lean	ALM	Muscle volume	Handgrip strength	Leg strength	Walk speed	Chair rise	TUG	SPPB	
Kim 2015 [232]	Janan	MFGM+MET	81.0	21.1	33	0	33	Community-dwelling frail, ,	1	7	21.5% per serve	NR	MET (RET, BalaT, MobT),	Moderate intensity;	2	12	NR		V		V	V	V		V		0, 12, 28
	(Asia)	PLA+MET	81.1	22.2	33	0	33	medically stable elderly		7	26.3% per serve		supervised, 60 min,	RPE (15-point Borg scale):													
		MFGM	81.0	22.1	32	0	32				(milk-based pill)		whole-body ex	12-14/20													
		PLA	80.3	22.9	32	0	32																				
Kirk 2019 [233];	UK	WP+MET	68.6	27.4	13	9	22	Sedentary, medically-	1	7	> 40	43.4	MET (RET, MobT),	≥12 RM for RET;	3	16	78.5	V			V	V	V			V	0, 16
2020 [234]	(Europe)	MET	66.6	28.1	12	12	24	stable, non-frail, untrained			(1.2~1.5 g・kg-1・ d-1)		supervised, 50 min,	RPE 7-9/10 (10-point Borg			77.1										
		WP	71.8	27.1	9	14	23	Community-dwelling				74.3	whole-body ex	scale) for MobT													
		RC	68.2	26.2	18	13	31	older adults																			
Koopmans 2024a [235];	Netherlands	Insect PS+AET	68.0	25.6	7	9	16	Active older adults	1	7	31	98	AET, walking	NR	7	12	NR	V			V	V					0, 12
2024b [236]	(Europe)	WP+AET	70.0	24.2	11	12	23				32	99															
		PLA+AET	68.0	23.9	11	9	20				3	100															
Korzepa 2025 [237]	UK	WP+RET	63.9	24.2	7	7	14	Untrained, medically-	1	7	29.0 (0.39 g・kg-1・ d-1)e	NR	RET, supervised	75% 1-RM	3	2	NR	V	V	V		V					0, 2
	(Europe)	SP(pea)+RET	58.9	24.9	7	6	13	stable older adults			28.7 (0.39 g・kg-1・ d-1)[f]		unilateral leg extend	(8 set x 10-12 rep)													
Krause 2019 [238]	Ireland	WP+RET	63.9	25.4	7	4	11	Untrained,	1	7	24 (0.33 g・kg-1・ d-1)	NR	RET	8-15 RM	3	12	NR	V			V		V	V			0, 12
	(Europe)	PLA+RET	63.9	25.0	5	5	10	medically-stable																			
		WP	62.3	27.2	3	4	7	older adults																			
		PLA	63.8	26.3	5	5	10																				
Kukuljan 2009a [239];	Australia	MP+RET	61.7	27.4	0	45	45	Medically-stable	1	7	13	92	RET, 60-75 min, supervised , whole-body ex	80-85% 1-RM	3	52	69	V		V		V	V				0, 12, 24, 36,
2009b [240]	(Oceania)	RET	60.7	28.1	0	46	46	older adults									65										52, 60, 72
		MP	61.7	27.7	0	45	45	with osteopenia				90															
		RC	59.9	26.7	0	44	44																				
Kuwaba 2023 [241]	Janan	Collagen+RET	51.9	22.8	0	10	10	Untrained, healthy middle-	1	7	9.2	NR	RET, bodyweight squats	NR	7	10	NR					V					0, 10
	(Asia)	PLA+RET	53.6	23.8	0	8	8	aged & older adult																			
Kwon 2015 [242]	Janan	DP+MET	76.5	NR	0	26	26	Community-dwelling	1	7	20-22	NR	MET (RET, MobT)	10 RM	1	12	NR				V		V				0, 12, 36
	(Asia)	MET	77.0	NR	0	25	25	prefrail older adults																			
		RC	76.9	NR	0	28	28																				
Lamb 2020 [243]	USA	SP(peanut)+RET	60.0	27.8	8	12	20	Untrained, medically-	1	7	35	NR	RET	RPE 7-9/10 (10-12 RM)	2	10	NR	V		V		V					0, 6, 10
	(America)	RET	58.0	29.7	9	10	19	stable older adults																			
Laviolette 2010 [244]	Canada	WP+MET	62.9	29.7	2	10	12	Sedentary older adults	1	7	20	NR	MET (AET, RET), 90 min,	80% HRmax for AET;	3	8	≥ 90			V		V					0, 8
	(America)	Casein+MET	67.6	26.7	6	4	10	with COPD		7	20		whole-body Ex	80% 1-RM for RET			≥ 90										
Leenders 2013 [245]	Netherlands	MP+RET	71.5	25.8	12	15	27	Untrained, medically-	1	7	15	NR	RET	75-80% 1-RM	3	24	90	V	V	V	V	V		V			0, 12, 24
	(Europe)	PLA+RET	69.5	25.9	12	14	26	stable elderly																			
Li DT 2021 [246]	China	DP+RET	71.6	NR	15	15	30	Community dwelling	1	7	1.2 g・kg-1・ d-1	NR	RET	30% 1-RM	7	24	NR		V		V		V				0, 24
	(Asia)	DP+AET	71.5		16	14	30	elderly with sarcopenia					AET, 40 min, treadmill walking	NR (low intensity)	7												
Li G 2025 [247]	Janan	DP+RET	64.0	24.4	0	7	7	Untrained, medically-	1	7	0.9~1.15 g・kg-1・ d-1f	NR	RET, home-based, 30 min,		7	2	NR	V	V	V	V				V		0, 2
	(Asia)	RET	64.0	24.4	0	7	7	stable older adults					lower-body Ex														
Li WL 2022 [248]	China	WP+MET	71.5	22.8	16	18	34	Community-dwelling	1	7	30	NR	MET (AET, RET), 90 min,	≥800 step/10 min for AET;	3	12	NR		V								0, 12
	(Asia)	WP	69.8	22.7	20	14	34	elderly with sarcopenia					home-based, UE/LE training,	8 RM for RET													
		RC	73.2	22.6	21	13	34						1-hour outdoor walking														

To be continued.

Table S2. Continued.

Study (year) [Reference No.][a]	Country (area)	Study arm	Age (years) Mean	BMI (kg/m²) Mean	Sex (n) Women	Sex (n) Men	Study sample (n)	Health status (medical condition)	Living status[d]	Protein supplementation: Frequency (day /week)	Protein supplementation: Intake amount (g/d or g/session)	Protein supplementation: Compliance (%)	Exercise intervention: Modality	Exercise intervention: Intensity (% 1-RM/RPE/HR_{max})	Exercise intervention: Frequency (session /week)	Exercise intervention: Duration (week)	Exercise intervention: Compliance (%)	Outcome measures[e] Muscle mass/volume: Whole body lean	Muscle mass/volume: ALM	Muscle mass/volume: Muscle volume	Strength: Handgrip strength	Strength: Leg strength	Mobility: Walk speed	Mobility: Chair rise	Mobility: TUG	Mobility: SPPB	Follow-up time point (week)
Li Y 2022 [249]	China	DP+MET	80.0	17.1	21	19	40	Community-dwelling	1	7	1.2~1.5 g・kg⁻¹・ d⁻¹	NR	MET (AET, RET, BalaT), 90 min,	80% 25~50 RM for RET	7	28	NR				V						0, 28
	(Asia)	MET	79.0	16.5	20	20	40	elderly with sarcopenia					whole-body Ex														
Li Z 2021 [250]	China	WP+MET	72.1	22.9	34	25	59	Community-dwelling	1	7	30	81.4	MET (AET, RET), 90 min,	≥800 step/10 min for AET;	3	12	81.4		V		V						0, 12
	(Asia)	MET	72.8	23.4	37	25	62	elderly with sarcopenia					home-based, UE/LE training,	8 RM for RET			59.7										
		WP	70.4	23.1	34	27	61					83.6	1-hour outdoor walking														
		RC	72.4	22.4	41	18	59										55.9										
Liang 2023 [252]	China	WP+RET	65.3	18.5	11	14	25	Untrained elderly	1	7	30	NR	RET, whole-body Ex, 60~90 min	RPE: moderate	3~4	8	NR						V				0, 8
	(Asia)	DP+MET	65.9	18.5	12	13	25	with sarcopenia		7	1.2~1.5 g・kg⁻¹・ d⁻¹	NR	MET (BalaT, MobT)	NR	7		NR										
Liang 2024 [251]	China	WP+RET	65.3	18.4	11	14	25	Patients with CVA	2	7	30	NR	RET, whole-body Ex, 60~90 min	RPE: moderate	3~4	8	NR						V				0, 8
	(Asia)	RC	65.8	18.5	12	13	25	and sarcopenia																			
Liao 2019 [255]	Taiwan	SP+MET	74.5	24.6	2	9	11	Older adults with CVA	1	3	17.5	NR	MET(AET, RET, MobT), 120 min,	70% HRmax for AET;	3	8	NR	V			V		V	V		V	0, 4, 8
	(Asia)	PLA+MET	71.0	24.0	3	8	11						post-stroke rehabilitation	RET: RPE 6-7/10 Borg sacle													
Liao 2021 [253]	Taiwan	WP+RET	68.6	28.1	36	0	36	Older adults with	1	2	14	100	RET	RPE 13-15/20	2	12	84.3	V	V		V		V				0, 12
	(Asia)	RET	69.8	27.4	36	0	36	knee osteoarthritis									82.6										
Liao 2022 [254]	China	WP+RET	68.4	19.7	0	30	30	Elderly with sarcopenia	1	7	25	NR	RET, 60 min	20-30 RM	7	24	NR		V		V					V	0, 24
	(Asia)	DP+AET	68.4	19.7	0	30	30			7	1.2 g・kg⁻¹・ d⁻¹	NR	AET	NR	7												
Liu 2025 [256]	China	Casein+MET	56.3	25.1	27	5	32	Older adults with	1	7	480 mg/d	NR	MET(AET, RET, BalaT), 45~60 min,	10 RM	2	24	70.6					V	V	V			0, 12, 24
	(Asia)	PLA+MET	56.6	24.9	29	4	33	knee osteoarthritis					supervised, whole-body Ex				68.5										
Llaneza 2011 [257]	Spain	SP+AET	57.3	34.7	43	0	43	Obese postmenopausal	1	7	200 mg/d	NR	AET (1-hour walking)	NR	7	24	76.7	V									0, 24
	(Europe)	AET	56.1	35.2	44	0	44	women									84.1										
Long 2021 [258]	China	DP+RET	75.3	21.5	23	37	60	Elderly with sarcopenia	1	7	NR	NR	RET, UE/LE training	10~15 RM	7	24	NR		V		V		V				0, 12, 24
	(Asia)	RC	76.2	21.0	21	39	60																				
Ma 2023 [259]	China	DP+RET	69.1	NR	29	20	49	Elderly with DM	2	7	0.8 g・kg⁻¹・ d⁻¹	NR	RET, inpatient supervised,	75~85% 1-RM	3~5	12	NR			V	V				V		0, 12
	(Asia)	RC	70.8		28	24	52	and sarcopenia					outpatient home-based														
Macpherson 2022 [260]	Australia	WP+MET	70.0	27.0	51	22	73	Sedentary older adults	1	7	20	83.3	MET(AET, RET), 45~60 min,	RPE: 5-8/10 Borg scale	2	24	76.9	V	V		V		V	V	V		0, 24
	(Oceania)	PLA (MP)+MET	70.5	26.4	52	22	74	at risk of dementia		7	4	90	supervised, whole-body Ex	for both AET & RET			76										
Maesta 2007 [261]	Brazil	SP+RET	57.6	27.8	14	0	14	Untrained,	1	NR	25	NR	RET, supervised, 40-50 min,	60-80% 1-RM	3	16	NR	V	V								0, 16
	(America)	PLA (MP)+RET	60.7	27.7	11	0	11	medically-stable,			12.2		whole-body Ex														
		SP	61.3	27.2	10	0	10	postmenopausal women																			
		PLA (MP)	57.9	26.6	11	0	11																				
Magrans-Courtney 2011 [262]	USA	DP+RET	53.8	33.3	14	0	14	Untrained older adults	1	7	55~63% protein of	NR	RET, supervised,	60~80% HRmax	3	14	≥70	V									0, 10, 14
	(America)	RET	54.8	33.2	16	0	16	with knee osteoarthritis			daily diet		whole-body Ex														
Malafarina 2017 [263]	Spain	MP+MET	85.7	24.9	33	16	49	Older adults with	2	7	40	>80	MET (RET, BalaT, MobT),	NR	5	6	NR	V	V		V		V				0, 6
	(Europe)	DP+MET	84.7	26.0	35	8	43	hip fracture and sarcopenia		7	23.3% protein (87.4 g/day)	NR	50 min, rehabilitation														
Maltais 2016 [264]	Canada	MP+RET	68.0	25.8	0	10	10	Sarcopenic elderly men	1	3	13.5	>90	RET, 60 min	80% 1-RM	3	16	≥ 90	V	V			V	V	V	V		0, 16
	(America)	SP+RET	64.0	27.0	0	10	10				12	>90		(6~8 RM)			≥ 90										
		PLA (rice milk)+RET	64.0	25.9	0	10	10				0.6	>90					≥ 90										

To be continued.

Table S2. Continued.

Study (year) [Reference No.][a]	Country (area)	Study arm	Age (years)	BMI (kg/m^2)	Sex (n)		Study sample (n)	Health status (medical condition)	Living status[d]	Protein supplementation			Exercise intervention					Outcome measures[e]									Follow-up time point (week)
																		Muscle mass/volume			Strength		Mobility				
			Mean	Mean	Women	Men				Frequency (day /week)	Intake amount (g/d or g/session)	Compliance (%)	Modality	Intensity (% 1-RM/RPE/HR$_{max}$)	Frequency (session /week)	Duration (week)	Compliance (%)	Whole body lean	ALM	Muscle volume	Handgrip strength	Leg strength	Walk speed	Chair rise	TUG	SPPB	
Matsuda 2022 [265]	Janan	SP+MET	73.0	23.1	5	10	15	Medically-stable elderly	1	7	7.5	NR	MET(AET, RET), lower-limb Ex	10 RM	3	24	NR		V		V	V					0, 8, 16, 24
	(Asia)	PLA (BCAA)+MET	73.0	24.3	8	13	21	with type 2 DM			4																
McKenna 2021 [266]	USA	Meat+RET	49.0	27.6	14	14	28	Overweight middle-	1	7	30	97	RET	75% 1-RM	3	10	89	V	V		V	V	V				0, 10
	(America)	PLA+RET	50.0	27.5	13	9	22	aged and older adults				92					87										
Memelink 2021 [267]	Netherlands	WP+MET	66.8	32.8	20	42	62	Older adults	1	7	42	90	MET (AET, RET), supervised,	AET: 85~95% Hrmax;	3	13	82	V	V			V	V	V			0, 13
	(Europe)	PLA+MET	65.8	33.5	23	38	61	with obesity and DM				97	60 min, UE/LE ex	RET: 80% 1-RM			89										
Meredith 1992 [268]	USA	MP+RET	67.8	24.8	0	6	6	Sedentary older adults	1	7	19.9 (1.5 g・kg^{-1}・ d^{-1})	NR	RET, 20~30 min, supervised,	80% 1-RM	3	12	NR	V		V		V					0, 6, 12
	(America)	RET	64.8	25.4	0	5	5						lower-limb Ex														
Mertz 2021 [269]	Denmark	WP+RET(high load)	70.3	25.9	18	18	36	Community-dwelling	1	7	40	88	RET, 60 min, supervised, UE/LE ex	6~12 RM	3	52	72	V		V		V	V				0, 52
	(Europe)	WP+RET(low load)	70.4	25.7	18	18	36	older adults				88	RET, 60 min, home-based, UE/LE ex	NR	3~5		89										
		WP	70.3	25.2	22	28	50					88															
		Collagen	70.4	25.4	23	27	50			7	40	96															
		PLA(CHO)	69.6	26.0	18	18	36					95															
Midttun 2024 [270]	Denmark	WP+RET	77.4[f]	26.7[f]	0	72	72	Frail elderly with	1	3	34	100	RET, supervised, group training	10-12 RM	3	16	77.3~85.1[f]	V	V					V	V		0, 16
	(Europe)	RC	76.9[f]	27.2[f]	0	76	76	high fall risk						(2-3 set x 10-15 rep)													
Miller EG 2021 [271]	Australia	WP+RET	61.1	29.6	36	62	98	Older adults with obesity	1	7	40	78.6	RET	RPE 12-15/20	3	24	67.5	V	V	V		V		V	V		0, 12, 24
	(Oceania)	PLA+RET	62.0	31.1	36	64	100	and type 2 DM				91.5					57.9										
Miller GD 2006 [273]; 2012 [272]	USA	MP+MET	69.7	34.9	28	16	44	Older obese patients with	1	7	18~36	75	MET(AET, RET, StreE), facility-based,	AET: 50~85% HRmax;	3	24	77.5	V					V				0, 24
	(America)	RC	69.3	34.3	26	17	43	symptomatic knee OA					supervised, 60 min	RET: 12 RM (2 set x 12 rep)													
Miller MD 2005 [275]; 2006 [274]	Australia	MP+RET	82.7	23.2	17	7	24	Hospitalized and	2	7	35~50[f]	66	RET, 20~30 min, supervised,	8 RM	3	12	≥ 86			V		V	V				0, 12
	(Oceania)	RET	84.8	21.4	20	5	25	malnourished elder adults					lower-limb Ex				≥ 86										
		MP	83.5	21.9	21	4	25	with lower limb fracture				76															
		RC	83.1	22.1	21	5	26																				
Mitchell 2018 [276]	New Zealand	MP+RET	51.5	27.5	0	15	15	Immobilized middle-aged	1	3	20	NR	RET	80% 1-RM	3	2	NR		V	V		V					0, 2
	(Oceania)	PLA+RET	48.5	28.3	0	15	15	and older adults																			
Mogelberg 2022 [277]	USA	Collagen+MET	68.3	22.2	4	3	7	Older adults with COPD	1	7	≥ 25% TEE/d	66	MET(AET, RET), group-based,	NR	2	12	75	V		V	V		V				0, 12
	(America)	MET	71.7	21.0	3	0	3						supervised, 60 min				81										
Mojtahedi 2011 [278]	USA	WP+AET	64.7	32.3	13	0	13	Over weight/obese	1	7	45	81.4	AET, 20~30 min, home-based walk	RPE: 10-15/20	2	24	87.9	V	V	V		V					0, 24
	(America)	PLA+AET	64.6	32.7	13	0	13	postmenopausal women				71.3		(15-pont Borg sacle)			78.6										
Molnar 2016 [279]	Hungary	WP+RET	66.6	22.6	10	7	17	Institutionalized elderly	2	7	40	NR	RET, 30 min, supervised,	NR	2	12	NR	V			V					V	0, 12
	(Europe)	RET	66.4	28.1	12	5	17	With sarcopenia risk					whole-body ex														
Mori 2014 [281]	Janan	WP+MET	66.1	22.8	9	3	12	Untrained Community-	1	2	25	91.7	MET(AET, RET), 90 min,	AET: 50~60% HRmax;	2	9	91.7	V			V	V	V		V		0, 9
	(Asia)	PLA+MET	66.3	22.4	9	3	12	dwelling elderly				92.6	supervised	RET: 8~12 RM			92.6										
Mori 2018 [282]	Janan	WP+RET	70.6	22.1	25	0	25	Untrained Community-	1	2	11	90.1	RET	50-70% 1-RM	2	24	90.1	V	V		V	V	V				0, 24
	(Asia)	RET	70.6	22.9	25	0	25	dwelling elderly									86.6										
		WP	70.6	22.3	25	0	25			7	11	86.5															

To be continued.

Table S2. Continued.

Study (year) [Reference No.][a]	Country (area)	Study arm	Age (years) Mean	BMI (kg/m^2) Mean	Sex (n) Women	Sex (n) Men	Study sample (n)	Health status (medical condition)	Living status[d]	Protein supplementation: Frequency (day /week)	Protein supplementation: Intake amount (g/d or g/session)	Protein supplementation: Compliance (%)	Exercise intervention: Modality	Exercise intervention: Intensity (% 1-RM/RPE/HR_{max})	Exercise intervention: Frequency (session /week)	Exercise intervention: Duration (week)	Exercise intervention: Compliance (%)	Outcome measures[e] Muscle mass/volume: Whole body lean	Muscle mass/volume: ALM	Muscle mass/volume: Muscle volume	Strength: Handgrip strength	Strength: Leg strength	Mobility: Walk speed	Mobility: Chair rise	Mobility: TUG	Mobility: SPPB	Follow-up time point (week)
Mori 2020 [280]	Janan	WP+RET	77.7	20.6	13	2	15	Older adults with	1	2	11	83.3	RET, supervised	Low to moderate (10~15 RM);	2	24	83.3	V			V	V	V				0, 24
	(Asia)	RET	77.7	20.8	13	2	15	sarcopenia						RPE: easy to slightly hard			83.3										
		WP	77.2	20.2	13	2	15			2	11	83.3															
Mori 2021 [283]	Janan	WP+RET	78.1	20.3	20	0	20	Older adults with	1	2	11	74.6	RET, supervised, 60~90 min,	RPE: easy to slightly hard	2	24	74.6	V			V	V	V				0, 24
	(Asia)	RET	78.1	19.6	19	0	19	sarcopenia					whole-body ex				73.4										
		WP	77.0	19.6	20	0	20			2	11	75.6															
Mori 2022 [284]	Janan	WP+RET	77.7	20.3	20	3	23	Older adults with	1	2	11	73.2	RET	50-70% 1-RM	2	24	73.2		V		V	V	V				0, 24, 36, 48
	(Asia)	RET	77.6	20.3	19	4	23	sarcopenia									72.4										
		WP	77.8	20.1	20	4	24			7	11	73															
Morikawa 2018 [285]	Janan	SP+AET	64.0	21.9	14	0	14	Medically-stable older	1	7	9.6	94.2	AET, 15 min, supervised,	≥ 70% $\dot{V}O_2$ peak	4	20	75			V		V					0, 20
	(Asia)	PLA+AET	65.0	21.4	16	0	16	postmenopausal women		7	0.2	90.9	interval walking training				57.5										
Moyama 2023 [286]	Janan	DP+RET	79.0	22.3	21	23	44	Hospitalized elderly	2	7	$1.5\ g \cdot kg^{-1} \cdot d^{-1}$	NR	RET, UE/LE ex, unsupervised	Light	3	24	NR	V			V						0, 12, 24
	(Asia)	RET	79.0	21.3	21	28	49	with nutritional risk																			
Munk 2021 [287]	Denmark	DP+RET	75.0	22.0	61	32	93	Acute-stay discharged	1	7	65~85 g/d	58	RET, chair-stand strength training,	NR	7	16	NR							V			0, 16
	(Europe)	RC	76.0	22.0	66	32	98	older patients				18	home-based														
Murphy 2016 [38]	Canada	DP+RET	73.0	26.7	0	10	10	Free-living older	1	7	$> 1.2\ g \cdot kg^{-1} \cdot d^{-1}$	95-98	RET, supervised,	40~50% 1-RM	2	1	NR	V									0, 1
	(America)	PLA+RET	72.0	28.2	0	10	10	individuals			$0.8\ g \cdot kg^{-1} \cdot d^{-1}$	98	lower-body ex	(3 set x 15~24 rep)													
Nabuco 2018 [291];	Brazil	WP+RET	66.9	25.9	47	0	47	Pre-conditioned	1	3	35	NR	RET, supervised, whole-body ex	NR (8-12 RM)	3	12	NR	V	V			V	V	V			0, 12
2019a [292]	(America)	PLA+RET	66.5	23.8	23	0	23	older adults			0.3																
Nabuco 2019b [289]	Brazil	WP+RET	68.0	26.4	13	0	13	Older adults	1	3	35	NR	RET, supervised, whole-body ex	NR (12 RM)	3	12	NR	V	V								0, 12
	(America)	PLA+RET	70.1	27.4	13	0	13	with sarcopenic obesity																			
Nabuco 2019c [290]	Brazil	WP+RET	69.2	27.4	15	0	15	Physically independent	1	3	35	NR	RET, supervised, whole-body ex	60-70% 1-RM	3	12	NR	V				V					0, 12
	(America)	PLA+RET	68.4	26.6	15	0	15	older adults						(12 RM)													
Nakagawa 2024 [293]	Janan	WP+RET	71.6[f]	24.3	9	22	31	Older stroke patients	1	3	10	100	RET, spervised	NR	3	12	100	V	V		V	V	V	V			0, 12
	(Asia)	RET	71.1[f]	23.8	12	17	29										100										
Nakayama 2021 [294]	Janan	MP+RET	71.4	23.1	45	16	61	Untrained, physically	1	7	10	96.4	RET, supervised/unsupervised,	< 70% (12 RM, low to	7	24	92.6	V			V	V	V	V	V		0, 24
	(Asia)	PLA+RET	70.4	22.8	47	14	61	independent older adults			0.1	97.3		moderate intensity)			92										
Nambi 2025 [295]	Saudi Arabia	DP+MET	68.1	23.2	0	38	38	Post COVID- 19 elderly	1	7	$1.0\sim1.3\ g \cdot kg^{-1} \cdot d^{-1}$	90[f]	MET(AET, RET), supervised	AET: 40~50 HR_{max};	7	8	NR			V	V						0, 4, 8, 24
	(Asia)	PLA+MET	68.5	23.4	0	38	38	with sarcopenia			$0.8\sim1.0\ g \cdot kg^{-1} \cdot d^{-1}$	100[f]		RET: 50~100% 10-RM													
Ng 2015 [296]	Singapore	MP+MET	70.4	24.4	23	26	49	Prefrail & frail,	1	7	26.3	88	MET(RET, BalaT, MobT),	8~15 RM	7	24	88					V	V				0, 12, 24, 52
	(Asia)	MET	70.3	23.5	21	27	48	independent walk,					home-based ex	(60~80% 10-RM)			85										
		MP	69.7	24.0	17	32	49	free-living, older adults				91															
		PLA	70.1	23.6	22	28	50	(community residents)				94															
		RC	69.7	23.1	12	38	50																				
Ni 2019 [297]	China	WP+MET	69.3	20.2	4	26	30	Hospitalized prefrail	2	7	25.1	NR	MET(AET, RET, BalaT),	AET: RPE slightly hard to hard;	2~3	24	NR				V					V	0, 4, 12, 24
	(Asia)	RC	68.9	20.2	6	24	30	older adults					supervised, 80 min	RET: 8 RM													
Niccoli 2017 [298]	Canada	WP+MET	81.8	24.2	15	7	22	Hospitalized geriatric	2	7	24	78	MET(AET, RET, BalaT, MobT),	NR (ligh weight)	7	4	NR				V	V	V		V		0, 4
	(America)	MP+MET	80.3	26.4	17	8	25	patients		7	NR	78	whole-body ex														

To be continued.

Table S2. Continued.

Study (year) [Reference No.][a]	Country (area)	Study arm	Age (years) Mean	BMI (kg/m^2) Mean	Sex (n) Women	Sex (n) Men	Study sample (n)	Health status (medical condition)	Living status[d]	Protein supplementation: Frequency (day /week)	Protein supplementation: Intake amount (g/d or g/session)	Protein supplementation: Compliance (%)	Exercise intervention: Modality	Exercise intervention: Intensity (% 1-RM/RPE/HR_{max})	Exercise intervention: Frequency (session /week)	Exercise intervention: Duration (week)	Exercise intervention: Compliance (%)	Outcome measures[e] Muscle mass/volume: Whole body lean	Muscle mass/volume: ALM	Muscle mass/volume: Muscle volume	Strength: Handgrip strength	Strength: Leg strength	Mobility: Walk speed	Mobility: Chair rise	Mobility: TUG	Mobility: SPPB	Follow-up time point (week)
Niitsu 2016 [299]	Janan	WP+MET	80.5	19.3	20	0	20	Older inpatients	2	7	32.2	NR (80)	MET (RET, MobT), inpatient PT	50~100 RM for STS;	7	8	NR (100)					V					0, 8
	(Asia)	MET	78.8	20.1	18	0	18	with hip fracture					(STS/gait training)	300 m for gait ex			NR (94.4)										
Nilsson 2020 [300]	Canada	MP+RET	77.4	29.3	0	16	16	Overweight/obese elderly	1	7	24	89.3	RET	NR (10-15 RM)	3	12	84.1	V	V		V	V	V	V	V	V	0, 12
	(America)	PLA+RET	74.4	27.4	0	16	16	with sarcopenia				95.4					89.1										
Ninomiya 2023 [301]	Janan	WP+RET	70.3	22.2	29	0	29	Prefrail/frail elder patients	1	7	22	81.2	RET, unsupervised, home-based	low intensity; RPE: 11-13/20	7	12	84.6			V	V	V			V		0, 12
	(Asia)	RET	70.9	23.1	29	0	29	with total hip arthroplasty						(15-pont Borg sacle)			82.7										
Oh 2022 [303]	Korea	MP+RET	50~70[c]	23.3	NR	NR	21	Untrained, sedentary,	1	7	40	NR	RET, 60 min, supervised,	RPE: 7~8/10 Borg	3	12	NR	V	V		V			V	V		0, 12
	(Asia)	PLA+RET		24.0			20	older adults					whole-body ex														
Oikawa 2018 [304]	Canada	WP+AET	69.0	31.2	8	8	16	Untrained, physically	1	7	30 (1.6 g・kg^{-1}・ d^{-1})	100[f]	AET (walking)	NR	7	1	100[f]	V	V								0, 1
	(America)	Collagen+AET	68.0	28.0	7	8	15	independent, elderly		7	30 (1.6 g・kg^{-1}・ d^{-1})	100[f]					100[f]										
Orsatti 2018 [305]	Brazil	SP+RET	56.8	27.5	16	0	16	Medically-stable	1	7	31.4	NR	RET, supervised, whole-body ex	60-80% 1-RM	3	16	100	V				V					0, 16
	(America)	PLA (MP)+RET	58.8	27.3	16	0	16	postmenopausal women			6.4			(8-12 RM)			100										
Osuka 2017 [306]	Janan	MP+RET	70.6	22.9	18	10	28	Untrained, medically-	1	7	10.5	95.1	RET, 65~80 min, supervised, whole-body ex	RET: ≥ 70% 1-RM;	2	12	88.8	V	V			V		V			0, 12
	(Asia)	MP+MET	69.6	22.7	20	8	28	stable elderly				95.4	MET (AET, RET), 65~80 min, supervised	AET: 40%-50% V̇O2 peak			91.4										
Pan 2022 [307]	China	DP+RET	71.6	24.9	12	22	34	Elderly with	1	7	1.5 g・kg^{-1}・ d^{-1}	NR	RET, supervised/unsupervised	Moderate Borg RPE	3	12	NR		V	V	V		V	V	V	V	0, 12
	(Asia)	RC	72.2	25.0	11	19	30	probable sarcopenia			(50% protein of daily diet)		home-based,														
Park 2023 [308]	Korea	DP+MET	79.7	24.9	14	0	14	Community-dwelling	1	7	20~40 (> 0.8 g・kg^{-1}・ d^{-1})	91	MET (AET, RET), 45 min, supervised,	RPE (10-point Borg): 5-6/10	3	8	97				V		V			V	0, 8
	(Asia)	DP+AET	82.9	25.9	15	0	15	prefrail older women				97	AET(stepping), supervised, 45 min	50~60% Hrmax			97										
		DP	82.0	24.7	13	0	13					95															
		RC	81.5	25.4	14	0	14																				
Pedersen LR 2019 [309]	Denmark	DP+AET	63.8	31.3	8	21	29	Overweight older patients	1	7	1.2~1.5 g・kg^{-1}・ d^{-1}		AET(HIIT), supervised	RPE: 17-18/20 (15-pont Borg)	2~3	12		V									0, 12, 52
	(Europe)	AET	62.3	31.5	4	22	26	with CAD			(25% TEE)			70~90% Hrmax													
Pedersen MM 2019 [310]	Denmark	MP+RET	82.1	25.3	30	12	42	Hospitalized older patients	2	3	18	100	RET, supervised, 60 min,	60-70% 1-RM	3	5	83				V	V	V	V			0, 4, 24
	(Europe)	RC	82.5	24.5	26	17	43						lower-body ex (ankle weights)	(8-12 RM; 3 set x 8-12 rep)													
Peng 2024 [311]	Taiwan	Meat+RET	65.1	22.9	36	11	47	Older age with	1	3	24-30	NR	MET (AET, RET), 60 min,	Moderate	1	12	NR		V		V		V	V			0, 4, 12
	(Asia)	PLA+RET	64.2	22.7	43	7	50	inadequate protein intake					supervised, whole-body ex														
Puente-Fernandez 2025 [312]	UK	WP+RET	52.0	26.5	5	5	10	Older physically	1	3	26	NR	RET, supervised, 40~60 min,	70-75% 1-RM	3	6	NR	V		V		V					
	(Europe)	PLA+RET	51.0	28.1	5	5	10	active individuals					whole-limb Ex	(10-12 RM)													
Rabadi 2008 [313]	USA	MP+MET	73.6	23.9[f]	23	35	58	Hospitalized, elder	2	7	33	NR	MET	NR	7	5	NR						V				0, 5
	(America)	PLA+MET	75.0	23.8[f]	25	33	58	patients with CVA					(inpatient rehabilitation)														
Reidy 2017 [315];	USA	WP+RET	70.0	25.7	2	8	10	Physically active	1	7	64.5	NR	RET, NMES, 40 min, supervised,	Maximal intensity	7 (12	1	NR	V	V	V		V	V		V		0, 1
2020 [314]	(America)	PLA	69.0	25.3	1	9	10	older adults					lower-limb Ex, isometric contraction ex	as tolerated	sessions)												
Romera-Liebana 2018 [316]	Spain	MP+MET	77.2	28.9	134	42	176	Community-dwelling frail	1	7	11.8	NR	MET (RET, MobT, BalaT, StreE), 60 min,	NR	2	6	54				V					V	0, 12, 72
	(Europe)	RC	77.4	30.5	131	45	176	elderly individuals					supervised, whole-body ex				48										
Rondanelli 2016 [318]	Italy	WP+MET	80.8	23.9	40	29	69	Elderly people	1	7	22	NR	MET (RET, MobT, BalaT), 20 min,	RPE (15-point Borg scale):	7	12	NR	V	V		V						0, 12
	(Europe)	PLA (CHO)+MET	80.2	23.9	37	24	61	with sarcopenia					supervised, whole-body ex	12-14/20													

To be continued.

Table S2. Continued.

Study (year) [Reference No.][a]	Country (area)	Study arm	Age (years) Mean	BMI (kg/m²) Mean	Sex (n) Women	Sex (n) Men	Study sample (n)	Health status (medical condition)	Living status[d]	Protein supplementation: Frequency (day /week)	Protein supplementation: Intake amount (g/d or g/session)	Protein supplementation: Compliance (%)	Exercise intervention: Modality	Exercise intervention: Intensity (% 1-RM/RPE/HR_{max})	Exercise intervention: Frequency (session /week)	Exercise intervention: Duration (week)	Exercise intervention: Compliance (%)	Outcome measures[e] Muscle mass/volume: Whole body lean	Muscle mass/volume: ALM	Muscle mass/volume: Muscle volume	Strength: Handgrip strength	Strength: Leg strength	Mobility: Walk speed	Mobility: Chair rise	Mobility: TUG	Mobility: SPPB	Follow-up time point (week)
Rondanelli 2020 [317]	Italy	WP+MET	80.0	21.1	41	29	70	Hospitaliized elderly	2	7	40	92	MET (RET, MobT, BalaT), 20~30 min,	Moderate intensity;	5	8	NR	V	V		V			V	V	V	0, 8
	(Europe)	PLA (CHO)+MET	81.0	22.1	47	23	70	with sarcopenia				90	supervised, whole-body ex	RPE (15-point Borg): 12-14/20													
Roschel 2021 [120]	Brazil	WP+RET	72.5	26.7	22	23	45	Frail edlerly	1	7	30	NR	RET, 30~60 min, supervised,	70	2	16	NR	V	V	V	V	V		V	V		0, 16
	(America)	SP+RET	72.0	28.6	22	0	22						whole-body ex														
		PLA (CHO)+RET	72.5	28.8	22	23	45																				
Rydwik 2008 [320]	Sweden	DP+MET	83.1	21.9	16	9	25	Frail, independent walk,	1	7	NR	73	MET (AET, RET, MobT, BalaT),	80% HRmax for AET;	2	12	65	V				V	V	V	V		0, 12, 36
	(Europe)	MET	83.5	21.9	11	12	23	medically-stable elderly					supervised	80% 1-RM for RET;			65										
		DP	83.1	21.8	15	10	25					73		RPE 3~4/10 (Borg)													
		RC	82.9	21.6	16	7	23																				
Sato 2022 [321]	Janan	MP+MET	76.1	23.1	5	3	8	Older adults	1	6	20	NR	MET (AET, RET, MobT),	RPE 11-13/20	11 (33	3	NR					V	V				0, 3
	(Asia)	PLA (CHO)+MET	75.6	23.1	3	5	8	with chronic CVA					60 min, supervised	(15-point Borg sacle)	sessions)												
Seino 2017 [322]	Janan	DP+RET	74.9	23.3	14	24	38	Pre-frail or frail	1	2	$1.2\ g \cdot kg^{-1} \cdot d^{-1}$	90.4	RET, 60 min, supervised,	20 RM;	2	12	90.4				V		V		V		0, 12, 24
	(Asia)	RC	74.3	23.7	10	29	39	older adults				88.9	lower-limb Ex	RPE: somewhat hard			88.9										
Seino 2018 [323]	Janan	MP+RET	73.4	22.9	34	6	40	Sedentary, medically-	1	7	10.5	>90	RET	RPE 5-7/10 (200 RM)	2	12	95.3	V	V		V	V	V	V	V		0, 12
	(Asia)	RET	73.7	22.9	33	7	40	stable older adults									94.6										
Serra-Prat 2017 [324]	Spain	DP+MET	77.9	29.3	41	39	80	Pre-frail	1	7	$1.2\ g \cdot kg^{-1} \cdot d^{-1}$	47.5	MET (AET, RET, BalaT), 60 min,	10~15 RM	≥4	52	47.5				V		V		V		0, 52
	(Europe)	RC	78.8	28.5	56	36	92	older people					Walking; home-based UE/LE ex														
Shahar 2013 [325]	Malaysia	SP+MET	65.2	26.5	18[c]	47[c]	15	Elderly with sarcopenia	1	7	20 (men); 40 (women)	86.7	MET (AET, RET, BalaT), 60 min.	Moderate intensity;	2	12	86.7	V	V								0, 6, 12
	(Asia)	MET	69.7	23.7			19						supervised whole-body Ex	RPE (15-point Borg scale):													
		SP	65.9	24.3			15							12-14/20													
		RC	67.3	26.4			16																				
Shenoy 2013 [326]	India	SP+RET	54.1	28.9	20	0	20	Osteopenic/osteoporotic	1	7	40	NR	RET, 40~50 min,	60~80% 1-RM	4	12	NR					V					0, 12
	(Asia)	SP	54.6	31.0	20	0	20	postmenopausal women					supervised, UE/LE Ex														
		RC	54.1	30.0	20	0	20																				
Soares 2023 [327]	Brazil	WP+RET	68.1	29.3	0	14	14	Older adults	1	2	20	NR	RET, 40~60 min, supervised, whole-body Ex	70% 1-RM	2	12	NR	V			V	V					0, 4, 8, 12
	(America)	PLA (CHO)+RET	68.9	26.8	0	14	14	with type 2 DM						(RPE 7-8/10 OMNI sacle)													
Spoelder 2023 [328]	Netherlands	WP+AET	72.0	23.7	3	12	15	Untrained, independent	1	7	25	NR	AET, long-distance walking	NR	7	2	NR	V	V		V	V					0, 1, 2
	(Europe)	SP+AET	69.0	24.6	3	12	15	walk, medically-stable			25		(≥20 km, <30 km)														
		PLA (CHO)+AET	69.0	24.4	3	12	15	older adults																			
Sugawara 2010 [331];	Janan	WP+MET	77.4	19.6	2	15	17	Malnourished older adults	1	7	20	NR	MET (AET, RET, StreE), unsupervised	Low intensity,	7	12	NR	V		V		V	V				0, 12
2012 [332]	(Asia)	RC	77.1	20.7	0	14	14	with COPD					home-based, UE/LE Ex, level walking	40~50% VO2 peak													
Swanenburg 2007 [334]	Switzerland	MP+MET	71.8	23.9	12	0	12	Elderly with osteopenia	1	7	18.2	NR	MET (AET, RET, BalaT), 70 min,	8 RM	3	12	93	V				V					0, 12
	(Europe)	RC	70.7	22.6	12	0	12	Or osteoporosis					supervised														
Tang 2020 [335]	China	WP+RET	76.6	19.3	0	100	100	Elder inpatient	2	7	25	NR	RET	20-30 RM	6	24	NR				V					V	0, 24
	(Asia)	RC	77.0	19.5	0	100	100	with sarcopenia																			
Tarazona-Santabalbina	Spain	DP+MET	79.7	29.9	29	22	51	Frail elderly	1	7	$0.8\ g \cdot kg^{-1} \cdot d^{-1}$	NR	MET (AET, RET, BalaT, StreE), 65 min,	AET: 65% HR_{max};	5	24	47.3	V		V			V		V	V	0, 24
2016 [336]	(Europe)	DP	80.3	30.0	25	24	49				(50% protein of daily diet)		supervised, walking, UE/LE ex	RET: 75% 1-RM													

To be continued.

Table S2. Continued.

Study (year) [Reference No.][a]	Country (area)	Study arm	Age (years) Mean	BMI (kg/m^2) Mean	Sex (n) Women	Sex (n) Men	Study sample (n)	Health status (medical condition)	Living status[d]	Protein supplementation: Frequency (day /week)	Protein supplementation: Intake amount (g/d or g/session)	Protein supplementation: Compliance (%)	Exercise intervention: Modality	Exercise intervention: Intensity (% 1-RM/RPE/HR$_{max}$)	Exercise intervention: Frequency (session /week)	Exercise intervention: Duration (week)	Exercise intervention: Compliance (%)	Outcome measures[e] Muscle mass/volume: Whole body lean	Muscle mass/volume: ALM	Muscle mass/volume: Muscle volume	Strength: Handgrip strength	Strength: Leg strength	Mobility: Walk speed	Mobility: Chair rise	Mobility: TUG	Mobility: SPPB	Follow-up time point (week)
Ten Haaf 2019 [337]	Netherlands	MP+AET	69.0	27.2	11	47	58	Malnourished older adults	1	7	31	96	AET, 4-day walking event of	NR	4	12	82.6[f]	V	V		V	V	V	V	V	V	0, 12
	(Europe)	PLA (CHO)+AET	69.0	26.3	10	46	56	Low protein intake				95	30~50 km/day				74.5[f]										
Thomson 2016 [338]	Australia	MP+RET	61.3	27.7	29	25	54	Untrained overweight	1	7	27	97	RET, supervised, whole-body ex	8-12 RM	3	12	91.4	V			V	V	V				0, 12
	(Oceania)	SP+RET	61.7	27.5	35	29	64	older adults			27	98.1					93.2										
		PLA+RET	61.5	27.6	34	27	61				1.1	98.4					92.9										
Travers 2023 [340]	Ireland	DP+MET	77.6	28.1	54	25	79	Pre-frail and frail	1	7	20	82.3	MET (AET, RET), 30~40 min,	RET: 10~15 RM	3~7	12	92.4	V			V						0, 12
	(Europe)	RC	76.5	27.4	51	26	77	elderly			(1.2 g・kg^{-1}・ d^{-1})		home-based														
Trevisan 2010 [341]	Brazil	SP+RET	58.0	28.0	15	0	15	Postmenopausal women	1	7	25	NR	RET, 90 min, supervised,	60-80% 1-RM	3	12	NR	V									0, 12
	(America)	PLA (CHO)+RET	57.0	28.0	15	0	15	with sarcopenia risk					whole-body ex														
		SP	60.0	29.0	15	0	15																				
		PLA (CHO)	60.0	27.0	15	0	15																				
Tsurumi 2022 [342]	Janan	MP+RET	74[c]	23.9	4[c]	7[c]	11	Patients with end-stage	1	3	15	NR	RET, NMES, 30 min, supervised,	Maximal intensity as tolerated	3	12	NR	V		V	V		V		V	V	0, 12
	(Asia)	MP		23.8			11	diabetic kidney disease					lower-limb Ex, isometric contraction ex														
Uchida 2024 [343]	Janan	Meat+RET	59~79[c]	21.1	18	0	18	Community- dwelling,	1	3	22.5	>90	RET, 30 min, supervised,	70% 1-RM	3	12	≥ 90	V	V			V					0, 12
	(Asia)	PLA (CHO)+RET		23.0	20	0	20	sedentary, older women					lower-limb Ex				≥ 90										
		Meat		22.6	22	0	22					>90															
		PLA (CHO)		22.6	21	0	21					>90															
Unterberger 2022 [344]	Australia	SP+RET	73.2	26.2	28	20	48	Untrained	1	2	32 (2.0 g・kg^{-1}・ d^{-1})	NR	RET, 45~60 min, supervised,	OMNI RPE 6-7/10	2	10	NR	V	V		V	V	V	V	V		0, 10
	(Oceania)	PLA (CHO)+RET	72.4	26.3	21	20	41	older adults			1.0 g・kg^{-1}・ d^{-1}		whole-body Ex														
		RC	73.0	26.0	24	23	47																				
van den Helder 2020 [346]	Netherlands	DP+MET	70.8	27.0	49	19	68	Community-dwelling	1	7	1.2~1.5 g・kg^{-1}・ d^{-1}	40~69	MET (RET, BalaT, MobT, StreE),	RPE: moderate to vigorous,	2~5	52	49		V		V		V		V	V	0, 24, 52
	(Europe)	MET	72.3	25.3	43	22	65	older adults				13~35	home-based	5-8/10 (10-point Borg)			75										
		RC	72.8	25.7	66	25	91					14~40															
van Dongen 2020 [347]	Netherlands	DP+RET	74.7	27.1	51	31	82	Frail/prefrail	1	7	75	98.8	RET, 60 min, supervised, group-based,	75~80% 1-RM	2	12	83.6	V	V			V	V	V	V	V	0, 12
	(Europe)	DP+MET	74.7	27.1	51	31	82	older adults		7	NR	76.8	MET (RET, BalaT, MobT), 65 min,		1~2		63.6										
		RC	75.9	26.3	51	35	86						supervised, group-based, lower-limb ex														
Verceles 2023 [348]	USA	WP+MET	62.0	29.0	8	8	16	Acutely Hospitalized	2	7	1.75 g・kg^{-1}・ d^{-1}	92.9	MET (AET, RET, MobT), 60 min,	Maximal intensity as tolerated	5 (14	2	82			V							0, 1, 2
	(America)	MET	62.0	30.0	13	10	23	older patients			(50% protein of daily diet)		Supervised inpatient physiotherapy		sessions)		64										
Verdijk 2009 [349]	Netherlands	Casein+RET	72.0	26.5	0	13	13	Untrained, free-living	1	3	20	NR	RET, supervised, whole-body Ex	75-80% 1-RM	3	12	97.2	V	V	V							0, 12
	(Europe)	PLA(water)+RET	72.0	27.4	0	13	13	elderly people									97.2										
Verreijen 2015 [351]	Netherlands	WP+RET	63.7	32.7	16	14	30	Untrained, obese	1	7	20 (non-training day);	91	RET, 60 min, supervised,	Maximal weight as	3	13	72	V	V		V		V	V			0, 13
	(Europe)	PLA (CHO)+RET	63.0	33.3	16	14	30	older adults			40 (training day)	97	whole-body Ex	tolerated (12 RM)			88										
Verreijen 2017 [350]	Netherlands	DP+RET	61.5	31.6	19	13	32	Overweight/obese	1	7	1.3 g・kg^{-1}・ d^{-1}	87	RET, 60 min, supervised,	10 RM	3	10	93.3	V			V		V	V			0, 10
	(Europe)	RET	63.1	32.2	16	9	25	older adults				78	whole-body Ex				93.3										
		DP	61.9	32.1	13	8	21					87															
		RC	63.4	33.2	16	6	22					78															
Vijayakumaran 2023 [352]	Malaysia	WP+RET	66.6	26.4	8	0	8	Inactive, sarcopenic	1	5	15	90	RET, home-based,	75-80% 1-RM	3	12	90		V	V	V			V		V	0, 12
	(Asia)	RET	65.5	24.5	8	0	8	older women					whole-body Ex				90										

To be continued.

Table S2. Continued.

Study (year) [Reference No.][a]	Country (area)	Study arm	Age (years) Mean	BMI (kg/m²) Mean	Sex (n) Women	Sex (n) Men	Study sample (n)	Health status (medical condition)	Living status[d]	Protein supplementation: Frequency (day /week)	Protein supplementation: Intake amount (g/d or g/session)	Protein supplementation: Compliance (%)	Exercise intervention: Modality	Exercise intervention: Intensity (% 1-RM/RPE/HR_{max})	Exercise intervention: Frequency (session /week)	Exercise intervention: Duration (week)	Exercise intervention: Compliance (%)	Outcome measures[e]: Muscle mass/volume: Whole body lean	Muscle mass/volume: ALM	Muscle mass/volume: Muscle volume	Strength: Handgrip strength	Strength: Leg strength	Mobility: Walk speed	Mobility: Chair rise	Mobility: TUG	Mobility: SPPB	Follow-up time point (week)
Vikberg 2019 [353]	Sweden	MP+RET	70.9	22.7	20	16	36	Old individuals with	1	7	20-30	84	RET, 45 min, supervised,	Moderate to high:	3	10	91	V	V		V		V	V	V	V	0, 10
	(Europe)	RC	70.0	23.3	18	16	34	pre-sarcopenia					group-based, whole-body Ex	RPE 6-7/10 (10-point Borg)													
Villanueva 2014 [354]	USA	WP+Cre+RET	68.7	25.3	0	7	7	Untrained, recreationally	1	7	35 (>1.0 $g \cdot kg^{-1} \cdot d^{-1}$)	100	RET, individually supervised,	70% 1-RM	3	12	94	V				V	V				0, 6, 12
	(America)	RET	68.7	27.1	0	7	7	active older adults					lower-body Ex				94										
		RC	67.1	26.8	0	8	8																				
Vorup 2017 [355]	Denmark	MP+MET	69.0	26.0	7	6	13	Untrained, recreational	1	2~3	36	100	MET (RET, BalaT, MobT), 20 min,	72~73% HR_{max}	2~3	12	72.9	V	V			V		V	V		0, 12
	(Europe)	PLA+MET	74.0	28.0	10	8	18	active, older adults			6	100	small-sided floorball training,				67										
		RC	72.0	25.0	9	8	17						supervised, group-based														
Wang ZY 2022 [357]	China	DP+MET	70.2	23.7	42	8	50	Elderly people	1	7	10		MET (AET, RET), 40~60 min,	Moderate to high	≥3	12			V				V	V			0, 12
	(Asia)	MET	69.7	23.2	40	10	50	with sarcopenia					home-based, whole-body ex														
		DP	68.2	22.3	41	9	50																				
		RC	69.9	22.7	44	7	51																				
Wang XJ 2023 [356]	China	WP+RET	68.1	22.6	44	31	75	Elderly people	1	7	30	NR	RET, 40~50 min, supervised, UE/LE Ex	NR (8~10 RM)	7	24	NR	V				V				V	0, 24
	(Asia)	DP+AET	68.2	22.9	41	34	75	with sarcopenia					AET	NR	7		NR										
Weinheimer 2012 [358]	USA	WP+MET	47.5	30.3	83	53	136	Overweight/obese	1	7	20~60	74.8~80.6[f]	MET (AET, RET), 60 min,	AET: 70% HR_{max};	3	12	88.1~91.0[f]	V	V								0, 6, 12
	(America)	PLA(CHO)+MET	49.0	29.9	50	34	84	middle-aged adults				73.9~78.5[f]	supervised, whole-body ex	RET: 80% 1-RM;			88.1~90.5[f]										
														RPE: 12~15/20 Borg score													
Weisgarber 2015 [359]	Canada	WP+RET	57[c]	28.3[c]	12	0	12	Untrained, sedentary,	1	4	40	100	RET, supervised,	30% 1-RM	4	10	NR		V	V		V					0, 10
	(America)	PLA(CHO)+RET			12	0	12	postmenopausal women					machine-based UE/LE Ex														
Wu 2018 [360]	Taiwan	SP+MET	74.4[c]	26.0[c]	20[c]	16[c]	5	Community-dwelling	1	7	16	86.3~97.7[c]	MET, physical therapy	NR	NR	12	NR				V		V				0, 12
	(Asia)	DP+MET					5	pre-frail or frail			25																
		MET					9	older adults																			
		SP					4				16																
		DP					4				25																
		RC					9																				
Wu 2019 [361]	China	DP+RET	78.9	NR	19	21	40	Elderly patients	1	7	1.2 $g \cdot kg^{-1} \cdot d^{-1}$	NR	RET, 40 min, supervised,	30 RM;	3	48	NR		V		V		V		V		0, 48
	(Asia)	DP	79.9		20	20	40	with sarcopenia			(50% protein of daily diet)		machine-based UE/LE Ex	3 set x 30 rep	(6 sessions)												
Xie 2021 [362]	China	WP+MET	75.7	NR	16	24	40	Elderly inpatients	2	7	32	NR	MET (AET, RET, BalaT, MobT),	Maximal intensity	2	52	NR		V		V		V				0, 52
	(Asia)	RC	72.2		13	17	30	with sarcopenia					20~30 min, group-based	as tolerated													
Xing 2018 [363]	China	WP+MET	80.3	23.9	35	49	84	Elderly patients	1	7	25 (1.2 $g \cdot kg^{-1} \cdot d^{-1}$;	NR	MET (AET, RET), 60 min, supervised	70% $\dot{V}O_2$ peak	7	12	NR						V				0, 12
	(Asia)	DP+AET	80.9	23.8	41	44	85	with sarcopenia		7	50% protein of daily diet)	NR	AET, 30 min, unsupervised	NR	7												
Xu 2021 [364]	China	DP+MET	72.3	19.3	31	57	88	Elderly inpatients	2	7	NR	NR	MET (AET, RET), 30 min,	Maximal intensity	3~5	12	NR		V							V	0, 12
	(Asia)	RC	71.6	19.4	36	53	89	with sarcopenia					outdoor aerobic ex, supervised, UE/LE ex	as tolerated													
Xue 2023 [365]	China	WP+RET	69.9	NR	6	9	15	Elderly inpatient	2	7	1.0 $g \cdot kg^{-1} \cdot d^{-1}$	NR	RET, supervised	10~15 RM,	5	4	NR		V		V						0, 4
	(Asia)	RET	69.9		4	11	15	with CVA and sarcopenia						2 set/d													
		WP	70.0		6	9	15																				
		RC	70.9		8	7	15																				

To be continued.

Table S2. Continued.

Study (year) [Reference No.][a]	Country (area)	Study arm	Age (years) Mean	BMI (kg/m²) Mean	Sex (n) Women	Sex (n) Men	Study sample (n)	Health status (medical condition)	Living status[d]	Protein supplementation: Frequency (day /week)	Protein supplementation: Intake amount (g/d or g/session)	Protein supplementation: Compliance (%)	Exercise intervention: Modality	Exercise intervention: Intensity (% 1-RM/RPE/HR_{max})	Exercise intervention: Frequency (session /week)	Exercise intervention: Duration (week)	Exercise intervention: Compliance (%)	Outcome measures[e] Muscle mass/volume: Whole body lean	Muscle mass/volume: ALM	Muscle mass/volume: Muscle volume	Strength: Handgrip strength	Strength: Leg strength	Mobility: Walk speed	Mobility: Chair rise	Mobility: TUG	Mobility: SPPB	Follow-up time point (week)
Yamada 2015 [367]	Janan	MP+AET	76.3	22.3	50	27	77	Community-Dwelling,	1	7	10	79.7[f]	AET, supervised,	Increase the number of daily		24	100	V									0, 24
	(Asia)	AET	75.8	22.7	48	22	70	frail and non-frail					pedometer-based walking	steps by 10% per month			100										
		RC	75.7	23.1	44	31	75	older adults					with 0.5-kg ankle weight														
Yamada 2019 [366]	Janan	WP+RET	84.9	21.3	20	8	28	Older adults with	1	7	10	89.5[f]	RET, 30 min, supervised,	20 RM	7	12	85.6[f]		V		V	V	V	V			0, 12
	(Asia)	RET	84.7	22.6	18	10	28	Sarcopenia/dynapenia					body-weight lower-limb ex;	3 set x 20 rep			78.2[f]										
		WP	83.2	22.6	20	8	28					97.0[f]	home-based daily self-ex														
		RC	83.9	21.2	15	13	28																				
Yin 2019 [368]	China	DP+RET	69.5	NR	17	33	50	sarcopenic older adults	1	7	1.0~1.2 $g \cdot kg^{-1} \cdot d^{-1}$	NR	RET, supervised, individualized	Maximal intensity	NR	12	NR		V		V						0, 12
	(Asia)	RET	68.5		15	35	50							as tolerated													
Yoshimura 2016 [369]	Janan	MP+RET	78.4	20.8	15	4	19	Hospitalized frail elderly	2	7	10	NR	RET, supervised	10 RM	7	10	NR			V	V						0, 10
	(Asia)	PLA+RET	82.3	20.4	11	6	17						machine-based UE/LE Ex	3 set x 10 rep													
Zak 2009 [370]	Poland	MP+RET	78.1	24.7	16	3	19	Institutionalized/	2	5	12	100	RET, 45 min, supervised, lower-limb ex	80% 1-RM	5	7	100					V	V				0, 7
	(Europe)	MP+AET	78.3	25.2	14	5	19	free-living				100	AET, 45 min, pedal exerciser	NR	5		100										
		PLA (CHO)+RET	79.2	24.3	17	4	21	frail elderly				100					100										
		PLA (CHO)+AET	81.1	25.2	17	4	21					100					100										
Zdzieblik 2015 [372]	Germany	Collagen+RET	72.3	NR	0	26	26	Elderly men	1	7	15	100	RET, 60 min, supervised,	8 RM	3	12	≥90	V				V					0, 12
	(Europe)	PLA (silica)+RET	72.1		0	27	27	with sarcopenia				100	individualized, whole-body ex				≥90										
Zdzieblik 2021 [371]	Germany	WP+RET	49.6	30.2	0	36	36	Sedentary, overweight	1	7	15	100	RET, 60 min, supervised,	85% 1-RM	3	12	>83.3	V	V			V					0, 12
	(Europe)	Collagen+RET	51.8	31.0	0	30	30	middle-aged adults		7	15	100	whole-body ex	3 set x 8 rep			>83.3										
		PLA (silica)+RET	47.4	29.9	0	31	31					100					>83.3										
Zhang LY 2023a [374]	China	MP+RET	75.6	NR	7	32	39	Elderly Patients	1	7	20	NR	RET, 40 min, supervised, UE/LE ex	≥70% 1-RM	6	24	≥70		V		V						0, 24
	(Asia)	RC	75.6		8	31	39	with sarcopenia						3 set x 30 rep													
Zhang LY 2023b [373]	China	DP+RET	75.0	NR	7	32	39	Elderly with DM	1	7	50	NR	RET, 40 min, supervised,	≥70% 1-RM	6	24	≥70		V		V						0, 24
	(Asia)	RC	74.5		8	31	39	and sarcopenia					UE/LE ex	3 set x 30 rep													
Zhao YF 2021 [376]	China	DP+MET	71.8	22.4	22	19	41	Elderly Patients	1	7	1.2 $g \cdot kg^{-1} \cdot d^{-1}$	100	MET (AET, RET), 45 min,	Maximal intensity	5	12	100		V		V		V		V		0, 12
	(Asia)	DP	71.8	22.5	10	11	21	with sarcopenia				100	supervised, treadmill-level walk,	as tolerated													
Zhao YY 2021 [377]	China	DP+RET	71.1	19.5	19	23	42	Elderly Patients	1	7	1.0~1.2 $g \cdot kg^{-1} \cdot d^{-1}$	NR	RET, 40 min, supervised,	≥70% 1-RM	6	24	NR		V		V					V	0, 24
	(Asia)	RET	70.6	19.5	16	26	42	with sarcopenia					UE/LE ex	3 set x 30 rep													
Zhao 2022 [375]	China	WP+RET	61.9	19.9	12	8	20	Elderly Patients	1	7	24	NR	RET, 50 min, supervised,	8 RM	7	12	NR	V			V						0, 4, 8, 12
	(Asia)	WP	64.9	18.6	12	8	20	with sarcopenia					whole-body ex		(14												
		RC	62.9	19.8	12	8	20								sessions)												
Zhou 2023 [378]	China	DP+RET	79.9	22.5	152	98	250	Elderly patients	1	7	1.0~1.2 $g \cdot kg^{-1} \cdot d^{-1}$	NR	RET, supervised, whole-body ex	NR	7	24	NR		V		V		V				0, 24
	(Asia)	DP	80.1	22.3	149	101	250	with sarcopenia																			
Zhu 2019 [379]	China	SP+MET	74.8	18.8	29	7	36	Elderly patients	1	7	17.2	86.3	MET (AET, RET), 90 min,	RET: 40% 1-RM	3	12	92.8		V		V	V	V	V			0, 12, 24
	(Asia)	MET	74.5	18.8	29	11	40	with sarcopenia					supervised, group & home-based,	6 set x 6~8 rep			95.3										
		RC	72.2	18.9	29	8	37						UE/LE ex														
Zong 2023 [380]	China	WP+MET	80.5	21.6	14	13	27	Elderly inpatients	2	7	20	94.5[f]	MET(AET, RET, breathing),	AET: 85% HR_{max};	4	12	>90[f]	V	V		V		V				0, 12
	(Asia)	MET	81.1	24.6	14	15	29	with COPD					20 min, supervised	RET: 70~85% 1-RM		12											
		RC	85.9	22.1	18	10	28							(low to moderate)		12											

To be continued.

Table S2. Continued.

Study (year) [Reference No.][a]	Country (area)	Study arm	Age (years)	BMI (kg/m²)	Sex (n)		Study sample (n)	Health status (medical condition)	Living status[d]	Protein supplementation			Exercise intervention					Outcome measures[e]									Follow-up time point (week)
																		Muscle mass/volume			Strength		Mobility				
			Mean	Mean	Women	Men				Frequency (day /week)	Intake amount (g/d or g/session)	Compliance (%)	Modality	Intensity (% 1-RM/RPE/HR_{max})	Frequency (session /week)	Duration (week)	Compliance (%)	Whole body lean	ALM	Muscle volume	Handgrip strength	Leg strength	Walk speed	Chair rise	TUG	SPPB	
Blanc-Bisson 2008 [142]	France	MP+RET	85.5	25.1	25	13	38	Hospitalized older patients	2	7	15	NR	RET, 90 min, supervised, inpatient and	10 RM	5	6	NR			V	V						0, 6
	(Europe)	MP	85.4	22.9	30	8	38	under acute care					home-based physiotherapy														
Myint 2013 [288]	Hong Kong	MP+MET	80.9	21.6	42	19	61	Hospitalized elderly	2	7	36~48	77.7	MET	NR	7	4	NR			V	V	V				V	0, 4
	(Asia)	MET	81.7	24.6	38	22	60	with hip fracture					(inpatient rehabilitation)														
van de Bool 2017 [345]	Netherlands	WP+MET	62.8	21.6	24	18	42	Older patients with COPD	1	7	18.8~28.2	70	MET (AET, RET), supervised, cycle ergometry;	AET:high intensity;	2~3	16	NR	V	V			V	V				0, 16
	(Europe)	PLA+MET	62.2	24.6	16	23	39					17	treadmill walking, UE/LE ex	RET: 75% 1-RM	40 sessions												

[a]Complete citation details for each reference number can be found at the end of the main text.

[b]Data is represented as total number of study sample.

[c]Data is represented as mean value of study sample.

[d]Coding: Noninstitutionalized = 1; Institutionalized = 2.

[e]Whole body lean measures including skeletal muscle mass (kg), fat-free mass (kg), and lean mass index (kg/m²); walking speed including measures of gait speed (m/s), 6-minuate walk distance (m), and 400-meter walk time (s).

[f]Data is estimated.

1-RM, repetition maximum; ADL, activity of daily life; AET, aerobic exercise training; ALM, appendicular lean mass; BalaT, balance training; BMI, body mass index; BCAA, branched-chain amino acids; CHO, carbohydrates; COPD, chronic obstructive pulmonary disease; Cre, creatine; CSA, cross-section area; CVA, cerebrovascular accident; DM, diabetes mellitus; REHC, restricted energy higher carbohydrate; UK, United Kingdom; USA, United States of America; MobT, mobility training; StreE, stretching exercise; MFGM, milk fat globule membrane; NMES, neuromuscular electrical stimulation; NR not reproted; SPPB, short physical performance battery; TEE, total energy expenditure; TUG, timed up-and-go; ROB, risk of bias; ω3FA, omega-3 fatty acids; DP, dietary protein; Insect-PS, insect protein supplement; MET, multicomponent exercise training; MP, milk protein; RET, resistance exercise training; SP, soy protein; WP, whey protein; RC, regular care.

Supplementary table S3. Outcome measures identified among the included trials.

Study (Author, year)	Muscle mass/volume			Strength		Mobility			
	Whole body	Appendicular (Arm and leg)	Volume	Handgrip	Leg	Walk speed	Chair stand	TUG	Global mobility
Aas 2020		Leg lean mass	Thigh muscle thickness		1-RM leg press	10-meter walk	5-time CR		
Amasene 2021	WBLM	Leg lean mass	Calf girth	Handgrip, MVIC (handled dynamometer)		6-minute walk	30-second STS		SPPB
Arciero 2014	WBLM								
Arentson-Lantz 2019	WBLM	Leg lean mass			Isokinetic knee extension	3-meter walk	5-time CR		SPPB
Arentson-Lantz 2020	WBLM	Leg lean mass			Knee extension, MVIC	3-meter walk	5-time CR		SPPB
Arnarson 2013	WBLM	ALM			1-RM leg press	6-minute walk		3-meter TUG	
Assantachai 2020		ALMI		Handgrip, MVIC (handled dynamometer)	Knee extension, MVIC	Usual walk speed			
Atherton 2020	FFM			Handgrip, MVIC (handled dynamometer)	1-RM leg press				
Bagheri 2022	WBLM				1-RM leg press				
Bauer 2024	FFM	ALM		Handgrip, MVIC (handled dynamometer)		Usual walk speed			
Beck 2016				Handgrip, MVIC (handled dynamometer)			30-second STS		
Bell 2017	WBLM	ALM			1-RM leg press				
Bemben 2010; Eliot 2008	FFM	ALM			1-RM leg press				
Bernabei 2022		ALM		Handgrip, MVIC (handled dynamometer)					SPPB
Biesek 2021	FFM	ALM	Thigh muscle thickness	Handgrip, MVIC (handled dynamometer)	Calf muscle strength				
Bijeh 2022	WBLM			Handgrip, MVIC (handled dynamometer)	1-RM leg press				
Bjorkman 2011	WBLM	ALM		Handgrip, MVIC (handled dynamometer)		10-meter walk	5-time CR		
Bjorkman 2012	LBMI			Handgrip, MVIC (handled dynamometer)	Knee extension, MVIC				
Bjorkman 2020	LBMI			Handgrip, MVIC (handled dynamometer)					SPPB
Blanc-Bisson 2008			Arm and calf girth						
Bonnefoy 2003	FFM				Isokinetic knee extension	6-meter walk	5-time CR		
Bonnefoy 2012	FFM					Usual walk speed	60-second STS	3-meter TUG	
Botella-Carretero 2008			Arm girth						
Boutry-Regard 2020	WBLM		Thigh muscle thickness		1-RM leg press	6-meter walk			
Buhl 2016	WBLM	ALM		Handgrip, MVIC (handled dynamometer)			30-second STS		
Bunout 2004	WBLM		Arm girth	Handgrip, MVIC (handled dynamometer)	1-RM leg press	12-minute walk			
Candow 2006	WBLM		Thigh muscle thickness		1-RM leg press				
Candow 2008	WBLM		Thigh muscle thickness		1-RM leg press				
Cao 2007	FFM			Handgrip, MVIC (handled dynamometer)		10-meter walk	30-second STS		
Carlsson 2011; Rosendahl 2006					1-RM leg press	3-meter walk	10-time CR		
Carroll 2024			Thigh volumn		1-RM leg press				
Centner 2019			Thigh muscle CSA		1-RM leg press				
Chale 2013	WBLM		Thigh muscle CSA		Isokinetic knee extension	400-meter walk	10-time CR		SPPB
Chang 2019				Handgrip, MVIC (handled dynamometer)					SPPB
Chatterjee 2018				Handgrip, MVIC (handled dynamometer)		Usual walk speed			
Chen 2017				Handgrip, MVIC (handled dynamometer)					SPPB
Chen 2021; Hsu 2021	WBLM	ALM			Isokinetic knee extension		30-second STS		

To be continued.

Supplementary table S3. Continued.

Study (Author, year)	Muscle mass/volume: Whole body	Muscle mass/volume: Appendicular (Arm and leg)	Muscle mass/volume: Volume	Strength: Handgrip	Strength: Leg	Mobility: Walk speed	Mobility: Chair stand	Mobility: TUG	Mobility: Global mobility
Chen 2024	FFM	ALM		Handgrip, MVIC (handled dynamometer)		6-minute walk			SPPB
Chin A Paw 2001; de Jong 2000	WBLM			Handgrip, MVIC (handled dynamometer)	Knee extension, MVIC	Usual walk speed	5-time CR		
Colonetti 2023	WBLM	Leg lean mass							
Corcoran 2017				Handgrip, MVIC (handled dynamometer)		400-meter walk			SPPB
Dalla Via 2021	WBLM	ALM	Arm CSA (mm^2)	Handgrip, MVIC (handled dynamometer)	3-RM leg press	Usual walk speed	30-second STS	3-meter TUG	
Daly 2014	WBLM		Thigh muscle CSA		1-RM leg press		30-second STS	3-meter TUG	
Daly 2020	WBLM	ALM	Thigh muscle CSA	Handgrip, MVIC (handled dynamometer)	1-RM leg press	Usual walk speed	5-time CR	3-meter TUG	
de Azevedo Bach 2022	WBLM	ALM			1-RM leg press		30-second STS	3-meter TUG	
de Carvalho Bastone 2020	LBMI			Handgrip, MVIC (handled dynamometer)		Usual walk speed	5-time CR	3-meter TUG	
Deer 2019	WBLM	ALM				Usual walk speed			SPPB
Deibert 2011	FFM				1-RM leg press				
Duff 2014	WBLM		Thigh muscle thickness		1-RM leg press				
Dulac 2021	WBLM	Leg lean mass	Thigh muscle CSA	Handgrip, MVIC (handled dynamometer)	1-RM leg press	4-meter walk	5-time CR	3-meter TUG	
Edholm 2017; Strandberg 2015	WBLM	Leg lean mass			1-RM leg press		5-time CR	3-meter TUG	
Englund 2017; Fielding 2017	WBLM	ALM	Thigh muscle CSA		Knee extension, MVIC	400-meter walk			SPPB
Evans 2007	WBLM								
Fernandes 2018; Sugihara Junior 2018	WBLM	ALM			Knee extension, MVIC				
Fiatarone 1994			Thigh muscle CSA		1-RM leg press	6-meter walk			
Filho 2022			Thigh muscle thickness	Handgrip, MVIC (handled dynamometer)	Isokinetic knee extension	4-meter walk	5-time CR	3-meter TUG	
Flodin 2015	FFM	ALM		Handgrip, MVIC (handled dynamometer)					
Formica 2020	WBLM	ALM	Thigh muscle CSA		Knee extension, MVIC	4-meter walk	30-second STS	3-meter TUG	
Francis 2017	WBLM	Leg lean mass			Isokinetic knee extension	900-meter walk	5-time CR		
Franzke 2015a; 2015b				Handgrip, MVIC (handled dynamometer)		6-minute walk	30-second STS		
Fujie 2025			Thigh muscle CSA		1-RM leg press				
Furtado 2024	WBLM	ALMI	Calf girth	Handgrip, MVIC (handled dynamometer)	1-RM leg press				
Gade 2019	WBLM	ALM		Handgrip, MVIC (handled dynamometer)		4-meter walk	30-second STS		
Gaffney 2018			Thigh muscle thickness		1-RM leg press				
Galbreath 2018	FFM				1-RM leg press	6-minute walk			
Gao 2019	LBMI			Handgrip, MVIC (handled dynamometer)		6-meter walk	5-time CR	3-meter TUG	
George 2017				Handgrip, MVIC (handled dynamometer)	1-RM leg press	6-minute walk		3-meter TUG	
Grabovac 2018; Haider 2017; Kapan 2017	WBLM	ALM		Handgrip, MVIC (handled dynamometer)	1-RM leg press		5-time CR		SPPB
Granic 2020		ALM		Handgrip, MVIC (handled dynamometer)		Usual walk speed	5-time CR		SPPB
Griffen 2022	FFM	ALM		Handgrip, MVIC (handled dynamometer)	1-RM leg press	4-meter walk			SPPB
Gronstedt 2020	FFM					Usual walk speed	30-second STS		
Gusdon 2024			Thigh volumn						
Gryson 2014	FFM	ALM			Knee extension, MVIC				
Haß 2022	FFMI			Handgrip, MVIC (handled dynamometer)	1-RM leg press	4-meter walk	5-time CR		

To be continued.

Supplementary table S3. Continued.

Study (Author, year)	Muscle mass/volume: Whole body	Appendicular (Arm and leg)	Volume	Strength: Handgrip	Leg	Mobility: Walk speed	Chair stand	TUG	Global mobility
Hamarsland 2019	WBLM	ALM	Thigh muscle thickness		1-RM leg press		5-time CR		
Han 2021	FFM			Handgrip, MVIC (handled dynamometer)	1-RM leg press	Usual walk speed			
Han 2024		ALMI		Handgrip, MVIC (handled dynamometer)		6-minute walk			
Hankey 1993			Arm girth						
Haub 2002; 2005	FFM		Thigh muscle CSA		1-RM leg press				
He 2022				Handgrip, MVIC (handled dynamometer)					
Hegerova 2015	WBLM								
Herda 2021	FFM			Handgrip, MVIC (handled dynamometer)	5-RM leg press	30-minute walk		3-meter TUG	
Hofmann 2016; Oesen 2015; Strasser 2022	WBLM		Thigh muscle thickness	Handgrip, MVIC (handled dynamometer)	Isokinetic knee extension	6-meter walk	30-second STS		
Holm 2008	WBLM		Thigh muscle CSA		1-RM leg press				
Holwerda 2018	WBLM	ALM			1-RM leg press	4-meter walk	5-time CR		SPPB
Hotta 2021	WBLM	ALMI		Handgrip, MVIC (handled dynamometer)	Isokinetic knee extension	6-minute walk			
Hsieh 2019				Handgrip, MVIC (handled dynamometer)	1-RM leg press	10-meter walk			
Imaoka 2016	LBMI			Handgrip, MVIC (handled dynamometer)					
Imaoka 2019	LBMI			Handgrip, MVIC (handled dynamometer)		Usual walk speed			
Jadczak 2021	FFM	ALM		Handgrip, MVIC (handled dynamometer)		Usual walk speed		3-meter TUG	SPPB
Ji 2025a; 2025b	LBMI			Handgrip, MVIC (handled dynamometer)		Usual walk speed			SPPB
Jiang 2023	LBMI			Handgrip, MVIC (handled dynamometer)		Usual walk speed			
Jin 2016		ALMI		Handgrip, MVIC (handled dynamometer)		Usual walk speed			
Jyvakorpi 2023				Handgrip, MVIC (handled dynamometer)		Usual walk speed	5-time CR		SPPB
Kang 2019				Handgrip, MVIC (handled dynamometer)		4-meter walk	5-time CR		SPPB
Kang 2020	WBLM	ALM	Arm circumference (cm)	Handgrip, MVIC (handled dynamometer)	Knee extension, MVIC				SPPB
Karelis 2015	WBLM	ALM			1-RM leg press				
Kemmler 2016		ALMI		Handgrip, MVIC (handled dynamometer)		Usual walk speed			
Kemmler 2017		ALMI		Handgrip, MVIC (handled dynamometer)					
Kemmler 2020a; 2020b	LBMI			Handgrip, MVIC (handled dynamometer)	1-RM leg press	Usual walk speed			
Kim 2015		ALM		Handgrip, MVIC (handled dynamometer)	Knee extension, MVIC	5-meter walk		3-meter TUG	
Kirk 2019; 2020	WBLM			Handgrip, MVIC (handled dynamometer)	1-RM leg press	6-minute walk			SPPB
Koopmans 2024a; 2024b	WBLM			Handgrip, MVIC (handled dynamometer)	1-RM leg press				
Korzepa 2025	FFM	Leg lean mass	Thigh muscle thickness		Knee extension, MVIC				
Krause 2019	WBLM			Handgrip, MVIC (handled dynamometer)		10-meter walk	5-time CR		
Kukuljan 2009a; 2009b	WBLM		Thigh muscle CSA		1-RM leg press	Usual walk speed			
Kuwaba 2023					Knee extension, MVIC				
Kwon 2015				Handgrip, MVIC (handled dynamometer)		Usual walk speed			
Lamb 2020	WBLM		Thigh muscle CSA		1-RM leg press				
Laviolette 2010			Thigh muscle CSA		Knee extension, MVIC				
Leenders 2013	WBLM	Leg lean mass	Thigh muscle CSA	Handgrip, MVIC (handled dynamometer)	Knee extension, MVIC		5-time CR		

To be continued.

Supplementary table S3. Continued.

Study (Author, year)	Muscle mass/volume: Whole body	Muscle mass/volume: Appendicular (Arm and leg)	Muscle mass/volume: Volume	Strength: Handgrip	Strength: Leg	Mobility: Walk speed	Mobility: Chair stand	Mobility: TUG	Mobility: Global mobility
Li DT 2021		ALMI		Handgrip, MVIC (handled dynamometer)		Usual walk speed			
Li G 2025	WBLM	ALM	Calf girth	Handgrip, MVIC (handled dynamometer)				3-meter TUG	
Li WL 2022		ALM							
Li Y 2022				Handgrip, MVIC (handled dynamometer)					
Li Z 2021		ALM		Handgrip, MVIC (handled dynamometer)					
Liang 2023									
Liang 2024									
Liao 2019	WBLM			Handgrip, MVIC (handled dynamometer)		3-meter walk	5-time CR		SPPB
Liao 2021	LBMI	ALMI		Handgrip, MVIC (handled dynamometer)		Usual walk speed			
Liao 2022		ALMI		Handgrip, MVIC (handled dynamometer)					SPPB
Liu 2025					Isokinetic knee extension	6-minute walk	30-second STS		
Llaneza 2011	WBLM								
Long 2021		ALMI		Handgrip, MVIC (handled dynamometer)		Usual walk speed			
Ma 2023			Calf girth	Handgrip, MVIC (handled dynamometer)				3-meter TUG	
Macpherson 2022	WBLM	ALM		Handgrip, MVIC (handled dynamometer)		4-meter walk	30-second STS	3-meter TUG	
Maesta 2007	FFM	ALM							
Magrans-Courtney 2011	FFM								
Malafarina 2017	WBLM	ALM		Handgrip, MVIC (handled dynamometer)		Usual walk speed			
Maltais 2016	WBLM	ALMI				6-meter walk	5-time CR	3-meter TUG	
Matsuda 2022		ALM		Handgrip, MVIC (handled dynamometer)	Knee extension, MVIC				
McKenna 2021	WBLM	ALMI		Handgrip, MVIC (handled dynamometer)	Knee extension, MVIC	4-meter walk			
Memelink 2021	WBLM	ALM			10-RM leg press	Usual walk speed	5-time CR		
Meredith 1992	FFM		Thigh muscle CSA		1-RM leg press				
Mertz 2021	WBLM		Thigh muscle CSA		Knee extension, MVIC	400-meter walk			
Midttun 2024	FFM	Leg lean mass					30-second STS	3-meter TUG	
Miller EG 2021	WBLM	ALM	Thigh muscle CSA		1-RM leg press		30-second STS	3-meter TUG	
Miller GD 2006; 2012	FFM					6-minute walk			
Miller MD 2005; 2006			Arm girth		Knee extension, MVIC	Usual walk speed			
Mitchell 2018		Leg lean mass	Thigh muscle CSA		Isokinetic knee extension				
Mogelberg 2022	FFM		Thigh girth	Handgrip, MVIC (handled dynamometer)		6-minute walk			
Mojtahedi 2011	WBLM	Leg lean mass	Thigh volumn		1-RM leg press				
Molnar 2016	FFM			Handgrip, MVIC (handled dynamometer)					SPPB
Mori 2014	FFM			Handgrip, MVIC (handled dynamometer)	1-RM leg press	Usual walk speed		3-meter TUG	
Mori 2018	LBMI	ALM		Handgrip, MVIC (handled dynamometer)	1-RM leg press	Usual walk speed			
Mori 2020	LBMI			Handgrip, MVIC (handled dynamometer)	1-RM leg press	Usual walk speed			
Mori 2021	LBMI			Handgrip, MVIC (handled dynamometer)	1-RM leg press	Usual walk speed			
Mori 2022		ALMI		Handgrip, MVIC (handled dynamometer)	1-RM leg press	Usual walk speed			

To be continued.

Supplementary table S3. Continued.

Study (Author, year)	Muscle mass/volume			Strength		Mobility			
	Whole body	Appendicular (Arm and leg)	Volume	Handgrip	Leg	Walk speed	Chair stand	TUG	Global mobility
Morikawa 2018			Thigh muscle CSA		Knee extension, MVIC				
Moyama 2023	LBMI			Handgrip, MVIC (handled dynamometer)					
Munk 2021							30-second STS		
Murphy 2016	FFM								
Myint 2013			Arm girth						
Nabuco 2018; 2019a	WBLM	ALM			1-RM leg press	10-meter walk	5-time CR		
Nabuco 2019b	WBLM	ALM							
Nabuco 2019c	WBLM				Isokinetic knee extension				
Nakagawa 2024	FFM	ALM		Handgrip, MVIC (handled dynamometer)	Isokinetic knee extension	10-meter walk	30-second STS		
Nakayama 2021			Arm and leg CSA	Handgrip, MVIC (handled dynamometer)					
Nambi 2025	WBLM			Handgrip, MVIC (handled dynamometer)	Knee extension, MVIC	Usual walk speed	5-time CR	3-meter TUG	
Ng 2015					1-RM leg press	6-meter walk			
Ni 2019				Handgrip, MVIC (handled dynamometer)					SPPB
Niccoli 2017				Handgrip, MVIC (handled dynamometer)	Isokinetic knee extension	Usual walk speed		3-meter TUG	
Niitsu 2016					Knee extension, MVIC				
Nilsson 2020	WBLM	ALM		Handgrip, MVIC (handled dynamometer)	1-RM leg press	4-meter walk	5-time CR	3-meter TUG	SPPB
Ninomiya 2023			Thigh muscle thickness	Handgrip, MVIC (handled dynamometer)	Knee extension, MVIC			3-meter TUG	
Oh 2022	WBLM	ALM		Handgrip, MVIC (handled dynamometer)			30-second STS	3-meter TUG	
Oikawa 2018	WBLM	Leg lean mass							
Orsatti 2018	WBLM				1-RM leg press				
Osuka 2017	WBLM	ALM			1-RM leg press		30-second STS		
Pan 2022		ALM	Calf girth	Handgrip, MVIC (handled dynamometer)		4-meter walk	5-time CR	3-meter TUG	SPPB
Park 2023				Handgrip, MVIC (handled dynamometer)		4-meter walk			SPPB
Pedersen LR 2019	FFM								
Pedersen MM 2019				Handgrip, MVIC (handled dynamometer)	Isokinetic knee extension	4-meter walk	30-second STS		
Peng 2024		ALMI		Handgrip, MVIC (handled dynamometer)		6-meter walk	5-time CR		
Puente-Fernandez 2025	FFM		Arm muscle thickness						
Rabadi 2008						2-minute walk			
Reidy 2017; 2020	WBLM	ALM	Thigh muscle CSA		Knee extension, MVIC	6-minute walk		3-meter TUG	
Romera-Liebana 2018				Handgrip, MVIC (handled dynamometer)					SPPB
Rondanelli 2016	FFM	ALMI		Handgrip, MVIC (handled dynamometer)					
Rondanelli 2020	LBMI	ALM		Handgrip, MVIC (handled dynamometer)			5-time CR	3-meter TUG	SPPB
Roschel 2021	WBLM	ALM	Thigh muscle CSA	Handgrip, MVIC (handled dynamometer)	1-RM leg press		5-time CR	3-meter TUG	
Rydwik 2008	FFM				1-RM leg press	Usual walk speed	30-second STS	3-meter TUG	
Sato 2022					Knee extension, MVIC	Usual walk speed			
Seino 2017				Handgrip, MVIC (handled dynamometer)		Usual walk speed		3-meter TUG	
Seino 2018	WBLM	ALM		Handgrip, MVIC (handled dynamometer)	Knee extension, MVIC	Usual walk speed	5-time CR	3-meter TUG	

To be continued.

Supplementary table S3. Continued.

Study (Author, year)	Muscle mass/volume			Strength		Mobility			
	Whole body	Appendicular (Arm and leg)	Volume	Handgrip	Leg	Walk speed	Chair stand	TUG	Global mobility
Serra-Prat 2017				Handgrip, MVIC (handled dynamometer)		Usual walk speed		3-meter TUG	
Shahar 2013	FFM	ALM							
Shenoy 2013					Knee extension, MVIC				
Soares 2023	WBLM			Handgrip, MVIC (handled dynamometer)	1-RM leg press				
Spoelder 2023	WBLM	ALM		Handgrip, MVIC (handled dynamometer)	1-RM leg press				
Sugawara 2010; 2012	FFMI		Arm girth		1-RM leg press	6-minute walk			
Swanenburg 2007	WBLM				Knee extension, MVIC				
Tang 2020				Handgrip, MVIC (handled dynamometer)					SPPB
Tarazona-Santabalbina 2016	WBLM		Arm girth						SPPB
Ten Haaf 2019	WBLM	ALM		Handgrip, MVIC (handled dynamometer)	Knee extension, MVIC	Usual walk speed	5-time CR	3-meter TUG	SPPB
Thomson 2016	WBLM			Handgrip, MVIC (handled dynamometer)	Knee extension, MVIC	6-minute walk			
Dirks 2017; Tieland 2012	WBLM	ALM	Thigh muscle CSA	Handgrip, MVIC (handled dynamometer)	1-RM leg press	Usual walk speed	5-time CR		SPPB
Travers 2023	WBLM			Handgrip, MVIC (handled dynamometer)					
Trevisan 2010	WBLM								
Tsurumi 2022	LBMI		Thigh muscle thickness	Handgrip, MVIC (handled dynamometer)		6-minute walk		3-meter TUG	SPPB
Uchida 2024	WBLM	ALM			Knee extension, MVIC				
Unterberger 2022	WBLM	ALM		Handgrip, MVIC (handled dynamometer)	1-RM leg press	Usual walk speed	30-second STS	3-meter TUG	
van de Bool 2017	WBLM	ALM							
van den Helder 2020		ALM		Handgrip, MVIC (handled dynamometer)		6-meter walk		3-meter TUG	SPPB
van Dongen 2020	WBLM	ALM			Knee extension, MVIC	4-meter walk	5-time CR	3-meter TUG	SPPB
Verceles 2023			Thigh muscle CSA						
Verdijk 2009	WBLM	Leg lean mass	Thigh muscle CSA						
Verreijen 2015	FFM	ALM		Handgrip, MVIC (handled dynamometer)		4-meter walk	5-time CR		
Verreijen 2017	FFM			Handgrip, MVIC (handled dynamometer)		4-meter walk	5-time CR		
Vijayakumaran 2023		ALMI	Calf girth	Handgrip, MVIC (handled dynamometer)			5-time CR		SPPB
Vikberg 2019	WBLM	ALM		Handgrip, MVIC (handled dynamometer)		4-meter walk	5-time CR	3-meter TUG	SPPB
Villanueva 2014	WBLM				1-RM leg press	400-meter walk			
Vorup 2017	WBLM	ALM			Knee extension, MVIC		30-second STS	3-meter TUG	
Wang XJ 2023		ALM				4-meter walk	5-time CR		
Wang ZY 2022	WBLM				1-RM leg press				SPPB
Weinheimer 2012	WBLM	ALM							
Weisgarber 2015		ALM	Thigh muscle thickness		1-RM leg press				
Wu 2018				Handgrip, MVIC (handled dynamometer)		10-meter walk			
Wu 2019		ALMI		Handgrip, MVIC (handled dynamometer)		Usual walk speed		3-meter TUG	
Xie 2021		ALMI		Handgrip, MVIC (handled dynamometer)		Usual walk speed			
Xing 2018									
Xu 2021		ALMI							SPPB

To be continued.

Supplementary table S3. Continued.

Study (Author, year)	Muscle mass/volume			Strength		Mobility			
	Whole body	Appendicular (Arm and leg)	Volume	Handgrip	Leg	Walk speed	Chair stand	TUG	Global mobility
Xue 2023		ALMI		Handgrip, MVIC (handled dynamometer)					
Yamada 2015	LBMI								
Yamada 2019		ALM		Handgrip, MVIC (handled dynamometer)	Knee extension, MVIC	5-meter walk	5-time CR		
Yin 2019		ALMI		Handgrip, MVIC (handled dynamometer)					
Yoshimura 2016			Arm girth	Handgrip, MVIC (handled dynamometer)					
Zak 2009					Knee extension, MVIC	6-minute walk			
Zdzieblik 2015	FFM				Knee extension, MVIC				
Zdzieblik 2021	FFM	ALMI			Knee extension, MVIC				
Zhang 2023a		ALMI		Handgrip, MVIC (handled dynamometer)					
Zhang 2023b		ALMI		Handgrip, MVIC (handled dynamometer)					
Zhao 2022		ALMI		Handgrip, MVIC (handled dynamometer)		6-meter walk		3-meter TUG	
Zhao YF 2021		ALMI		Handgrip, MVIC (handled dynamometer)					SPPB
Zhao YY 2021	LBMI			Handgrip, MVIC (handled dynamometer)					
Zhou 2023		ALMI		Handgrip, MVIC (handled dynamometer)		Usual walk speed			
Zhu 2019		ALM		Handgrip, MVIC (handled dynamometer)	1-RM leg press	Usual walk speed	5-time CR		
Zong 2023	FFMI	ALMI		Handgrip, MVIC (handled dynamometer)		6-minute walk			

1-RM, one repetition maximum; ALM, appendicular lean mass; ALMI, appendicular lean mass index; CR, chair rise; CSA, cross-sectional area; FFM, fat-free mass; FFMI, fat-free mass index; LBMI, lean body mass index; MVIC, maximal voluntary isometric contraction; STS, sit to stand; SPPB, short physical performance battery; WBLM, whole-body lean mass.

Table S4. Direct and network estimates for muscle mass.

	Direct evidence of pairwise meta-analyses (row compared with column)											
	WP+RET	WP+AET	WP+MET	MP+RET	MP+AET	MP+MET	Casein+RET	Casein+MET	SP+RET	SP+AET	SP+MET	Collagen+RET
WP+RET		.	**1.03 (0.06, 1.99)**	0.22 (-0.46, 0.91)	.	.	0.47 (-0.46, 1.39)	.	0.10 (-0.88, 1.09)	.	.	0.09 (-0.88, 1.05)
WP+AET	**0.99 (0.32, 1.65)**		.	.	.	.	.	.	.	0.04 (-1.38, 1.45)	.	.
WP+MET	**0.60 (0.28, 0.92)**	-0.39 (-1.08, 0.30)		.	.	0.80 (-0.48, 2.08)	.	0.53 (-0.32, 1.38)	.	.	.	.
MP+RET	0.16 (-0.13, 0.45)	**-0.83 (-1.53, -0.13)**	**-0.44 (-0.82, -0.06)**		.	0.18 (-1.15, 1.51)	.	.	-0.17 (-0.89, 0.55)	.	.	.
MP+AET	**0.90 (0.24, 1.56)**	-0.08 (-0.85, 0.68)	0.30 (-0.38, 0.99)	**0.74 (0.06, 1.43)**		.	.	.	.	-0.56 (-2.06, 0.94)	.	.
MP+MET	**0.59 (0.20, 0.98)**	-0.40 (-1.12, 0.33)	-0.01 (-0.40, 0.39)	**0.43 (0.01, 0.85)**	-0.31 (-1.02, 0.40)		.	.	.	.	0.04 (-1.29, 1.36)	.
Casein+RET	0.41 (-0.37, 1.20)	-0.57 (-1.59, 0.45)	-0.18 (-1.02, 0.66)	0.26 (-0.57, 1.08)	-0.49 (-1.51, 0.53)	-0.18 (-1.04, 0.69)		.	.	.	.	.
Casein+MET	0.64 (-0.05, 1.34)	-0.34 (-1.26, 0.58)	0.05 (-0.62, 0.71)	0.49 (-0.23, 1.21)	-0.26 (-1.17, 0.66)	0.05 (-0.68, 0.78)	0.23 (-0.81, 1.27)		.	.	.	.
SP+RET	0.21 (-0.15, 0.56)	**-0.78 (-1.50, -0.06)**	-0.39 (-0.81, 0.03)	0.05 (-0.33, 0.44)	-0.69 (-1.40, 0.02)	-0.38 (-0.85, 0.08)	-0.21 (-1.06, 0.64)	-0.44 (-1.18, 0.31)		.	.	.
SP+AET	**0.78 (0.05, 1.50)**	-0.21 (-0.96, 0.53)	0.18 (-0.57, 0.92)	0.62 (-0.13, 1.37)	-0.13 (-0.91, 0.65)	0.19 (-0.59, 0.96)	0.36 (-0.70, 1.42)	0.13 (-0.83, 1.09)	0.57 (-0.20, 1.34)		.	.
SP+MET	**0.58 (0.06, 1.10)**	-0.41 (-1.21, 0.39)	-0.02 (-0.55, 0.51)	0.42 (-0.14, 0.98)	-0.32 (-1.12, 0.47)	-0.01 (-0.57, 0.55)	0.16 (-0.77, 1.10)	-0.07 (-0.87, 0.74)	0.37 (-0.21, 0.95)	-0.20 (-1.04, 0.65)		.
Collagen+RET	0.06 (-0.60, 0.72)	-0.93 (-1.85, 0.00)	-0.54 (-1.25, 0.17)	-0.10 (-0.80, 0.60)	-0.84 (-1.76, 0.07)	-0.53 (-1.27, 0.21)	-0.35 (-1.37, 0.66)	-0.58 (-1.52, 0.36)	-0.15 (-0.87, 0.58)	-0.71 (-1.68, 0.25)	-0.52 (-1.34, 0.30)	
Collagen+AET	0.50 (-0.68, 1.69)	-0.48 (-1.68, 0.72)	-0.09 (-1.30, 1.11)	0.35 (-0.86, 1.55)	-0.40 (-1.67, 0.88)	-0.09 (-1.31, 1.14)	0.09 (-1.33, 1.51)	-0.14 (-1.49, 1.21)	0.30 (-0.92, 1.52)	-0.27 (-1.56, 1.01)	-0.07 (-1.35, 1.20)	0.44 (-0.90, 1.79)
Collagen+MET	0.71 (-1.15, 2.56)	-0.28 (-2.24, 1.67)	0.11 (-1.74, 1.95)	0.55 (-1.32, 2.41)	-0.20 (-2.15, 1.75)	0.11 (-1.75, 1.98)	0.29 (-1.72, 2.30)	0.06 (-1.89, 2.01)	0.50 (-1.38, 2.37)	-0.07 (-2.04, 1.90)	0.13 (-1.77, 2.02)	0.64 (-1.32, 2.61)
Meat+RET	0.13 (-0.37, 0.64)	**-0.85 (-1.66, -0.05)**	-0.47 (-1.02, 0.09)	-0.03 (-0.56, 0.51)	-0.77 (-1.57, 0.03)	-0.46 (-1.04, 0.13)	-0.28 (-1.21, 0.64)	-0.51 (-1.34, 0.31)	-0.08 (-0.63, 0.48)	-0.64 (-1.50, 0.21)	-0.45 (-1.13, 0.24)	0.07 (-0.74, 0.88)
Meat+MET	0.83 (-0.10, 1.76)	-0.16 (-1.27, 0.95)	0.23 (-0.70, 1.16)	0.67 (-0.28, 1.62)	-0.08 (-1.18, 1.03)	0.24 (-0.72, 1.19)	0.41 (-0.80, 1.62)	0.18 (-0.93, 1.29)	0.62 (-0.35, 1.58)	0.05 (-1.09, 1.20)	0.25 (-0.77, 1.27)	0.77 (-0.36, 1.89)
DP+RET	**0.34 (0.02, 0.66)**	-0.65 (-1.35, 0.05)	-0.26 (-0.64, 0.12)	0.18 (-0.20, 0.56)	-0.57 (-1.25, 0.12)	-0.25 (-0.69, 0.18)	-0.08 (-0.92, 0.76)	-0.31 (-1.03, 0.41)	0.13 (-0.30, 0.55)	-0.44 (-1.19, 0.31)	-0.24 (-0.80, 0.32)	0.27 (-0.43, 0.98)
DP+AET	**1.06 (0.50, 1.63)**	0.08 (-0.74, 0.89)	0.46 (-0.16, 1.08)	**0.90 (0.29, 1.52)**	0.16 (-0.66, 0.98)	0.47 (-0.19, 1.13)	0.65 (-0.31, 1.61)	0.42 (-0.45, 1.29)	**0.85 (0.21, 1.50)**	0.29 (-0.57, 1.15)	0.48 (-0.26, 1.23)	**1.00 (0.14, 1.86)**
DP+MET	**0.92 (0.57, 1.26)**	-0.07 (-0.77, 0.63)	0.32 (-0.04, 0.67)	**0.76 (0.36, 1.15)**	0.01 (-0.68, 0.70)	0.32 (-0.08, 0.73)	0.50 (-0.35, 1.35)	0.27 (-0.41, 0.95)	**0.71 (0.27, 1.14)**	0.14 (-0.61, 0.89)	0.34 (-0.21, 0.88)	**0.85 (0.13, 1.58)**
Rice+RET	**0.82 (0.03, 1.62)**	-0.17 (-1.18, 0.84)	0.22 (-0.60, 1.04)	0.66 (-0.14, 1.46)	-0.08 (-1.08, 0.92)	0.23 (-0.57, 1.03)	0.41 (-0.70, 1.52)	0.18 (-0.84, 1.20)	0.61 (-0.20, 1.43)	0.05 (-1.00, 1.09)	0.24 (-0.67, 1.15)	0.76 (-0.26, 1.78)
Rice+MET	1.25 (-0.13, 2.63)	0.26 (-1.25, 1.77)	0.65 (-0.69, 2.00)	1.09 (-0.30, 2.49)	0.35 (-1.16, 1.86)	0.66 (-0.74, 2.06)	0.84 (-0.75, 2.42)	0.61 (-0.89, 2.10)	1.04 (-0.37, 2.45)	0.48 (-1.06, 2.01)	0.67 (-0.77, 2.12)	1.19 (-0.33, 2.71)
Ins-PS+AET	0.46 (-0.86, 1.79)	-0.52 (-1.76, 0.72)	-0.13 (-1.47, 1.20)	0.31 (-1.03, 1.64)	-0.44 (-1.80, 0.92)	-0.13 (-1.48, 1.22)	0.05 (-1.48, 1.58)	-0.18 (-1.65, 1.29)	0.26 (-1.09, 1.60)	-0.31 (-1.67, 1.05)	-0.11 (-1.51, 1.28)	0.40 (-1.06, 1.87)
Oat+MET	1.05 (-0.45, 2.55)	0.06 (-1.56, 1.68)	0.45 (-1.02, 1.92)	0.89 (-0.63, 2.41)	0.15 (-1.47, 1.77)	0.46 (-1.06, 1.98)	0.63 (-1.06, 2.32)	0.40 (-1.21, 2.01)	0.84 (-0.69, 2.37)	0.27 (-1.37, 1.92)	0.47 (-1.09, 2.03)	0.99 (-0.64, 2.62)
WP	**1.02 (0.75, 1.29)**	0.03 (-0.64, 0.70)	**0.42 (0.10, 0.74)**	**0.86 (0.51, 1.21)**	0.11 (-0.55, 0.78)	**0.43 (0.02, 0.83)**	0.60 (-0.22, 1.42)	0.37 (-0.32, 1.06)	**0.81 (0.41, 1.20)**	0.24 (-0.49, 0.97)	0.44 (-0.10, 0.97)	**0.96 (0.26, 1.65)**
MP	**0.94 (0.50, 1.39)**	-0.04 (-0.79, 0.70)	0.34 (-0.13, 0.82)	**0.79 (0.33, 1.24)**	0.04 (-0.68, 0.77)	0.35 (-0.12, 0.83)	0.53 (-0.36, 1.42)	0.30 (-0.48, 1.08)	**0.74 (0.23, 1.24)**	0.17 (-0.61, 0.95)	0.36 (-0.26, 0.99)	**0.88 (0.11, 1.66)**
Casein	1.02 (-0.49, 2.53)	0.03 (-1.60, 1.66)	0.42 (-1.10, 1.94)	0.86 (-0.67, 2.38)	0.11 (-1.51, 1.74)	0.43 (-1.11, 1.97)	0.60 (-1.09, 2.30)	0.37 (-1.27, 2.01)	0.81 (-0.73, 2.34)	0.24 (-1.41, 1.90)	0.44 (-1.14, 2.02)	0.96 (-0.68, 2.59)
SP	**1.08 (0.67, 1.48)**	0.09 (-0.64, 0.82)	**0.48 (0.03, 0.93)**	**0.92 (0.48, 1.36)**	0.18 (-0.54, 0.89)	0.49 (-0.01, 0.98)	0.66 (-0.21, 1.54)	0.43 (-0.33, 1.19)	**0.87 (0.43, 1.31)**	0.30 (-0.47, 1.07)	0.50 (-0.08, 1.08)	**1.02 (0.26, 1.77)**
Collagen	**0.87 (0.06, 1.68)**	-0.12 (-1.05, 0.81)	0.27 (-0.57, 1.10)	0.71 (-0.13, 1.55)	-0.03 (-1.01, 0.94)	0.28 (-0.59, 1.14)	0.45 (-0.67, 1.58)	0.22 (-0.81, 1.26)	0.66 (-0.20, 1.52)	0.09 (-0.91, 1.10)	0.29 (-0.64, 1.22)	0.81 (-0.23, 1.84)
Meat	**0.80 (0.15, 1.45)**	-0.19 (-1.08, 0.71)	0.20 (-0.47, 0.87)	0.64 (-0.03, 1.32)	-0.10 (-0.99, 0.78)	0.21 (-0.50, 0.92)	0.38 (-0.62, 1.39)	0.15 (-0.76, 1.06)	0.59 (-0.10, 1.28)	0.02 (-0.91, 0.96)	0.22 (-0.57, 1.01)	0.74 (-0.17, 1.64)
DP	**1.07 (0.65, 1.48)**	0.08 (-0.64, 0.80)	**0.47 (0.03, 0.91)**	**0.91 (0.45, 1.37)**	0.16 (-0.55, 0.88)	0.48 (-0.01, 0.97)	0.65 (-0.23, 1.53)	0.42 (-0.33, 1.17)	**0.86 (0.36, 1.35)**	0.29 (-0.48, 1.06)	0.49 (-0.12, 1.09)	**1.01 (0.25, 1.76)**
RET	**0.67 (0.50, 0.85)**	-0.31 (-0.98, 0.35)	0.07 (-0.24, 0.39)	**0.51 (0.25, 0.78)**	-0.23 (-0.88, 0.43)	0.08 (-0.30, 0.46)	0.26 (-0.53, 1.05)	0.03 (-0.66, 0.72)	**0.46 (0.13, 0.80)**	-0.10 (-0.82, 0.62)	0.09 (-0.42, 0.61)	0.61 (-0.04, 1.26)
AET	**1.01 (0.51, 1.52)**	0.03 (-0.50, 0.56)	0.42 (-0.13, 0.96)	**0.86 (0.31, 1.40)**	0.11 (-0.47, 0.69)	0.42 (-0.16, 1.00)	0.60 (-0.33, 1.53)	0.37 (-0.45, 1.19)	**0.81 (0.23, 1.38)**	0.24 (-0.35, 0.83)	0.44 (-0.24, 1.11)	**0.95 (0.14, 1.77)**
MET	**1.06 (0.76, 1.37)**	0.08 (-0.60, 0.76)	**0.47 (0.22, 0.72)**	**0.91 (0.54, 1.27)**	0.16 (-0.51, 0.83)	**0.47 (0.11, 0.84)**	0.65 (-0.18, 1.48)	0.42 (-0.24, 1.08)	**0.86 (0.45, 1.26)**	0.29 (-0.45, 1.02)	0.49 (-0.01, 0.98)	**1.00 (0.30, 1.71)**
RC	**1.29 (1.08, 1.51)**	0.31 (-0.34, 0.95)	**0.70 (0.43, 0.96)**	**1.14 (0.84, 1.43)**	0.39 (-0.24, 1.03)	**0.70 (0.36, 1.05)**	**0.88 (0.07, 1.68)**	0.65 (-0.02, 1.32)	**1.09 (0.74, 1.43)**	0.52 (-0.18, 1.22)	**0.72 (0.23, 1.20)**	**1.23 (0.56, 1.90)**

Relative effects of NMA (column compared with row)

To be continued.

Table S4. Continued.

	Direct evidence of pairwise meta-analyses (row compared with column)											
	Collagen+AET	Collagen+MET	Meat+RET	Meat+MET	DP+RET	DP+AET	DP+MET	Rice+RET	Rice+MET	Ins-PS+AET	Oat+MET	WP
WP+RET	.	.	.	.	-0.04 (-1.36, 1.29)	**1.41 (0.63, 2.19)**	.	.	.	.	.	**0.87 (0.52, 1.23)**
WP+AET	0.99 (-0.43, 2.41)	.	.	.	.	.	.	.	.	-0.62 (-2.01, 0.76)	.	**2.45 (1.00, 3.90)**
WP+MET	.	.	.	.	**-1.71 (-3.06, -0.36)**	.	0.86 (-0.70, 2.42)	.	0.65 (-0.69, 2.00)	.	0.45 (-1.02, 1.92)	0.33 (-0.14, 0.80)
MP+RET	.	.	.	.	.	.	.	0.29 (-1.25, 1.83)	.	.	.	.
MP+AET	.	.	.	.	.	.	.	.	.	.	.	.
MP+MET	.	.	.	.	.	.	0.67 (-0.25, 1.60)	0.40 (-0.85, 1.65)	.	.	.	.
Casein+RET	.	.	.	.	.	.	.	.	.	.	.	.
Casein+MET	.	.	.	.	.	.	1.27 (-0.03, 2.57)	.	.	.	.	0.32 (-1.20, 1.85)
SP+RET	.	.	-0.64 (-2.15, 0.87)	.	.	.	.	0.96 (-0.60, 2.52)	.	.	.	0.22 (-1.21, 1.66)
SP+AET	.	.	.	.	.	.	.	.	.	.	.	.
SP+MET	.	.	.	.	.	.	.	.	.	.	.	.
Collagen+RET	.	.	.	.	.	.	.	.	.	.	.	.
Collagen+AET		.	.	.	.	.	.	.	.	.	.	**1.46 (0.03, 2.89)**
Collagen+MET	0.20 (-1.99, 2.39)		.	.	.	.	.	.	.	.	.	.
Meat+RET	-0.37 (-1.65, 0.90)	-0.57 (-2.48, 1.34)		.	-0.51 (-2.00, 0.98)	.	.	0.53 (-0.77, 1.82)	.	.	.	.
Meat+MET	0.32 (-1.16, 1.81)	0.12 (-1.93, 2.17)	0.70 (-0.33, 1.72)		.	.	0.27 (-0.99, 1.54)	.	.	.	.	.
DP+RET	-0.17 (-1.38, 1.04)	-0.37 (-2.24, 1.50)	0.20 (-0.34, 0.75)	-0.49 (-1.44, 0.46)		0.84 (-0.49, 2.18)	0.58 (-0.69, 1.84)	.	.	.	.	.
DP+AET	0.56 (-0.73, 1.84)	0.36 (-1.57, 2.29)	**0.93 (0.19, 1.67)**	0.24 (-0.83, 1.30)	**0.73 (0.12, 1.33)**		.	.	.	.	.	.
DP+MET	0.41 (-0.80, 1.62)	0.21 (-1.65, 2.07)	**0.78 (0.22, 1.35)**	0.09 (-0.82, 0.99)	**0.58 (0.19, 0.97)**	-0.15 (-0.78, 0.48)		.	.	.	.	.
Rice+RET	0.32 (-1.09, 1.73)	0.12 (-1.89, 2.12)	0.69 (-0.13, 1.51)	-0.01 (-1.20, 1.19)	0.48 (-0.34, 1.31)	-0.24 (-1.20, 0.72)	-0.09 (-0.92, 0.73)		.	.	.	.
Rice+MET	0.75 (-1.06, 2.55)	0.55 (-1.74, 2.83)	1.12 (-0.33, 2.57)	0.42 (-1.21, 2.06)	0.91 (-0.48, 2.31)	0.19 (-1.29, 1.67)	0.34 (-1.05, 1.73)	0.43 (-1.14, 2.00)		.	.	.
Ins-PS+AET	-0.04 (-1.71, 1.63)	-0.24 (-2.50, 2.02)	0.33 (-1.07, 1.73)	-0.36 (-1.95, 1.23)	0.13 (-1.21, 1.46)	-0.60 (-2.00, 0.80)	-0.45 (-1.79, 0.89)	-0.36 (-1.88, 1.17)	-0.79 (-2.68, 1.11)		.	.
Oat+MET	0.54 (-1.35, 2.44)	0.34 (-2.02, 2.70)	0.92 (-0.65, 2.49)	0.22 (-1.52, 1.96)	0.71 (-0.80, 2.23)	-0.01 (-1.61, 1.58)	0.13 (-1.38, 1.64)	0.23 (-1.45, 1.91)	-0.20 (-2.19, 1.79)	0.58 (-1.40, 2.57)		.
WP	0.51 (-0.67, 1.69)	0.31 (-1.55, 2.17)	**0.88 (0.35, 1.42)**	0.19 (-0.75, 1.12)	**0.68 (0.32, 1.04)**	-0.05 (-0.65, 0.56)	0.10 (-0.26, 0.47)	0.20 (-0.62, 1.01)	-0.23 (-1.62, 1.15)	0.55 (-0.77, 1.88)	-0.03 (-1.53, 1.47)	
MP	0.44 (-0.80, 1.68)	0.24 (-1.65, 2.13)	**0.81 (0.18, 1.44)**	0.12 (-0.87, 1.11)	**0.61 (0.12, 1.10)**	-0.12 (-0.81, 0.57)	0.03 (-0.46, 0.52)	0.12 (-0.74, 0.99)	-0.31 (-1.73, 1.12)	0.48 (-0.88, 1.84)	-0.10 (-1.65, 1.44)	-0.07 (-0.54, 0.40)
Casein	0.51 (-1.38, 2.41)	0.31 (-2.07, 2.69)	0.88 (-0.69, 2.46)	0.19 (-1.57, 1.94)	0.68 (-0.85, 2.21)	-0.05 (-1.65, 1.56)	0.10 (-1.43, 1.63)	0.20 (-1.50, 1.89)	-0.23 (-2.26, 1.79)	0.55 (-1.44, 2.54)	-0.03 (-2.14, 2.08)	0.00 (-1.48, 1.48)
SP	0.57 (-0.65, 1.80)	0.37 (-1.51, 2.25)	**0.95 (0.35, 1.54)**	0.25 (-0.72, 1.23)	**0.74 (0.28, 1.20)**	0.02 (-0.65, 0.68)	0.16 (-0.30, 0.63)	0.26 (-0.60, 1.11)	-0.17 (-1.59, 1.24)	0.61 (-0.74, 1.97)	0.03 (-1.51, 1.56)	0.06 (-0.37, 0.49)
Collagen	0.36 (-0.91, 1.64)	0.16 (-1.85, 2.17)	0.74 (-0.20, 1.67)	0.04 (-1.17, 1.25)	0.53 (-0.31, 1.38)	-0.19 (-1.16, 0.77)	-0.05 (-0.89, 0.80)	0.05 (-1.07, 1.16)	-0.38 (-1.97, 1.20)	0.40 (-1.08, 1.88)	-0.18 (-1.87, 1.51)	-0.15 (-0.95, 0.66)
Meat	0.29 (-1.04, 1.63)	0.09 (-1.85, 2.04)	0.67 (-0.06, 1.40)	-0.03 (-1.06, 1.00)	0.46 (-0.22, 1.14)	-0.26 (-1.10, 0.58)	-0.12 (-0.80, 0.56)	-0.02 (-1.00, 0.96)	-0.45 (-1.95, 1.05)	0.33 (-1.12, 1.79)	-0.25 (-1.86, 1.37)	-0.22 (-0.88, 0.45)
DP	0.56 (-0.66, 1.79)	0.36 (-1.52, 2.24)	**0.94 (0.32, 1.55)**	0.24 (-0.73, 1.21)	**0.73 (0.30, 1.16)**	0.00 (-0.66, 0.67)	0.15 (-0.27, 0.57)	0.25 (-0.62, 1.11)	-0.18 (-1.60, 1.23)	0.60 (-0.75, 1.95)	0.02 (-1.51, 1.55)	0.05 (-0.38, 0.48)
RET	0.17 (-1.02, 1.36)	-0.03 (-1.89, 1.82)	**0.54 (0.06, 1.02)**	-0.15 (-1.08, 0.77)	**0.34 (0.04, 0.64)**	-0.39 (-0.96, 0.18)	-0.24 (-0.58, 0.09)	-0.15 (-0.93, 0.64)	-0.58 (-1.96, 0.80)	0.21 (-1.11, 1.53)	-0.38 (-1.88, 1.13)	**-0.34 (-0.61, -0.07)**
AET	0.51 (-0.66, 1.68)	0.31 (-1.60, 2.22)	**0.88 (0.20, 1.57)**	0.19 (-0.84, 1.21)	**0.68 (0.13, 1.23)**	-0.05 (-0.73, 0.63)	0.10 (-0.45, 0.65)	0.19 (-0.72, 1.11)	-0.24 (-1.68, 1.21)	0.55 (-0.69, 1.79)	-0.03 (-1.60, 1.53)	-0.00 (-0.52, 0.52)
MET	0.56 (-0.64, 1.76)	0.36 (-1.47, 2.19)	**0.93 (0.39, 1.47)**	0.24 (-0.68, 1.15)	**0.73 (0.37, 1.09)**	0.00 (-0.61, 0.61)	0.15 (-0.17, 0.47)	0.24 (-0.56, 1.05)	-0.19 (-1.55, 1.18)	0.60 (-0.73, 1.93)	0.02 (-1.47, 1.51)	0.05 (-0.26, 0.36)
RC	0.79 (-0.39, 1.97)	0.59 (-1.26, 2.44)	**1.16 (0.66, 1.66)**	0.47 (-0.44, 1.38)	**0.96 (0.67, 1.25)**	0.23 (-0.34, 0.80)	**0.38 (0.09, 0.67)**	0.47 (-0.31, 1.26)	0.04 (-1.33, 1.41)	0.83 (-0.48, 2.14)	0.25 (-1.25, 1.74)	**0.28 (0.02, 0.53)**
	Relative effects of NMA (column compared with row)											

To be continued.

Table S4. Continued.

	Direct evidence of pairwise meta-analyses (row compared with column)									
	MP	Casein	SP	Collagen	Meat	DP	RET	AET	MET	RC
WP+RET	.	.	0.16 (-1.28, 1.60)	0.04 (-1.24, 1.33)	.	1.36 (-0.15, 2.87)	**0.61 (0.41, 0.81)**	.	.	**1.31 (0.96, 1.65)**
WP+AET	.	.	.	**1.60 (0.17, 3.03)**	.	.	.	0.04 (-0.51, 0.60)	.	**-2.41 (-3.72, -1.11)**
WP+MET	.	.	.	.	.	.	.	.	**0.38 (0.09, 0.66)**	**0.80 (0.40, 1.20)**
MP+RET	**0.82 (0.11, 1.53)**	.	0.74 (-0.76, 2.24)	.	.	.	**0.65 (0.30, 1.01)**	.	.	**0.90 (0.27, 1.52)**
MP+AET	-1.11 (-2.61, 0.39)	.	**-1.78 (-3.33, -0.23)**	.	.	.	.	0.15 (-0.51, 0.82)	.	**1.26 (0.32, 2.20)**
MP+MET	0.27 (-0.49, 1.03)	.	.	.	.	.	.	.	0.41 (-0.15, 0.98)	**0.62 (0.08, 1.15)**
Casein+RET	.	.	.	.	.	.	0.39 (-1.06, 1.84)	.	.	.
Casein+MET	.	.	.	.	.	.	.	.	-0.05 (-1.09, 0.99)	0.75 (-0.79, 2.29)
SP+RET	0.54 (-0.92, 2.00)	.	**0.95 (0.41, 1.48)**	.	.	.	**0.54 (0.12, 0.97)**	.	.	**0.95 (0.42, 1.47)**
SP+AET	-0.55 (-2.02, 0.93)	.	-1.22 (-2.74, 0.30)	.	.	.	.	0.44 (-0.24, 1.13)	.	.
SP+MET	.	.	0.45 (-0.57, 1.47)	.	.	.	.	.	0.51 (-0.12, 1.14)	**0.65 (0.03, 1.26)**
Collagen+RET	.	.	.	.	.	.	0.54 (-0.26, 1.33)	.	.	**2.08 (0.47, 3.70)**
Collagen+AET	.	.	.	0.61 (-0.81, 2.03)	.	.	.	0.87 (-0.50, 2.25)	.	.
Collagen+MET	.	.	.	.	.	.	.	.	0.36 (-1.47, 2.19)	.
Meat+RET	.	.	.	.	0.69 (-0.28, 1.67)	.	**0.58 (0.01, 1.16)**	.	.	**1.06 (0.08, 2.04)**
Meat+MET	.	.	.	.	-0.00 (-1.51, 1.51)	.	.	.	0.06 (-1.48, 1.59)	0.06 (-1.50, 1.61)
DP+RET	.	.	.	.	.	0.35 (-0.40, 1.10)	0.31 (-0.12, 0.74)	.	.	**0.84 (0.44, 1.24)**
DP+AET	.	.	.	.	.	.	.	0.38 (-0.94, 1.69)	.	0.96 (-0.40, 2.31)
DP+MET	.	.	.	.	.	0.44 (-0.17, 1.04)	.	.	0.45 (-0.02, 0.92)	0.30 (-0.06, 0.66)
Rice+RET	.	.	.	.	.	.	.	.	.	.
Rice+MET	.	.	.	.	.	.	.	.	.	.
Ins-PS+AET	.	.	.	.	.	.	.	0.45 (-0.94, 1.84)	.	.
Oat+MET	.	.	.	.	.	.	.	.	.	.
WP	.	-0.00 (-1.48, 1.48)	-0.16 (-1.60, 1.28)	-0.56 (-1.52, 0.39)	.	-0.70 (-2.13, 0.72)	-0.14 (-0.57, 0.30)	-0.59 (-1.95, 0.78)	-0.14 (-0.67, 0.39)	**0.35 (0.02, 0.69)**
MP		.	-0.24 (-1.29, 0.82)	.	.	0.22 (-1.09, 1.52)	-0.40 (-1.70, 0.89)	.	-0.10 (-1.42, 1.22)	0.27 (-0.40, 0.93)
Casein	0.07 (-1.48, 1.63)		.	.	.	.	.	.	.	.
SP	0.13 (-0.39, 0.66)	0.06 (-1.48, 1.61)		.	-0.65 (-2.15, 0.86)	.	**-0.74 (-1.37, -0.12)**	.	-0.36 (-1.38, 0.66)	-0.01 (-0.54, 0.51)
Collagen	-0.08 (-0.97, 0.82)	-0.15 (-1.84, 1.54)	-0.21 (-1.09, 0.67)		.	.	.	0.26 (-1.11, 1.63)	.	0.09 (-1.21, 1.39)
Meat	-0.14 (-0.89, 0.60)	-0.22 (-1.84, 1.41)	-0.28 (-0.97, 0.41)	-0.07 (-1.08, 0.94)		.	-0.01 (-0.98, 0.96)	.	0.06 (-1.48, 1.59)	0.28 (-0.54, 1.10)
DP	0.12 (-0.41, 0.66)	0.05 (-1.49, 1.60)	-0.01 (-0.53, 0.51)	0.20 (-0.67, 1.07)	0.27 (-0.46, 0.99)		-0.10 (-1.45, 1.26)	-0.89 (-2.21, 0.43)	0.65 (-0.15, 1.45)	0.32 (-0.29, 0.94)
RET	-0.27 (-0.70, 0.16)	-0.34 (-1.85, 1.17)	**-0.40 (-0.79, -0.01)**	-0.19 (-1.01, 0.62)	-0.13 (-0.76, 0.51)	-0.39 (-0.80, 0.01)		.	.	**0.81 (0.53, 1.09)**
AET	0.07 (-0.54, 0.68)	-0.00 (-1.57, 1.57)	-0.06 (-0.65, 0.53)	0.15 (-0.71, 1.00)	0.22 (-0.57, 1.00)	-0.05 (-0.63, 0.52)	0.34 (-0.16, 0.85)		.	0.43 (-0.31, 1.16)
MET	0.12 (-0.34, 0.58)	0.05 (-1.47, 1.56)	-0.01 (-0.44, 0.42)	0.20 (-0.63, 1.03)	0.27 (-0.39, 0.92)	-0.00 (-0.42, 0.41)	**0.39 (0.10, 0.69)**	0.05 (-0.48, 0.58)		0.24 (-0.08, 0.57)
RC	0.35 (-0.07, 0.77)	0.28 (-1.23, 1.78)	0.22 (-0.16, 0.60)	0.43 (-0.38, 1.23)	0.50 (-0.14, 1.13)	0.23 (-0.15, 0.61)	**0.62 (0.42, 0.82)**	0.28 (-0.20, 0.76)	0.23 (-0.01, 0.47)	
	Relative effects of NMA (column compared with row)									

Pairwise (upper right portion) and network (lower left portion) meta-analysis results are presented for mean change (from baseline) in global function. Effect estimation is presented in standardized mean difference with 95% confidence interval. Significant results ($p < 0.05$) are marked in bold.

AET, aerobic exercise training; DP, dietary protein; Ins-PS, insect protein supplement; MET, multicomponent exercise training; MP, milk protein; RET, resistance exercise training; SP, soy protein; WP, whey protein; RC, regular care.

Table S5. Direct and network estimates for handgrip strength.

	Direct evidence of pairwise meta-analyses (row compared with column)									
	WP+RET	WP+AET	WP+MET	MP+RET	MP+AET	MP+MET	Casein+RET	Casein+MET	SP+RET	SP+AET
WP+RET		.	-0.19 (-1.23, 0.84)	0.07 (-0.71, 0.85)	.	.	0.05 (-1.02, 1.12)	.	0.19 (-0.97, 1.34)	.
WP+AET	0.46 (-0.39, 1.31)		.	.	.	.	.	.	.	0.48 (-0.75, 1.70)
WP+MET	0.20 (-0.12, 0.52)	-0.26 (-1.13, 0.61)		.	.	**0.94 (0.15, 1.73)**	.	0.88 (-0.30, 2.05)	.	.
MP+RET	0.21 (-0.09, 0.52)	-0.25 (-1.13, 0.64)	0.02 (-0.37, 0.40)		.	.	.	.	-0.18 (-1.29, 0.93)	.
MP+AET	0.29 (-0.40, 0.98)	-0.17 (-1.04, 0.70)	0.09 (-0.62, 0.81)	0.08 (-0.65, 0.80)		.	.	.	.	.
MP+MET	**0.60 (0.22, 0.99)**	0.14 (-0.76, 1.04)	**0.41 (0.04, 0.77)**	0.39 (-0.04, 0.82)	0.31 (-0.43, 1.06)		.	.	.	.
Casein+RET	0.32 (-0.46, 1.10)	-0.14 (-1.28, 1.01)	0.12 (-0.69, 0.94)	0.11 (-0.72, 0.93)	0.03 (-1.00, 1.06)	-0.28 (-1.13, 0.56)		.	.	.
Casein+MET	**0.79 (0.15, 1.42)**	0.33 (-0.70, 1.36)	0.59 (-0.02, 1.20)	0.57 (-0.09, 1.24)	0.50 (-0.40, 1.39)	0.18 (-0.48, 0.84)	0.46 (-0.52, 1.45)		.	.
SP+RET	-0.08 (-0.56, 0.39)	-0.54 (-1.50, 0.41)	-0.28 (-0.81, 0.24)	-0.30 (-0.81, 0.21)	-0.38 (-1.19, 0.44)	**-0.69 (-1.26, -0.12)**	-0.41 (-1.31, 0.50)	**-0.87 (-1.63, -0.11)**		.
SP+AET	0.68 (-0.46, 1.82)	0.22 (-0.64, 1.08)	0.48 (-0.67, 1.64)	0.47 (-0.70, 1.63)	0.39 (-0.73, 1.51)	0.08 (-1.10, 1.25)	0.36 (-1.01, 1.73)	-0.11 (-1.38, 1.17)	0.76 (-0.45, 1.98)	
SP+MET	0.45 (-0.16, 1.07)	-0.01 (-1.03, 1.02)	0.26 (-0.36, 0.87)	0.24 (-0.41, 0.89)	0.16 (-0.73, 1.05)	-0.15 (-0.80, 0.50)	0.13 (-0.84, 1.11)	-0.33 (-1.15, 0.48)	0.54 (-0.20, 1.28)	-0.23 (-1.50, 1.05)
Collagen+RET	0.11 (-1.10, 1.32)	-0.35 (-1.83, 1.13)	-0.09 (-1.33, 1.16)	-0.10 (-1.35, 1.14)	-0.18 (-1.57, 1.21)	-0.49 (-1.76, 0.78)	-0.21 (-1.65, 1.23)	-0.67 (-2.04, 0.69)	0.20 (-1.10, 1.50)	-0.57 (-2.23, 1.09)
Collagen+MET	0.78 (-0.93, 2.48)	0.32 (-1.57, 2.20)	0.58 (-1.12, 2.27)	0.56 (-1.16, 2.28)	0.48 (-1.34, 2.30)	0.17 (-1.54, 1.88)	0.45 (-1.41, 2.32)	-0.01 (-1.79, 1.77)	0.86 (-0.90, 2.61)	0.10 (-1.94, 2.13)
Meat+RET	0.10 (-0.70, 0.90)	-0.36 (-1.51, 0.80)	-0.10 (-0.93, 0.74)	-0.11 (-0.94, 0.71)	-0.19 (-1.22, 0.85)	-0.50 (-1.37, 0.36)	-0.22 (-1.33, 0.89)	-0.68 (-1.68, 0.32)	0.19 (-0.72, 1.09)	-0.58 (-1.96, 0.80)
Meat+MET	**1.31 (0.10, 2.51)**	0.85 (-0.61, 2.30)	1.11 (-0.09, 2.31)	1.09 (-0.13, 2.31)	1.01 (-0.35, 2.38)	0.70 (-0.52, 1.92)	0.98 (-0.44, 2.40)	0.52 (-0.80, 1.84)	**1.39 (0.12, 2.66)**	0.63 (-1.01, 2.26)
DP+RET	0.12 (-0.19, 0.43)	-0.34 (-1.21, 0.53)	-0.08 (-0.43, 0.28)	-0.09 (-0.47, 0.28)	-0.17 (-0.89, 0.55)	**-0.48 (-0.89, -0.07)**	-0.20 (-1.02, 0.62)	**-0.66 (-1.31, -0.02)**	0.21 (-0.32, 0.73)	-0.56 (-1.71, 0.60)
DP+AET	**0.98 (0.45, 1.51)**	0.52 (-0.36, 1.40)	**0.78 (0.22, 1.34)**	**0.77 (0.19, 1.34)**	0.69 (-0.12, 1.50)	0.38 (-0.22, 0.97)	0.66 (-0.27, 1.58)	0.19 (-0.59, 0.97)	**1.06 (0.38, 1.75)**	0.30 (-0.87, 1.47)
DP+MET	**0.65 (0.32, 0.97)**	0.19 (-0.69, 1.06)	**0.45 (0.12, 0.78)**	**0.43 (0.04, 0.82)**	0.35 (-0.37, 1.07)	0.04 (-0.33, 0.42)	0.32 (-0.50, 1.14)	-0.14 (-0.75, 0.47)	**0.73 (0.20, 1.26)**	-0.03 (-1.19, 1.12)
Rice+MET	0.46 (-0.36, 1.27)	-0.00 (-1.16, 1.15)	0.26 (-0.53, 1.04)	0.24 (-0.60, 1.08)	0.16 (-0.88, 1.20)	-0.15 (-0.93, 0.63)	0.13 (-0.98, 1.25)	-0.33 (-1.31, 0.64)	0.54 (-0.38, 1.46)	-0.22 (-1.60, 1.16)
Ins-PS+AET	0.24 (-0.86, 1.35)	-0.22 (-0.99, 0.56)	0.05 (-1.07, 1.17)	0.03 (-1.10, 1.16)	-0.05 (-1.14, 1.05)	-0.36 (-1.50, 0.78)	-0.08 (-1.42, 1.26)	-0.54 (-1.79, 0.70)	0.33 (-0.86, 1.51)	-0.44 (-1.50, 0.63)
WP	**0.61 (0.33, 0.89)**	0.15 (-0.69, 0.99)	**0.41 (0.09, 0.74)**	**0.40 (0.03, 0.76)**	0.32 (-0.36, 1.00)	0.01 (-0.40, 0.41)	0.29 (-0.52, 1.10)	-0.18 (-0.82, 0.46)	**0.69 (0.18, 1.21)**	-0.07 (-1.20, 1.06)
MP	**0.83 (0.33, 1.34)**	0.37 (-0.59, 1.33)	**0.63 (0.13, 1.14)**	**0.62 (0.09, 1.15)**	0.54 (-0.28, 1.36)	0.23 (-0.22, 0.68)	0.51 (-0.40, 1.42)	0.05 (-0.70, 0.79)	**0.92 (0.26, 1.57)**	0.15 (-1.07, 1.37)
Casein	0.77 (-0.17, 1.71)	0.31 (-0.93, 1.55)	0.57 (-0.36, 1.50)	0.55 (-0.41, 1.51)	0.48 (-0.66, 1.61)	0.16 (-0.80, 1.12)	0.45 (-0.76, 1.65)	-0.02 (-0.99, 0.95)	0.85 (-0.18, 1.88)	0.09 (-1.37, 1.54)
SP	**0.70 (0.06, 1.34)**	0.24 (-0.80, 1.28)	0.50 (-0.16, 1.16)	0.48 (-0.18, 1.15)	0.41 (-0.50, 1.32)	0.09 (-0.60, 0.79)	0.38 (-0.61, 1.37)	-0.09 (-0.94, 0.77)	**0.78 (0.09, 1.47)**	0.02 (-1.27, 1.30)
Meat	**1.31 (0.10, 2.51)**	0.85 (-0.61, 2.30)	1.11 (-0.09, 2.31)	1.09 (-0.13, 2.31)	1.01 (-0.35, 2.38)	0.70 (-0.52, 1.92)	0.98 (-0.44, 2.40)	0.52 (-0.80, 1.84)	**1.39 (0.12, 2.66)**	0.63 (-1.01, 2.26)
DP	**1.13 (0.70, 1.56)**	0.67 (-0.25, 1.59)	**0.93 (0.48, 1.39)**	**0.92 (0.44, 1.40)**	**0.84 (0.06, 1.62)**	**0.53 (0.03, 1.02)**	0.81 (-0.06, 1.68)	0.35 (-0.36, 1.05)	**1.22 (0.61, 1.82)**	0.45 (-0.74, 1.64)
RET	**0.36 (0.16, 0.56)**	-0.10 (-0.95, 0.75)	0.16 (-0.15, 0.47)	0.14 (-0.13, 0.41)	0.07 (-0.62, 0.75)	-0.25 (-0.62, 0.13)	0.04 (-0.76, 0.83)	-0.43 (-1.06, 0.20)	0.44 (-0.02, 0.90)	-0.32 (-1.46, 0.82)
AET	0.51 (-0.38, 1.40)	0.05 (-0.49, 0.59)	0.31 (-0.59, 1.22)	0.30 (-0.62, 1.21)	0.22 (-0.62, 1.06)	-0.09 (-1.03, 0.84)	0.19 (-0.98, 1.36)	-0.28 (-1.33, 0.78)	0.59 (-0.39, 1.58)	-0.17 (-0.94, 0.61)
MET	**0.66 (0.35, 0.96)**	0.20 (-0.67, 1.06)	**0.46 (0.20, 0.72)**	**0.44 (0.07, 0.81)**	0.37 (-0.34, 1.08)	0.05 (-0.30, 0.40)	0.34 (-0.48, 1.15)	-0.13 (-0.72, 0.47)	**0.74 (0.22, 1.26)**	-0.02 (-1.17, 1.13)
RC	**0.86 (0.63, 1.08)**	0.40 (-0.45, 1.24)	**0.66 (0.41, 0.91)**	**0.64 (0.34, 0.95)**	0.57 (-0.11, 1.25)	0.25 (-0.07, 0.58)	0.54 (-0.25, 1.32)	0.07 (-0.53, 0.67)	**0.94 (0.47, 1.41)**	0.18 (-0.96, 1.31)
	Relative effects of NMA (column compared with row)									

To be continued.

Table S5. Continued.

	Direct evidence of pairwise meta-analyses (row compared with column)									
	SP+MET	Collagen+RET	Collagen+MET	Meat+RET	Meat+MET	DP+RET	DP+AET	DP+MET	Rice+MET	Ins-PS+AET
WP+RET	.	0.11 (-1.10, 1.32)	.	.	.	0.25 (-0.80, 1.31)	**2.39 (1.20, 3.58)**	.	.	.
WP+AET	.	.	.	.	.	.	0.25 (-0.91, 1.40)	.	.	-0.18 (-1.01, 0.65)
WP+MET	.	.	.	.	.	0.44 (-0.60, 1.48)	.	.	-0.03 (-1.15, 1.10)	.
MP+RET	.	.	.	.	.	.	.	.	.	.
MP+AET	.	.	.	.	.	.	.	.	.	.
MP+MET	.	.	.	.	.	.	.	0.12 (-0.64, 0.87)	0.08 (-0.94, 1.11)	.
Casein+RET	.	.	.	.	.	.	.	.	.	.
Casein+MET	.	.	.	.	.	.	.	-0.11 (-1.18, 0.95)	.	.
SP+RET	.	.	.	.	.	.	.	.	.	.
SP+AET	.	.	.	.	.	.	.	.	.	.
SP+MET		.	.	.	.	.	.	-0.11 (-1.70, 1.48)	.	.
Collagen+RET	-0.34 (-1.70, 1.01)		.	.	.	.	.	.	.	.
Collagen+MET	0.32 (-1.46, 2.10)	0.66 (-1.43, 2.75)		.	.	.	.	.	.	.
Meat+RET	-0.35 (-1.34, 0.64)	-0.01 (-1.46, 1.44)	-0.67 (-2.55, 1.20)		.	.	.	.	.	.
Meat+MET	0.85 (-0.46, 2.16)	1.19 (-0.51, 2.90)	0.53 (-1.52, 2.58)	1.20 (-0.23, 2.64)		.	.	.	.	.
DP+RET	-0.33 (-0.97, 0.30)	0.01 (-1.24, 1.26)	-0.65 (-2.36, 1.06)	0.02 (-0.81, 0.85)	-1.18 (-2.40, 0.03)		**1.92 (0.76, 3.08)**	.	.	.
DP+AET	0.53 (-0.24, 1.30)	0.87 (-0.45, 2.19)	0.20 (-1.56, 1.97)	0.88 (-0.06, 1.82)	-0.33 (-1.62, 0.96)	**0.86 (0.32, 1.40)**		0.53 (-0.70, 1.76)	.	.
DP+MET	0.19 (-0.43, 0.81)	0.53 (-0.72, 1.79)	-0.13 (-1.83, 1.57)	0.54 (-0.30, 1.39)	-0.66 (-1.87, 0.55)	**0.52 (0.17, 0.88)**	-0.33 (-0.89, 0.22)		.	.
Rice+MET	0.00 (-0.97, 0.97)	0.34 (-1.12, 1.80)	-0.32 (-2.18, 1.54)	0.35 (-0.77, 1.48)	-0.85 (-2.27, 0.57)	0.33 (-0.50, 1.17)	-0.52 (-1.46, 0.41)	-0.19 (-1.01, 0.63)		.
Ins-PS+AET	-0.21 (-1.45, 1.03)	0.13 (-1.51, 1.77)	-0.53 (-2.54, 1.48)	0.14 (-1.21, 1.49)	-1.06 (-2.67, 0.55)	0.12 (-1.00, 1.24)	-0.74 (-1.87, 0.40)	-0.40 (-1.52, 0.72)	-0.21 (-1.56, 1.14)	
WP	0.16 (-0.47, 0.78)	0.50 (-0.74, 1.74)	-0.17 (-1.87, 1.54)	0.51 (-0.32, 1.33)	-0.70 (-1.91, 0.51)	**0.49 (0.14, 0.84)**	-0.37 (-0.91, 0.17)	-0.04 (-0.39, 0.32)	0.15 (-0.67, 0.98)	0.37 (-0.73, 1.46)
MP	0.38 (-0.36, 1.11)	0.72 (-0.59, 2.03)	0.06 (-1.69, 1.80)	0.73 (-0.20, 1.65)	-0.47 (-1.74, 0.79)	**0.71 (0.19, 1.23)**	-0.15 (-0.83, 0.53)	0.19 (-0.32, 0.69)	0.38 (-0.50, 1.25)	0.59 (-0.60, 1.77)
Casein	0.31 (-0.76, 1.38)	0.65 (-0.87, 2.18)	-0.01 (-1.92, 1.90)	0.67 (-0.55, 1.88)	-0.54 (-2.03, 0.95)	0.65 (-0.30, 1.60)	-0.21 (-1.26, 0.83)	0.12 (-0.81, 1.06)	0.31 (-0.89, 1.51)	0.52 (-0.90, 1.95)
SP	0.24 (-0.59, 1.07)	0.59 (-0.78, 1.95)	-0.08 (-1.88, 1.72)	0.60 (-0.40, 1.59)	-0.61 (-1.94, 0.73)	0.58 (-0.09, 1.24)	-0.28 (-1.08, 0.51)	0.05 (-0.61, 0.71)	0.24 (-0.76, 1.24)	0.45 (-0.80, 1.71)
Meat	0.85 (-0.46, 2.16)	1.19 (-0.51, 2.90)	0.53 (-1.52, 2.58)	1.20 (-0.23, 2.64)	0.00 (-1.32, 1.32)	1.18 (-0.03, 2.40)	0.33 (-0.96, 1.62)	0.66 (-0.55, 1.87)	0.85 (-0.57, 2.27)	1.06 (-0.55, 2.67)
DP	0.68 (-0.01, 1.37)	1.02 (-0.26, 2.30)	0.36 (-1.38, 2.09)	**1.03 (0.14, 1.92)**	-0.17 (-1.42, 1.07)	**1.01 (0.59, 1.43)**	0.15 (-0.46, 0.76)	**0.49 (0.05, 0.93)**	0.68 (-0.20, 1.56)	0.89 (-0.27, 2.04)
RET	-0.10 (-0.71, 0.52)	0.25 (-0.98, 1.47)	-0.42 (-2.12, 1.29)	0.26 (-0.52, 1.03)	-0.95 (-2.15, 0.26)	0.24 (-0.06, 0.53)	**-0.62 (-1.15, -0.09)**	-0.29 (-0.61, 0.04)	-0.10 (-0.91, 0.72)	0.11 (-0.99, 1.22)
AET	0.06 (-1.00, 1.11)	0.40 (-1.10, 1.90)	-0.26 (-2.17, 1.64)	0.41 (-0.77, 1.59)	-0.79 (-2.27, 0.68)	0.39 (-0.52, 1.30)	-0.47 (-1.40, 0.46)	-0.13 (-1.05, 0.78)	0.06 (-1.12, 1.24)	0.27 (-0.51, 1.05)
MET	0.20 (-0.38, 0.79)	0.54 (-0.70, 1.79)	-0.12 (-1.79, 1.56)	0.56 (-0.28, 1.39)	-0.65 (-1.83, 0.54)	**0.54 (0.19, 0.88)**	-0.32 (-0.88, 0.23)	0.01 (-0.28, 0.30)	0.20 (-0.60, 1.00)	0.41 (-0.70, 1.53)
RC	0.40 (-0.18, 0.98)	0.74 (-0.48, 1.97)	0.08 (-1.61, 1.77)	0.76 (-0.05, 1.56)	-0.45 (-1.63, 0.74)	**0.74 (0.47, 1.00)**	-0.12 (-0.64, 0.39)	0.21 (-0.04, 0.46)	0.40 (-0.39, 1.20)	0.61 (-0.49, 1.71)
	Relative effects of NMA (column compared with row)									

To be continued.

Table S5. Continued.

	Direct evidence of pairwise meta-analyses (row compared with column)									
	WP	MP	Casein	SP	Meat	DP	RET	AET	MET	RC
WP+RET	**0.77 (0.39, 1.16)**	.	.	.	.	**1.28 (0.08, 2.48)**	0.18 (-0.05, 0.40)	.	.	**1.08 (0.75, 1.40)**
WP+AET	-0.42 (-1.57, 0.74)	.	.	.	.	.	.	0.18 (-0.40, 0.76)	.	.
WP+MET	0.05 (-0.44, 0.54)	.	.	.	.	.	.	.	**0.34 (0.02, 0.65)**	**0.61 (0.26, 0.95)**
MP+RET	.	0.48 (-0.92, 1.89)	.	.	.	.	0.16 (-0.17, 0.49)	.	.	0.49 (-0.07, 1.06)
MP+AET	0.50 (-0.39, 1.39)	.	.	.	.	.	0.47 (-0.43, 1.37)	-0.25 (-1.31, 0.81)	.	0.67 (-0.23, 1.57)
MP+MET	.	0.23 (-0.31, 0.76)	.	.	.	.	.	.	0.04 (-0.58, 0.65)	0.33 (-0.16, 0.81)
Casein+RET	.	.	.	.	.	.	.	.	.	0.24 (-0.88, 1.36)
Casein+MET	.	.	0.09 (-1.00, 1.17)	.	.	.	.	.	-0.07 (-0.87, 0.72)	0.17 (-0.91, 1.25)
SP+RET	.	.	.	**1.44 (0.58, 2.30)**	.	.	**0.52 (0.00, 1.04)**	.	.	**1.08 (0.40, 1.77)**
SP+AET	.	.	.	.	.	.	.	-0.09 (-0.90, 0.73)	.	.
SP+MET	.	.	.	-0.00 (-1.65, 1.65)	.	-0.11 (-1.76, 1.53)	.	.	0.41 (-0.32, 1.14)	0.25 (-0.45, 0.96)
Collagen+RET	.	.	.	.	.	.	.	.	.	.
Collagen+MET	.	.	.	.	.	.	.	.	-0.12 (-1.79, 1.56)	.
Meat+RET	.	.	.	.	.	.	0.26 (-0.52, 1.03)	.	.	.
Meat+MET	.	.	.	.	-0.00 (-1.32, 1.32)	.	.	.	-0.55 (-1.92, 0.81)	-0.55 (-1.94, 0.84)
DP+RET	.	.	.	.	.	**1.06 (0.43, 1.68)**	**0.72 (0.25, 1.20)**	.	.	**0.34 (0.00, 0.69)**
DP+AET	-0.67 (-1.82, 0.49)	.	.	.	.	0.15 (-1.09, 1.39)	.	.	.	**1.27 (0.42, 2.12)**
DP+MET	.	.	.	0.11 (-1.53, 1.76)	.	0.58 (-0.17, 1.34)	.	.	0.21 (-0.21, 0.64)	0.12 (-0.18, 0.42)
Rice+MET	.	.	.	.	.	.	.	.	.	.
Ins-PS+AET	.	.	.	.	.	.	.	0.30 (-0.54, 1.15)	.	.
WP		.	.	.	.	.	-0.34 (-0.73, 0.05)	.	-0.19 (-0.67, 0.29)	0.35 (-0.01, 0.71)
MP	0.22 (-0.30, 0.74)		.	.	.	0.19 (-0.90, 1.27)	.	.	-0.21 (-1.24, 0.83)	0.12 (-0.63, 0.87)
Casein	0.16 (-0.79, 1.10)	-0.06 (-1.08, 0.95)		.	.	.	.	.	0.00 (-1.08, 1.08)	0.09 (-1.00, 1.17)
SP	0.09 (-0.57, 0.75)	-0.13 (-0.90, 0.63)	-0.07 (-1.17, 1.03)		.	-0.11 (-1.81, 1.59)	-0.14 (-0.99, 0.70)	.	0.16 (-1.38, 1.70)	0.45 (-0.28, 1.18)
Meat	0.70 (-0.51, 1.91)	0.47 (-0.79, 1.74)	0.54 (-0.95, 2.03)	0.61 (-0.73, 1.94)		.	.	.	-0.55 (-1.92, 0.81)	-0.55 (-1.94, 0.84)
DP	**0.52 (0.06, 0.98)**	0.30 (-0.27, 0.87)	0.36 (-0.63, 1.36)	0.43 (-0.28, 1.15)	-0.17 (-1.42, 1.07)		0.01 (-1.14, 1.16)	.	0.27 (-1.27, 1.81)	0.12 (-0.49, 0.74)
RET	-0.25 (-0.53, 0.03)	-0.47 (-0.97, 0.03)	-0.41 (-1.35, 0.53)	-0.34 (-0.96, 0.28)	-0.95 (-2.15, 0.26)	**-0.77 (-1.20, -0.35)**		.	.	**0.40 (0.09, 0.70)**
AET	-0.10 (-0.98, 0.78)	-0.32 (-1.31, 0.67)	-0.26 (-1.52, 1.01)	-0.19 (-1.26, 0.88)	-0.79 (-2.27, 0.68)	-0.62 (-1.58, 0.33)	0.15 (-0.73, 1.04)		.	.
MET	0.05 (-0.27, 0.36)	-0.17 (-0.66, 0.32)	-0.11 (-1.02, 0.80)	-0.04 (-0.69, 0.61)	-0.65 (-1.83, 0.54)	**-0.48 (-0.92, -0.03)**	**0.30 (0.00, 0.60)**	0.15 (-0.76, 1.05)		**0.41 (0.10, 0.72)**
RC	0.25 (-0.02, 0.51)	0.03 (-0.44, 0.49)	0.09 (-0.82, 1.00)	0.16 (-0.46, 0.78)	-0.45 (-1.63, 0.74)	-0.27 (-0.67, 0.12)	**0.50 (0.28, 0.71)**	0.35 (-0.53, 1.23)	0.20 (-0.03, 0.43)	
	Relative effects of NMA (column compared with row)									

Pairwise (upper right portion) and network (lower left portion) meta-analysis results are presented for mean change (from baseline) in global function. Effect estimation is presented in standardized mean difference with 95% confidence interval. Significant results (p <0.05) are marked in bold.

AET, aerobic exercise training; DP, dietary protein; Ins-PS, insect protein supplement; MET, multicomponent exercise training; MP, milk protein; RET, resistance exercise training; SP, soy protein; WP, whey protein; RC, regular care.

Table S6. Direct and network estimates for leg strength.

	Direct evidence of pairwise meta-analyses (row compared with column)										
	WP+RET	WP+AET	WP+MET	MP+RET	MP+AET	MP+MET	Casein+RET	Casein+MET	SP+RET	SP+AET	SP+MET
WP+RET		.	.	-0.05 (-0.81, 0.71)	.	.	0.06 (-0.81, 0.92)	.	0.34 (-0.60, 1.28)	.	.
WP+AET	0.24 (-0.56, 1.05)		.	.	.	.	.	.	.	0.18 (-1.16, 1.52)	.
WP+MET	0.05 (-0.35, 0.46)	-0.19 (-1.04, 0.66)		.	.	0.94 (-0.35, 2.22)	.	**1.57 (0.66, 2.49)**	.	.	.
MP+RET	0.23 (-0.08, 0.53)	-0.02 (-0.81, 0.78)	0.17 (-0.25, 0.60)		0.38 (-0.54, 1.30)	-0.05 (-1.30, 1.21)	.	.	-0.13 (-0.91, 0.65)	.	.
MP+AET	0.58 (-0.14, 1.29)	0.33 (-0.49, 1.15)	0.52 (-0.25, 1.30)	0.35 (-0.35, 1.05)		.	.	.	.	.	.
MP+MET	**0.50 (0.06, 0.94)**	0.25 (-0.61, 1.12)	0.44 (-0.01, 0.89)	0.27 (-0.17, 0.71)	-0.08 (-0.87, 0.71)		.	.	.	.	.
Casein+RET	0.06 (-0.81, 0.92)	-0.19 (-1.36, 0.99)	0.00 (-0.95, 0.96)	-0.17 (-1.08, 0.75)	-0.52 (-1.64, 0.60)	-0.44 (-1.41, 0.53)		.	.	.	.
Casein+MET	0.57 (-0.05, 1.19)	0.33 (-0.64, 1.30)	0.52 (-0.06, 1.09)	0.35 (-0.29, 0.98)	-0.01 (-0.91, 0.90)	0.08 (-0.58, 0.73)	0.51 (-0.55, 1.58)		.	.	.
SP+RET	0.05 (-0.31, 0.41)	-0.19 (-1.03, 0.65)	-0.00 (-0.46, 0.46)	-0.17 (-0.55, 0.21)	-0.52 (-1.28, 0.23)	-0.44 (-0.94, 0.05)	-0.00 (-0.94, 0.93)	-0.52 (-1.18, 0.14)		.	.
SP+AET	0.32 (-0.77, 1.42)	0.08 (-0.89, 1.05)	0.27 (-0.86, 1.40)	0.10 (-0.99, 1.18)	-0.25 (-1.34, 0.83)	-0.17 (-1.32, 0.97)	0.27 (-1.13, 1.66)	-0.25 (-1.47, 0.98)	0.27 (-0.85, 1.39)		.
SP+MET	0.59 (-0.13, 1.31)	0.35 (-0.69, 1.38)	0.54 (-0.20, 1.27)	0.36 (-0.37, 1.10)	0.01 (-0.97, 0.99)	0.09 (-0.68, 0.86)	0.53 (-0.59, 1.66)	0.02 (-0.85, 0.89)	0.54 (-0.21, 1.29)	0.27 (-1.01, 1.54)	
Collagen+RET	-0.04 (-0.66, 0.59)	-0.28 (-1.27, 0.71)	-0.09 (-0.80, 0.62)	-0.26 (-0.92, 0.39)	-0.62 (-1.54, 0.31)	-0.53 (-1.27, 0.20)	-0.09 (-1.16, 0.97)	-0.61 (-1.46, 0.24)	-0.09 (-0.77, 0.59)	-0.36 (-1.60, 0.88)	-0.63 (-1.55, 0.30)
Meat+RET	0.22 (-0.31, 0.76)	-0.02 (-0.95, 0.91)	0.17 (-0.44, 0.78)	-0.00 (-0.56, 0.55)	-0.36 (-1.21, 0.50)	-0.27 (-0.90, 0.35)	0.16 (-0.85, 1.18)	-0.35 (-1.12, 0.42)	0.17 (-0.38, 0.72)	-0.10 (-1.29, 1.09)	-0.37 (-1.22, 0.49)
Meat+MET	**1.21 (0.29, 2.13)**	0.96 (-0.22, 2.15)	**1.15 (0.23, 2.08)**	**0.98 (0.05, 1.91)**	0.63 (-0.51, 1.76)	0.71 (-0.25, 1.67)	1.15 (-0.11, 2.41)	0.63 (-0.41, 1.68)	**1.15 (0.21, 2.10)**	0.88 (-0.52, 2.28)	0.62 (-0.49, 1.73)
DP+RET	0.41 (-0.14, 0.97)	0.17 (-0.77, 1.10)	0.36 (-0.25, 0.96)	0.19 (-0.39, 0.76)	-0.17 (-1.03, 0.70)	-0.09 (-0.72, 0.55)	0.35 (-0.67, 1.38)	-0.16 (-0.93, 0.61)	0.36 (-0.24, 0.96)	0.09 (-1.11, 1.28)	-0.18 (-1.03, 0.67)
DP+AET	**1.47 (0.57, 2.37)**	**1.23 (0.02, 2.43)**	**1.42 (0.43, 2.40)**	**1.24 (0.29, 2.19)**	0.89 (-0.26, 2.04)	0.97 (-0.03, 1.98)	**1.41 (0.17, 2.66)**	0.90 (-0.20, 1.99)	**1.42 (0.45, 2.39)**	1.15 (-0.27, 2.56)	0.88 (-0.27, 2.03)
DP+MET	**1.10 (0.53, 1.66)**	0.85 (-0.08, 1.79)	**1.04 (0.48, 1.61)**	**0.87 (0.29, 1.45)**	0.52 (-0.35, 1.39)	0.60 (-0.02, 1.22)	**1.04 (0.01, 2.07)**	0.52 (-0.22, 1.27)	**1.04 (0.44, 1.65)**	0.77 (-0.42, 1.97)	0.51 (-0.33, 1.35)
Rice+RET	0.47 (-0.30, 1.23)	0.22 (-0.85, 1.29)	0.41 (-0.39, 1.21)	0.24 (-0.52, 1.00)	-0.11 (-1.12, 0.90)	-0.03 (-0.79, 0.73)	0.41 (-0.74, 1.56)	-0.11 (-1.04, 0.83)	0.41 (-0.36, 1.18)	0.14 (-1.16, 1.45)	-0.12 (-1.13, 0.88)
Ins-PS+AET	0.09 (-1.24, 1.41)	-0.16 (-1.33, 1.02)	0.03 (-1.32, 1.39)	-0.14 (-1.46, 1.18)	-0.49 (-1.81, 0.83)	-0.41 (-1.77, 0.95)	0.03 (-1.55, 1.61)	-0.49 (-1.92, 0.95)	0.03 (-1.31, 1.38)	-0.24 (-1.68, 1.21)	-0.50 (-1.98, 0.98)
Oat+MET	0.36 (-1.09, 1.81)	0.12 (-1.51, 1.75)	0.31 (-1.08, 1.70)	0.14 (-1.32, 1.59)	-0.21 (-1.81, 1.38)	-0.13 (-1.60, 1.33)	0.31 (-1.38, 1.99)	-0.21 (-1.71, 1.30)	0.31 (-1.16, 1.78)	0.04 (-1.75, 1.83)	-0.22 (-1.80, 1.35)
WP	**1.21 (0.89, 1.54)**	**0.97 (0.14, 1.80)**	**1.16 (0.76, 1.56)**	**0.99 (0.61, 1.37)**	0.63 (-0.12, 1.39)	**0.72 (0.25, 1.18)**	**1.15 (0.23, 2.08)**	**0.64 (0.02, 1.26)**	**1.16 (0.74, 1.58)**	0.89 (-0.22, 2.00)	0.62 (-0.11, 1.36)
MP	**1.06 (0.66, 1.45)**	0.81 (-0.03, 1.66)	**1.00 (0.56, 1.45)**	**0.83 (0.44, 1.22)**	0.48 (-0.29, 1.24)	**0.56 (0.14, 0.99)**	**1.00 (0.05, 1.95)**	0.48 (-0.16, 1.13)	**1.00 (0.55, 1.46)**	0.73 (-0.39, 1.86)	0.47 (-0.28, 1.22)
Casein	0.79 (-0.07, 1.66)	0.55 (-0.60, 1.70)	0.74 (-0.13, 1.61)	0.57 (-0.31, 1.45)	0.22 (-0.88, 1.31)	0.30 (-0.61, 1.20)	0.74 (-0.48, 1.96)	0.22 (-0.70, 1.14)	0.74 (-0.16, 1.64)	0.47 (-0.90, 1.84)	0.21 (-0.86, 1.27)
SP	**1.02 (0.57, 1.47)**	0.78 (-0.10, 1.66)	**0.97 (0.45, 1.49)**	**0.80 (0.32, 1.27)**	0.44 (-0.36, 1.25)	0.53 (-0.03, 1.08)	0.96 (-0.01, 1.94)	0.45 (-0.25, 1.15)	**0.97 (0.51, 1.43)**	0.70 (-0.45, 1.85)	0.43 (-0.34, 1.20)
Collagen	**1.21 (0.21, 2.22)**	0.97 (-0.30, 2.24)	**1.16 (0.11, 2.21)**	0.99 (-0.04, 2.02)	0.64 (-0.58, 1.85)	0.72 (-0.35, 1.78)	1.16 (-0.17, 2.48)	0.64 (-0.51, 1.79)	**1.16 (0.12, 2.20)**	0.89 (-0.58, 2.36)	0.62 (-0.58, 1.83)
Meat	**1.36 (0.62, 2.10)**	**1.12 (0.06, 2.17)**	**1.31 (0.53, 2.08)**	**1.13 (0.38, 1.89)**	0.78 (-0.21, 1.78)	**0.86 (0.06, 1.66)**	**1.30 (0.17, 2.44)**	0.79 (-0.12, 1.69)	**1.31 (0.54, 2.07)**	1.04 (-0.25, 2.33)	0.77 (-0.20, 1.75)
DP	**1.39 (0.69, 2.10)**	**1.15 (0.12, 2.18)**	**1.34 (0.62, 2.07)**	**1.17 (0.45, 1.89)**	0.82 (-0.15, 1.79)	**0.90 (0.14, 1.65)**	**1.34 (0.22, 2.45)**	0.82 (-0.04, 1.69)	**1.34 (0.60, 2.09)**	1.07 (-0.20, 2.34)	0.81 (-0.14, 1.75)
RET	**0.37 (0.18, 0.57)**	0.13 (-0.66, 0.92)	0.32 (-0.06, 0.70)	0.15 (-0.12, 0.41)	-0.20 (-0.90, 0.49)	-0.12 (-0.54, 0.30)	0.32 (-0.57, 1.20)	-0.20 (-0.81, 0.41)	**0.32 (0.00, 0.65)**	0.05 (-1.03, 1.13)	-0.22 (-0.92, 0.49)
AET	**0.66 (0.02, 1.31)**	0.42 (-0.15, 0.98)	0.61 (-0.10, 1.31)	0.43 (-0.20, 1.07)	0.08 (-0.54, 0.70)	0.16 (-0.56, 0.89)	0.60 (-0.47, 1.68)	0.09 (-0.76, 0.94)	0.61 (-0.08, 1.30)	0.34 (-0.56, 1.24)	0.07 (-0.85, 1.00)
MET	**0.63 (0.27, 0.99)**	0.39 (-0.44, 1.22)	**0.58 (0.30, 0.86)**	**0.41 (0.02, 0.79)**	0.06 (-0.70, 0.81)	0.14 (-0.27, 0.54)	0.58 (-0.36, 1.51)	0.06 (-0.49, 0.61)	**0.58 (0.15, 1.01)**	0.31 (-0.81, 1.43)	0.04 (-0.66, 0.74)
RC	**1.16 (0.90, 1.42)**	**0.92 (0.13, 1.70)**	**1.11 (0.77, 1.44)**	**0.93 (0.64, 1.23)**	0.58 (-0.13, 1.29)	**0.66 (0.27, 1.05)**	**1.10 (0.20, 2.00)**	**0.59 (0.01, 1.17)**	**1.11 (0.77, 1.45)**	0.84 (-0.25, 1.92)	0.57 (-0.11, 1.25)
	Relative effects of NMA (column compared with row)										

To be continued.

Table S6. Continued.

	Direct evidence of pairwise meta-analyses (row compared with column)									
	Collagen+RET	Meat+RET	Meat+MET	DP+RET	DP+AET	DP+MET	Rice+RET	Ins-PS+AET	Oat+MET	WP
WP+RET	-0.22 (-1.45, 1.02)	.	.	.	**1.47 (0.57, 2.37)**	.	.	.	.	**1.07 (0.64, 1.50)**
WP+AET	.	.	.	.	.	.	.	-0.41 (-1.72, 0.89)	.	.
WP+MET	.	.	.	.	.	0.32 (-1.12, 1.76)	.	.	0.31 (-1.08, 1.70)	**1.53 (0.90, 2.17)**
MP+RET	.	.	.	.	.	.	0.13 (-1.34, 1.60)	.	.	.
MP+AET	.	.	.	.	.	.	.	.	.	.
MP+MET	.	.	.	.	.	.	0.16 (-1.01, 1.33)	.	.	.
Casein+RET	.	.	.	.	.	.	.	.	.	.
Casein+MET	.	.	.	.	.	.	.	.	.	**2.07 (0.56, 3.58)**
SP+RET	.	0.32 (-0.70, 1.34)	.	.	.	.	0.08 (-1.39, 1.55)	.	.	-0.16 (-1.59, 1.27)
SP+AET	.	.	.	.	.	.	.	.	.	.
SP+MET	.	.	.	.	.	.	.	.	.	.
Collagen+RET		.	.	.	.	.	.	.	.	.
Meat+RET	0.26 (-0.53, 1.05)		.	0.76 (-0.67, 2.18)	.	.	0.25 (-0.97, 1.46)	.	.	.
Meat+MET	**1.24 (0.15, 2.33)**	0.98 (-0.04, 2.01)		.	.	0.02 (-1.16, 1.20)	.	.	.	.
DP+RET	0.45 (-0.35, 1.25)	0.19 (-0.50, 0.88)	-0.80 (-1.81, 0.22)		.	0.98 (-0.20, 2.15)	.	.	.	.
DP+AET	**1.51 (0.41, 2.60)**	**1.25 (0.20, 2.30)**	0.26 (-1.03, 1.55)	**1.06 (0.00, 2.12)**		.	.	.	.	.
DP+MET	**1.13 (0.32, 1.95)**	**0.88 (0.15, 1.60)**	-0.11 (-0.99, 0.77)	**0.69 (0.00, 1.37)**	-0.37 (-1.44, 0.69)		.	.	.	.
Rice+RET	0.50 (-0.46, 1.46)	0.24 (-0.54, 1.03)	-0.74 (-1.89, 0.41)	0.06 (-0.84, 0.95)	-1.00 (-2.18, 0.18)	-0.63 (-1.53, 0.27)		.	.	.
Ins-PS+AET	0.12 (-1.32, 1.57)	-0.14 (-1.54, 1.27)	-1.12 (-2.71, 0.47)	-0.32 (-1.73, 1.08)	-1.38 (-2.98, 0.22)	-1.01 (-2.42, 0.40)	-0.38 (-1.88, 1.12)		.	.
Oat+MET	0.40 (-1.16, 1.96)	0.14 (-1.38, 1.66)	-0.84 (-2.51, 0.83)	-0.05 (-1.57, 1.47)	-1.11 (-2.81, 0.60)	-0.73 (-2.23, 0.77)	-0.10 (-1.71, 1.50)	0.28 (-1.67, 2.22)		.
WP	**1.25 (0.57, 1.93)**	**0.99 (0.41, 1.57)**	0.01 (-0.93, 0.94)	**0.80 (0.21, 1.39)**	-0.26 (-1.21, 0.70)	0.12 (-0.46, 0.69)	0.75 (-0.04, 1.53)	1.13 (-0.22, 2.47)	0.85 (-0.60, 2.30)	
MP	**1.09 (0.39, 1.80)**	**0.84 (0.23, 1.44)**	-0.15 (-1.09, 0.79)	**0.65 (0.04, 1.26)**	-0.41 (-1.40, 0.57)	-0.04 (-0.64, 0.56)	0.59 (-0.20, 1.38)	0.97 (-0.38, 2.32)	0.69 (-0.77, 2.15)	-0.15 (-0.58, 0.27)
Casein	0.83 (-0.21, 1.88)	0.57 (-0.41, 1.55)	-0.41 (-1.63, 0.80)	0.38 (-0.60, 1.37)	-0.68 (-1.92, 0.57)	-0.30 (-1.27, 0.67)	0.33 (-0.79, 1.44)	0.71 (-0.85, 2.26)	0.43 (-1.21, 2.07)	-0.42 (-1.27, 0.43)
SP	**1.06 (0.32, 1.80)**	**0.80 (0.16, 1.43)**	-0.19 (-1.16, 0.79)	0.61 (-0.04, 1.27)	-0.45 (-1.46, 0.56)	-0.08 (-0.73, 0.58)	0.55 (-0.28, 1.39)	0.93 (-0.44, 2.31)	0.66 (-0.83, 2.14)	-0.19 (-0.68, 0.30)
Collagen	**1.25 (0.08, 2.42)**	0.99 (-0.13, 2.11)	0.01 (-1.33, 1.34)	0.80 (-0.32, 1.92)	-0.26 (-1.60, 1.09)	0.12 (-1.00, 1.24)	0.75 (-0.49, 1.99)	1.13 (-0.52, 2.77)	0.85 (-0.89, 2.59)	0.00 (-1.01, 1.01)
Meat	**1.40 (0.46, 2.34)**	**1.14 (0.31, 1.97)**	0.15 (-0.89, 1.20)	**0.95 (0.08, 1.82)**	-0.11 (-1.28, 1.06)	0.26 (-0.59, 1.12)	0.89 (-0.12, 1.90)	1.27 (-0.22, 2.76)	1.00 (-0.60, 2.59)	0.15 (-0.61, 0.91)
DP	**1.43 (0.52, 2.35)**	**1.17 (0.33, 2.02)**	0.19 (-0.90, 1.28)	**0.98 (0.14, 1.82)**	-0.07 (-1.22, 1.07)	0.30 (-0.50, 1.09)	0.93 (-0.07, 1.92)	1.31 (-0.17, 2.78)	1.03 (-0.54, 2.60)	0.18 (-0.52, 0.89)
RET	0.41 (-0.20, 1.02)	0.15 (-0.36, 0.66)	-0.83 (-1.75, 0.08)	-0.04 (-0.57, 0.50)	**-1.10 (-2.02, -0.17)**	**-0.72 (-1.27, -0.17)**	-0.09 (-0.84, 0.66)	0.29 (-1.03, 1.60)	0.01 (-1.43, 1.45)	**-0.84 (-1.15, -0.52)**
AET	0.70 (-0.17, 1.57)	0.44 (-0.36, 1.23)	-0.55 (-1.63, 0.54)	0.25 (-0.55, 1.05)	-0.81 (-1.92, 0.30)	-0.44 (-1.25, 0.37)	0.19 (-0.76, 1.15)	0.57 (-0.60, 1.75)	0.30 (-1.26, 1.86)	-0.55 (-1.23, 0.13)
MET	0.67 (-0.02, 1.36)	0.41 (-0.17, 0.99)	-0.57 (-1.48, 0.33)	0.22 (-0.36, 0.80)	-0.84 (-1.81, 0.13)	-0.46 (-1.00, 0.07)	0.17 (-0.61, 0.95)	0.55 (-0.80, 1.89)	0.27 (-1.15, 1.69)	**-0.58 (-0.94, -0.21)**
RC	**1.20 (0.56, 1.83)**	**0.94 (0.41, 1.46)**	-0.05 (-0.94, 0.84)	**0.75 (0.23, 1.27)**	-0.31 (-1.25, 0.63)	0.06 (-0.45, 0.58)	0.69 (-0.06, 1.44)	1.07 (-0.24, 2.39)	0.80 (-0.64, 2.23)	-0.05 (-0.36, 0.25)
	Relative effects of NMA (column compared with row)									

To be continued.

Table S6. Continued.

	Direct evidence of pairwise meta-analyses (row compared with column)									
	MP	Casein	SP	Collagen	Meat	DP	RET	AET	MET	RC
WP+RET	.	.	-0.04 (-1.42, 1.34)	0.79 (-0.41, 1.99)	.	.	**0.44 (0.23, 0.66)**	.	.	**1.02 (0.57, 1.48)**
WP+AET	.	.	.	.	.	.	.	0.36 (-0.22, 0.95)	.	**1.45 (0.15, 2.75)**
WP+MET	.	.	.	.	.	.	.	.	**0.60 (0.28, 0.91)**	**1.17 (0.67, 1.68)**
MP+RET	**0.75 (0.20, 1.31)**	.	.	.	.	.	0.17 (-0.15, 0.49)	0.44 (-0.47, 1.36)	.	**0.79 (0.27, 1.30)**
MP+AET	.	.	.	.	.	.	-0.28 (-1.20, 0.64)	0.10 (-0.54, 0.74)	.	.
MP+MET	0.44 (-0.10, 0.99)	.	.	.	.	.	.	.	0.17 (-0.37, 0.71)	**0.78 (0.20, 1.35)**
Casein+RET	.	.	.	.	.	.	.	.	.	.
Casein+MET	.	0.13 (-1.09, 1.34)	.	.	.	.	.	.	0.25 (-0.40, 0.90)	0.90 (-0.05, 1.85)
SP+RET	.	.	**1.31 (0.77, 1.85)**	.	.	.	0.03 (-0.39, 0.45)	.	.	1.53 (1.03, 2.03)
SP+AET	.	.	.	.	.	.	.	0.29 (-0.66, 1.24)	.	.
SP+MET	.	.	0.21 (-1.18, 1.61)	.	.	.	.	.	0.24 (-0.69, 1.16)	0.60 (-0.15, 1.36)
Collagen+RET	.	.	.	.	.	.	0.40 (-0.25, 1.05)	.	.	0.90 (-0.57, 2.38)
Meat+RET	.	.	.	.	1.17 (-0.14, 2.48)	.	0.12 (-0.54, 0.78)	.	.	1.09 (-0.22, 2.40)
Meat+MET	.	.	.	.	-0.00 (-1.43, 1.43)	.	.	.	-0.43 (-1.90, 1.04)	-0.43 (-1.93, 1.06)
DP+RET	.	.	.	.	.	.	0.12 (-0.65, 0.89)	.	.	**0.76 (0.13, 1.39)**
DP+AET	.	.	.	.	.	.	.	.	.	.
DP+MET	.	.	.	.	.	0.76 (-0.51, 2.02)	.	.	0.03 (-0.87, 0.93)	-0.33 (-1.03, 0.36)
Rice+RET	.	.	.	.	.	.	.	.	.	.
Ins-PS+AET	.	.	.	.	.	.	.	0.31 (-1.00, 1.63)	.	.
Oat+MET	.	.	.	.	.	.	.	.	.	.
WP	.	-0.39 (-1.81, 1.03)	-0.04 (-1.47, 1.39)	0.11 (-1.10, 1.32)	.	0.41 (-1.03, 1.85)	**-0.70 (-1.22, -0.19)**	.	**-1.00 (-1.62, -0.39)**	-0.02 (-0.43, 0.39)
MP		.	.	.	.	-0.30 (-1.52, 0.93)	-0.56 (-1.18, 0.06)	.	-0.41 (-1.12, 0.29)	-0.02 (-0.43, 0.39)
Casein	-0.26 (-1.16, 0.63)		.	.	.	.	.	.	0.01 (-1.21, 1.23)	0.14 (-1.09, 1.36)
SP	-0.04 (-0.56, 0.49)	0.23 (-0.70, 1.16)		.	0.39 (-1.04, 1.81)	.	**-0.95 (-1.60, -0.30)**	.	0.26 (-1.14, 1.67)	0.40 (-0.14, 0.93)
Collagen	0.16 (-0.89, 1.21)	0.42 (-0.87, 1.71)	0.19 (-0.88, 1.27)		.	.	.	.	.	-0.39 (-1.62, 0.83)
Meat	0.30 (-0.48, 1.08)	0.57 (-0.53, 1.66)	0.34 (-0.43, 1.11)	0.15 (-1.08, 1.37)		.	-1.20 (-2.50, 0.10)	.	-0.43 (-1.90, 1.04)	-0.23 (-1.20, 0.74)
DP	0.34 (-0.38, 1.05)	0.60 (-0.46, 1.66)	0.37 (-0.41, 1.16)	0.18 (-1.01, 1.38)	0.04 (-0.94, 1.01)		.	.	-0.95 (-2.23, 0.33)	-0.21 (-1.09, 0.67)
RET	**-0.68 (-1.05, -0.32)**	-0.42 (-1.28, 0.44)	**-0.65 (-1.08, -0.22)**	-0.84 (-1.85, 0.17)	**-0.99 (-1.71, -0.26)**	**-1.02 (-1.72, -0.33)**		0.35 (-0.56, 1.26)	.	**0.94 (0.65, 1.24)**
AET	-0.40 (-1.09, 0.30)	-0.13 (-1.18, 0.91)	-0.36 (-1.10, 0.38)	-0.55 (-1.72, 0.62)	-0.70 (-1.64, 0.24)	-0.73 (-1.65, 0.18)	0.29 (-0.34, 0.91)		.	0.24 (-1.03, 1.52)
MET	**-0.42 (-0.82, -0.03)**	-0.16 (-1.01, 0.68)	-0.39 (-0.88, 0.10)	-0.58 (-1.61, 0.45)	-0.73 (-1.47, 0.02)	**-0.76 (-1.46, -0.06)**	0.26 (-0.08, 0.60)	-0.03 (-0.71, 0.66)		**0.54 (0.19, 0.89)**
RC	0.10 (-0.24, 0.44)	0.37 (-0.47, 1.21)	0.14 (-0.28, 0.56)	-0.05 (-1.06, 0.95)	-0.20 (-0.92, 0.52)	-0.24 (-0.91, 0.44)	**0.79 (0.56, 1.01)**	0.50 (-0.13, 1.13)	**0.53 (0.24, 0.81)**	
	Relative effects of NMA (column compared with row)									

Pairwise (upper right portion) and network (lower left portion) meta-analysis results are presented for mean change (from baseline) in global function. Effect estimation is presented in standardized mean difference with 95% confidence interval. Significant results ($p < 0.05$) are marked in bold.

AET, aerobic exercise training; DP, dietary protein; Ins-PS, insect protein supplement; MET, multicomponent exercise training; MP, milk protein; RET, resistance exercise training; SP, soy protein; WP, whey protein; RC, regular care.

Table S7. Direct and network estimates for walking speed.

	Direct evidence of pairwise meta-analyses (row compared with column)										
	WP+RET	WP+MET	MP+RET	MP+AET	MP+MET	Casein+RET	Casein+AET	Casein+MET	SP+RET	SP+AET	SP+MET
WP+RET		0.51 (-0.57, 1.59)	0.18 (-0.91, 1.27)	.	.	-0.16 (-1.27, 0.96)	.	.	.	.	.
WP+MET	0.25 (-0.10, 0.60)		.	.	-0.27 (-1.08, 0.53)	.	.	-0.34 (-1.54, 0.87)	.	.	.
MP+RET	0.31 (-0.02, 0.64)	0.06 (-0.35, 0.47)		-0.03 (-1.25, 1.19)	.	.	.	.	-0.12 (-1.02, 0.78)	.	.
MP+AET	0.14 (-0.84, 1.12)	-0.11 (-1.12, 0.90)	-0.17 (-1.13, 0.79)		.	.	.	.	.	.	.
MP+MET	0.35 (-0.07, 0.76)	0.10 (-0.29, 0.49)	0.04 (-0.42, 0.49)	0.21 (-0.83, 1.24)		.	.	.	.	.	.
Casein+RET	-0.16 (-1.27, 0.96)	-0.41 (-1.58, 0.76)	-0.47 (-1.63, 0.70)	-0.30 (-1.78, 1.19)	-0.50 (-1.69, 0.69)		.	.	.	.	.
Casein+AET	**1.43 (0.24, 2.62)**	**1.18 (0.00, 2.36)**	1.12 (-0.09, 2.32)	1.29 (-0.23, 2.81)	1.08 (-0.13, 2.29)	1.58 (-0.05, 3.21)		.	.	.	.
Casein+MET	0.35 (-0.26, 0.95)	0.10 (-0.48, 0.67)	0.03 (-0.60, 0.67)	0.20 (-0.92, 1.33)	-0.00 (-0.64, 0.63)	0.50 (-0.77, 1.77)	-1.08 (-2.34, 0.17)		.	.	.
SP+RET	-0.02 (-0.49, 0.45)	-0.27 (-0.79, 0.25)	-0.33 (-0.82, 0.15)	-0.16 (-1.21, 0.88)	-0.37 (-0.93, 0.19)	0.13 (-1.08, 1.34)	**-1.45 (-2.70, -0.20)**	-0.37 (-1.08, 0.35)		.	.
SP+AET	0.45 (-1.05, 1.95)	0.20 (-1.32, 1.73)	0.14 (-1.35, 1.63)	0.31 (-1.10, 1.71)	0.10 (-1.44, 1.64)	0.61 (-1.26, 2.48)	-0.98 (-2.88, 0.92)	0.10 (-1.50, 1.71)	0.47 (-1.08, 2.02)		.
SP+MET	**0.71 (0.15, 1.28)**	0.46 (-0.10, 1.03)	0.40 (-0.20, 1.00)	0.57 (-0.53, 1.67)	0.37 (-0.25, 0.98)	0.87 (-0.38, 2.12)	-0.71 (-1.99, 0.56)	0.37 (-0.37, 1.11)	**0.73 (0.06, 1.41)**	0.26 (-1.32, 1.85)	
Collagen+RET	-0.17 (-1.41, 1.08)	-0.42 (-1.71, 0.88)	-0.48 (-1.77, 0.81)	-0.31 (-1.89, 1.28)	-0.51 (-1.83, 0.80)	-0.01 (-1.68, 1.66)	-1.59 (-3.32, 0.13)	-0.51 (-1.90, 0.88)	-0.14 (-1.48, 1.19)	-0.62 (-2.57, 1.34)	-0.88 (-2.25, 0.49)
Collagen+MET	0.31 (-1.44, 2.05)	0.06 (-1.68, 1.79)	-0.00 (-1.76, 1.75)	0.17 (-1.82, 2.15)	-0.04 (-1.79, 1.71)	0.46 (-1.61, 2.53)	-1.12 (-3.20, 0.96)	-0.04 (-1.83, 1.76)	0.33 (-1.46, 2.11)	-0.14 (-2.43, 2.15)	-0.41 (-2.20, 1.39)
Meat+RET	0.38 (-0.82, 1.58)	0.13 (-1.10, 1.36)	0.07 (-1.15, 1.28)	0.24 (-1.28, 1.76)	0.03 (-1.22, 1.28)	0.53 (-1.10, 2.17)	-1.05 (-2.72, 0.62)	0.03 (-1.29, 1.36)	0.40 (-0.86, 1.66)	-0.07 (-1.97, 1.83)	-0.33 (-1.64, 0.97)
Meat+MET	0.45 (-0.40, 1.31)	0.20 (-0.65, 1.06)	0.14 (-0.74, 1.02)	0.31 (-0.96, 1.59)	0.11 (-0.78, 0.99)	0.61 (-0.79, 2.02)	-0.97 (-2.39, 0.45)	0.11 (-0.87, 1.09)	0.48 (-0.46, 1.41)	0.00 (-1.71, 1.72)	-0.26 (-1.22, 0.70)
DP+RET	**0.43 (0.04, 0.83)**	0.18 (-0.23, 0.60)	0.12 (-0.33, 0.57)	0.29 (-0.74, 1.32)	0.09 (-0.39, 0.56)	0.59 (-0.59, 1.77)	-0.99 (-2.19, 0.20)	0.09 (-0.55, 0.73)	0.46 (-0.10, 1.01)	-0.02 (-1.55, 1.52)	-0.28 (-0.89, 0.33)
DP+AET	**0.70 (0.12, 1.28)**	0.45 (-0.11, 1.01)	0.39 (-0.23, 1.01)	0.56 (-0.55, 1.67)	0.35 (-0.27, 0.98)	0.86 (-0.40, 2.11)	-0.73 (-1.84, 0.39)	0.36 (-0.39, 1.10)	**0.72 (0.03, 1.42)**	0.25 (-1.34, 1.84)	-0.01 (-0.75, 0.72)
DP+MET	**0.50 (0.16, 0.85)**	0.25 (-0.10, 0.60)	0.19 (-0.21, 0.60)	0.36 (-0.65, 1.37)	0.16 (-0.25, 0.57)	0.66 (-0.51, 1.83)	-0.92 (-2.11, 0.26)	0.16 (-0.44, 0.76)	**0.53 (0.01, 1.04)**	0.05 (-1.47, 1.58)	-0.21 (-0.78, 0.36)
Rice+RET	1.24 (-0.04, 2.51)	0.99 (-0.31, 2.28)	0.93 (-0.33, 2.18)	1.10 (-0.47, 2.67)	0.89 (-0.42, 2.20)	1.39 (-0.30, 3.09)	-0.19 (-1.91, 1.53)	0.89 (-0.49, 2.28)	**1.26 (0.00, 2.52)**	0.79 (-1.15, 2.73)	0.53 (-0.84, 1.89)
Rice+MET	0.43 (-0.43, 1.29)	0.18 (-0.64, 1.00)	0.12 (-0.76, 1.00)	0.29 (-0.99, 1.57)	0.08 (-0.73, 0.89)	0.59 (-0.82, 2.00)	-1.00 (-2.42, 0.43)	0.09 (-0.89, 1.06)	0.45 (-0.49, 1.39)	-0.02 (-1.73, 1.69)	-0.28 (-1.25, 0.69)
WP	**0.44 (0.14, 0.74)**	0.19 (-0.21, 0.59)	0.13 (-0.27, 0.52)	0.30 (-0.70, 1.30)	0.09 (-0.37, 0.55)	0.60 (-0.56, 1.75)	-0.99 (-2.19, 0.22)	0.09 (-0.54, 0.73)	0.46 (-0.05, 0.98)	-0.01 (-1.53, 1.51)	-0.27 (-0.87, 0.32)
MP	**0.65 (0.23, 1.06)**	0.40 (-0.04, 0.83)	0.33 (-0.09, 0.76)	0.50 (-0.52, 1.53)	0.30 (-0.13, 0.73)	0.80 (-0.39, 1.99)	-0.78 (-2.00, 0.44)	0.30 (-0.36, 0.96)	**0.67 (0.11, 1.23)**	0.20 (-1.34, 1.73)	-0.07 (-0.69, 0.56)
Casein	0.65 (-0.16, 1.45)	0.40 (-0.40, 1.19)	0.34 (-0.49, 1.17)	0.51 (-0.74, 1.75)	0.30 (-0.53, 1.14)	0.80 (-0.57, 2.18)	-0.78 (-1.90, 0.34)	0.30 (-0.56, 1.17)	0.67 (-0.22, 1.56)	0.20 (-1.49, 1.89)	-0.06 (-0.98, 0.85)
SP	**0.66 (0.15, 1.18)**	0.41 (-0.13, 0.96)	0.35 (-0.19, 0.90)	0.52 (-0.55, 1.60)	0.32 (-0.27, 0.91)	0.82 (-0.41, 2.05)	-0.76 (-2.02, 0.50)	0.32 (-0.41, 1.05)	**0.69 (0.12, 1.25)**	0.21 (-1.35, 1.78)	-0.05 (-0.72, 0.62)
Collagen	0.57 (-0.36, 1.49)	0.32 (-0.64, 1.28)	0.26 (-0.70, 1.22)	0.43 (-0.91, 1.76)	0.22 (-0.77, 1.21)	0.73 (-0.72, 2.17)	-0.86 (-2.35, 0.63)	0.22 (-0.86, 1.31)	0.59 (-0.43, 1.61)	0.12 (-1.64, 1.87)	-0.14 (-1.20, 0.92)
Meat	0.81 (-0.37, 2.00)	0.56 (-0.62, 1.75)	0.50 (-0.70, 1.71)	0.67 (-0.85, 2.19)	0.47 (-0.74, 1.67)	0.97 (-0.66, 2.60)	-0.61 (-2.26, 1.03)	0.47 (-0.81, 1.75)	0.83 (-0.41, 2.08)	0.36 (-1.54, 2.26)	0.10 (-1.17, 1.37)
DP	**0.91 (0.51, 1.30)**	**0.66 (0.25, 1.06)**	**0.60 (0.15, 1.04)**	0.77 (-0.26, 1.79)	**0.56 (0.10, 1.02)**	1.06 (-0.12, 2.24)	-0.52 (-1.72, 0.68)	0.56 (-0.07, 1.19)	**0.93 (0.38, 1.48)**	0.46 (-1.08, 1.99)	0.19 (-0.40, 0.79)
RET	**0.30 (0.09, 0.52)**	0.06 (-0.29, 0.40)	-0.01 (-0.30, 0.28)	0.16 (-0.80, 1.12)	-0.04 (-0.45, 0.37)	0.46 (-0.67, 1.60)	-1.12 (-2.31, 0.07)	-0.04 (-0.64, 0.56)	0.33 (-0.12, 0.77)	-0.15 (-1.64, 1.35)	-0.41 (-0.97, 0.15)
AET	0.12 (-0.86, 1.09)	-0.13 (-1.14, 0.87)	-0.19 (-1.15, 0.76)	-0.02 (-0.84, 0.79)	-0.23 (-1.26, 0.80)	0.27 (-1.21, 1.75)	-1.31 (-2.83, 0.21)	-0.23 (-1.35, 0.89)	0.14 (-0.90, 1.18)	-0.33 (-1.48, 0.81)	-0.60 (-1.70, 0.50)
MET	**0.68 (0.36, 1.01)**	**0.43 (0.16, 0.71)**	0.37 (-0.01, 0.75)	0.54 (-0.46, 1.54)	0.34 (-0.03, 0.70)	0.84 (-0.32, 2.00)	-0.75 (-1.92, 0.43)	0.34 (-0.20, 0.87)	**0.70 (0.20, 1.20)**	0.23 (-1.29, 1.75)	-0.03 (-0.56, 0.50)
RC	**1.02 (0.78, 1.26)**	**0.77 (0.49, 1.05)**	**0.71 (0.40, 1.02)**	0.88 (-0.10, 1.86)	**0.67 (0.32, 1.03)**	**1.18 (0.04, 2.32)**	-0.41 (-1.58, 0.76)	**0.67 (0.11, 1.24)**	**1.04 (0.59, 1.49)**	0.57 (-0.93, 2.07)	0.31 (-0.21, 0.82)
	Relative effects of NMA (column compared with row)										

To be continued.

Table S7. Continued.

	Direct evidence of pairwise meta-analyses (row compared with column)									
	Collagen+RET	Collagen+MET	Meat+RET	Meat+MET	DP+RET	DP+AET	DP+MET	Rice+RET	Rice+MET	WP
WP+RET	-0.17 (-1.41, 1.08)	.	.	.	-0.34 (-1.44, 0.76)	.	3.22 (1.89, 4.56)	.	.	0.33 (-0.01, 0.68)
WP+MET	.	.	.	.	-0.85 (-1.94, 0.24)	**1.24 (0.15, 2.33)**	-0.26 (-1.62, 1.10)	.	0.28 (-0.89, 1.45)	0.31 (-0.93, 1.55)
MP+RET	.	.	.	.	.	.	.	0.74 (-0.67, 2.14)	.	.
MP+AET	.	.	.	.	.	.	.	.	.	.
MP+MET	.	.	.	.	.	.	-0.00 (-1.13, 1.13)	.	0.00 (-1.07, 1.07)	.
Casein+RET	.	.	.	.	.	.	.	.	.	.
Casein+AET	.	.	.	.	.	-0.75 (-1.95, 0.45)	.	.	.	.
Casein+MET	.	.	.	.	.	.	.	.	.	.
SP+RET	.	.	.	.	.	.	.	**1.47 (0.02, 2.92)**	.	.
SP+AET	.	.	.	.	.	.	.	.	.	.
SP+MET	.	.	.	.	.	.	-0.48 (-2.10, 1.14)	.	.	.
Collagen+RET		.	.	.	.	.	.	.	.	.
Collagen+MET	0.47 (-1.67, 2.62)		.	.	.	.	.	.	.	.
Meat+RET	0.55 (-1.18, 2.27)	0.07 (-2.03, 2.17)		.	.	.	.	.	.	.
Meat+MET	0.62 (-0.89, 2.13)	0.15 (-1.76, 2.05)	0.08 (-1.38, 1.53)		.	.	0.38 (-0.71, 1.46)	.	.	.
DP+RET	0.60 (-0.71, 1.91)	0.13 (-1.63, 1.88)	0.06 (-1.19, 1.30)	-0.02 (-0.90, 0.86)		0.25 (-0.90, 1.41)	-0.00 (-1.08, 1.08)	.	.	.
DP+AET	0.87 (-0.51, 2.24)	0.39 (-1.41, 2.20)	0.32 (-0.99, 1.64)	0.25 (-0.73, 1.22)	0.27 (-0.32, 0.85)		-0.57 (-1.84, 0.70)	.	.	.
DP+MET	0.67 (-0.63, 1.97)	0.20 (-1.54, 1.94)	0.12 (-1.10, 1.35)	0.05 (-0.77, 0.86)	0.07 (-0.33, 0.47)	-0.20 (-0.77, 0.38)		.	.	.
Rice+RET	1.40 (-0.38, 3.19)	0.93 (-1.21, 3.07)	0.86 (-0.87, 2.59)	0.78 (-0.73, 2.29)	0.80 (-0.50, 2.11)	0.54 (-0.84, 1.91)	0.73 (-0.56, 2.03)		.	.
Rice+MET	0.60 (-0.92, 2.11)	0.12 (-1.78, 2.03)	0.05 (-1.40, 1.51)	-0.02 (-1.18, 1.14)	-0.00 (-0.89, 0.89)	-0.27 (-1.24, 0.70)	-0.07 (-0.93, 0.79)	-0.81 (-2.32, 0.71)		.
WP	0.61 (-0.68, 1.89)	0.13 (-1.62, 1.89)	0.06 (-1.16, 1.28)	-0.01 (-0.89, 0.86)	0.01 (-0.44, 0.45)	-0.26 (-0.87, 0.35)	-0.06 (-0.46, 0.33)	-0.80 (-2.09, 0.49)	0.01 (-0.87, 0.89)	
MP	0.81 (-0.50, 2.13)	0.34 (-1.42, 2.10)	0.27 (-0.98, 1.51)	0.19 (-0.70, 1.09)	0.21 (-0.28, 0.70)	-0.06 (-0.70, 0.59)	0.14 (-0.30, 0.58)	-0.59 (-1.90, 0.71)	0.21 (-0.67, 1.09)	0.21 (-0.26, 0.67)
Casein	0.82 (-0.67, 2.30)	0.34 (-1.54, 2.22)	0.27 (-1.16, 1.70)	0.19 (-0.92, 1.31)	0.21 (-0.61, 1.04)	-0.05 (-0.86, 0.76)	0.15 (-0.66, 0.95)	-0.59 (-2.07, 0.89)	0.22 (-0.90, 1.34)	0.21 (-0.62, 1.04)
SP	0.83 (-0.52, 2.18)	0.36 (-1.43, 2.15)	0.29 (-0.99, 1.56)	0.21 (-0.74, 1.16)	0.23 (-0.35, 0.81)	-0.04 (-0.75, 0.68)	0.16 (-0.38, 0.70)	-0.57 (-1.90, 0.76)	0.23 (-0.72, 1.19)	0.23 (-0.33, 0.78)
Collagen	0.74 (-0.82, 2.29)	0.26 (-1.70, 2.22)	0.19 (-1.31, 1.69)	0.12 (-1.12, 1.35)	0.14 (-0.85, 1.12)	-0.13 (-1.20, 0.94)	0.07 (-0.90, 1.03)	-0.67 (-2.23, 0.89)	0.14 (-1.10, 1.38)	0.13 (-0.80, 1.06)
Meat	0.98 (-0.74, 2.70)	0.51 (-1.57, 2.58)	0.43 (-1.24, 2.11)	0.36 (-0.88, 1.60)	0.38 (-0.83, 1.59)	0.11 (-1.16, 1.39)	0.31 (-0.87, 1.49)	-0.43 (-2.15, 1.30)	0.38 (-1.04, 1.80)	0.37 (-0.83, 1.58)
DP	1.07 (-0.23, 2.38)	0.60 (-1.15, 2.35)	0.53 (-0.71, 1.77)	0.45 (-0.42, 1.32)	**0.47 (0.06, 0.88)**	0.21 (-0.39, 0.80)	**0.40 (0.04, 0.76)**	-0.33 (-1.64, 0.97)	0.48 (-0.41, 1.36)	**0.47 (0.04, 0.90)**
RET	0.47 (-0.80, 1.74)	-0.00 (-1.74, 1.74)	-0.07 (-1.25, 1.10)	-0.15 (-1.00, 0.70)	-0.13 (-0.52, 0.26)	-0.40 (-0.98, 0.18)	-0.20 (-0.54, 0.15)	-0.93 (-2.20, 0.33)	-0.13 (-0.98, 0.73)	-0.13 (-0.44, 0.17)
AET	0.28 (-1.30, 1.87)	-0.19 (-2.17, 1.79)	-0.26 (-1.78, 1.25)	-0.34 (-1.61, 0.94)	-0.32 (-1.34, 0.71)	-0.58 (-1.69, 0.52)	-0.39 (-1.39, 0.62)	-1.12 (-2.69, 0.45)	-0.31 (-1.59, 0.96)	-0.32 (-1.32, 0.67)
MET	0.85 (-0.44, 2.14)	0.38 (-1.34, 2.09)	0.30 (-0.92, 1.52)	0.23 (-0.60, 1.06)	0.25 (-0.14, 0.64)	-0.02 (-0.58, 0.54)	0.18 (-0.12, 0.48)	-0.56 (-1.84, 0.73)	0.25 (-0.58, 1.09)	0.24 (-0.13, 0.62)
RC	1.19 (-0.08, 2.46)	0.71 (-1.02, 2.44)	0.64 (-0.56, 1.84)	0.57 (-0.26, 1.39)	**0.59 (0.25, 0.92)**	0.32 (-0.22, 0.86)	**0.52 (0.24, 0.79)**	-0.22 (-1.49, 1.05)	0.59 (-0.24, 1.42)	**0.58 (0.27, 0.89)**
	Relative effects of NMA (column compared with row)									

To be continued.

Table S7. Continued.

	Direct evidence of pairwise meta-analyses (row compared with column)									
	MP	Casein	SP	Collagen	Meat	DP	RET	AET	MET	RC
WP+RET	.	.	.	0.32 (-0.79, 1.42)	.	.	0.18 (-0.06, 0.42)	.	.	**1.08 (0.76, 1.40)**
WP+MET	.	.	.	.	.	.	.	.	**0.36 (0.00, 0.71)**	**1.03 (0.59, 1.47)**
MP+RET	0.18 (-0.43, 0.78)	.	.	.	.	.	0.08 (-0.27, 0.43)	-0.08 (-1.29, 1.12)	.	0.35 (-0.18, 0.87)
MP+AET	.	.	.	.	.	.	0.27 (-0.94, 1.48)	-0.02 (-0.84, 0.79)	.	.
MP+MET	0.28 (-0.28, 0.84)	.	.	.	.	.	.	.	0.30 (-0.28, 0.89)	0.25 (-0.30, 0.81)
Casein+RET	.	.	.	.	.	.	.	.	.	.
Casein+AET	.	-0.75 (-1.95, 0.45)	.	.	.	.	.	.	.	.
Casein+MET	.	0.10 (-1.03, 1.22)	.	.	.	.	.	.	0.30 (-0.28, 0.87)	0.38 (-0.74, 1.51)
SP+RET	.	.	**0.77 (0.10, 1.45)**	.	.	.	0.27 (-0.26, 0.79)	.	.	**1.23 (0.64, 1.81)**
SP+AET	.	.	.	.	.	.	.	-0.33 (-1.48, 0.81)	.	.
SP+MET	.	.	0.04 (-0.99, 1.07)	.	.	-0.48 (-2.16, 1.20)	.	.	0.10 (-0.56, 0.75)	0.20 (-0.42, 0.82)
Collagen+RET	.	.	.	.	.	.	.	.	.	.
Collagen+MET	.	.	.	.	.	.	.	.	0.38 (-1.34, 2.09)	.
Meat+RET	.	.	.	.	.	.	-0.07 (-1.25, 1.10)	.	.	.
Meat+MET	.	.	.	.	0.10 (-1.25, 1.46)	.	.	.	-0.03 (-1.43, 1.36)	0.02 (-1.40, 1.44)
DP+RET	.	.	.	.	.	0.42 (-0.23, 1.06)	0.08 (-1.08, 1.24)	.	.	0.37 (-0.05, 0.80)
DP+AET	.	-0.00 (-1.19, 1.19)	.	.	.	0.43 (-0.85, 1.71)	.	.	.	**0.92 (0.05, 1.79)**
DP+MET	.	.	0.48 (-1.20, 2.16)	.	.	**0.50 (0.05, 0.96)**	.	.	0.07 (-0.35, 0.48)	**0.57 (0.23, 0.90)**
Rice+RET	.	.	.	.	.	.	.	.	.	.
Rice+MET	.	.	.	.	.	.	.	.	.	.
WP	.	.	.	-0.01 (-1.12, 1.11)	.	0.37 (-0.99, 1.74)	-0.04 (-0.44, 0.37)	.	-0.15 (-1.35, 1.04)	**0.54 (0.14, 0.95)**
MP		.	.	.	.	0.15 (-0.98, 1.28)	0.23 (-0.58, 1.03)	.	-0.01 (-0.78, 0.77)	0.33 (-0.17, 0.83)
Casein	0.00 (-0.85, 0.85)		.	.	.	.	.	.	0.00 (-1.13, 1.13)	0.29 (-0.84, 1.42)
SP	0.02 (-0.58, 0.61)	0.02 (-0.89, 0.92)		.	.	-0.48 (-2.21, 1.26)	-0.42 (-1.11, 0.27)	.	-0.09 (-1.10, 0.92)	0.54 (-0.03, 1.10)
Collagen	-0.08 (-1.07, 0.92)	-0.08 (-1.28, 1.13)	-0.10 (-1.13, 0.94)		.	.	.	.	.	0.04 (-1.09, 1.17)
Meat	0.17 (-1.05, 1.38)	0.16 (-1.23, 1.55)	0.15 (-1.11, 1.41)	0.24 (-1.24, 1.73)		.	.	.	-0.14 (-1.53, 1.25)	-0.08 (-1.50, 1.33)
DP	0.26 (-0.21, 0.73)	0.26 (-0.57, 1.08)	0.24 (-0.33, 0.81)	0.34 (-0.64, 1.32)	0.09 (-1.11, 1.30)		0.00 (-1.19, 1.19)	.	-0.03 (-0.63, 0.58)	0.21 (-0.24, 0.67)
RET	-0.34 (-0.74, 0.06)	-0.34 (-1.15, 0.46)	-0.36 (-0.86, 0.14)	-0.26 (-1.20, 0.67)	-0.51 (-1.69, 0.68)	**-0.60 (-0.99, -0.22)**		-0.32 (-1.53, 0.88)	.	**0.66 (0.36, 0.96)**
AET	-0.53 (-1.55, 0.49)	-0.53 (-1.77, 0.71)	-0.55 (-1.62, 0.52)	-0.45 (-1.78, 0.88)	-0.70 (-2.21, 0.82)	-0.79 (-1.81, 0.23)	-0.19 (-1.14, 0.77)		.	.
MET	0.04 (-0.37, 0.45)	0.03 (-0.74, 0.81)	0.02 (-0.50, 0.54)	0.11 (-0.84, 1.07)	-0.13 (-1.30, 1.04)	-0.22 (-0.59, 0.14)	**0.38 (0.06, 0.70)**	0.57 (-0.43, 1.56)		0.26 (-0.04, 0.56)
RC	**0.37 (0.00, 0.75)**	0.37 (-0.40, 1.15)	0.36 (-0.13, 0.84)	0.45 (-0.48, 1.38)	0.21 (-0.96, 1.37)	0.11 (-0.22, 0.44)	**0.72 (0.48, 0.95)**	0.90 (-0.07, 1.88)	**0.34 (0.10, 0.58)**	
	Relative effects of NMA (column compared with row)									

Pairwise (upper right portion) and network (lower left portion) meta-analysis results are presented for mean change (from baseline) in global function. Effect estimation is presented in standardized mean difference with 95% confidence interval. Significant results ($p < 0.05$) are marked in bold.

AET, aerobic exercise training; DP, dietary protein; Ins-PS, insect protein supplement; MET, multicomponent exercise training; MP, milk protein; RET, resistance exercise training; SP, soy protein; WP, whey protein; RC, regular care.

Table S8. Direct and network estimates for chair stand.

	Direct evidence of pairwise meta-analyses (row compared with column)								
	WP+RET	WP+AET	WP+MET	MP+RET	MP+AET	MP+MET	Casein+RET	Casein+MET	SP+RET
WP+RET		.	.	-0.27 (-1.03, 0.49)	.	.	-0.16 (-1.19, 0.87)	.	-0.00 (-1.11, 1.11)
WP+AET	-0.13 (-1.28, 1.02)		.	.	.	.	.	.	.
WP+MET	-0.12 (-0.64, 0.39)	0.01 (-1.19, 1.20)		.	.	-0.01 (-1.01, 0.99)	.	-0.15 (-1.32, 1.02)	.
MP+RET	0.08 (-0.26, 0.41)	0.21 (-0.96, 1.38)	0.20 (-0.34, 0.75)		.	-0.67 (-1.76, 0.41)	.	.	-0.58 (-1.95, 0.79)
MP+AET	-0.17 (-1.70, 1.36)	-0.04 (-1.54, 1.46)	-0.04 (-1.61, 1.52)	-0.25 (-1.79, 1.30)		.	.	.	.
MP+MET	-0.08 (-0.64, 0.49)	0.06 (-1.17, 1.28)	0.05 (-0.53, 0.63)	-0.15 (-0.72, 0.42)	0.09 (-1.49, 1.68)		.	.	.
Casein+RET	-0.16 (-1.19, 0.87)	-0.03 (-1.57, 1.52)	-0.03 (-1.18, 1.12)	-0.24 (-1.32, 0.84)	0.01 (-1.84, 1.86)	-0.08 (-1.26, 1.09)		.	.
Casein+MET	0.18 (-0.58, 0.95)	0.32 (-1.01, 1.64)	0.31 (-0.43, 1.05)	0.11 (-0.68, 0.89)	0.35 (-1.31, 2.02)	0.26 (-0.58, 1.10)	0.34 (-0.94, 1.62)		.
SP+RET	0.09 (-0.53, 0.71)	0.22 (-1.06, 1.50)	0.22 (-0.54, 0.97)	0.02 (-0.62, 0.65)	0.26 (-1.37, 1.89)	0.17 (-0.62, 0.96)	0.25 (-0.95, 1.45)	-0.09 (-1.04, 0.85)	
SP+MET	0.29 (-0.32, 0.91)	0.42 (-0.82, 1.66)	0.42 (-0.24, 1.07)	0.22 (-0.43, 0.86)	0.46 (-1.14, 2.06)	0.37 (-0.36, 1.09)	0.45 (-0.75, 1.65)	0.11 (-0.75, 0.97)	0.20 (-0.63, 1.03)
Collagen+RET	-0.03 (-1.20, 1.14)	0.10 (-1.54, 1.74)	0.09 (-1.18, 1.37)	-0.11 (-1.32, 1.11)	0.14 (-1.79, 2.07)	0.05 (-1.25, 1.34)	0.13 (-1.43, 1.69)	-0.21 (-1.61, 1.18)	-0.12 (-1.45, 1.20)
Meat+RET	0.56 (-1.02, 2.15)	0.69 (-1.25, 2.63)	0.69 (-0.96, 2.33)	0.49 (-1.08, 2.05)	0.73 (-1.46, 2.92)	0.64 (-1.02, 2.29)	0.72 (-1.17, 2.61)	0.38 (-1.36, 2.11)	0.47 (-1.10, 2.04)
Meat+MET	-0.04 (-1.15, 1.07)	0.09 (-1.46, 1.64)	0.08 (-1.05, 1.21)	-0.12 (-1.24, 1.01)	0.13 (-1.72, 1.98)	0.03 (-1.14, 1.21)	0.12 (-1.40, 1.63)	-0.23 (-1.49, 1.04)	-0.13 (-1.38, 1.11)
DP+RET	0.22 (-0.24, 0.68)	0.35 (-0.83, 1.53)	0.34 (-0.22, 0.91)	0.14 (-0.35, 0.64)	0.39 (-1.16, 1.94)	0.29 (-0.33, 0.92)	0.38 (-0.75, 1.51)	0.04 (-0.76, 0.83)	0.13 (-0.59, 0.85)
DP+MET	-0.04 (-0.54, 0.45)	0.09 (-1.10, 1.28)	0.08 (-0.45, 0.62)	-0.12 (-0.65, 0.41)	0.13 (-1.43, 1.69)	0.03 (-0.59, 0.66)	0.12 (-1.02, 1.26)	-0.23 (-1.01, 0.56)	-0.13 (-0.88, 0.61)
Rice+RET	0.21 (-0.99, 1.41)	0.34 (-1.30, 1.98)	0.34 (-0.94, 1.61)	0.14 (-1.04, 1.32)	0.38 (-1.54, 2.31)	0.29 (-1.00, 1.58)	0.37 (-1.21, 1.95)	0.03 (-1.37, 1.42)	0.12 (-1.06, 1.30)
Rice+MET	0.13 (-1.00, 1.26)	0.26 (-1.31, 1.82)	0.25 (-0.89, 1.39)	0.05 (-1.08, 1.18)	0.30 (-1.57, 2.16)	0.20 (-0.78, 1.18)	0.29 (-1.24, 1.81)	-0.06 (-1.35, 1.23)	0.03 (-1.22, 1.29)
WP	**0.65 (0.22, 1.07)**	0.78 (-0.40, 1.96)	**0.77 (0.21, 1.33)**	**0.57 (0.09, 1.05)**	0.82 (-0.74, 2.37)	**0.72 (0.10, 1.35)**	0.81 (-0.30, 1.92)	0.46 (-0.34, 1.26)	0.56 (-0.15, 1.27)
MP	0.69 (-0.08, 1.47)	0.83 (-0.52, 2.17)	0.82 (-0.04, 1.67)	0.62 (-0.14, 1.38)	0.86 (-0.82, 2.54)	0.77 (-0.12, 1.66)	0.85 (-0.43, 2.14)	0.51 (-0.51, 1.53)	0.60 (-0.35, 1.55)
Casein	0.50 (-0.45, 1.44)	0.63 (-0.80, 2.06)	0.62 (-0.33, 1.57)	0.42 (-0.54, 1.38)	0.67 (-1.09, 2.42)	0.57 (-0.44, 1.59)	0.66 (-0.74, 2.05)	0.31 (-0.65, 1.28)	0.41 (-0.69, 1.50)
SP	0.81 (-0.25, 1.88)	0.95 (-0.57, 2.46)	0.94 (-0.14, 2.02)	0.74 (-0.34, 1.82)	0.98 (-0.84, 2.80)	0.89 (-0.24, 2.02)	0.97 (-0.50, 2.45)	0.63 (-0.59, 1.85)	0.72 (-0.47, 1.92)
DP	**0.55 (0.04, 1.06)**	0.68 (-0.52, 1.88)	**0.67 (0.08, 1.27)**	0.47 (-0.07, 1.01)	0.72 (-0.85, 2.29)	0.63 (-0.03, 1.28)	0.71 (-0.44, 1.86)	0.37 (-0.45, 1.18)	0.46 (-0.30, 1.21)
RET	0.17 (-0.06, 0.40)	0.30 (-0.85, 1.45)	0.30 (-0.21, 0.81)	0.10 (-0.19, 0.38)	0.34 (-1.19, 1.87)	0.25 (-0.31, 0.80)	0.33 (-0.72, 1.38)	-0.01 (-0.77, 0.75)	0.08 (-0.52, 0.69)
AET	-0.12 (-1.27, 1.03)	0.01 (-1.10, 1.12)	0.00 (-1.19, 1.20)	-0.20 (-1.37, 0.97)	0.05 (-0.96, 1.06)	-0.05 (-1.27, 1.18)	0.04 (-1.51, 1.58)	-0.31 (-1.63, 1.02)	-0.21 (-1.49, 1.07)
MET	0.42 (-0.01, 0.85)	0.55 (-0.61, 1.71)	**0.54 (0.13, 0.96)**	0.34 (-0.13, 0.81)	0.59 (-0.95, 2.13)	0.49 (-0.06, 1.05)	0.58 (-0.54, 1.69)	0.23 (-0.45, 0.92)	0.33 (-0.38, 1.03)
RC	**0.67 (0.38, 0.97)**	0.81 (-0.31, 1.92)	**0.80 (0.36, 1.24)**	**0.60 (0.25, 0.95)**	0.84 (-0.66, 2.35)	**0.75 (0.24, 1.25)**	0.83 (-0.24, 1.90)	0.49 (-0.22, 1.20)	0.58 (-0.05, 1.21)
	Relative effects of NMA (column compared with row)								

To be continued.

Table S8. Continued.

	Direct evidence of pairwise meta-analyses (row compared with column)								
	SP+MET	Collagen+RET	Meat+RET	Meat+MET	DP+RET	DP+MET	Rice+RET	Rice+MET	WP
WP+RET	.	-0.03 (-1.20, 1.14)	.	.	.	.	.	.	**1.02 (0.46, 1.59)**
WP+AET	.	.	.	.	.	.	.	.	.
WP+MET	.	.	.	.	.	0.22 (-1.07, 1.51)	.	.	0.31 (-0.85, 1.48)
MP+RET	.	.	.	.	.	.	-0.16 (-1.48, 1.17)	.	.
MP+AET	.	.	.	.	.	.	.	.	.
MP+MET	.	.	.	.	.	.	.	0.20 (-0.78, 1.18)	.
Casein+RET	.	.	.	.	.	.	.	.	.
Casein+MET	.	.	.	.	.	.	.	.	.
SP+RET	.	.	.	.	.	.	0.42 (-0.91, 1.75)	.	.
SP+MET		.	.	.	.	.	.	.	.
Collagen+RET	-0.32 (-1.65, 1.00)		.	.	.	.	.	.	.
Meat+RET	0.27 (-1.41, 1.95)	0.59 (-1.38, 2.56)		.	.	.	-0.35 (-1.38, 0.69)	.	.
Meat+MET	-0.33 (-1.53, 0.86)	-0.01 (-1.63, 1.60)	-0.60 (-2.52, 1.31)		.	-0.00 (-1.00, 1.00)	.	.	.
DP+RET	-0.07 (-0.73, 0.59)	0.25 (-1.01, 1.51)	-0.34 (-1.97, 1.28)	0.26 (-0.86, 1.38)		0.23 (-0.76, 1.22)	.	.	.
DP+MET	-0.33 (-0.99, 0.32)	-0.01 (-1.28, 1.26)	-0.60 (-2.24, 1.03)	0.00 (-1.00, 1.00)	-0.26 (-0.78, 0.26)		.	.	.
Rice+RET	-0.08 (-1.40, 1.24)	0.24 (-1.43, 1.92)	-0.35 (-1.38, 0.69)	0.25 (-1.36, 1.87)	-0.01 (-1.26, 1.25)	0.25 (-1.01, 1.52)		.	.
Rice+MET	-0.17 (-1.39, 1.05)	0.16 (-1.47, 1.78)	-0.44 (-2.36, 1.49)	0.17 (-1.37, 1.70)	-0.09 (-1.26, 1.07)	0.17 (-1.00, 1.33)	-0.09 (-1.71, 1.53)		.
WP	0.36 (-0.31, 1.02)	0.68 (-0.57, 1.92)	0.09 (-1.54, 1.71)	0.69 (-0.45, 1.83)	0.43 (-0.11, 0.96)	**0.69 (0.14, 1.24)**	0.43 (-0.81, 1.68)	0.52 (-0.64, 1.68)	
MP	0.40 (-0.52, 1.32)	0.72 (-0.68, 2.13)	0.13 (-1.60, 1.87)	0.74 (-0.56, 2.03)	0.47 (-0.35, 1.30)	0.74 (-0.10, 1.57)	0.48 (-0.91, 1.87)	0.57 (-0.75, 1.89)	0.05 (-0.78, 0.87)
Casein	0.21 (-0.82, 1.23)	0.53 (-0.98, 2.03)	-0.06 (-1.89, 1.76)	0.54 (-0.85, 1.93)	0.28 (-0.69, 1.25)	0.54 (-0.43, 1.51)	0.29 (-1.22, 1.79)	0.37 (-1.04, 1.78)	-0.15 (-1.12, 0.83)
SP	0.52 (-0.53, 1.58)	0.84 (-0.74, 2.42)	0.25 (-1.63, 2.14)	0.86 (-0.62, 2.33)	0.59 (-0.49, 1.68)	0.86 (-0.23, 1.94)	0.60 (-0.98, 2.18)	0.69 (-0.81, 2.18)	0.17 (-0.92, 1.26)
DP	0.26 (-0.43, 0.94)	0.58 (-0.70, 1.86)	-0.01 (-1.65, 1.63)	0.59 (-0.54, 1.72)	0.33 (-0.22, 0.88)	**0.59 (0.05, 1.13)**	0.34 (-0.94, 1.61)	0.42 (-0.76, 1.60)	-0.10 (-0.65, 0.46)
RET	-0.12 (-0.73, 0.49)	0.20 (-0.99, 1.39)	-0.39 (-1.97, 1.19)	0.21 (-0.90, 1.32)	-0.05 (-0.49, 0.40)	0.21 (-0.27, 0.70)	-0.04 (-1.23, 1.15)	0.05 (-1.08, 1.17)	**-0.48 (-0.90, -0.05)**
AET	-0.41 (-1.65, 0.83)	-0.09 (-1.73, 1.55)	-0.68 (-2.62, 1.26)	-0.08 (-1.63, 1.47)	-0.34 (-1.52, 0.84)	-0.08 (-1.27, 1.11)	-0.33 (-1.97, 1.31)	-0.25 (-1.81, 1.32)	-0.77 (-1.95, 0.41)
MET	0.13 (-0.43, 0.68)	0.45 (-0.80, 1.70)	-0.14 (-1.76, 1.48)	0.46 (-0.63, 1.55)	0.20 (-0.29, 0.69)	**0.46 (0.01, 0.91)**	0.21 (-1.04, 1.45)	0.29 (-0.83, 1.42)	-0.23 (-0.72, 0.26)
RC	0.38 (-0.16, 0.93)	0.70 (-0.50, 1.91)	0.11 (-1.48, 1.70)	0.71 (-0.36, 1.79)	**0.45 (0.08, 0.83)**	**0.71 (0.31, 1.12)**	0.46 (-0.74, 1.67)	0.55 (-0.55, 1.65)	0.03 (-0.37, 0.42)
	Relative effects of NMA (column compared with row)								

To be continued.

Table S8. Continued.

	Direct evidence of pairwise meta-analyses (row compared with column)							
	MP	Casein	SP	DP	RET	AET	MET	RC
WP+RET	.	.	.	.	0.19 (-0.06, 0.44)	.	.	**0.82 (0.44, 1.20)**
WP+AET	.	.	.	.	.	0.01 (-1.10, 1.12)	.	0.81 (-0.31, 1.92)
WP+MET	.	.	.	.	.	.	**0.66 (0.16, 1.16)**	0.56 (-0.24, 1.35)
MP+RET	0.34 (-0.84, 1.52)	.	.	.	0.16 (-0.19, 0.50)	.	.	0.51 (-0.29, 1.32)
MP+AET	.	.	.	.	.	0.05 (-0.96, 1.06)	.	.
MP+MET	.	.	.	.	.	.	0.08 (-1.11, 1.26)	0.62 (-0.05, 1.30)
Casein+RET	.	.	.	.	.	.	.	.
Casein+MET	.	0.25 (-0.79, 1.29)	.	.	.	.	0.08 (-0.70, 0.86)	0.39 (-0.65, 1.43)
SP+RET	.	.	.	.	-0.09 (-0.85, 0.68)	.	.	0.30 (-0.74, 1.34)
SP+MET	.	.	0.39 (-0.84, 1.62)	.	.	.	0.25 (-0.43, 0.93)	0.33 (-0.30, 0.97)
Collagen+RET	.	.	.	.	.	.	.	.
Meat+RET	.	.	.	.	.	.	.	.
Meat+MET	.	.	.	.	.	.	.	.
DP+RET	.	.	.	-0.08 (-1.18, 1.01)	0.12 (-0.67, 0.90)	.	.	**0.43 (0.03, 0.83)**
DP+MET	.	.	.	0.33 (-0.42, 1.07)	.	.	0.38 (-0.22, 0.99)	**0.84 (0.37, 1.30)**
Rice+RET	.	.	.	.	.	.	.	.
Rice+MET	.	.	.	.	.	.	.	.
WP	.	.	.	0.85 (-0.47, 2.16)	**-0.72 (-1.29, -0.16)**	.	0.07 (-1.05, 1.18)	-0.04 (-0.51, 0.42)
MP		.	.	-0.22 (-1.27, 0.83)	.	.	.	-0.27 (-1.32, 0.77)
Casein	-0.20 (-1.36, 0.97)		.	.	.	.	-0.11 (-1.15, 0.93)	0.14 (-0.90, 1.18)
SP	0.12 (-1.14, 1.38)	0.32 (-1.02, 1.66)		.	.	.	-0.24 (-1.48, 1.01)	-0.41 (-1.63, 0.81)
DP	-0.14 (-0.93, 0.64)	0.05 (-0.94, 1.04)	-0.26 (-1.37, 0.84)		0.25 (-0.86, 1.36)	.	-0.14 (-0.89, 0.61)	0.05 (-0.48, 0.58)
RET	-0.52 (-1.29, 0.24)	-0.33 (-1.27, 0.61)	-0.64 (-1.70, 0.42)	-0.38 (-0.88, 0.12)		.	.	**0.77 (0.38, 1.17)**
AET	-0.81 (-2.16, 0.53)	-0.62 (-2.05, 0.81)	-0.94 (-2.45, 0.58)	-0.67 (-1.87, 0.53)	-0.29 (-1.44, 0.86)		.	0.79 (-0.32, 1.91)
MET	-0.28 (-1.08, 0.53)	-0.08 (-0.97, 0.81)	-0.40 (-1.42, 0.63)	-0.13 (-0.64, 0.37)	0.25 (-0.18, 0.67)	0.54 (-0.62, 1.70)		**0.39 (0.00, 0.78)**
RC	-0.02 (-0.77, 0.73)	0.18 (-0.72, 1.08)	-0.14 (-1.16, 0.88)	0.12 (-0.32, 0.57)	**0.50 (0.21, 0.79)**	0.79 (-0.32, 1.91)	0.26 (-0.07, 0.59)	
	Relative effects of NMA (column compared with row)							

Pairwise (upper right portion) and network (lower left portion) meta-analysis results are presented for mean change (from baseline) in global function. Effect estimation is presented in standardized mean difference with 95% confidence interval. Significant results (p <0.05) are marked in bold.

AET, aerobic exercise training; DP, dietary protein; Ins-PS, insect protein supplement; MET, multicomponent exercise training; MP, milk protein; RET, resistance exercise training; SP, soy protein; WP, whey protein; RC, regular care.

Table S9. Direct and network estimates for timed up-and-go performance.

	Direct evidence of pairwise meta-analyses (row compared with column)									
	WP+RET	WP+MET	MP+RET	MP+AET	MP+MET	Casein+MET	SP+RET	SP+MET	Meat+RET	Meat+MET
WP+RET		.	.	.	.	.	0.48 (-0.46, 1.42)	.	.	.
WP+MET	-0.23 (-0.70, 0.25)		.	.	-0.06 (-0.66, 0.55)	-0.06 (-1.01, 0.89)	.	.	.	.
MP+RET	0.21 (-0.25, 0.67)	0.44 (-0.14, 1.01)		0.03 (-0.93, 0.99)	.	.	-1.25 (-2.51, 0.02)	.	.	.
MP+AET	0.39 (-0.42, 1.19)	0.61 (-0.28, 1.50)	0.17 (-0.60, 0.95)		.	.	.	.	.	.
MP+MET	-0.12 (-0.61, 0.36)	0.10 (-0.30, 0.51)	-0.34 (-0.91, 0.24)	-0.51 (-1.41, 0.39)		.	.	.	.	.
Casein+MET	-0.18 (-1.11, 0.75)	0.05 (-0.80, 0.89)	-0.39 (-1.37, 0.59)	-0.57 (-1.76, 0.63)	-0.06 (-0.96, 0.85)		.	.	.	.
SP+RET	0.03 (-0.50, 0.56)	0.26 (-0.39, 0.90)	-0.18 (-0.79, 0.43)	-0.35 (-1.26, 0.56)	0.16 (-0.49, 0.81)	0.21 (-0.81, 1.24)		.	.	.
SP+MET	0.24 (-0.64, 1.12)	0.47 (-0.44, 1.37)	0.03 (-0.91, 0.97)	-0.15 (-1.31, 1.01)	0.36 (-0.56, 1.28)	0.42 (-0.79, 1.63)	0.21 (-0.78, 1.19)		.	.
Meat+RET	0.38 (-0.64, 1.39)	0.60 (-0.45, 1.65)	0.16 (-0.89, 1.22)	-0.01 (-1.27, 1.25)	0.50 (-0.52, 1.52)	0.56 (-0.76, 1.88)	0.34 (-0.75, 1.43)	0.14 (-1.17, 1.44)		.
Meat+MET	-0.09 (-1.00, 0.83)	0.14 (-0.79, 1.07)	-0.30 (-1.27, 0.68)	-0.47 (-1.66, 0.72)	0.04 (-0.91, 0.98)	0.09 (-1.13, 1.32)	-0.12 (-1.13, 0.90)	-0.33 (-1.52, 0.87)	-0.46 (-1.79, 0.87)	
DP+RET	-0.05 (-0.47, 0.37)	0.18 (-0.31, 0.66)	-0.26 (-0.79, 0.27)	-0.43 (-1.29, 0.43)	0.08 (-0.43, 0.58)	0.13 (-0.80, 1.07)	-0.08 (-0.69, 0.52)	-0.29 (-1.17, 0.59)	-0.42 (-1.48, 0.63)	0.04 (-0.86, 0.94)
DP+MET	0.01 (-0.45, 0.48)	0.24 (-0.25, 0.73)	-0.20 (-0.76, 0.36)	-0.37 (-1.26, 0.51)	0.14 (-0.38, 0.65)	0.19 (-0.74, 1.12)	-0.02 (-0.65, 0.62)	-0.23 (-1.12, 0.67)	-0.36 (-1.43, 0.70)	0.10 (-0.69, 0.89)
Rice+RET	0.28 (-0.29, 0.85)	0.50 (-0.13, 1.13)	0.07 (-0.58, 0.71)	-0.11 (-1.05, 0.84)	0.40 (-0.17, 0.98)	0.46 (-0.56, 1.48)	0.25 (-0.45, 0.94)	0.04 (-0.96, 1.04)	-0.10 (-0.94, 0.74)	0.36 (-0.67, 1.39)
WP	0.29 (-0.33, 0.90)	0.51 (-0.13, 1.15)	0.07 (-0.63, 0.78)	-0.10 (-1.07, 0.87)	0.41 (-0.26, 1.08)	0.47 (-0.56, 1.49)	0.25 (-0.51, 1.01)	0.05 (-0.96, 1.05)	-0.09 (-1.24, 1.05)	0.37 (-0.66, 1.41)
MP	0.45 (-0.12, 1.02)	**0.68 (0.09, 1.26)**	0.24 (-0.38, 0.86)	0.07 (-0.86, 1.00)	0.58 (-0.01, 1.16)	0.63 (-0.36, 1.62)	0.42 (-0.29, 1.13)	0.21 (-0.75, 1.18)	0.08 (-1.03, 1.18)	0.54 (-0.45, 1.52)
DP	**0.61 (0.10, 1.12)**	**0.84 (0.30, 1.37)**	0.40 (-0.20, 0.99)	0.22 (-0.68, 1.13)	**0.73 (0.18, 1.29)**	0.79 (-0.17, 1.75)	0.58 (-0.09, 1.24)	0.37 (-0.55, 1.29)	0.23 (-0.85, 1.32)	0.70 (-0.20, 1.59)
RET	0.15 (-0.12, 0.42)	0.37 (-0.11, 0.86)	-0.06 (-0.48, 0.35)	-0.24 (-1.01, 0.54)	0.27 (-0.23, 0.77)	0.33 (-0.61, 1.26)	0.11 (-0.40, 0.63)	-0.09 (-0.98, 0.79)	-0.23 (-1.25, 0.80)	0.23 (-0.69, 1.16)
AET	0.33 (-0.47, 1.14)	0.56 (-0.33, 1.45)	0.12 (-0.65, 0.89)	-0.05 (-0.67, 0.57)	0.46 (-0.43, 1.35)	0.51 (-0.68, 1.71)	0.30 (-0.61, 1.21)	0.09 (-1.06, 1.25)	-0.04 (-1.30, 1.22)	0.42 (-0.77, 1.60)
MET	0.09 (-0.34, 0.53)	0.32 (-0.01, 0.64)	-0.12 (-0.66, 0.42)	-0.29 (-1.16, 0.58)	0.22 (-0.17, 0.60)	0.27 (-0.57, 1.11)	0.06 (-0.56, 0.67)	-0.15 (-1.04, 0.74)	-0.28 (-1.32, 0.75)	0.18 (-0.73, 1.08)
RC	0.28 (-0.03, 0.59)	**0.51 (0.12, 0.89)**	0.07 (-0.38, 0.52)	-0.11 (-0.92, 0.71)	0.40 (-0.01, 0.82)	0.46 (-0.43, 1.35)	0.25 (-0.29, 0.78)	0.04 (-0.78, 0.86)	-0.10 (-1.11, 0.92)	0.37 (-0.50, 1.23)
	Relative effects of NMA (column compared with row)									

To be continued.

Table S9. Continued.

	Direct evidence of pairwise meta-analyses (row compared with column)									
	DP+RET	DP+MET	Rice+RET	WP	MP	DP	RET	AET	MET	RC
WP+RET	.	.	0.17 (-0.72, 1.06)	0.50 (-0.45, 1.46)	.	.	0.15 (-0.15, 0.45)	.	.	0.19 (-0.21, 0.60)
WP+MET	.	.	.	-0.29 (-1.29, 0.71)	.	.	.	.	**0.51 (0.12, 0.89)**	0.07 (-0.55, 0.69)
MP+RET	.	.	0.21 (-0.97, 1.39)	.	0.32 (-0.91, 1.54)	.	-0.08 (-0.60, 0.45)	0.08 (-0.87, 1.04)	.	0.33 (-0.55, 1.20)
MP+AET	.	.	.	.	.	.	-0.27 (-1.23, 0.69)	-0.05 (-0.67, 0.57)	.	.
MP+MET	.	.	0.13 (-0.64, 0.91)	.	0.60 (-0.28, 1.47)	.	.	.	0.00 (-0.51, 0.51)	**0.81 (0.14, 1.48)**
Casein+MET	.	.	.	.	.	.	.	.	0.16 (-0.80, 1.12)	.
SP+RET	.	.	**1.46 (0.21, 2.70)**	.	.	.	-0.08 (-0.72, 0.55)	.	.	-0.03 (-0.88, 0.81)
SP+MET	.	.	.	.	.	.	.	.	.	0.04 (-0.78, 0.86)
Meat+RET	.	.	-0.10 (-0.94, 0.74)	.	.	.	.	.	.	.
Meat+MET	.	0.10 (-0.69, 0.89)	.	.	.	.	.	.	.	.
DP+RET		0.25 (-0.54, 1.04)	.	.	.	0.65 (-0.21, 1.50)	0.37 (-0.41, 1.15)	.	.	0.27 (-0.08, 0.61)
DP+MET	0.06 (-0.36, 0.49)		.	.	.	**0.78 (0.27, 1.30)**	.	.	0.01 (-0.60, 0.62)	0.10 (-0.31, 0.51)
Rice+RET	0.33 (-0.31, 0.96)	0.26 (-0.39, 0.92)		.	.	.	.	.	.	.
WP	0.33 (-0.32, 0.98)	0.27 (-0.39, 0.94)	0.01 (-0.77, 0.79)		.	.	-0.66 (-1.62, 0.30)	.	-0.26 (-1.21, 0.68)	0.06 (-0.63, 0.76)
MP	0.50 (-0.07, 1.07)	0.44 (-0.14, 1.02)	0.17 (-0.55, 0.90)	0.17 (-0.58, 0.91)		0.05 (-0.81, 0.90)	.	.	**-0.93 (-1.81, -0.05)**	0.17 (-0.44, 0.78)
DP	**0.66 (0.20, 1.12)**	**0.60 (0.18, 1.01)**	0.33 (-0.36, 1.02)	0.32 (-0.37, 1.02)	0.16 (-0.42, 0.74)		.	.	-0.19 (-1.11, 0.73)	0.19 (-0.44, 0.81)
RET	0.20 (-0.22, 0.61)	0.13 (-0.33, 0.60)	-0.13 (-0.72, 0.46)	-0.14 (-0.75, 0.48)	-0.30 (-0.88, 0.27)	-0.46 (-0.97, 0.05)		0.32 (-0.62, 1.27)	.	0.09 (-0.44, 0.63)
AET	0.38 (-0.47, 1.24)	0.32 (-0.56, 1.20)	0.06 (-0.89, 1.00)	0.05 (-0.92, 1.02)	-0.12 (-1.04, 0.81)	-0.28 (-1.18, 0.62)	0.19 (-0.58, 0.95)		.	.
MET	0.14 (-0.30, 0.58)	0.08 (-0.35, 0.51)	-0.19 (-0.79, 0.42)	-0.19 (-0.81, 0.42)	-0.36 (-0.90, 0.18)	**-0.52 (-1.00, -0.04)**	-0.06 (-0.50, 0.39)	-0.24 (-1.11, 0.62)		**0.51 (0.10, 0.91)**
RC	**0.33 (0.02, 0.64)**	0.27 (-0.09, 0.62)	0.00 (-0.57, 0.57)	-0.01 (-0.59, 0.57)	-0.17 (-0.67, 0.33)	-0.33 (-0.74, 0.08)	0.13 (-0.19, 0.46)	-0.05 (-0.87, 0.76)	0.19 (-0.14, 0.52)	
	Relative effects of NMA (column compared with row)									

Pairwise (upper right portion) and network (lower left portion) meta-analysis results are presented for mean change (from baseline) in global function. Effect estimation is presented in standardized mean difference with 95% confidence interval. Significant results ($p < 0.05$) are marked in bold.

AET, aerobic exercise training; DP, dietary protein; Ins-PS, insect protein supplement; MET, multicomponent exercise training; MP, milk protein; RET, resistance exercise training; SP, soy protein; WP, whey protein; RC, regular care.

Table S10. Direct and network estimates for global physical mobility (SPPB).

	Direct evidence of pairwise meta-analyses (row compared with column)									
	WP+RET	WP+AET	WP+MET	MP+RET	MP+AET	MP+MET	Casein+RET	Casein+MET	SP+MET	Collagen+RET
WP+RET		.	.	.	.	.	.	.	.	-0.00 (-1.22, 1.22)
WP+AET	-0.14 (-1.27, 1.00)		.	.	.	.	.	.	.	.
WP+MET	-0.06 (-0.77, 0.65)	0.08 (-1.19, 1.34)		.	.	.	.	.	.	.
MP+RET	0.36 (-0.20, 0.92)	0.50 (-0.72, 1.72)	0.42 (-0.37, 1.21)		.	.	.	.	.	.
MP+AET	**1.35 (0.12, 2.58)**	1.49 (-0.04, 3.01)	**1.41 (0.05, 2.78)**	0.99 (-0.33, 2.31)		.	.	.	.	.
MP+MET	0.13 (-0.60, 0.87)	0.27 (-1.01, 1.55)	0.19 (-0.57, 0.96)	-0.23 (-1.04, 0.58)	-1.22 (-2.60, 0.16)		.	.	.	.
Casein+RET	0.21 (-1.01, 1.43)	0.35 (-1.27, 1.96)	0.27 (-1.01, 1.54)	-0.15 (-1.42, 1.12)	-1.14 (-2.84, 0.55)	0.08 (-1.21, 1.37)		.	.	.
Casein+MET	0.16 (-1.06, 1.37)	0.29 (-1.31, 1.90)	0.22 (-1.02, 1.46)	-0.20 (-1.47, 1.06)	-1.19 (-2.87, 0.48)	0.02 (-1.24, 1.29)	-0.05 (-1.67, 1.57)		.	.
SP+MET	0.19 (-1.30, 1.68)	0.33 (-1.49, 2.15)	0.25 (-1.15, 1.65)	-0.17 (-1.70, 1.36)	-1.16 (-3.05, 0.73)	0.06 (-1.44, 1.56)	-0.02 (-1.85, 1.81)	0.04 (-1.76, 1.83)		.
Collagen+RET	-0.00 (-1.22, 1.22)	0.14 (-1.53, 1.80)	0.06 (-1.35, 1.47)	-0.36 (-1.71, 0.98)	-1.35 (-3.09, 0.38)	-0.13 (-1.56, 1.29)	-0.21 (-1.94, 1.52)	-0.16 (-1.88, 1.57)	-0.19 (-2.12, 1.73)	
DP+RET	0.21 (-0.40, 0.83)	0.35 (-0.87, 1.58)	0.27 (-0.46, 1.01)	-0.15 (-0.84, 0.54)	-1.14 (-2.46, 0.19)	0.08 (-0.68, 0.84)	0.00 (-1.23, 1.24)	0.06 (-1.17, 1.29)	0.02 (-1.48, 1.52)	0.21 (-1.15, 1.58)
DP+AET	**0.85 (0.25, 1.45)**	0.99 (-0.09, 2.07)	**0.91 (0.07, 1.75)**	0.49 (-0.28, 1.26)	-0.50 (-1.58, 0.57)	0.72 (-0.15, 1.58)	0.64 (-0.67, 1.95)	0.69 (-0.59, 1.98)	0.66 (-0.90, 2.21)	0.85 (-0.51, 2.21)
DP+MET	0.36 (-0.18, 0.91)	0.50 (-0.68, 1.68)	0.42 (-0.17, 1.02)	0.00 (-0.65, 0.66)	-0.99 (-2.27, 0.29)	0.23 (-0.42, 0.88)	0.15 (-1.05, 1.36)	0.21 (-0.88, 1.29)	0.17 (-1.26, 1.60)	0.36 (-0.97, 1.70)
Rice+MET	0.28 (-1.06, 1.62)	0.42 (-1.28, 2.12)	0.34 (-0.79, 1.48)	-0.08 (-1.46, 1.30)	-1.07 (-2.85, 0.71)	0.15 (-1.22, 1.52)	0.07 (-1.64, 1.78)	0.12 (-1.56, 1.81)	0.09 (-1.71, 1.89)	0.28 (-1.53, 2.09)
WP	**0.60 (0.00, 1.20)**	0.74 (-0.35, 1.82)	0.66 (-0.15, 1.47)	0.24 (-0.49, 0.97)	-0.75 (-2.04, 0.53)	0.47 (-0.37, 1.30)	0.39 (-0.89, 1.68)	0.44 (-0.83, 1.72)	0.41 (-1.13, 1.95)	0.60 (-0.76, 1.96)
MP	0.47 (-1.04, 1.99)	0.61 (-1.25, 2.47)	0.53 (-1.08, 2.15)	0.11 (-1.29, 1.52)	-0.88 (-2.81, 1.05)	0.34 (-1.28, 1.96)	0.26 (-1.63, 2.16)	0.32 (-1.58, 2.21)	0.28 (-1.80, 2.36)	0.47 (-1.47, 2.42)
DP	**1.67 (0.99, 2.35)**	**1.81 (0.58, 3.03)**	**1.73 (0.90, 2.56)**	**1.31 (0.51, 2.11)**	0.32 (-1.01, 1.65)	**1.54 (0.68, 2.40)**	**1.46 (0.15, 2.77)**	**1.51 (0.25, 2.78)**	1.48 (-0.07, 3.03)	**1.67 (0.27, 3.07)**
RET	0.37 (-0.02, 0.76)	0.51 (-0.66, 1.67)	0.43 (-0.30, 1.16)	0.01 (-0.45, 0.46)	-0.98 (-2.25, 0.29)	0.24 (-0.51, 0.99)	0.16 (-1.07, 1.39)	0.21 (-1.01, 1.44)	0.18 (-1.32, 1.68)	0.37 (-0.91, 1.66)
MET	0.32 (-0.39, 1.02)	0.45 (-0.81, 1.72)	0.37 (-0.12, 0.87)	-0.05 (-0.83, 0.74)	-1.04 (-2.40, 0.33)	0.18 (-0.55, 0.91)	0.11 (-1.17, 1.38)	0.16 (-1.07, 1.39)	0.12 (-1.19, 1.43)	0.32 (-1.10, 1.73)
RC	**0.73 (0.30, 1.17)**	0.87 (-0.27, 2.01)	**0.79 (0.22, 1.36)**	0.37 (-0.18, 0.92)	-0.62 (-1.87, 0.63)	**0.60 (0.00, 1.20)**	0.52 (-0.62, 1.66)	0.57 (-0.57, 1.72)	0.54 (-0.89, 1.97)	0.73 (-0.57, 2.03)
	Relative effects of NMA (column compared with row)									

To be continued.

Table S10. Continued.

	Direct evidence of pairwise meta-analyses (row compared with column)									
	DP+RET	DP+AET	DP+MET	Rice+MET	WP	MP	DP	RET	MET	RC
WP+RET	.	**1.48 (0.67, 2.28)**	.	.	0.06 (-0.86, 0.97)	.	**2.52 (1.21, 3.83)**	0.13 (-0.30, 0.56)	.	**0.71 (0.13, 1.29)**
WP+AET	.	0.76 (-0.42, 1.93)	.	.	0.97 (-0.21, 2.15)	.	.	.	.	.
WP+MET	.	.	-0.00 (-1.34, 1.34)	0.34 (-0.79, 1.48)	.	.	.	.	0.33 (-0.25, 0.91)	**1.04 (0.21, 1.87)**
MP+RET	.	.	.	.	.	0.11 (-1.29, 1.52)	.	0.16 (-0.36, 0.67)	.	-0.02 (-0.86, 0.82)
MP+AET	.	-0.50 (-1.58, 0.57)	.	.	.	.	.	.	.	.
MP+MET	.	.	0.34 (-0.73, 1.42)	.	.	.	.	.	0.05 (-1.02, 1.11)	0.61 (-0.14, 1.35)
Casein+RET	.	.	.	.	.	.	.	.	.	0.52 (-0.62, 1.66)
Casein+MET	.	.	0.21 (-0.88, 1.29)	.	.	.	.	.	.	.
SP+MET	.	.	.	.	.	.	.	.	0.12 (-1.19, 1.43)	.
Collagen+RET	.	.	.	.	.	.	.	.	.	.
DP+RET		.	0.07 (-0.98, 1.13)	.	.	.	.	0.52 (-0.58, 1.62)	.	0.43 (-0.11, 0.98)
DP+AET	0.64 (-0.14, 1.41)		0.17 (-1.08, 1.41)	.	0.22 (-0.95, 1.39)	.	1.16 (-0.11, 2.43)	.	.	0.83 (-0.43, 2.08)
DP+MET	0.15 (-0.43, 0.73)	-0.49 (-1.18, 0.21)		.	.	.	**1.20 (0.37, 2.03)**	.	-0.12 (-0.89, 0.64)	0.39 (-0.05, 0.83)
Rice+MET	0.07 (-1.29, 1.42)	-0.57 (-1.98, 0.84)	-0.08 (-1.37, 1.20)		.	.	.	.	.	.
WP	0.39 (-0.35, 1.12)	-0.25 (-0.95, 0.45)	0.24 (-0.43, 0.90)	0.32 (-1.08, 1.71)		.	0.42 (-0.92, 1.77)	0.20 (-0.71, 1.12)	.	0.28 (-0.48, 1.04)
MP	0.26 (-1.31, 1.83)	-0.38 (-1.98, 1.23)	0.11 (-1.44, 1.66)	0.19 (-1.78, 2.16)	-0.13 (-1.71, 1.46)		.	.	.	.
DP	**1.46 (0.67, 2.24)**	**0.82 (0.03, 1.60)**	**1.31 (0.65, 1.96)**	1.39 (-0.02, 2.79)	**1.07 (0.33, 1.81)**	1.20 (-0.42, 2.82)		.	.	-0.33 (-1.59, 0.93)
RET	0.16 (-0.45, 0.76)	-0.48 (-1.16, 0.19)	0.01 (-0.57, 0.58)	0.09 (-1.26, 1.44)	-0.23 (-0.86, 0.40)	-0.10 (-1.58, 1.38)	**-1.30 (-2.02, -0.58)**		.	0.17 (-0.75, 1.09)
MET	0.10 (-0.63, 0.83)	-0.54 (-1.37, 0.30)	-0.05 (-0.63, 0.53)	0.03 (-1.21, 1.27)	-0.29 (-1.09, 0.52)	-0.16 (-1.77, 1.45)	**-1.35 (-2.18, -0.53)**	-0.06 (-0.78, 0.67)		-0.08 (-1.14, 0.99)
RC	**0.52 (0.03, 1.00)**	-0.12 (-0.76, 0.52)	0.37 (-0.01, 0.74)	0.45 (-0.82, 1.72)	0.13 (-0.46, 0.72)	0.26 (-1.25, 1.77)	**-0.94 (-1.59, -0.29)**	0.36 (-0.10, 0.82)	0.42 (-0.15, 0.99)	
	Relative effects of NMA (column compared with row)									

Pairwise (upper right portion) and network (lower left portion) meta-analysis results are presented for mean change (from baseline) in global function. Effect estimation is presented in standardized mean difference with 95% confidence interval. Significant results ($p < 0.05$) are marked in bold.

AET, aerobic exercise training; DP, dietary protein; Ins-PS, insect protein supplement; MET, multicomponent exercise training; MP, milk protein; RET, resistance exercise training; SP, soy protein; WP, whey protein; RC, regular care; SPPB, Short Physical Performance Battery.

Supplementary table S11. Summary for meta-regression results.

Moderator	Muscle mass						Handgrip strength						Leg muscle strength					
	N	B	SE	Median	95% CrI		N	B	SE	Median	95% CrI		N	B	SE	Median	95% CrI	
**Participant factor**																		
Age range[a]	**218**	**–0.299**	**0.0027**	**–0.298**	**–0.591,**	**–0.006**	133	–0.042	0.0031	–0.046	–0.279,	0.201	**129**	**–0.421**	**0.0039**	**–0.426**	**–0.759,**	**–0.064**
BMI	206	–0.113	0.003	–0.113	–0.397,	0.179	**116**	**–0.260**	**0.0007**	**–0.261**	**–0.517,**	**–0.001**	124	0.529	0.0019	0.609	–0.283,	0.397
Sex[b]	**218**	**–0.327**	**0.0032**	**–0.327**	**–0.601,**	**–0.048**	132	–0.222	0.0031	–0.225	–0.492,	0.048	129	–0.103	0.0038	–0.102	–0.427,	0.219
Area of population[c]	**218**	**–0.327**	**0.0042**	**–0.325**	**–0.020,**	**–0.639**	**133**	**–0.419**	**0.003**	**–0.418**	**–0.697,**	**–0.143**	**129**	**–0.421**	**0.0018**	**–0.419**	**–0.751,**	**–0.099**
Health status[d]	**218**	**0.283**	**0.0025**	**0.281**	**0.011,**	**0.558**	133	0.009	0.0335	0.01	–0.279,	0.295	129	–0.270	0.0018	–0.271	–0.595,	0.057
**Study design factor**																		
ROB[e]	218	0.246	0.0037	0.246	–0.043,	0.539	133	0.209	0.0053	0.227	–1.280,	1.666	129	0.689	0.0027	0.684	–0.062,	1.432
Follow-up duration	218	–0.107	0.0038	–0.106	–0.309,	0.092	**133**	**0.224**	**0.0008**	**0.223**	**0.007,**	**0.449**	129	0.043	0.0013	0.044	–0.183,	0.263
**Intervention factor**																		
PS type[f]	**218**	**0.265**	**0.0013**	**0.264**	**0.034**	**0.499**	133	0.025	0.0357	0.217	–3.844,	0.468	129	–0.186	0.0021	–0.189	–0.450,	0.077
PS dose[g]	**218**	**0.283**	**0.0034**	**0.284**	**0.001**	**0.574**	**133**	**0.363**	**0.0035**	**0.364**	**0.082**	**0.641**	129	0.134	0.0012	0.133	–0.181,	0.454
Training type[h]	218	0.044	0.003	0.046	–0.355,	0.433	133	0.221	0.0069	0.223	–0.186,	0.568	129	0.103	0.0045	–0.099	–0.504,	0.277
Treatment duration	218	–0.084	0.0037	–0.085	–0.282,	0.118	**133**	**0.236**	**0.0035**	**0.235**	**0.018,**	**0.453**	129	0.044	0.0012	0.044	–0.174,	0.267

To be continued .

Supplementary table S11. Continued.

Moderator	Walking speed					Chair rise					Timed up and go					SPPB				
	N	B	SE	Median	95% CrI	N	B	SE	Median	95% CrI	N	B	SE	Median	95% CrI	N	B	SE	Median	95% CrI
**Participant factor**																				
Age range[a]	**126**	**−0.281**	**0.0027**	**−0.298**	**−0.520, −0.042**	68	−0.379	0.0058	−0.376	−0.909, 0.087	47	0.221	0.0063	0.227	−0.358, 0.782	49	0.151	0.0069	0.149	−0.466, 0.755
BMI	117	−0.213	0.002	−0.214	−0.496, 0.065	67	0.222	0.0037	0.219	−0.806, 1.247	44	−0.293	0.0015	−0.294	−0.719, 0.14	47	−0.378	0.0068	−0.374	−0.923, 0.156
Sex[b]	126	0.005	0.0036	0.006	−0.316, 0.311	**66**	**0.614**	**0.0067**	**0.633**	**0.233, 1.182**	45	0.411	0.0051	0.415	−0.036, 0.833	49	−0.040	0.0063	−0.045	−0.589, 0.493
Area of population[c]	126	−0.081	0.0001	−0.083	−0.355, 0.201	68	−0.211	0.0034	−0.215	−0.795, 0.382	47	−0.037	0.0019	−0.039	−0.552, 0.493	49	−0.187	0.0032	−0.186	−0.764, 0.381
Health status[d]	126	−0.127	0.001	−0.128	−0.397, 0.144	68	−0.343	0.0028	−0.345	−0.830, 0.156	47	0.097	0.0015	0.095	−0.313, 0.509	49	0.08	0.0017	0.078	−0.394, 0.559
**Study design factor**																				
ROB[e]	126	−0.058	0.001	−0.057	−0.333, 0.214	68	0.225	0.0031	0.224	−0.330, 0.777	47	−0.022	0.0014	−0.022	−0.414, 0.373	49	−0.156	0.0036	−0.159	−0.792, 0.473
Follow-up duration	126	−0.068	0.0008	−0.069	−0.284, 0.151	68	−0.015	0.0024	−0.017	−0.435, 0.401	47	−0.134	0.0013	−0.135	−0.489, 0.224	49	−0.044	0.0022	−0.045	−0.424, 0.348
**Intervention factor**																				
PS type[f]	126	−0.048	0.003	−0.046	−0.306, 0.2	68	0.057	0.0059	0.053	−0.464, 0.583	47	0.228	0.0047	0.224	−0.186, 0.635	49	0.007	0.0074	0.003	−0.612, 0.674
PS dose[g]	126	0.174	0.0037	0.169	−0.111, 0.468	68	0.177	0.0061	0.177	−0.296, 0.679	47	0.285	0.0034	0.284	−0.367, 0.946	49	0.266	0.0032	0.264	−0.364, 0.899
Training type[h]	126	0.144	0.004	0.146	−0.213, 0.493	68	0.136	0.0079	0.147	−0.610, 0.826	47	0.231	0.0069	0.232	−0.361, 0.85	49	0.409	0.0096	0.394	−0.381, 1.319
Treatment duration	126	−0.079	0.0008	−0.079	−0.293, 0.143	68	0.012	0.0024	0.012	−0.422, 0.448	47	−0.203	0.0013	−0.202	−0.554, 0.146	49	−0.056	0.0022	−0.054	−0.443, 0.324

Data represents the change in effects associated with the moderator indicated. B, beta coefficient; SE, standard error; 95% CrI, 95% credibility interval.

[a]Code for regression model: < 65 years = 1; 65–74.9 years = 2; 75–84.9 years = 3; ⩾ 85 years = 4.

[b]Code for regression model:; ≥ 50 % male participants in sample = 2; ≥ 50 % female participants in sample = 3; women only = 4.

[c]Code for regression model: America = 1; Asian = 2; Europe = 3; Oceania = 4.

[d]Code for regression model: healthy = 1; subhealthy = 2.

[e]Code for regression model: low risk of bias = 1; medium risk of bias = 2; high risk of bias = 3.

[f]Code for regression model: plant protein = 1; mixed protein = 2; animal protein = 3.

[g]Code for regression model: low dose (≤ 20 g/session or 1.0 g/kg body weight/day) = 1; higher dose (> 20 g/session or 1.0 g/kg body weight/day) = 2.

[h]Code for regression model: resistance training = 1; multicomponent exercise regimen = 2; aerobic training = 3.

95% CrI, credible interval; BMI, body mass index; ROB, risk of bias; SE, standard error.

Supplementary table S12. Assessment for treatment safety.

Study (year)	Study arm	Group sample (n)	Withdraw, attrition rate, or drop out (number of patients)				Side effects and complications (number of patients)				Serious adverse event (number of patients)		
			Related to PS	Related to ExT	Unrelated to intervention	Total sum	Related to PS	Related to ExT	Unrelated to intervention	Total sum	Related to intervention	Unrelated to intervention	Total sum
Aas 2020	Gr 1: MP + RET	11	0	0	0	0	0	0	0	0	0	0	0
	Gr 2: RC	11			0	0			0	0	0	0	0
Amasene 2019; 2021	Gr 1: WP + RET	21	5	0	1	6	1	0	0	1	NR	NR	
	Gr 2: PLA + RET	20	3	0	4	7	0	0	0	0	NR	NR	
Arciero 2014	Gr 1: WP + MET	28	2	6	3	11	NR	NR	NR		NR	NR	
	Gr 2: WP + RET	27	1	4	0	5	NR	NR	NR		NR	NR	
	Gr 3: WP	24	6		0	6	NR		NR		NR	NR	
Arentson-Lantz 2019	Gr 1: WP + MET	10	0	0	0	0	NR	NR	NR		NR	NR	
	Gr 2: DP + MET	11	0	0	1	1	NR	NR	NR		NR	NR	
Arentson-Lantz 2020	Gr 1: WP + MET	10	0	0	0	0	NR	NR	NR		NR	NR	
	Gr 2: PLA + MET	10	0	0	1	1	NR	NR	NR		NR	NR	
Arnarson 2013	Gr 1: WP + RET	83	0	0	8	8	NR	NR	NR		NR	NR	
	Gr 2: PLA + RET	78	0	0	12	12	NR	NR	NR		NR	NR	
Assantachai 2020	Gr 1: Meat + MET	10	1	0	0	1	1	0	0	1	0	1	1
	Gr 2: PLA + MET	8	0	0	0	0	0	0	0	0	0	0	0
	Gr 3: Meat	10	0		1	1	0		0	0	0	0	0
	Gr 4: PLA	7	0		0	0	0		0	0	0	0	0
Atherton 2020	Gr 1: WP + RET	10	0	0	0	0	0	0	0	0	0	0	0
	Gr 2: PLA + RET	10	0	0	1	1	0	0	0	0	0	0	0
Bagheri 2022	Gr 1: WP + RET	14	0	0	0	0	0	0	0	0	0	0	0
	Gr 2: PLA + RET	14	0	0	0	0	0	0	0	0	0	0	0
Bauer 2024	Gr 1: WP + RET	63	0	0	0	0	NR	NR	NR		NR	NR	
	Gr 2: DP + RET	55	0	0	0	0	NR	NR	NR		NR	NR	
	Gr 3: WP + MET	114	0	0	0	0	NR	NR	NR		NR	NR	
Beck 2008; 2016	Gr 1: MP + MET	62	0	0	0	0	0	0	0	0	0	0	0
	Gr 2: RC	59			0	0			0	0	0	0	0
Bell 2017	Gr 1: WP + MET	25	2	0	5	7	NR	NR	NR		NR	NR	
	Gr 2: PLA + MET	24	1	0	3	4	NR	NR	NR		NR	NR	
Bemben 2010; Eliot 2008	Gr 1: WP + Cre + RET	11	0	0	0	0	0	0	0	0	0	0	0
	Gr 2: WP + RET	11	0	0	0	0	0	0	0	0	0	0	0
	Gr 3: Cre + RET	10	0	0	0	0	0	0	0	0	0	0	0
	Gr 4: PLA + RET	10	0	0	0	0	0	0	0	0	0	0	0
Bernabei 2022	Gr 1: DP + MET	759	0	0	227	227	40	67	27	134	0	282	282
	Gr 2: RC	759			222	222			112	112	0	264	264
Blanc-Bisson 2008	Gr 1: MP + RET	38	0	0	13	13	0	0	9	9	0	3	3
	Gr 2: MP	38	0	0	12	12	0	0	5	5	0	2	2

To be continued.

Supplementary table S12. Continued.

Study (year)	Study arm	Group sample (n)	Withdraw, attrition rate, or drop out (number of patients)				Side effects and complications (number of patients)				Serious adverse event (number of patients)		
			Related to PS	Related to ExT	Unrelated to intervention	Total sum	Related to PS	Related to ExT	Unrelated to intervention	Total sum	Related to intervention	Unrelated to intervention	Total sum
Biesek 2021	Gr 1: WP + MET	18	0	0	2	2	NR	NR	NR		NR	NR	
	Gr 2: PLA + MET	18	0	0	3	3	NR	NR	NR		NR	NR	
	Gr 3: MET	18		0	3	3		NR	NR		NR	NR	
	Gr 4: WP	18	0		0	0	NR		NR		NR	NR	
	Gr 5: RC	18			3	3			NR		NR	NR	
Bijeh 2022	Gr 1: SP + RET	15	0	0	0	0	0	0	0	0	0	0	0
	Gr 2: RET	15		0	0	0		0	0	0	0	0	0
	Gr 3: SP	15	0		0	0	0		0	0	0	0	0
	Gr 4: RC	15			0	0			0	0	0	0	0
Bjorkman 2011	Gr 1: WP + RET	23	0	0	1	1	3	0	0	3	0	0	0
	Gr 2: Casein + RET	24	0	0	0	0	0	0	0	0	0	0	0
Bjorkman 2012	Gr 1: WP + AET	49	0	0	3	3	NR	NR	NR		0	3	3
	Gr 2: PLA + AET	57	0	0	6	6	NR	NR	NR		0	5	5
Bjorkman 2020	Gr 1: MP + MET	73	0	0	27	27	40	0	0	40	0	16	16
	Gr 2: DP + MET	72	0	0	27	27	7	0	0	7	0	13	13
	Gr 3: PLA + MET	73	0	0	27	27	40	0	0	40	0	17	17
Bonnefoy 2003	Gr 1: SP + MET	15	0	0	4	4	0	0	1	1	0	0	0
	Gr 2: PLA + MET	13	0	0	3	3	0	0	1	1	0	0	0
	Gr 3: SP	15	0		4	4	0		1	1	0	0	0
	Gr 4: PLA	14	0		4	4	0		2	2	0	1	1
Bonnefoy 2012	Gr 1: SP + MET	53	0	3	1	4	0	0	0	0	0	4	4
	Gr 2: RC	49			0	0			0	0	0	0	0
Botella-Carretero 2008	Gr 1: SP + MET	30	1	0	1	2	1	0	0	1	0	1	1
	Gr 2: MP + MET	30	0	0	0	0	0	0	0	0	0	0	0
	Gr 3: MET	30		0	3	3		0	0	0	0	1	1
Boutry-Regard 2020	Gr 1: ω3FA + WP + RET	13	0	0	3	3	3	0	0	3	0	0	0
	Gr 2: WP + RET	15	0	0	0	0	4	0	0	4	0	0	0
	Gr 3: PLA + RET	13	0	0	1	1	2	0	0	2	0	0	0
Buhl 2016	Gr 1: MP + RET	14	0	0	1	1	0	3	0	3	0	0	0
	Gr 2: RC	15			4	4			0	0	0	0	0
Bunout 2001; 2004	Gr 1: SP + RET	42	0	0	11	11	0	0	0	0	0	3	3
	Gr 2: RET	32		7	9	16		0	0	0	0	0	0
	Gr 3: SP	42	0		14	14	0		0	0	0	2	2
	Gr 4: RC	40			15	15			0	0	0	1	1

To be continued.

Supplementary table S12. Continued.

Study (year)	Study arm	Group sample (n)	Withdraw, attrition rate, or drop out (number of patients)				Side effects and complications (number of patients)				Serious adverse event (number of patients)		
			Related to PS	Related to ExT	Unrelated to intervention	Total sum	Related to PS	Related to ExT	Unrelated to intervention	Total sum	Related to intervention	Unrelated to intervention	Total sum
Candow 2006	Gr 1: WP + RET	25	0	0	6	6	NR	NR	NR		NR	NR	
	Gr 2: PLA + RET	13	0	0	3	3	NR	NR	NR		NR	NR	
Candow 2008	Gr 1: WP + Cre + RET	12	0	0	2	2	1	0	0	1	0	0	0
	Gr 2: Cre + RET	14	0	0	1	1	1	1	0	2	0	0	0
	Gr 3: PLA + RET	14	0	0	2	2	2	2	0	4	0	0	0
Cao 2007	Gr 1: DP + MET	41	0	0	1	1	NR	NR	NR		NR	NR	
	Gr 2: MET	49		0	1	1	NR	NR	NR		NR	NR	
	Gr 3: RC	47			9	9	NR	NR	NR		NR	NR	
Carlsson 2011; Rosendahl 2006	Gr 1: MP + MET	42	0	0	5	5	NR	NR	NR		0	0	0
	Gr 2: PLA + MET	41	0	0	15	15	NR	NR	NR		0	3	3
	Gr 3: MP	47	0		17	17	NR		NR		0	2	2
	Gr 4: PLA	47	0		8	8	NR		NR		0	1	1
Carroll 2024	Gr 1: Beef + RET	16	2	2	3	7	NR	NR	NR		NR	NR	
	Gr 2: DP + RET	17	0	0	3	3	NR	NR	NR		NR	NR	
	Gr 3: RET	16		1	4	5		NR	NR		NR	NR	
Centner 2019	Gr 1: Collagen + RET	13	0	0	2	2	NR	NR	NR		NR	NR	
	Gr 2: PLA + RET	13	0	0	2	2	NR	NR	NR		NR	NR	
	Gr 3: RC	13			5	5			NR		NR	NR	
Chale 2013	Gr 1: WP + RET	42	0	0	3	3	4	1	9	14	0	6	6
	Gr 2: PLA + RET	38	0	0	2	2	2	3	15	20	0	3	3
Chang 2019	Gr 1: Casein + RET	28	0	0	0	0	NR	NR	NR		NR	NR	
	Gr 2: RC	28			0	0			NR		NR	NR	
Chatterjee 2018	Gr 1: DP + AET	22	0	0	0	0	0	0	0	0	0	0	0
	Gr 2: WP + AET	22	0	0	0	0	1	0	0	1	0	0	0
	Gr 3: WP	22	0		0	0	2		0	2	0	0	0
Chen 2017	Gr 1: WP + RET	20	0	0	0	0	NR	NR	NR		NR	NR	
	Gr 2: DP	20	0		0	0	NR		NR		NR	NR	
Chen 2021; Hsu 2021	Gr 1: WP + AET	23	0	0	2	0	NR	NR	NR		NR	NR	
	Gr 2: AET	23		0	2	0		NR	NR		NR	NR	
	Gr 3: RC	23			0	0			NR		NR	NR	
Chen 2024	Gr 1: Casein + MET	41	0	0	6	6	0	0	3	3	0	1	1
	Gr 2: DP + MET	58	0	0	10	10	0	0	7	7	0	3	3
Chin A Paw 2001; de Jong 2000	Gr 1: Casein + MET	60	0	1	17	18	NR	NR	NR		NR	NR	
	Gr 2: PLA + MET	55	0	1	15	16	NR	NR	NR		NR	NR	
	Gr 3: Casein	58	0		19	19	NR		NR		NR	NR	
	Gr 4: PLA	44	0		7	7	NR		NR		NR	NR	

To be continued.

Supplementary table S12. Continued.

Study (year)	Study arm	Group sample (n)	Withdraw, attrition rate, or drop out (number of patients)				Side effects and complications (number of patients)				Serious adverse event (number of patients)		
			Related to PS	Related to ExT	Unrelated to intervention	Total sum	Related to PS	Related to ExT	Unrelated to intervention	Total sum	Related to intervention	Unrelated to intervention	Total sum
Colonetti 2023	Gr 1: WP + RET	10	0	2	0	2	0	0	0	0	0	0	0
	Gr 2: PLA + RET	10	0	0	0	0	0	0	0	0	0	0	0
	Gr 3: RC	10			2	2			0	0	0	0	0
Corcoran 2017	Gr 1: MP + MET	67	0	0	20	20	3	1	19		0	11	11
	Gr 2: RC	54			8	8			7		0	5	5
Dalla Via 2021	Gr 1: WP + MET	34	0	0	3	3	3	14	0	17	0	0	0
	Gr 2: RC	36			7	7			0	0	0	1	1
Daly 2014	Gr 1: Meat + RET	53	0	0	5	5	0	2	0	2	0	0	0
	Gr 2: PLA (rice) + RET	47	0	0	4	4	0	2	0	2	0	0	0
Daly 2020	Gr 1: MP + MET	123	0	0	15	15	22	28	0	50	0	0	0
	Gr 2: PLA (rice) + MET	121	0	0	13	13	8	21	0	29	0	0	0
de Azevedo Bach 2022	Gr 1: WP + RET	18	0	3	0	3	0	0	0	0	0	0	0
	Gr 2: PLA + RET	18	1	1	0	2	0	0	0	0	0	0	0
de Carvalho Bastone 2020	Gr 1: WP + RET	20	0	0	4	4	NR	NR	NR		NR	NR	
	Gr 2: RET	20		0	3	3		NR	NR		NR	NR	
	Gr 3: WP	20	0		2	2	NR		NR		NR	NR	
	Gr 4: RC	20			2	2			NR		NR	NR	
Deer 2019	Gr 1: WP + RET	20	0	0	4	4	NR	NR	NR		NR	NR	
	Gr 2: PLA + RET	21	0	0	4	4	NR	NR	NR		NR	NR	
	Gr 3: WP	20	0		5	5	NR		NR		NR	NR	
	Gr 4: PLA	20	0		7	7	NR		NR		NR	NR	
Deibert 2011	Gr 1: SP + RET	14	0	0	1	1	NR	NR	NR		NR	NR	
	Gr 2: RET	14		0	1	1		NR	NR		NR	NR	
	Gr 3: RC	12			3	3			NR		NR	NR	
Dirks 2017; Tieland 2012	Gr 1: MP + RET	31	0	0	5	5	NR	NR	NR		NR	NR	
	Gr 2: PLA (CHO) + RET	31	0	0	6	6	NR	NR	NR		NR	NR	
Duff 2014	Gr 1: MP + RET	19	0	0	0	0	2	0	0	2	0	0	0
	Gr 2: WP + RET	21	0	0	1	1	3	0	0	3	0	0	0
Dulac 2021	Gr 1: WP + RET	25	0	0	4	4	0	0	0	0	0	0	0
	Gr 2: Casein + RET	25	0	0	5	5	0	0	0	0	0	0	0
	Gr 3: PLA + RET	25	0	0	6	6	0	0	0	0	0	0	0

To be continued.

Supplementary table S12. Continued.

Study (year)	Study arm	Group sample (n)	Withdraw, attrition rate, or drop out (number of patients)				Side effects and complications (number of patients)				Serious adverse event (number of patients)		
			Related to PS	Related to ExT	Unrelated to intervention	Total sum	Related to PS	Related to ExT	Unrelated to intervention	Total sum	Related to intervention	Unrelated to intervention	Total sum
Edholm 2017; Strandberg 2015	Gr 1: DP + RET	21	0	0	1	1	NR	NR	NR		NR	NR	
	Gr 2: RET	21	0	0	4	4		NR	NR		NR	NR	
	Gr 3: RC	21	0	0	3	3			NR		NR	NR	
Englund 2017; Fielding 2017	Gr 1: WP + MET	74	0	0	4	4	18	26	5	49	0	5	5
	Gr 2: PLA + MET	75	0	0	8	8	9	27	5	41	0	7	7
Evans 2007	Gr 1: MP + AET	16	0	0	6	6	NR	NR	NR		NR	NR	
	Gr 2: SP + AET	17	0	0	6	6	NR	NR	NR		NR	NR	
	Gr 3: MP	15	0		3	3	NR		NR		NR	NR	
	Gr 4: SP	13	0		3	3	NR		NR		NR	NR	
Fernandes 2018; Sugihara Junior 2018	Gr 1: WP + RET	15	0	0	0	0	NR	NR	NR		NR	NR	
	Gr 2: PLA (CHO) + RET	16	0	0	0	0	NR	NR	NR		NR	NR	
Fiatarone 1994	Gr 1: SP + RET	25	0	0	1	1	1	1	0	2	0	0	0
	Gr 2: PLA + RET	25	0	1	1	2	0	2	1	3	0	0	0
	Gr 3: SP	24	0		2	2	1		0	1	0	0	0
	Gr 4: PLA	26	0		1	1	0		0	0	0	0	0
Filho 2022	Gr 1: WP + MET	18	0	0	2	2	NR	NR	NR		NR	NR	
	Gr 2: PLA + MET	18	0	2	1	3	NR	NR	NR		NR	NR	
	Gr 3: MET	18		0	3	3		NR	NR		NR	NR	
	Gr 4: WP	18	0		0	0	NR		NR		NR	NR	
	Gr 5: RC	18			2	2			NR		NR	NR	
Flodin 2015	Gr 1: MP + MET	26	0	0	8	8	NR	NR	NR		NR	NR	
	Gr 2: Bisphosphonates + MET	28	0	0	3	3	NR	NR	NR		NR	NR	
	Gr 3: Vitamin-D + MET	25	0	0	1	1	NR	NR	NR		NR	NR	
Formica 2020	Gr 1: Meat + RET	77	0	0	4	4	0	6	0	6	0	0	0
	Gr 2: RET	77		0	5	5		7	0	7	0	0	0
Francis 2017	Gr 1: MP + RET	29	7	0	13	20	NR	NR	NR		NR	NR	
	Gr 2: MP	28	9		13	22	NR		NR		NR	NR	
Franzke 2015a; 2015b	Gr 1: WP + RET	29	0	0	7	7	NR	NR	NR		NR	NR	
	Gr 2: RET	35		0	9	9		NR	NR		NR	NR	
	Gr 3: RC	33			11	11			NR		NR	NR	
Fujie 2025	Gr 1: Meat + RET	24	0	2	4	6	NR	NR	NR		NR	NR	
	Gr 2: RET	23		2	1	3		NR	NR		NR	NR	
	Gr 3: Meat	23	1		0	1	NR		NR		NR	NR	
	Gr 4: RC	23			2	2			NR		NR	NR	

To be continued.

Supplementary table S12. Continued.

Study (year)	Study arm	Group sample (n)	Withdraw, attrition rate, or drop out (number of patients)				Side effects and complications (number of patients)				Serious adverse event (number of patients)		
			Related to PS	Related to ExT	Unrelated to intervention	Total sum	Related to PS	Related to ExT	Unrelated to intervention	Total sum	Related to intervention	Unrelated to intervention	Total sum
Furtado 2024	Gr 1: WP + RET	19	0	0	1	1	NR	NR	NR		NR	NR	
	Gr 2: PLA (MP) + RET	20	0	0	3	3	NR	NR	NR		NR	NR	
Gade 2019	Gr 1: WP + RET	83	0	2	3	5	0	0	0	0	0	0	0
	Gr 2: PLA (MP) + RET	82	0	1	4	5	0	0	0	0	0	0	0
Gaffney 2018	Gr 1: WP + MET	12	0	0	0	0	NR	NR	NR		NR	NR	
	Gr 2: PLA (Oat) + MET	12	0	0	0	0	NR	NR	NR		NR	NR	
Galbreath 2018	Gr 1: DP + MET	24	1	0	6	7	NR	NR	NR		NR	NR	
	Gr 2: REHC + MET	24	0	0	6	6	NR	NR	NR		NR	NR	
	Gr 3: MET	24		0	5	5		NR	NR		NR	NR	
Gao 2019	Gr 1: DP + RET	38	0	0	0	0	NR	NR	NR		NR	NR	
	Gr 2: RC	38			0	0			NR		NR	NR	
George 2017	Gr 1: WP + MET	6	0	0	3	3	NR	NR	NR		NR	NR	
	Gr 2: RC	5			2	2			NR		NR	NR	
Grabovac 2018; Haider 2017; Kapan 2017	Gr 1: DP + RET	39	0	0	4	4	0	0	2	2	0	2	2
	Gr 2: RC	41			12	12			0	0	0	1	1
Granic 2020	Gr 1: Whole-MP + RET	10	0	0	1	1	0	0	0	0	0	0	0
	Gr 2: Skimmed-MP + RET	10	0	0	0	0	0	0	0	0	0	0	0
	Gr 3: PLA (CHO) + RET	10	0	0	0	0	0	0	0	0	0	0	0
Griffen 2022	Gr 1: WP + RET	9	0	0	0	0	0	0	0	0	0	0	0
	Gr 2: PLA + RET	10	0	0	1	1	0	1	0	1	0	0	0
	Gr 3: WP	10	0		1	1	0		0	0	0	0	0
	Gr 4: PLA	10	0		1	1	0		0	0	0	0	0
Gronstedt 2020	Gr 1: MP + RET	60	0	0	8	8	3	0	0	3	0	11	11
	Gr 2: RET	60		0	10	10		1	0	1	0	13	13
Gryson 2014	Gr 1: WP + MET	8	0	0	0	0	NR	NR	NR		NR	NR	
	Gr 2: Casein + MET	9	0	0	0	0	NR	NR	NR		NR	NR	
	Gr 3: PLA + MET	9	0	0	0	0	NR	NR	NR		NR	NR	
	Gr 4: WP	10	0		0	0	NR		NR		NR	NR	
	Gr 5: PLA	9	0		0	0	NR		NR		NR	NR	
Gusdon 2024	Gr 1: WP + RET	12	0	0	0	0	NR	NR	NR		NR	NR	
	Gr 2: DP	12	0		0	0	NR		NR		NR	NR	
Haß 2022	Gr 1: WP + Omega-3 + RET	28	2	0	5	7	NR	NR	NR		NR	NR	
	Gr 2: WP + RET	27	2	0	5	7	NR	NR	NR		NR	NR	
	Gr 3: RET	22		0	2	2		NR	NR		NR	NR	
Hamarsland 2019	Gr 1: MP + RET	19	0	0	4	4	NR	NR	NR		NR	NR	
	Gr 2: WP + RET	19	0	0	4	4	NR	NR	NR		NR	NR	

To be continued.

Supplementary table S12. Continued.

Study (year)	Study arm	Group sample (n)	Withdraw, attrition rate, or drop out (number of patients)				Side effects and complications (number of patients)				Serious adverse event (number of patients)		
			Related to PS	Related to ExT	Unrelated to intervention	Total sum	Related to PS	Related to ExT	Unrelated to intervention	Total sum	Related to intervention	Unrelated to intervention	Total sum
Han 2021	Gr 1: DP + MET	86	0	0	18	18	NR	NR	NR		0	9	9
	Gr 2: RC	89			23	23			NR		0	10	10
Han 2024	Gr 1: WP + RET	50	0	0	0	0	NR	NR	NR		NR	NR	
	Gr 2: RC	50			0	0			NR		NR	NR	
Hankey 1993	Gr 1: MP + MET	10	0	0	3	3	0	0	2	2	0	2	2
	Gr 2: MET	10		0	3	3		0	1	1	0	1	1
Haub 2002; 2005	Gr 1: Meat + RET	13	0	1	2	3	0	1	0	1	NR	NR	
	Gr 2: SP + RET	13	0	0	2	2	0	0	1	1	NR	NR	
He 2022	Gr 1: WP + RET	75	0	0	0	0	NR	NR	NR		NR	NR	
	Gr 2: RC	75			0	0			NR		NR	NR	
Hegerova 2015	Gr 1: WP + MET	100	0	0	0	0	NR	NR	NR		NR	NR	
	Gr 2: MET	100		0	0	0		NR	NR		NR	NR	
Herda 2021	Gr 1: WP + MET	62	0	2	14	16	3	0	5	8	0	0	0
	Gr 2: PLA + MET	62	0	1	6	7	0	1	2	3	0	0	0
Hofmann 2016;	Gr 1: WP + RET	36	0	0	11	11	0	0	0	0	0	0	0
Oesen 2015;	Gr 2: RET	41		0	10	10		0	0	0	0	0	0
Strasser 2022	Gr 3: RC	40			14	14			0	0	0	0	0
Holm 2008	Gr 1: WP + RET	13	0	0	5	5	NR	NR	NR		NR	NR	
	Gr 2: PLA + RET	16	0	0	4	4	NR	NR	NR		NR	NR	
Holwerda 2018	Gr 1: WP + RET	22	0	1	0	1	0	1	0	1	0	0	0
	Gr 2: PLA + RET	22	0	2	0	2	0	1	0	1	0	0	0
Hotta 2021	Gr 1: WP + MET	10	0	0	0	0	NR	NR	NR		NR	NR	
	Gr 2: MET	10		0	0	0		NR	NR		NR	NR	
Hsieh 2019	Gr 1: MP + MET	77	0	0	22	22	NR	NR	NR		NR	NR	
	Gr 2: MET	79		0	20	20		NR	NR		NR	NR	
	Gr 3: MP	83	0		14	14	NR		NR		NR	NR	
	Gr 4: RC	80			13	13			NR		NR	NR	
Imaoka 2016	Gr 1: WP + MET	23	0	0	4	4	NR	NR	NR		0	0	0
	Gr 2: MET	22		0	0	0		NR	NR		0	0	0
	Gr 3: WP	23	0		6	6	NR		NR		0	1	1
	Gr 4: RC	23			6	6			NR		0	1	1
Imaoka 2019	Gr 1: SP + AET	37	0	0	6	6	0	0	0	0	0	0	0
	Gr 2: AET	37		0	1	1		0	0	0	0	0	0
Jadczak 2021	Gr 1: WP + MET	34	6	0	5	11	8	0	1	9	0	1	1
	Gr 2: Rice + MET	36	3	0	3	6	8	0	2	10	0	2	2

To be continued.

Supplementary table S12. Continued.

Study (year)	Study arm	Group sample (n)	Withdraw, attrition rate, or drop out (number of patients)				Side effects and complications (number of patients)				Serious adverse event (number of patients)		
			Related to PS	Related to ExT	Unrelated to intervention	Total sum	Related to PS	Related to ExT	Unrelated to intervention	Total sum	Related to intervention	Unrelated to intervention	Total sum
Ji 2025a; 2025b	Gr 1: MP + MET	21	0	0	0	0	0	0	0	0	0	0	0
	Gr 2: RC	21			1	1	0	0	0	0	0	0	0
Jiang 2023	Gr 1: WP + MET	52	0	0	0	0	0	0	1	1	0	0	0
	Gr 2: RC	52			0	0			7	7	0	3	3
Jin 2016	Gr 1: DP + AET	25	0	0	0	0	0	0	3	3	0	0	0
	Gr 2: RC	25			0	0			9	9	0	0	0
Jyvakorpi 2023	Gr 1: MP + RET	51	4	0	3	7	NR	NR	NR		NR	NR	
	Gr 2: RET	50		0	0	0		NR	NR		NR	NR	
Kang 2019	Gr 1: WP + RET	66	0	0	0	0	NR	NR	NR		NR	NR	
	Gr 2: RET	49		0	0	0		NR	NR		NR	NR	
Kang 2020	Gr 1: MP + RET	60	0	0	6	6	1	0	2	3	0	0	0
	Gr 2: PLA + RET	60	0	2	3	5	0	0	0	0	0	0	0
Karelis 2015	Gr 1: WP + RET	49	1	3	4	8	1	2	0	3	0	0	0
	Gr 2: Casein + RET	50	1	2	4	7	1	1	0	2	0	0	0
Kemmler 2016	Gr 1: WP + RET(NMES)	25	0	0	4	4	0	0	2	2	0	1	1
	Gr 2: RET (NMES)	25		1	0	1		1	1	2	0	0	0
	Gr 3: RC	25			3	3			0	0	0	1	1
Kemmler 2017	Gr 1: WP + RET (NMES)	33	0	1	2	3	0	0	0	0	0	0	0
	Gr 2: WP	33	1		1	2	0		0	0	0	1	1
	Gr 3: RC	34			3	3			0	0	0	0	0
Kemmler 2020a; 2020b	Gr 1: WP + RET	21	0	0	2	2	NR	NR	NR		NR	NR	
	Gr 2: WP	22	0		2	2	NR		NR		NR	NR	
Kim 2015	Gr 1: MFGM + MET	33	0	0	0	0	0	0	0	0	0	0	0
	Gr 2: PLA + MET	33	0	0	2	2	0	0	1	1	0	0	0
	Gr 3: MFGM	32	0		5	5	0		0	0	0	4	4
	Gr 4: PLA	32	0		1	1	0		0	0	0	0	0
Kirk 2019; 2020	Gr 1: WP + MET	22	0	0	0	0	NR	NR	NR		NR	NR	
	Gr 2: MET	29		2	3	5		NR	NR		NR	NR	
	Gr 3: WP	38	15		0	15	NR		NR		NR	NR	
	Gr 4: RC	31			3	3			NR		NR	NR	
Koopmans 2024a; 2024b	Gr 1: Insect PS + AET	23	6	0	1	7	8	0	0	8	NR	NR	
	Gr 2: WP + AET	24	1	0	0	1	17	0	0	17	NR	NR	
	Gr 3: PLA + AET	20	2	0	1	3	10	0	0	10	NR	NR	
Korzepa 2025	Gr 1: WP + RET	14	0	0	2	2	NR	NR	NR		NR	NR	
	Gr 2: SP (pea) + RET	14	0	0	1	1	NR	NR	NR		NR	NR	
Krause 2019	Gr 1: WP + RET	11	0	0	0	0	NR	NR	NR		NR	NR	
	Gr 2: PLA + RET	10	0	0	0	0	NR	NR	NR		NR	NR	
	Gr 3: WP	7	0		0	0	NR		NR		NR	NR	
	Gr 4: PLA	10	0		0	0	NR		NR		NR	NR	

To be continued.

Supplementary table S12. Continued.

Study (year)	Study arm	Group sample (n)	Withdraw, attrition rate, or drop out (number of patients)				Side effects and complications (number of patients)				Serious adverse event (number of patients)		
			Related to PS	Related to ExT	Unrelated to intervention	Total sum	Related to PS	Related to ExT	Unrelated to intervention	Total sum	Related to intervention	Unrelated to intervention	Total sum
Kukuljan 2009a; 2009b	Gr 1: MP + RET	45	0	0	1	1	0	5	0	5	0	0	0
	Gr 2: RET	46		0	1	1		5	0	5	0	0	0
	Gr 3: MP	45	0		1	1	0		0	0	0	0	0
	Gr 4: RC	44			2	2			0	0	0	0	0
Kuwaba 2023	Gr 1: Collagen + RET	10	0	0	0	0	0	0	2	2	0	0	0
	Gr 2: PLA + RET	10	0	0	2	2	0	0	5	5	0	0	0
Kwon 2015	Gr 1: DP + MET	30	0	0	4	4	NR	NR	NR		NR	NR	
	Gr 2: MET	28		0	3	3		NR	NR		NR	NR	
	Gr 3: RC	31			3	3			NR		NR	NR	
Lamb 2020	Gr 1: SP (peanut) + RET	22	0	1	1	2	NR	NR	NR		NR	NR	
	Gr 2: RET	19		0	0	0		NR	NR		NR	NR	
Laviolette 2010	Gr 1: WP + MET	12	0	1	1	2	NR	NR	NR		NR	NR	
	Gr 2: Casein + MET	10	0	0	0	0	NR	NR	NR		NR	NR	
Leenders 2013	Gr 1: MP + RET	30	0	0	3	3	NR	NR	NR		NR	NR	
	Gr 2: PLA + RET	30	0	0	4	4	NR	NR	NR		NR	NR	
Li DT 2021	Gr 1: DP + RET	30	0	0	0	0	NR	NR	NR		NR	NR	
	Gr 2: DP + AET	30	0	0	0	0	NR	NR	NR		NR	NR	
Li G 2025	Gr 1: DP + RET	7	0	0	0	0	NR	NR	NR		NR	NR	
	Gr 2: RET	7		0	0	0		NR	NR		NR	NR	
Li WL 2022	Gr 1: WP + MET	34	0	0	0	0	NR	NR	NR		NR	NR	
	Gr 2: WP	34	0		0	0	NR		NR		NR	NR	
	Gr 3: RC	34			0	0			NR		NR	NR	
Li Y 2022	Gr 1: DP + MET	40	0	0	0	0	NR	NR	NR		NR	NR	
	Gr 2: MET	40		0	0	0		NR	NR		NR	NR	
Li Z 2021	Gr 1: WP + MET	59	0	0	11	11	NR	NR	NR		NR	NR	
	Gr 2: MET	62		0	25	25		NR	NR		NR	NR	
	Gr 3: WP	61	0		10	10	NR		NR		NR	NR	
	Gr 4: RC	59			26	26			NR		NR	NR	
Liang 2023	Gr 1: WP + RET	25	0	0	0	0	NR	NR	NR		NR	NR	
	Gr 2: DP + MET	25	0	0	0	0	NR	NR	NR		NR	NR	
Liang 2024	Gr 1: WP + RET	25	0	0	0	0	NR	NR	NR		NR	NR	
	Gr 2: RC	25			0	0			NR		NR	NR	
Liao 2019	Gr 1: SP + MET	11	0	1	0	1	NR	NR	NR		NR	NR	
	Gr 2: PLA + MET	11	0	0	0	0	NR	NR	NR		NR	NR	
Liao 2021	Gr 1: WP + RET	36	0	0	1	1	0	0	0	0	0	0	0
	Gr 2: RET	36		0	2	2		0	0	0	0	0	0
Liao 2022	Gr 1: WP + RET	30	0	0	0	0	NR	NR	NR		NR	NR	
	Gr 2: DP + AET	30	0	0	0	0	NR	NR	NR		NR	NR	

To be continued.

Supplementary table S12. Continued.

Study (year)	Study arm	Group sample (n)	Withdraw, attrition rate, or drop out (number of patients)				Side effects and complications (number of patients)				Serious adverse event (number of patients)		
			Related to PS	Related to ExT	Unrelated to intervention	Total sum	Related to PS	Related to ExT	Unrelated to intervention	Total sum	Related to intervention	Unrelated to intervention	Total sum
Liu 2025	Gr 1: Casein-derived peptide + MET	32	0	0	2	2	0	0	0	0	0	0	0
	Gr 2: PLA + MET	33	0	0	7	7	0	0	0	0	0	0	0
Llaneza 2011	Gr 1: SP + AET	43	5	5	0	10	0	0	0	0	0	0	0
	Gr 2: AET	44		7	0	7		0	0	0	0	0	0
Long 2021	Gr 1: DP + RET	60	0	0	0	0	NR	NR	NR		NR	NR	
	Gr 2: RC	60			0	0			NR		NR	NR	
Ma 2023	Gr 1: DP + RET	49	0	0	0	0	NR	NR	NR		NR	NR	
	Gr 2: RC	52			0	0			NR		NR	NR	
Macpherson 2022	Gr 1: WP + MET	73	0	0	5	5	6	8	0	14	0	1	1
	Gr 2: PLA (MP) + MET	74	0	0	3	3	6	7	0	13	0	2	2
Maesta 2007	Gr 1: SP + RET	15	1	0	0	1	1	0	0	1	0	0	0
	Gr 2: PLA (MP) + RET	15	2	0	2	4	2	0	0	2	0	0	0
	Gr 3: SP	15	3		2	5	3		0	3	0	0	0
	Gr 4: PLA (MP)	15	3		1	4	3		0	3	0	0	0
Magrans-Courtney 2011	Gr 1: DP + RET	14	0	0	1	1	NR	NR	NR		NR	NR	
	Gr 2: RET	16		0	1	1		NR	NR		NR	NR	
Malafarina 2017	Gr 1: MP + MET	49	0	0	6	6	NR	NR	NR		0	3	3
	Gr 2: DP + MET	43	0	0	9	9	NR	NR	NR		0	3	3
Maltais 2016	Gr 1: MP + RET	10	0	0	1	1	NR	NR	NR		NR	NR	
	Gr 2: SP + RET	10	0	0	1	1	NR	NR	NR		NR	NR	
	Gr 3: PLA (rice milk) + RET	10	0	0	2	2	NR	NR	NR		NR	NR	
Matsuda 2022	Gr 1: SP + MET	18	1	0	2	3	1	0	2	3	0	3	3
	Gr 2: PLA (BCAA) + MET	21	0	0	0	0	3	0	2	5	0	4	4
McKenna 2021	Gr 1: Meat + RET	28	0	0	7	7	NR	NR	NR		NR	NR	
	Gr 2: PLA + RET	22		1	3	4		NR	NR		NR	NR	
Memelink 2021	Gr 1: WP + MET	62	5	2	4	11	6	0	0	6	0	2	2
	Gr 2: PLA + MET	61	3	0	4	7	11	0	0	11	0	2	2
Meredith 1992	Gr 1: MP + RET	6	0	0	0	0	NR	NR	NR		NR	NR	
	Gr 2: RET	5		0	1	1		NR	NR		NR	NR	
Mertz 2021	Gr 1: WP + RET (high load)	36	0	0	4	4	NR	NR	NR		NR	NR	
	Gr 2: WP + RET (low load)	36	0	0	6	6	NR	NR	NR		NR	NR	
	Gr 3: WP	50	0		6	6	NR		NR		NR	NR	
	Gr 4: Collagen	50	0		6	6	NR		NR		NR	NR	
	Gr 5: PLA (CHO)	36			2	2			NR		NR	NR	
Midttun 2024	Gr 1: WP + RET	72	0	6	17	23	1	1	53	55	0	5	5
	Gr 2: RC	76	0	2	18	20	1	3	40	44	0	3	3

To be continued.

Supplementary table S12. Continued.

Study (year)	Study arm	Group sample (n)	Withdraw, attrition rate, or drop out (number of patients)				Side effects and complications (number of patients)				Serious adverse event (number of patients)		
			Related to PS	Related to ExT	Unrelated to intervention	Total sum	Related to PS	Related to ExT	Unrelated to intervention	Total sum	Related to intervention	Unrelated to intervention	Total sum
Miller EG 2021	Gr 1: WP + RET	98	0	1	10	11	35	11	0	46	0	0	0
	Gr 2: PLA + RET	100		3	17	20		12	0	12	0	0	0
Miller GD 2006; 2012	Gr 1: MP + MET	44	0	0	0	0	0	0	0	0	0	0	0
	Gr 2: RC	43			0	0			0	0	0	0	0
Miller MD 2005; 2006	Gr 1: MP + RET	24	0	0	2	2	NR	NR	NR		0	1	1
	Gr 2: RET	25		0	2	2		NR	NR		0	2	2
	Gr 3: MP	25	0		2	2	NR		NR		0	1	1
	Gr 4: RC	26			1	1			NR		0	0	0
Mitchell 2018	Gr 1: MP + RET	15	0	0	0	0	NR	NR	NR		NR	NR	
	Gr 2: PLA + RET	15	0	0	0	0	NR	NR	NR		NR	NR	
Mogelberg 2022	Gr 1: Collagen + MET	7	0	0	0	0	NR	NR	NR		0	0	0
	Gr 2: MET	6	0	0	3	3		NR	NR		0	3	3
Mojtahedi 2011	Gr 1: WP + AET	15	0	1	1	2	NR	NR	NR		NR	NR	
	Gr 2: PLA + AET	16	0	1	2	3	NR	NR	NR		NR	NR	
Molnar 2016	Gr 1: WP + RET	17	0	0	0	0	NR	NR	NR		NR	NR	
	Gr 2: RET	17		0	0	0		NR	NR		NR	NR	
Mori 2014	Gr 1: WP + MET	14	0	0	2	2	0	0	1	1	0	0	0
	Gr 2: PLA + MET	14	0	0	2	2	0	0	1	1	0	0	0
Mori 2018	Gr 1: WP + RET	27	1	0	3	4	NR	NR	NR		NR	NR	
	Gr 2: RET	27		0	4	4		NR	NR		NR	NR	
	Gr 3: WP	27	1		2	3	NR		NR		NR	NR	
Mori 2020	Gr 1: WP + RET	18	1	1	1	3	NR	NR	NR		NR	NR	
	Gr 2: RET	18		1	2	3		NR	NR		NR	NR	
	Gr 3: WP	18	1		2	3	NR		NR		NR	NR	
Mori 2021	Gr 1: WP + RET	23	1	0	2	3	NR	NR	NR		0	0	0
	Gr 2: RET	23		1	3	4		NR	NR		0	0	0
	Gr 3: WP	23	1		2	3	NR		NR		0	1	1
Mori 2022	Gr 1: WP + RET	27	0	0	2	2	NR	NR	NR		NR	NR	
	Gr 2: RET	27		0	2	2		NR	NR		NR	NR	
	Gr 3: WP	27	0		2	2	NR		NR		NR	NR	
Morikawa 2018	Gr 1: SP + AET	16	0	0	2	2	NR	NR	NR		NR	NR	
	Gr 2: PLA + AET	16	0	0	0	0	NR	NR	NR		NR	NR	
Moyama 2023	Gr 1: DP + RET	83	0	0	26	26	NR	NR	NR		0	1	1
	Gr 2: RET	86		0	27	27	NR	NR	NR		0	1	1
Munk 2021	Gr 1: DP + RET	105	0	0	12	12	NR	NR	NR		0	2	2
	Gr 2: RC	102			4	4			NR		0	1	1
Myint 2013	Gr 1: MP + MET	65	0	0	8	8	7	0	3	10	0	3	3
	Gr 2: MET	61	0	0	5	5	0	0	1	1	0	0	0

To be continued.

Supplementary table S12. Continued.

Study (year)	Study arm	Group sample (n)	Withdraw, attrition rate, or drop out (number of patients)				Side effects and complications (number of patients)				Serious adverse event (number of patients)		
			Related to PS	Related to ExT	Unrelated to intervention	Total sum	Related to PS	Related to ExT	Unrelated to intervention	Total sum	Related to intervention	Unrelated to intervention	Total sum
Murphy 2016	Gr 1: DP + RET	10	0	0	0	0	NR	NR	NR		NR	NR	
	Gr 2: PLA + RET	12	0	2	0	2	NR	NR	NR		NR	NR	
Nabuco 2018; 2019a	Gr 1: WP + RET	47	0	0	4	4	NR	NR	NR		NR	NR	
	Gr 2: PLA + RET	23	0	0	0	0	NR	NR	NR		NR	NR	
Nabuco 2019b	Gr 1: WP + RET	13	0	0	0	0	NR	NR	NR		NR	NR	
	Gr 2: PLA + RET	13	0	0	0	0	NR	NR	NR		NR	NR	
Nabuco 2019c	Gr 1: WP + RET	15	0	0	0	0	NR	NR	NR		NR	NR	
	Gr 2: PLA + RET	15	0	0	0	0	NR	NR	NR		NR	NR	
Nakagawa 2024	Gr 1: WP + RET	31	0	0	0	0	0	0	0	0	0	0	0
	Gr 2: RET	29		0	0	0		0	0	0	0	0	0
Nakayama 2021	Gr 1: MP + RET	63	1	2	0	3	NR	NR	NR		0	0	0
	Gr 2: PLA + RET	63	1	1	1	3	NR	NR	NR		0	1	1
Nambi 2025	Gr 1: DP + MET	38	0	0	2	2	NR	NR	NR		NR	NR	
	Gr 2: PLA + MET	38	0	0	3	3	NR	NR	NR		NR	NR	
Ng 2015	Gr 1: MP + MET	49	0	2	10	12	0	0	2	2	0	6	6
	Gr 2: MET	48		0	6	6		0	3	3	0	3	3
	Gr 3: MP	49	1		9	10	0		4	4	0	2	2
	Gr 4: PLA	50	2		4	6	0		5	5	0	3	3
	Gr 5: RC	50			9	9			2	2	0	5	5
Ni 2019	Gr 1: WP + MET	30	0	0	0	0	NR	NR	NR		NR	NR	
	Gr 2: RC	30			0	0			NR		NR	NR	
Niccoli 2017	Gr 1: WP + MET	27	2	0	3	5	NR	NR	NR		NR	NR	
	Gr 2: MP + MET	26	0	0	1	1	NR	NR	NR		NR	NR	
Niitsu 2016	Gr 1: WP + MET	20	4	0	1	5	NR	NR	NR		NR	NR	
	Gr 2: MET	18	0	1	0	1	NR	NR	NR		NR	NR	
Nilsson 2020	Gr 1: MP + RET	22	0	0	6	6	NR	NR	NR		NR	NR	
	Gr 2: PLA + RET	23	0	0	7	7	NR	NR	NR		NR	NR	
Ninomiya 2023	Gr 1: WP + RET	29	3	0	0	3	NR	NR	NR		NR	NR	
	Gr 2: RET	29		1	1	2		NR	NR		NR	NR	
Oh 2022	Gr 1: MP + RET	25	0	0	4	4	NR	NR	NR		NR	NR	
	Gr 2: PLA + RET	25	0	0	5	5	NR	NR	NR		NR	NR	
Oikawa 2018	Gr 1: WP + AET	16	0	0	0	0	NR	NR	NR		NR	NR	
	Gr 2: Collagen + AET	16	0	0	1	1	NR	NR	NR		NR	NR	
Orsatti 2018	Gr 1: SP + RET	21	0	2	3	5	0	0	0	0	0	0	0
	Gr 2: PLA (MP) + RET	20	0	2	2	4	0	0	0	0	0	0	0
Osuka 2017	Gr 1: MP + RET	28	0	0	1	1	NR	NR	NR		NR	NR	
	Gr 2: MP + MET	28	0	0	1	1	NR	NR	NR		NR	NR	

To be continued.

Supplementary table S12. Continued.

Study (year)	Study arm	Group sample (n)	Withdraw, attrition rate, or drop out (number of patients)				Side effects and complications (number of patients)				Serious adverse event (number of patients)		
			Related to PS	Related to ExT	Unrelated to intervention	Total sum	Related to PS	Related to ExT	Unrelated to intervention	Total sum	Related to intervention	Unrelated to intervention	Total sum
Pan 2022	Gr 1: DP + RET	34	0	0	0	0	NR	NR	NR		NR	NR	
	Gr 2: RC	30			0	0			NR		NR	NR	
Park 2023	Gr 1: DP + MET	14	0	0	1	1	0	0	0	0	0	0	0
	Gr 2: DP + AET	15	0	0	0	0	0	0	0	0	0	0	0
	Gr 3: DP	13	0		2	2	0		0	0	0	0	0
	Gr 4: RC	14			1	1			0	0	0	0	0
Pedersen LR 2019	Gr 1: DP + AET	35	0	0	9	9	NR	NR	NR		NR	NR	
	Gr 2: AET	35		0	6	6		NR	NR		NR	NR	
Pedersen MM 2019	Gr 1: MP + RET	43	0	0	13	13	0	0	0	0	0	0	0
	Gr 2: RC	42			18	18			0	0	0	0	0
Peng 2024	Gr 1: Meat + RET	50	0	1	2	3	NR	NR	NR		NR	NR	
	Gr 2: PLA + RET	50	0	0	0	0	NR	NR	NR		NR	NR	
Puente-Fernandez 2025	Gr 1: WP + RET	11	0	0	1	1	0	0	0	0	0	0	0
	Gr 2: PLA + RET	11	0	0	1	1	0	0	0	0	0	0	0
Rabadi 2008	Gr 1: MP + MET	58	0	0	7	7	0	0	3	3	0	0	0
	Gr 2: PLA + MET	58	0	0	7	7	0	0	1	1	0	2	2
Reidy 2017; 2020	Gr 1: WP + RET	10	0	0	0	0	NR	NR	NR		NR	NR	
	Gr 2: PLA	10	0	0	0	0	NR	NR	NR		NR	NR	
Romera-Liebana 2018	Gr 1: MP + MET	176	0	0	28	28	0	0	2	2	0	6	6
	Gr 2: RC	176			57	57			3	3	0	13	13
Rondanelli 2016	Gr 1: WP + MET	69	0	0	0	0	NR	NR	NR		NR	NR	
	Gr 2: PLA (CHO) + MET	61	0	0	0	0	NR	NR	NR		NR	NR	
Rondanelli 2020	Gr 1: WP + MET	70	6	0	0	6	0	0	0	0	0	0	0
	Gr 2: PLA (CHO) + MET	70	7	0	0	7	0	0	0	0	0	0	0
Roschel 2021	Gr 1: WP + RET	22	0	0	3	3	NR	NR	NR		NR	NR	
	Gr 2: SP + RET	22	0	0	2	2	NR	NR	NR		NR	NR	
	Gr 3: PLA (CHO) + RET	22	0	0	1	1	NR	NR	NR		NR	NR	
Rydwik 2008	Gr 1: DP + MET	25	0	1	10	11	0	0	4	4	0	3	3
	Gr 2: MET	23		1	3	4		0	0	0	0	1	1
	Gr 3: DP	25	0		7	7	0		1	1	0	1	1
	Gr 4: RC	23			9	9			0	0	0	1	1
Sato 2022	Gr 1: MP + MET	8	0	0	1	1	0	0	0	0	0	0	0
	Gr 2: PLA (CHO) + MET	8	0	0	1	1	0	0	0	0	0	0	0
Seino 2017	Gr 1: DP + RET	38	0	0	5	5	0	0	0	0	0	0	0
	Gr 2: RC	39			5	5			0	0	0	0	0
Seino 2018	Gr 1: MP + RET	41	3	0	0	3	2	0	0	2	0	0	0
	Gr 2: RET	41		0	1	1		0	0	0	0	0	0

To be continued.

Supplementary table S12. Continued.

Study (year)	Study arm	Group sample (n)	Withdraw, attrition rate, or drop out (number of patients)				Side effects and complications (number of patients)				Serious adverse event (number of patients)		
			Related to PS	Related to ExT	Unrelated to intervention	Total sum	Related to PS	Related to ExT	Unrelated to intervention	Total sum	Related to intervention	Unrelated to intervention	Total sum
Serra-Prat 2017	Gr 1: DP + MET	80	0	0	19	19	NR	NR	NR		0	0	0
	Gr 2: RC	92			20	20			NR		0	2	2
Shahar 2013	Gr 1: SP + MET	17	0	0	2	2	NR	NR	NR		NR	NR	
	Gr 2: MET	21		0	2	2		NR	NR		NR	NR	
	Gr 3: SP	18	0		3	3	NR		NR		NR	NR	
	Gr 4: RC	19			3	3			NR		NR	NR	
Shenoy 2013	Gr 1: SP + RET	20	0	0	0	0	NR	NR	NR		NR	NR	
	Gr 2: SP	20	0		0	0	NR		NR		NR	NR	
	Gr 3: RC	20			0	0			NR		NR	NR	
Soares 2023	Gr 1: WP + RET	14	0	0	1	1	NR	NR	NR		NR	NR	
	Gr 2: PLA (CHO) + RET	14	0	0	1	1	NR	NR	NR		NR	NR	
Spoelder 2023	Gr 1: WP + AET	15	0	0	0	0	0	0	0	0	0	0	0
	Gr 2: SP + AET	16	0	1	0	1	0	1	0	1	0	0	0
	Gr 3: PLA (CHO) + AET	16	0	0	1	1	0	0	1	1	0	0	0
Sugawara 2010	Gr 1: WP + MET	17	0	0	0	0	NR	NR	NR		NR	NR	
	Gr 2: RC	15			0	0			NR		NR	NR	
Sugawara 2012	Gr 1: WP + MET	18	0	0	1	1	NR	NR	NR		NR	NR	
	Gr 2: RC	18		0	4	4		NR	NR		NR	NR	
Swanenburg 2007	Gr 1: MP + MET	12	0	0	2	2	0	0	0	0	0	1	1
	Gr 2: RC	12			2	2			0	0	0	0	0
Tang 2020	Gr 1: WP + RET	100	0	0	0	0	NR	NR	NR		NR	NR	
	Gr 2: RC	100			0	0			NR		NR	NR	
Tarazona-Santabalbina 2016	Gr 1: DP + MET	51	0	0	11	11	NR	NR	NR		NR	NR	
	Gr 2: DP	49	0		9	9	NR		NR		NR	NR	
Ten Haaf 2019	Gr 1: MP + AET	58	0	0	0	0	3	0	0	3	0	0	0
	Gr 2: PLA (CHO) + AET	56	1	0	1	2	3	0	0	3	0	0	0
Thomson 2016	Gr 1: MP + RET	61	5	10	12	27	3	13	0	16	0	0	0
	Gr 2: SP + RET	64	6	20	12	38	1	13	0	14	0	0	0
	Gr 3: PLA + RET	62	15	13	11	39	4	12	0	16	0	0	0
Travers 2023	Gr 1: DP + MET	89	0	0	10	10	0	0	4	4	NR	NR	
	Gr 2: RC	79			2	2			0	0	NR	NR	
Trevisan 2010	Gr 1: SP + RET	15	0	0	0	0	NR	NR	NR		NR	NR	
	Gr 2: PLA (CHO) + RET	15	0	0	0	0	NR	NR	NR		NR	NR	
	Gr 3: SP	15	0		0	0	NR		NR		NR	NR	
	Gr 4: PLA (CHO)	15	0		0	0	NR		NR		NR	NR	
Tsurumi 2022	Gr 1: MP + RET(NMES)	11	0	0	2	2	NR	NR	NR		NR	NR	
	Gr 2: MP	11	0		1	1	NR		NR		NR	NR	

To be continued.

Supplementary table S12. Continued.

Study (year)	Study arm	Group sample (n)	Withdraw, attrition rate, or drop out (number of patients)				Side effects and complications (number of patients)				Serious adverse event (number of patients)		
			Related to PS	Related to ExT	Unrelated to intervention	Total sum	Related to PS	Related to ExT	Unrelated to intervention	Total sum	Related to intervention	Unrelated to intervention	Total sum
Uchida 2024	Gr 1: Meat + RET	24	0	2	4	6	0	0	0	0	0	1	1
	Gr 2: PLA (CHO) + RET	23	0	2	1	3	0	0	1	1	0	0	0
	Gr 3: Meat	23	1		0	1	0		0	0	0	0	0
	Gr 4: PLA (CHO)	23	0		2	2	0		0	0	0	2	2
Unterberger 2022	Gr 1: SP + RET	48	0	0	9	9	0	0	0	0	0	0	0
	Gr 2: PLA (CHO) + RET	41	0	0	5	5	0	0	0	0	0	0	0
	Gr 3: RC	47			6	6			0	0	0	0	0
van den Helder 2020	Gr 1: DP + MET	68	0	5	25	30	0	0	0	0	0	1	1
	Gr 2: MET	65		2	17	19		0	0	0	0	1	1
	Gr 3: RC	91			13	13			0	0	0	5	5
van Dongen 2020	Gr 1: DP + RET	82	0	3	5	8	NR	NR	NR		NR	NR	
	Gr 2: DP + MET	74	0	1	2	3	NR	NR	NR		NR	NR	
	Gr 3: RC	86			5	5			NR		NR	NR	
Verceles 2023	Gr 1: WP + MET	16	0	0	8	8	NR	NR	NR		NR	NR	
	Gr 2: MET	23		0	12	12		NR	NR		NR	NR	
Verdijk 2009	Gr 1: Casein + RET	14	0	0	1	1	NR	NR	NR		NR	NR	
	Gr 2: PLA(water) + RET	14	0	0	1	1	NR	NR	NR		NR	NR	
Verreijen 2015	Gr 1: WP + RET	40	0	3	5	8	0	3	0	3	0	0	0
	Gr 2: PLA (CHO) + RET	40	0	3	4	7	0	3	0	3	0	0	0
Verreijen 2017	Gr 1: DP + RET	32	0	0	10	10	0	0	0	0	0	0	0
	Gr 2: RET	29		0	10	10		0	0	0	0	0	0
	Gr 3: DP	31	0		18	18	0		0	0	0	0	0
	Gr 4: RC	30			16	16			1	1	0	0	0
Vijayakumaran 2023	Gr 1: WP + RET	8	0	0	0	0	0	0	0	0	0	0	0
	Gr 2: RET	8		0	0	0		0	0	0	0	0	0
Vikberg 2019	Gr 1: MP + RET	36	0	0	5	5	0	4	1	5	0	0	0
	Gr 2: RC	34			1	1			0	0	0	0	0
Villanueva 2014	Gr 1: WP + Cre + RET	8	0	0	1	1	0	0	0	0	0	0	0
	Gr 2: RET	8		0	1	1		0	0	0	0	0	0
	Gr 3: RC	9			1	1			0	0	0	0	0
Vorup 2017	Gr 1: MP + MET	23	1	2	7	10	1	1	0	2	0	0	0
	Gr 2: PLA + MET	22	0	3	1	4	0	1	0	1	0	0	0
	Gr 3: RC	22			5	5			0	0	0	0	0
van de Bool 2017	Gr 1: WP + MET	42	0	0	4	4	5	0	2	7	0	1	1
	Gr 2: PLA + MET	39	0	1	3	4	1	0	5	6	0	0	0

To be continued.

Supplementary table S12. Continued.

Study (year)	Study arm	Group sample (n)	Withdraw, attrition rate, or drop out (number of patients)				Side effects and complications (number of patients)				Serious adverse event (number of patients)		
			Related to PS	Related to ExT	Unrelated to intervention	Total sum	Related to PS	Related to ExT	Unrelated to intervention	Total sum	Related to intervention	Unrelated to intervention	Total sum
Wang ZY 2022	Gr 1: DP + MET	60	0	0	10	10	NR	NR	NR		NR	NR	
	Gr 2: MET	62		0	12	12		NR	NR		NR	NR	
	Gr 3: DP	58	0		8	8	NR		NR		NR	NR	
	Gr 4: RC	54			3	3			NR		NR	NR	
Wang XJ 2023	Gr 1: WP + RET	75	0	0	0	0	NR	NR	NR		NR	NR	
	Gr 2: DP + AET	75	0	0	0	0	NR	NR	NR		NR	NR	
Weinheimer 2012	Gr 1: WP + MET	201	22	10	50	82	0	0	1	1	0	0	0
	Gr 2: PLA (CHO) + MET	126	0	8	48	56	0	1	1	2	0	0	0
Weisgarber 2015	Gr 1: WP + RET	17	0	0	2	2	0	0	0	0	0	0	0
	Gr 2: PLA (CHO) + RET	17	0	0	3	3	0	0	0	0	0	0	0
Wu 2018	Gr 1: SP + MET	10	0	0	1	1	NR	NR	NR		NR	NR	
	Gr 2: DP + MET	10	0	0	0	0	NR	NR	NR		NR	NR	
	Gr 3: PLA	10	2		0	2	NR		NR		NR	NR	
	Gr 4: RC	10			1	1			NR		NR	NR	
Wu 2019	Gr 1: DP + RET	40	0	0	0	0	NR	NR	NR		NR	NR	
	Gr 2: DP	40	0		0	0	NR		NR		NR	NR	
Xie 2021	Gr 1: WP + MET	40	0	0	0	0	NR	NR	NR		NR	NR	
	Gr 2: RC	30			0	0			NR		NR	NR	
Xing 2018	Gr 1: WP + MET	84	0	0	0	0	0	0	0	0	0	0	0
	Gr 2: DP + AET	85	0	0	1	1	0	0	0	0	0	1	1
Xu 2021	Gr 1: DP + MET	88	0	0	0	0	NR	NR	NR		NR	NR	
	Gr 2: RC	89			0	0			NR		NR	NR	
Xue 2023	Gr 1: WP + RET	15	0	0	3	3	0	0	0	0	0	0	0
	Gr 2: RET	15		0	1	1		0	0	0	0	0	0
	Gr 3: WP	15	0		2	2	0		0	0	0	0	0
	Gr 4: RC	15			4	4			1	1	0	0	0
Yamada 2015	Gr 1: MP + AET	79	0	0	2	2	0	20	0	20	0	1	1
	Gr 2: AET	71		0	1	1		20	0	20	0	0	0
	Gr 3: RC	77			2	2			0	0	0	1	1
Yamada 2019	Gr 1: WP + RET	28	2	2	2	6	0	0	0	0	0	0	0
	Gr 2: RET	28		2	2	4		0	0	0.0	0	0	0
	Gr 3: WP	28	4		2	6	0		0	0.0	0	0	0
	Gr 4: RC	28			2	2			0	0.0	0	0	0
Yin 2019	Gr 1: DP + RET	50	0	0	0	0	NR	NR	NR		NR	NR	
	Gr 2: RET	50		0	0	0		NR	NR		NR	NR	
Yoshimura 2016	Gr 1: MP + RET	20	0	0	1	1	0	0	0	0	0	0	0
	Gr 2: PLA + RET	19	0	0	2	2	0	0	1	1	0	0	0

To be continued.

Supplementary table S12. Continued.

Study (year)	Study arm	Group sample (n)	Withdraw, attrition rate, or drop out (number of patients)				Side effects and complications (number of patients)				Serious adverse event (number of patients)		
			Related to PS	Related to ExT	Unrelated to intervention	Total sum	Related to PS	Related to ExT	Unrelated to intervention	Total sum	Related to intervention	Unrelated to intervention	Total sum
Zak 2009	Gr 1: MP + RET	22	0	0	3	3	NR	NR	NR		NR	NR	
	Gr 2: MP + AET	23	0	0	4	4	NR	NR	NR		NR	NR	
	Gr 3: PLA (CHO) + RET	23	0	0	2	2	NR	NR	NR		NR	NR	
	Gr 4: PLA (CHO) + AET	23	0	0	2	2	NR	NR	NR		NR	NR	
Zdzieblik 2015	Gr 1: Collagen + RET	30	2	2	0	4	0	0	0	0	0	0	0
	Gr 2: PLA (silica) + RET	30	1	2	0	3	0	0	0	0	0	0	0
Zdzieblik 2021	Gr 1: WP + RET	40	0	2	2	4	0	0	0	0	0	0	0
	Gr 2: Collagen + RET	40	0	7	3	10	0	0	0	0	0	0	0
	Gr 3: PLA (silica) + RET	40	0	7	2	9	0	0	0	0	0	0	0
Zhang 2023a	Gr 1: MP + RET	39	0	0	0	0	0	0	4	4	0	0	0
	Gr 2: RC	39			0	0			14	14	0	0	0
Zhang 2023b	Gr 1: DP + RET	39	0	0	0	0	0	0	2	2	0	0	0
	Gr 2: RC	39			0	0			9	9	0	0	0
Zhao YF 2021	Gr 1: DP + MET	41	0	0	0	0	0	2	0	2	0	0	0
	Gr 2: DP	21	0		0	0	0		0	0	0	0	0
Zhao YY 2021	Gr 1: DP + RET	42	0	0	0	0	NR	NR	NR		NR	NR	
	Gr 2: RET	42		0	0	0		NR	NR		NR	NR	
Zhao 2022	Gr 1: WP + RET	20	0	0	0	0	NR	NR	NR		NR	NR	
	Gr 2: WP	20	0		0	0.0	NR		NR		NR	NR	
	Gr 3: RC	20			0	0.0			NR		NR	NR	
Zhou 2023	Gr 1: DP + RET	250	0	0	0	0	NR	NR	NR		NR	NR	
	Gr 2: DP	250	0		0	0	NR		NR		NR	NR	
Zhu 2019	Gr 1: SP + MET	36	1	1	6	8	0	0	1	1	0	0	0
	Gr 2: MET	40		5	11	16		0	1	1	0	2	2
	Gr 3: RC	37			13	13			2	2.0	0	4	4
Zong 2023	Gr 1: WP + MET	30	3	0	0	3	3	0	0	3	0	0	0
	Gr 2: MET	30		0	1	1		0	1	1	0	0	0
	Gr 3: RC	30			2	2.0			1	1.0	0	1	1

AET, aerobic exercise training; BCAA, branched-chain amino acids; CHO, carbohydrates; Cre, creatine; DP, dietary protein; ExT, exercise training; MET, multicomponent exercise training; NMES, neuromuscular electrical stimulation; MP, milk protein; NR, not reported. PLA, placebo supplementation; PS, protein supplementation; RC, regular care; REHC, restricted energy higher carbohydrate; RET, resistance exercise training ; SMD, standardized mean difference; SP, soy protein; STS, sit to stand; WP, whey protein; ω3FA, omega-3 fatty acids.

Supplementary table S13. GRADE certainty rating of treatment efficacy for muscle mass gain.

Treatment (Common comparator: RC)	Involved studies for direct evidence (n)[a]	Participants (n)	Study limitation: Contribution of risks of bias of direct estimates to network estimates – High	Mod	Low	Study limitation: Judgment	Inconsistency (incoherence): Treatment effect, SMD (95%CI) – Direct estimate	Indirect estimate	Network estimate	Inconsistency: Judgment	Indirection (transitivity): PICO assessment – Participant (P)	Intervention (I)	Comparison (C)	Outcome (O)	Indirection: Judgment	Imprecision: Network estimate	Imprecision: Judgment	Publication bias: Statistical test[b]	Publication bias: Judgment	Certainty of evidence[c]
A. Combined therapy																				
(a) Protein supplementation plus RET																				
Casein+RET	0		8.52%	88.64%	2.84%	No serious		0.88 [0.07; 1.68)	0.88 [0.07; 1.68)	No serious	Elder people	PS+RET	RC, PLA, AC	LBM	No serious	0.88 [0.07; 1.68)	No serious	*p* < 0.01	Strongly suspected	⊕⊕⊕⊖
Collagen+RET	1	19	9.09%	78.82%	12.09%	No serious	2.08 [0.47; 3.70]	1.06 [0.32; 1.79]	1.23 [0.56; 1.90]	No serious	Elder people	PS+RET	RC, PLA, AC	LBM	No serious	1.23 [0.56; 1.90]	No serious	*p* < 0.01	Strongly suspected	⊕⊕⊕⊖
DP+RET	11	897	60.48%	38.94%	0.58%	Serious	0.84 [0.44; 1.24]	1.09 [0.67; 1.51]	0.96 [0.67; 1.25]	No serious	Elder people	PS+RET	RC, PLA, AC	LBM	No serious	0.96 [0.67; 1.25]	No serious	*p* < 0.01	Strongly suspected	⊕⊕⊖⊖
Meat+RET	2	78	19.01%	80.25%	0.74%	No serious	1.06 [0.08; 2.04]	1.20 [0.62; 1.78]	1.16 [0.66; 1.66]	No serious	Elder people	PS+RET	RC, PLA, AC	LBM	No serious	1.16 [0.66; 1.66]	No serious	*p* < 0.01	Strongly suspected	⊕⊕⊕⊖
MP+RET	5	277	29.87%	62.17%	7.96%	No serious	0.90 [0.27; 1.52]	1.20 [0.87; 1.54]	1.14 [0.84; 1.43]	No serious	Elder people	PS+RET	RC, PLA, AC	LBM	No serious	1.14 [0.84; 1.43]	No serious	*p* < 0.01	Strongly suspected	⊕⊕⊕⊖
Rice+RET	0		15.84%	80.40%	3.76%	No serious		0.47 [-0.31; 1.26)	0.47 [-0.31; 1.26)	No serious	Elder people	PS+RET	RC, PLA, AC	LBM	No serious	0.47 [-0.31; 1.26)	Serious	*p* < 0.01	Strongly suspected	⊕⊕⊖⊖
SP+RET	7	333	12.02%	83.71%	4.27%	No serious	0.95 [0.42; 1.47]	1.19 [0.74; 1.64]	1.09 [0.74; 1.43]	No serious	Elder people	PS+RET	RC, PLA, AC	LBM	No serious	1.09 [0.74; 1.43]	No serious	*p* < 0.01	Strongly suspected	⊕⊕⊕⊖
WP+RET	18	776	10.97%	83.89%	5.14%	No serious	1.31 [0.96; 1.65]	1.29 [1.01; 1.57]	1.29 [1.08; 1.51]	No serious	Elder people	PS+RET	RC, PLA, AC	LBM	No serious	1.29 [1.08; 1.51]	No serious	*p* < 0.01	Strongly suspected	⊕⊕⊕⊖
(b) Protein supplementation plus AET																				
Collagen+AET	0		7.92%	43.22%	48.86%	No serious		0.79 [-0.39; 1.97)	0.79 [-0.39; 1.97)	No serious	Elder people	PS+AET	RC, PLA, AC	LBM	No serious	0.79 [-0.39; 1.97)	Serious	*p* < 0.01	Strongly suspected	⊕⊕⊖⊖
DP+AET	1	50	29.01%	68.03%	2.96%	No serious	0.96 [-0.40; 2.31]	0.08 [-0.55; 0.71]	0.23 [-0.34; 0.80]	No serious	Elder people	PS+AET	RC, PLA, AC	LBM	No serious	0.23 [-0.34; 0.80]	Serious	*p* < 0.01	Strongly suspected	⊕⊕⊖⊖
Ins-PS+AET	0		8.55%	77.61%	13.84%	No serious		0.83 [-0.48; 2.14)	0.83 [-0.48; 2.14)	No serious	Elder people	PS+AET	RC, PLA, AC	LBM	No serious	0.83 [-0.48; 2.14)	Serious	*p* < 0.01	Strongly suspected	⊕⊕⊖⊖
MP+AET	2	152	35.69%	57.98%	6.33%	No serious	1.26 [0.32; 2.20]	-0.33 [-1.20; 0.53]	0.39 [-0.24; 1.03]	Serious	Elder people	PS+AET	RC, PLA, AC	LBM	No serious	0.39 [-0.24; 1.03]	Serious	*p* < 0.01	Strongly suspected	⊕⊖⊖⊖
SP+AET	0		11.54%	78.32%	10.14%	No serious		0.52 [-0.18; 1.22)	0.52 [-0.18; 1.22)	No serious	Elder people	PS+AET	RC, PLA, AC	LBM	No serious	0.52 [-0.18; 1.22)	Serious	*p* < 0.01	Strongly suspected	⊕⊕⊖⊖
WP+AET	2	46	8.59%	63.88%	27.53%	No serious	-2.41 [-3.72; -1.11]	1.19 [0.45; 1.93]	0.31 [-0.34; 0.95]	Serious	Elder people	PS+AET	RC, PLA, AC	LBM	No serious	0.31 [-0.34; 0.95]	Serious	*p* < 0.01	Strongly suspected	⊕⊖⊖⊖
(c) Protein supplementation plus MET																				
Casein+MET	1	18	19.03%	54.35%	26.62%	No serious	0.75 [-0.79; 2.29]	0.63 [-0.11; 1.37]	0.65 [-0.02; 1.32]	No serious	Elder people	PS+MET	RC, PLA, AC	LBM	No serious	0.65 [-0.02; 1.32]	Serious	*p* < 0.01	Strongly suspected	⊕⊕⊖⊖
Collegen+MET	0		43.59%	55.41%	1.00%	No serious		0.59 [-1.26; 2.44)	0.59 [-1.26; 2.44)	No serious	Elder people	PS+MET	RC, PLA, AC	LBM	No serious	0.59 [-1.26; 2.44)	Serious	*p* < 0.01	Strongly suspected	⊕⊕⊖⊖
DP+MET	13	2525	15.33%	83.34%	1.33%	No serious	0.30 [-0.06; 0.66]	0.52 [0.05; 0.99]	0.38 [0.09; 0.67]	No serious	Elder people	PS+MET	RC, PLA, AC	LBM	No serious	0.38 [0.09; 0.67]	No serious	*p* < 0.01	Strongly suspected	⊕⊕⊕⊖
Meat+MET	1	17	27.50%	71.85%	0.65%	No serious	0.06 [-1.50; 1.61]	0.68 [-0.44; 1.80]	0.47 [-0.44; 1.38]	No serious	Elder people	PS+MET	RC, PLA, AC	LBM	No serious	0.47 [-0.44; 1.38]	Serious	*p* < 0.01	Strongly suspected	⊕⊕⊖⊖
MP+MET	7	291	42.29%	49.49%	8.22%	No serious	0.62 [0.08; 1.15]	0.76 [0.32; 1.21]	0.70 [0.36; 1.05]	No serious	Elder people	PS+MET	RC, PLA, AC	LBM	No serious	0.70 [0.36; 1.05]	No serious	*p* < 0.01	Strongly suspected	⊕⊕⊕⊖
Oat+MET	0		7.67%	53.90%	38.43%	No serious		0.25 [-1.25; 1.74)	0.25 [-1.25; 1.74)	No serious	Elder people	PS+MET	RC, PLA, AC	LBM	No serious	0.25 [-1.25; 1.74)	Serious	*p* < 0.01	Strongly suspected	⊕⊕⊖⊖
Rice+MET	0		7.67%	89.47%	2.86%	No serious		0.04 [-1.33; 1.41)	0.04 [-1.33; 1.41)	No serious	Elder people	PS+MET	RC, PLA, AC	LBM	No serious	0.04 [-1.33; 1.41)	Serious	*p* < 0.01	Strongly suspected	⊕⊕⊖⊖
SP+MET	5	265	5.80%	93.43%	0.77%	No serious	0.65 [0.03; 1.26]	0.83 [0.04; 1.61]	0.72 [0.23; 1.20]	No serious	Elder people	PS+MET	RC, PLA, AC	LBM	No serious	0.72 [0.23; 1.20]	No serious	*p* < 0.01	Strongly suspected	⊕⊕⊕⊖
WP+MET	12	600	10.81%	85.16%	4.03%	No serious	0.80 [0.40; 1.20]	0.61 [0.24; 0.97]	0.70 [0.43; 0.96]	No serious	Elder people	PS+MET	RC, PLA, AC	LBM	No serious	0.70 [0.43; 0.96]	No serious	*p* < 0.01	Strongly suspected	⊕⊕⊕⊖
B. Monotherapy																				
(a) Exercise training alone																				
RET	26	988	15.49%	83.27%	1.24%	No serious	0.81 [0.53; 1.09]	0.42 [0.13; 0.71]	0.62 [0.42; 0.82]	No serious	Elder people	RET	RC, PLA, AC	LBM	No serious	0.62 [0.42; 0.82]	No serious	*p* < 0.01	Strongly suspected	⊕⊕⊕⊖
AET	4	191	13.92%	73.57%	12.51%	No serious	0.43 [-0.31; 1.16]	0.17 [-0.47; 0.81]	0.28 [-0.20; 0.76]	No serious	Elder people	AET	RC, PLA, AC	LBM	No serious	0.28 [-0.20; 0.76]	Serious	*p* < 0.01	Strongly suspected	⊕⊕⊖⊖
MET	18	896	10.13%	88.60%	1.27%	No serious	0.24 [-0.08; 0.57]	0.21 [-0.15; 0.57]	0.23 [-0.01; 0.47]	No serious	Elder people	MET	RC, PLA, AC	LBM	No serious	0.23 [-0.01; 0.47]	Serious	*p* < 0.01	Strongly suspected	⊕⊕⊖⊖
(b) Protein supplementation alone																				
Casein	0		8.56%	52.28%	39.16%	No serious		0.28 [-1.23; 1.78)	0.28 [-1.23; 1.78)	No serious	Elder people	PS	RC, PLA, AC	LBM	No serious	0.28 [-1.23; 1.78)	Serious	*p* < 0.01	Strongly suspected	⊕⊕⊖⊖
Collagen	1	78	6.38%	39.77%	53.85%	No serious	0.09 [-1.21; 1.39]	0.63 [-0.39; 1.65]	0.43 [-0.38; 1.23]	No serious	Elder people	PS	RC, PLA, AC	LBM	No serious	0.43 [-0.38; 1.23]	Serious	*p* < 0.01	Strongly suspected	⊕⊕⊖⊖
DP	5	271	52.06%	46.71%	1.23%	Serious	0.32 [-0.29; 0.94]	0.17 [-0.32; 0.65]	0.23 [-0.15; 0.61]	No serious	Elder people	PS	RC, PLA, AC	LBM	No serious	0.23 [-0.15; 0.61]	Serious	*p* < 0.01	Strongly suspected	⊕⊖⊖⊖
Meat	3	103	13.48%	86.09%	0.43%	No serious	0.28 [-0.54; 1.10]	0.81 [-0.18; 1.79]	0.50 [-0.14; 1.13]	No serious	Elder people	PS	RC, PLA, AC	LBM	No serious	0.50 [-0.14; 1.13]	Serious	*p* < 0.01	Strongly suspected	⊕⊕⊖⊖
MP	4	272	15.00%	74.78%	10.22%	No serious	0.27 [-0.40; 0.93]	0.41 [-0.13; 0.94]	0.35 [-0.07; 0.77]	No serious	Elder people	PS	RC, PLA, AC	LBM	No serious	0.35 [-0.07; 0.77]	Serious	*p* < 0.01	Strongly suspected	⊕⊕⊖⊖
SP	7	278	9.88%	88.47%	1.65%	No serious	-0.01 [-0.54; 0.51]	0.47 [-0.08; 1.03]	0.22 [-0.16; 0.60]	No serious	Elder people	PS	RC, PLA, AC	LBM	No serious	0.22 [-0.16; 0.60]	Serious	*p* < 0.01	Strongly suspected	⊕⊕⊖⊖
WP	18	652	12.01%	83.81%	4.18%	No serious	0.35 [0.02; 0.69]	0.18 [-0.21; 0.56]	0.28 [0.02; 0.53]	No serious	Elder people	PS	RC, PLA, AC	LBM	No serious	0.28 [0.02; 0.53]	No serious	*p* < 0.01	Strongly suspected	⊕⊕⊕⊖

[a]Values present the number of studies which provided direct comparisons for each indicated treatment arm versus the regular control.

[b]Performed by Begg–Mazumdar rank correlation test

[c]Certainty of evidence is graded as follows: High: ⊕⊕⊕⊕; Moderate: ⊕⊕⊕⊖; Low: ⊕⊕⊖⊖; Very low: ⊕⊖⊖⊖.

AC, active comparater; AET, aerobic exercise training; 95% CI, 95% confidence interval; DP, dietary protein; GRADE, Grading of Recommendations, Assessment, Development and Evaluations; Ins-PS, insect protein supplement; LBM, lean body mass; MET, multicomponent exercise training; MP, milk protein; PLA, placebo supplementation; PS, protein supplementation; RC, regular care; RET, resistance exercise training ; SMD, standardized mean difference; SP, soy protein; WP, whey protein.

Supplementary table S14. GRADE certainty rating of treatment efficacy for handgrip strength outcome.

Treatment (Common comparator: RC)	Involved studies for direct evidence (n)[a]	Participants (n)	Study limitation				Inconsistency (incoherence)				Indirection (transitivity)					Imprecision		Publication bias		Certainty of evidence[c]
			Contribution of risks of bias of direct estimates to network estimates			Judgment	Treatment effect, SMD (95%CI)			Judgment	PICO assessment				Judgment	Network estimate	Judgment	Statistical test[b]	Judgment	
			High	Mod	Low		Direct estimate	Indirect estimate	Network estimate		Participant (P)	Intervention (I)	Comparison (C)	Outcome (O)						
A. Combined therapy																				
(a) Protein supplementation plus RET																				
Casein+RET	0		8.52%	88.64%	2.84%	No serious		0.88 [0.07; 1.68)	0.88 [0.07; 1.68)	No serious	Elder people	PS+RET	RC, PLA, AC	HGS	No serious	0.54 [-0.25; 1.32]	Serious	*p = 0.13*	Undetected	⊕⊕⊕⊖
Collagen+RET	1	19	9.09%	78.82%	12.09%	No serious	2.08 [0.47; 3.70]	1.06 [0.32; 1.79]	1.23 [0.56; 1.90]	No serious	Elder people	PS+RET	RC, PLA, AC	HGS	No serious	0.74 [-0.48; 1.97]	Serious	*p = 0.13*	Undetected	⊕⊕⊕⊖
DP+RET	11	897	60.48%	38.94%	0.58%	Serious	0.84 [0.44; 1.24]	1.09 [0.67; 1.51]	0.96 [0.67; 1.25]	No serious	Elder people	PS+RET	RC, PLA, AC	HGS	No serious	0.74 [0.47; 1.00]	No serious	*p = 0.13*	Undetected	⊕⊕⊕⊖
Meat+RET	2	78	19.01%	80.25%	0.74%	No serious	1.06 [0.08; 2.04]	1.20 [0.62; 1.78]	1.16 [0.66; 1.66]	No serious	Elder people	PS+RET	RC, PLA, AC	HGS	No serious	0.76 [-0.05; 1.56]	Serious	*p = 0.13*	Undetected	⊕⊕⊕⊖
MP+RET	5	277	29.87%	62.17%	7.96%	No serious	0.90 [0.27; 1.52]	1.20 [0.87; 1.54]	1.14 [0.84; 1.43]	No serious	Elder people	PS+RET	RC, PLA, AC	HGS	No serious	0.64 [0.34; 0.95]	No serious	*p = 0.13*	Undetected	⊕⊕⊕⊕
SP+RET	0		15.84%	80.40%	3.76%	No serious		0.47 [-0.31; 1.26)	0.47 [-0.31; 1.26)	No serious	Elder people	PS+RET	RC, PLA, AC	HGS	No serious	0.94 [0.47; 1.41]	No serious	*p = 0.13*	Undetected	⊕⊕⊕⊕
WP+RET	7	333	12.02%	83.71%	4.27%	No serious	0.95 [0.42; 1.47]	1.19 [0.74; 1.64]	1.09 [0.74; 1.43]	No serious	Elder people	PS+RET	RC, PLA, AC	HGS	No serious	0.86 [0.63; 1.08]	No serious	*p = 0.13*	Undetected	⊕⊕⊕⊕
(b) Protein supplementation plus AET																				
DP+AET	2	79	38.46%	61.07%	0.47%	No Serious	1.27 [0.42; 2.12]	-0.91 [-1.56; -0.27]	-0.12 [-0.64; 0.39]	Serious	Elder people	PS+AET	RC, PLA, AC	HGS	No serious	-0.12 [-0.64; 0.39]	Serious	*p = 0.13*	Undetected	⊕⊕⊖⊖
Insect-PS+AET	0		23.72%	76.06%	0.22%	No Serious		0.61 [-0.49; 1.71]	0.61 [-0.49; 1.71]	No serious	Elder people	PS+AET	RC, PLA, AC	HGS	No serious	0.61 [-0.49; 1.71]	Serious	*p = 0.13*	Undetected	⊕⊕⊕⊖
MP+AET	2	56	8.55%	91.31%	0.14%	No Serious	0.67 [-0.23; 1.57]	0.43 [-0.60; 1.47]	0.57 [-0.11; 1.25]	No serious	Elder people	PS+AET	RC, PLA, AC	HGS	No serious	0.57 [-0.11; 1.25]	Serious	*p = 0.13*	Undetected	⊕⊕⊕⊖
SP+AET	0		21.32%	78.50%	0.18%	No Serious		0.18 [-0.96; 1.31]	0.18 [-0.96; 1.31]	No serious	Elder people	PS+AET	RC, PLA, AC	HGS	No serious	0.18 [-0.96; 1.31]	Serious	*p = 0.13*	Undetected	⊕⊕⊕⊖
WP+AET	0		37.12%	62.69%	0.19%	No Serious		0.40 [-0.45; 1.24]	0.40 [-0.45; 1.24]	No serious	Elder people	PS+AET	RC, PLA, AC	HGS	No serious	0.40 [-0.45; 1.24]	Serious	*p = 0.13*	Undetected	⊕⊕⊕⊖
(c) Protein supplementation plus MET																				
Casein+MET	1	79	16.65%	82.95%	0.40%	No Serious	0.17 [-0.91; 1.25]	0.03 [-0.69; 0.74]	0.07 [-0.53; 0.67]	No serious	Elder people	PS+MET	RC, PLA, AC	HGS	No serious	0.07 [-0.53; 0.67]	Serious	*p = 0.13*	Undetected	⊕⊕⊕⊖
Collagen+MET	0		40.87%	58.93%	0.20%	No Serious		0.08 [-1.61; 1.77]	0.08 [-1.61; 1.77]	No serious	Elder people	PS+MET	RC, PLA, AC	HGS	No serious	0.08 [-1.61; 1.77]	Serious	*p = 0.13*	Undetected	⊕⊕⊕⊖
DP+MET	13	2324	5.99%	93.82%	0.19%	No Serious	0.12 [-0.18; 0.42]	0.43 [-0.04; 0.89]	0.21 [-0.04; 0.46]	No serious	Elder people	PS+MET	RC, PLA, AC	HGS	No serious	0.21 [-0.04; 0.46]	Serious	*p = 0.13*	Undetected	⊕⊕⊕⊖
Meat+MET	1	17	1.59%	98.33%	0.08%	No Serious	-0.55 [-1.94; 0.84]	-0.17 [-2.45; 2.11]	-0.45 [-1.63; 0.74]	No serious	Elder people	PS+MET	RC, PLA, AC	HGS	No serious	-0.45 [-1.63; 0.74]	Serious	*p = 0.13*	Undetected	⊕⊕⊕⊖
MP+MET	5	725	2.26%	91.05%	6.69%	No Serious	0.33 [-0.16; 0.81]	0.19 [-0.24; 0.63]	0.25 [-0.07; 0.58]	No serious	Elder people	PS+MET	RC, PLA, AC	HGS	No serious	0.25 [-0.07; 0.58]	Serious	*p = 0.13*	Undetected	⊕⊕⊕⊖
Rice+MET	0		3.23%	96.05%	0.72%	No Serious		0.40 [-0.39; 1.20]	0.40 [-0.39; 1.20]	No serious	Elder people	PS+MET	RC, PLA, AC	HGS	No serious	0.40 [-0.39; 1.20]	Serious	*p = 0.13*	Undetected	⊕⊕⊕⊖
SP+MET	3	123	6.49%	93.37%	0.14%	No Serious	0.25 [-0.45; 0.96]	0.71 [-0.31; 1.74]	0.40 [-0.18; 0.98]	No serious	Elder people	PS+MET	RC, PLA, AC	HGS	No serious	0.40 [-0.18; 0.98]	Serious	*p = 0.13*	Undetected	⊕⊕⊕⊖
WP+MET	12	591	6.74%	89.56%	3.70%	No Serious	0.61 [0.26; 0.95]	0.72 [0.35; 1.10]	0.66 [0.41; 0.91]	No serious	Elder people	PS+MET	RC, PLA, AC	HGS	No serious	0.66 [0.41; 0.91]	No serious	*p = 0.13*	Undetected	⊕⊕⊕⊕
B. Monotherapy																				
(a) Exercise training alone																				
RET	15	626	12.66%	87.19%	0.15%	No Serious	0.40 [0.09; 0.70]	0.60 [0.30; 0.90]	0.50 [0.28; 0.71]	No serious	Elder people	RET	RC, PLA, AC	HGS	No serious	0.50 [0.28; 0.71]	No serious	*p = 0.13*	Undetected	⊕⊕⊕⊕
AET	0		20.47%	79.28%	0.25%	No Serious		0.35 [-0.53; 1.23]	0.35 [-0.53; 1.23]	No serious	Elder people	AET	RC, PLA, AC	HGS	No serious	0.35 [-0.53; 1.23]	Serious	*p = 0.13*	Undetected	⊕⊕⊕⊖
MET	14	894	4.70%	95.04%	0.26%	No Serious	0.41 [0.10; 0.72]	-0.08 [-0.45; 0.28]	0.20 [-0.03; 0.43]	Serious	Elder people	MET	RC, PLA, AC	HGS	No serious	0.20 [-0.03; 0.43]	Serious	*p = 0.13*	Undetected	⊕⊕⊖⊖
(b) Protein supplementation alone																				
Casein	1	76	5.09%	94.76%	0.15%	No Serious	0.09 [-1.00; 1.17]	0.10 [-1.59; 1.79]	0.09 [-0.82; 1.00]	No serious	Elder people	PS	RC, PLA, AC	HGS	No serious	0.09 [-0.82; 1.00]	Serious	*p = 0.13*	Undetected	⊕⊕⊕⊖
DP	4	162	26.91%	72.76%	0.33%	No Serious	0.12 [-0.49; 0.74]	-0.56 [-1.08; -0.04]	-0.27 [-0.67; 0.12]	No serious	Elder people	PS	RC, PLA, AC	HGS	No serious	-0.27 [-0.67; 0.12]	Serious	*p = 0.13*	Undetected	⊕⊕⊕⊖
Meat	1	17	1.59%	98.33%	0.08%	No Serious	-0.55 [-1.94; 0.84]	-0.17 [-2.45; 2.11]	-0.45 [-1.63; 0.74]	No serious	Elder people	PS	RC, PLA, AC	HGS	No serious	-0.45 [-1.63; 0.74]	Serious	*p = 0.13*	Undetected	⊕⊕⊕⊖
MP	2	236	4.28%	92.89%	2.83%	No Serious	0.12 [-0.63; 0.87]	-0.04 [-0.63; 0.56]	0.03 [-0.44; 0.49]	No serious	Elder people	PS	RC, PLA, AC	HGS	No serious	0.03 [-0.44; 0.49]	Serious	*p = 0.13*	Undetected	⊕⊕⊕⊖
SP	3	97	11.79%	87.17%	1.04%	No Serious	0.45 [-0.28; 1.18]	-0.56 [-1.72; 0.60]	0.16 [-0.46; 0.78]	No serious	Elder people	PS	RC, PLA, AC	HGS	No serious	0.16 [-0.46; 0.78]	Serious	*p = 0.13*	Undetected	⊕⊕⊕⊖
WP	12	397	22.07%	77.05%	0.88%	No Serious	0.35 [-0.01; 0.71]	0.12 [-0.27; 0.52]	0.25 [-0.02; 0.51]	No serious	Elder people	PS	RC, PLA, AC	HGS	No serious	0.25 [-0.02; 0.51]	Serious	*p = 0.13*	Undetected	⊕⊕⊕⊖

[a]Values present the number of studies which provided direct comparisons for each indicated treatment arm versus the regular control.

[b]Performed by Begg–Mazumdar rank correlation test

[c]Certainty of evidence is graded as follows: High: ⊕⊕⊕⊕; Moderate: ⊕⊕⊕⊖; Low: ⊕⊕⊖⊖; Very low: ⊕⊖⊖⊖.

AC, active comparater; AET, aerobic exercise training; 95% CI, 95% confidence interval; DP, dietary protein; GRADE, Grading of Recommendations, Assessment, Development and Evaluations; Ins-PS, insect protein supplement; HGS, handgrip strength; MET, multicomponent exercise training; MP, milk protein; PLA, placebo supplementation; PS, protein supplementation; RC, regular care; RET, resistance exercise training ; SMD, standardized mean difference; SP, soy protein; WP, whey protein.

Supplementary table S15. GRADE certainty rating of treatment efficacy for leg strength outcome.

Treatment (Common comparator: RC)	Involved studies for direct evidence (n)[a]	Participants (n)	Study limitation				Inconsistency (incoherence)				Indirection (transitivity)					Imprecision		Publication bias		Certainty of evidence[c]
			Contribution of risks of bias of direct estimates to network estimates			Judgment	Treatment effect, SMD (95%CI)			Judgment	PICO assessment				Judgment	Network estimate	Judgment	Statistical test[b]	Judgment	
			High	Mod	Low		Direct estimate	Indirect estimate	Network estimate		Participant (P)	Intervention (I)	Comparison (C)	Outcome (O)						
A. Combined therapy																				
(a) Protein supplementation plus RET																				
Casein+RET	0		27.37%	68.47%	4.16%	No Serious		1.10 [0.20; 2.00]	1.10 [0.20; 2.00]	No serious	Elder people	PS+RET	RC, PLA, AC	QdS	No serious	1.10 [0.20; 2.00]	No serious	*p < 0.01*	Strongly suspected	⊕⊕⊕⊖
Collagen+RET	1	19	36.80%	46.55%	16.65%	No Serious	0.90 [-0.57; 2.38]	1.26 [0.56; 1.97]	1.20 [0.56; 1.83]	No serious	Elder people	PS+RET	RC, PLA, AC	QdS	No serious	1.20 [0.56; 1.83]	No serious	*p < 0.01*	Strongly suspected	⊕⊕⊕⊖
DP+RET	4	327	5.30%	91.42%	3.28%	No Serious	0.76 [0.13; 1.39]	0.73 [-0.20; 1.66]	0.75 [0.23; 1.27]	No serious	Elder people	PS+RET	RC, PLA, AC	QdS	No serious	0.75 [0.23; 1.27]	No serious	*p < 0.01*	Strongly suspected	⊕⊕⊕⊖
Meat+RET	1	39	9.52%	84.03%	6.45%	No Serious	1.09 [-0.22; 2.40]	0.91 [0.33; 1.48]	0.94 [0.41; 1.46]	No serious	Elder people	PS+RET	RC, PLA, AC	QdS	No serious	0.94 [0.41; 1.46]	No serious	*p < 0.01*	Strongly suspected	⊕⊕⊕⊖
MP+RET	6	384	9.43%	78.87%	11.70%	No Serious	0.79 [0.27; 1.30]	1.00 [0.65; 1.36]	0.93 [0.64; 1.23]	No serious	Elder people	PS+RET	RC, PLA, AC	QdS	No serious	0.93 [0.64; 1.23]	No serious	*p < 0.01*	Strongly suspected	⊕⊕⊕⊖
Rice+RET	0		6.49%	80.38%	13.13%	No Serious		0.69 [-0.06; 1.44]	0.69 [-0.06; 1.44]	No serious	Elder people	PS+RET	RC, PLA, AC	QdS	No serious	0.69 [-0.06; 1.44]	Serious	*p < 0.01*	Strongly suspected	⊕⊕⊖⊖
SP+RET	7	336	10.91%	84.25%	4.84%	No Serious	1.53 [1.03; 2.03]	0.74 [0.27; 1.21]	1.11 [0.77; 1.45]	Serious	Elder people	PS+RET	RC, PLA, AC	QdS	No serious	1.11 [0.77; 1.45]	No serious	*p < 0.01*	Strongly suspected	⊕⊕⊖⊖
WP+RET	9	466	44.95%	49.58%	5.47%	No Serious	1.02 [0.57; 1.48]	1.23 [0.91; 1.54]	1.16 [0.90; 1.42]	No serious	Elder people	PS+RET	RC, PLA, AC	QdS	No serious	1.16 [0.90; 1.42]	No serious	*p < 0.01*	Strongly suspected	⊕⊕⊕⊖
(b) Protein supplementation plus AET																				
DP+AET	0		27.37%	68.47%	4.16%	No Serious		-0.31 [-1.25; 0.63]	-0.31 [-1.25; 0.63]	No serious	Elder people	PS+AET	RC, PLA, AC	QdS	No serious	-0.31 [-1.25; 0.63]	Serious	*p < 0.01*	Strongly suspected	⊕⊕⊖⊖
Insect-PS+AET	0		35.08%	39.78%	25.14%	No Serious		1.07 [-0.24; 2.39]	1.07 [-0.24; 2.39]	No serious	Elder people	PS+AET	RC, PLA, AC	QdS	No serious	1.07 [-0.24; 2.39]	Serious	*p < 0.01*	Strongly suspected	⊕⊕⊖⊖
MP+AET	0		12.04%	76.91%	11.05%	No Serious		0.58 [-0.13; 1.29]	0.58 [-0.13; 1.29]	No serious	Elder people	PS+AET	RC, PLA, AC	QdS	No serious	0.58 [-0.13; 1.29]	Serious	*p < 0.01*	Strongly suspected	⊕⊕⊖⊖
SP+AET	0		40.67%	38.23%	21.10%	No Serious		0.84 [-0.25; 1.92]	0.84 [-0.25; 1.92]	No serious	Elder people	PS+AET	RC, PLA, AC	QdS	No serious	0.84 [-0.25; 1.92]	Serious	*p < 0.01*	Strongly suspected	⊕⊕⊖⊖
WP+AET	1	46	16.77%	71.33%	11.90%	No Serious	1.45 [0.15; 2.75]	0.60 [-0.39; 1.60]	0.92 [0.13; 1.70]	No serious	Elder people	PS+AET	RC, PLA, AC	QdS	No serious	0.92 [0.13; 1.70]	No serious	*p < 0.01*	Strongly suspected	⊕⊕⊕⊖
(c) Protein supplementation plus MET																				
Casein+MET	2	97	21.05%	62.25%	16.70%	No Serious	0.90 [-0.05; 1.85]	0.40 [-0.32; 1.13]	0.59 [0.01; 1.17]	No serious	Elder people	PS+MET	RC, PLA, AC	QdS	No serious	0.59 [0.01; 1.17]		*p < 0.01*	Strongly suspected	⊕⊕⊕⊖
DP+MET	3	390	6.80%	82.07%	11.13%	No Serious	-0.33 [-1.03; 0.36]	0.54 [-0.23; 1.31]	0.06 [-0.45; 0.58]	No serious	Elder people	PS+MET	RC, PLA, AC	QdS	No serious	0.06 [-0.45; 0.58]	Serious	*p < 0.01*	Strongly suspected	⊕⊕⊖⊖
Meat+MET	1	17	18.43%	52.07%	29.50%	No Serious	-0.43 [-1.93; 1.06]	0.17 [-0.95; 1.28]	-0.05 [-0.94; 0.84]	No serious	Elder people	PS+MET	RC, PLA, AC	QdS	No serious	-0.05 [-0.94; 0.84]	Serious	*p < 0.01*	Strongly suspected	⊕⊕⊖⊖
MP+MET	5	333	5.94%	48.22%	45.84%	No Serious	0.78 [0.20; 1.35]	0.57 [0.05; 1.09]	0.66 [0.27; 1.05]	No serious	Elder people	PS+MET	RC, PLA, AC	QdS	No serious	0.66 [0.27; 1.05]	No serious	*p < 0.01*	Strongly suspected	⊕⊕⊕⊖
Oat+MET	0		17.20%	70.54%	12.26%	No Serious		0.80 [-0.64; 2.23]	0.80 [-0.64; 2.23]	No serious	Elder people	PS+MET	RC, PLA, AC	QdS	No serious					⊕⊕⊕⊖
SP+MET	3	132	4.60%	71.41%	23.99%	No Serious	0.60 [-0.15; 1.36]	0.44 [-1.10; 1.98]	0.57 [-0.11; 1.25]	No serious	Elder people	PS+MET	RC, PLA, AC	QdS	No serious	0.57 [-0.11; 1.25]	Serious	*p < 0.01*	Strongly suspected	⊕⊕⊖⊖
WP+MET	8	222	32.55%	49.60%	17.85%	No Serious	1.17 [0.67; 1.68]	1.05 [0.61; 1.50]	1.11 [0.77; 1.44]	No serious	Elder people	PS+MET	RC, PLA, AC	QdS	No serious	1.11 [0.77; 1.44]	No serious	*p < 0.01*	Strongly suspected	⊕⊕⊕⊖
B. Monotherapy																				
(a) Exercise training alone																				
RET	20	870	19.24%	72.49%	8.27%	No Serious	0.94 [0.65; 1.24]	0.57 [0.22; 0.92]	0.79 [0.56; 1.01]	No serious	Elder people	RET	RC, PLA, AC	QdS	No serious	0.79 [0.56; 1.01]	No serious	*p < 0.01*	Strongly suspected	⊕⊕⊕⊖
AET	1	46	25.48%	52.61%	21.91%	No Serious	0.24 [-1.03; 1.52]	0.58 [-0.15; 1.31]	0.50 [-0.13; 1.13]	No serious	Elder people	AET	RC, PLA, AC	QdS	No serious	0.50 [-0.13; 1.13]	Serious	*p < 0.01*	Strongly suspected	⊕⊕⊖⊖
MET	14	703	9.77%	46.66%	43.57%	No Serious	0.54 [0.19; 0.89]	0.51 [0.02; 0.99]	0.53 [0.24; 0.81]	No serious	Elder people	MET	RC, PLA, AC	QdS	No serious	0.53 [0.24; 0.81]	No serious	*p < 0.01*	Strongly suspected	⊕⊕⊕⊖
(b) Protein supplementation alone																				
Casein	1	76	7.71%	81.52%	10.77%	No Serious	0.14 [-1.09; 1.36]	0.57 [-0.59; 1.73]	0.37 [-0.47; 1.21]	No serious	Elder people	PS	RC, PLA, AC	QdS	No serious	0.37 [-0.47; 1.21]	Serious	*p < 0.01*	Strongly suspected	⊕⊕⊖⊖
Collagen	1	78	13.27%	83.56%	3.17%	No Serious	-0.39 [-1.62; 0.83]	0.64 [-1.11; 2.39]	-0.05 [-1.06; 0.95]	No serious	Elder people	PS	RC, PLA, AC	QdS	No serious	-0.05 [-1.06; 0.95]	Serious	*p < 0.01*	Strongly suspected	⊕⊕⊖⊖
DP	2	128	11.62%	76.87%	11.51%	No Serious	-0.21 [-1.09; 0.67]	-0.27 [-1.32; 0.78]	-0.24 [-0.91; 0.44]	No serious	Elder people	PS	RC, PLA, AC	QdS	No serious	-0.24 [-0.91; 0.44]	Serious	*p < 0.01*	Strongly suspected	⊕⊕⊖⊖
Meat	2	60	12.98%	51.16%	35.86%	No Serious	-0.23 [-1.20; 0.74]	-0.17 [-1.22; 0.89]	-0.20 [-0.92; 0.52]	No serious	Elder people	PS	RC, PLA, AC	QdS	No serious	-0.20 [-0.92; 0.52]	Serious	*p < 0.01*	Strongly suspected	⊕⊕⊖⊖
MP	9	672	4.63%	47.30%	48.07%	No Serious	-0.02 [-0.43; 0.39]	0.37 [-0.24; 0.98]	0.10 [-0.24; 0.44]	No serious	Elder people	PS	RC, PLA, AC	QdS	No serious	0.10 [-0.24; 0.44]	Serious	*p < 0.01*	Strongly suspected	⊕⊕⊖⊖
SP	6	250	39.81%	44.58%	15.61%	No Serious	0.40 [-0.14; 0.93]	-0.30 [-1.00; 0.39]	0.14 [-0.28; 0.56]	No serious	Elder people	PS	RC, PLA, AC	QdS	No serious	0.14 [-0.28; 0.56]	Serious	*p < 0.01*	Strongly suspected	⊕⊕⊖⊖
WP	11	359	12.97%	80.33%	6.70%	No Serious	-0.02 [-0.43; 0.39]	-0.09 [-0.54; 0.36]	-0.05 [-0.36; 0.25]	No serious	Elder people	PS	RC, PLA, AC	QdS	No serious	-0.05 [-0.36; 0.25]	Serious	*p < 0.01*	Strongly suspected	⊕⊕⊖⊖

[a]Values present the number of studies which provided direct comparisons for each indicated treatment arm versus the regular control.
[b]Performed by Begg–Mazumdar rank correlation test
[c]Certainty of evidence is graded as follows: High: ⊕⊕⊕⊕; Moderate: ⊕⊕⊕⊖; Low: ⊕⊕⊖⊖; Very low: ⊕⊖⊖⊖.
AC, active comparater; AET, aerobic exercise training; 95% CI, 95% confidence interval; DP, dietary protein; GRADE, Grading of Recommendations, Assessment, Development and Evaluations; Ins-PS, insect protein supplement; QdS, quadriceps strength; MET, multicomponent exercise training; MP, milk protein; PLA, placebo supplementation; PS, protein supplementation; RC, regular care; RET, resistance exercise training ; SMD, standardized mean difference; SP, soy protein; WP, whey protein.

Supplementary table S16. GRADE certainty rating of treatment efficacy for walking speed outcome.

Treatment (Common comparator: RC)	Involved studies for direct evidence (n)[a]	Participants (n)	Study limitation				Inconsistency (incoherence)				Indirection (transitivity)					Imprecision		Publication bias		Certainty of evidence[c]
			Contribution of risks of bias of direct estimates to network estimates			Judgment	Treatment effect, SMD (95%CI)			Judgment	PICO assessment				Judgment	Network estimate	Judgment	Statistical test[b]	Judgment	
			High	Mod	Low		Direct estimate	Indirect estimate	Network estimate		Participant (P)	Intervention (I)	Comparison (C)	Outcome (O)						
A. Combined therapy																				
(a) Protein supplementation plus RET																				
Casein+RET	0		8.27%	89.41%	2.32%	No Serious		1.18 [0.04; 2.32]	1.18 [0.04; 2.32]	No serious	Elder people	PS+RET	RC, PLA, AC	GS	No serious	1.18 [0.04; 2.32]	No serious	*p = 0.35*	Undetected	⊕⊕⊕⊕
Collagen+RET	0		8.27%	89.41%	2.32%	No Serious		1.19 [-0.08; 2.46]	1.19 [-0.08; 2.46]	No serious	Elder people	PS+RET	RC, PLA, AC	GS	No serious	1.19 [-0.08; 2.46]	Serious	*p = 0.35*	Undetected	⊕⊕⊕⊖
DP+RET	7	678	24.19%	75.41%	0.40%	No Serious	0.37 [-0.05; 0.80]	0.93 [0.39; 1.48]	0.59 [0.25; 0.92]	No serious	Elder people	PS+RET	RC, PLA, AC	GS	No serious	0.59 [0.25; 0.92]	No serious	*p = 0.35*	Undetected	⊕⊕⊕⊕
Meat+RET	0		9.77%	89.10%	1.13%	No Serious		0.64 [-0.56; 1.84]	0.64 [-0.56; 1.84]	No serious	Elder people	PS+RET	RC, PLA, AC	GS	No serious	0.64 [-0.56; 1.84]	Serious	*p = 0.35*	Undetected	⊕⊕⊕⊖
MP+RET	5	310	39.02%	55.96%	5.02%	No Serious	0.35 [-0.18; 0.87]	0.91 [0.52; 1.30]	0.71 [0.40; 1.02]	No serious	Elder people	PS+RET	RC, PLA, AC	GS	No serious	0.71 [0.40; 1.02]	No serious	*p = 0.35*	Undetected	⊕⊕⊕⊕
Rice+RET	0		24.10%	73.57%	2.33%	No Serious		-0.22 [-1.49; 1.05]	-0.22 [-1.49; 1.05]	No serious	Elder people	PS+RET	RC, PLA, AC	GS	No serious	-0.22 [-1.49; 1.05]	Serious	*p = 0.35*	Undetected	⊕⊕⊕⊖
SP+RET	4	244	43.83%	55.14%	1.03%	No Serious	1.23 [0.64; 1.81]	0.78 [0.09; 1.48]	1.04 [0.59; 1.49]	No serious	Elder people	PS+RET	RC, PLA, AC	GS	No serious	1.04 [0.59; 1.49]	No serious	*p = 0.35*	Undetected	⊕⊕⊕⊕
WP+RET	16	797	11.73%	84.90%	3.37%	No Serious	1.08 [0.76; 1.40]	0.94 [0.57; 1.30]	1.02 [0.78; 1.26]	No serious	Elder people	PS+RET	RC, PLA, AC	GS	No serious	1.02 [0.78; 1.26]	No serious	*p = 0.35*	Undetected	⊕⊕⊕⊕
(b) Protein supplementation plus AET																				
Casein+AET	0		35.80%	63.47%	0.73%	No Serious		-0.41 [-1.58; 0.76]	-0.41 [-1.58; 0.76]	No serious	Elder people	PS+AET	RC, PLA, AC	GS	No serious	-0.41 [-1.58; 0.76]	Serious	*p = 0.35*	Undetected	⊕⊕⊕⊖
DP+AET	2	79	50.49%	48.41%	1.10%	Serious	0.92 [0.04; 1.79]	-0.05 [-0.73; 0.64]	0.32 [-0.22; 0.86]	No serious	Elder people	PS+AET	RC, PLA, AC	GS	No serious	0.32 [-0.22; 0.86]	Serious	*p = 0.35*	Undetected	⊕⊕⊖⊖
MP+AET	0		16.23%	81.69%	2.08%	No Serious		0.88 [-0.10; 1.86]	0.88 [-0.10; 1.86]	No serious	Elder people	PS+AET	RC, PLA, AC	GS	No serious	0.88 [-0.10; 1.86]	Serious	*p = 0.35*	Undetected	⊕⊕⊕⊖
SP+AET	0		11.71%	86.59%	1.70%	No Serious		0.57 [-0.93; 2.07]	0.57 [-0.93; 2.07]	No serious	Elder people	PS+AET	RC, PLA, AC	GS	No serious	0.57 [-0.93; 2.07]	Serious	*p = 0.35*	Undetected	⊕⊕⊕⊖
(c) Protein supplementation plus MET																				
Casein+MET	1	79	16.46%	82.47%	1.07%	No Serious	0.38 [-0.75; 1.51]	0.83 [0.19; 1.47]	0.67 [0.11; 1.24]	No serious	Elder people	PS+MET	RC, PLA, AC	GS	No serious	0.67 [0.11; 1.24]	No serious	*p = 0.35*	Undetected	⊕⊕⊕⊕
Collagen+MET	0		52.01%	46.61%	1.38%	Serious		0.71 [-1.02; 2.44]	0.71 [-1.02; 2.44]	No serious	Elder people	PS+MET	RC, PLA, AC	GS	No serious	0.71 [-1.02; 2.44]	Serious	*p = 0.35*	Undetected	⊕⊕⊖⊖
DP+MET	12	1111	68.45%	30.86%	0.69%	Serious	0.57 [0.23; 0.90]	0.35 [-0.10; 0.80]	0.52 [0.24; 0.79]	No serious	Elder people	PS+MET	RC, PLA, AC	GS	No serious	0.52 [0.24; 0.79]	No serious	*p = 0.35*	Undetected	⊕⊕⊕⊖
Meat+MET	1	17	43.97%	55.50%	0.53%	No Serious	0.02 [-1.40; 1.44]	0.83 [-0.19; 1.84]	0.57 [-0.26; 1.39]	No serious	Elder people	PS+MET	RC, PLA, AC	GS	No serious	0.57 [-0.26; 1.39]	Serious	*p = 0.35*	Undetected	⊕⊕⊕⊖
MP+MET	4	448	15.40%	69.31%	15.29%	No Serious	0.25 [-0.30; 0.81]	0.97 [0.51; 1.43]	0.67 [0.32; 1.03]	No serious	Elder people	PS+MET	RC, PLA, AC	GS	No serious	0.67 [0.32; 1.03]	No serious	*p = 0.35*	Undetected	⊕⊕⊕⊕
Rice+MET	0		11.36%	83.69%	4.95%	No Serious		0.59 [-0.24; 1.42]	0.59 [-0.24; 1.42]	No serious	Elder people	PS+MET	RC, PLA, AC	GS	No serious	0.59 [-0.24; 1.42]	Serious	*p = 0.35*	Undetected	⊕⊕⊕⊖
SP+MET	4	212	12.76%	86.73%	0.51%	No Serious	0.20 [-0.42; 0.82]	0.57 [-0.38; 1.52]	0.31 [-0.21; 0.82]	No serious	Elder people	PS+MET	RC, PLA, AC	GS	No serious	0.31 [-0.21; 0.82]	Serious	*p = 0.35*	Undetected	⊕⊕⊕⊖
WP+MET	8	399	15.02%	79.02%	5.96%	No Serious	1.03 [0.59; 1.47]	0.61 [0.25; 0.97]	0.77 [0.49; 1.05]	No serious	Elder people	PS+MET	RC, PLA, AC	GS	No serious	0.77 [0.49; 1.05]	No serious	*p = 0.35*	Undetected	⊕⊕⊕⊕
B. Monotherapy																				
(a) Exercise training alone																				
RET	17	732	14.03%	84.60%	1.37%	No Serious	0.66 [0.36; 0.96]	0.79 [0.43; 1.15]	0.72 [0.48; 0.95]	No serious	Elder people	RET	RC, PLA, AC	GS	No serious	0.72 [0.48; 0.95]	No serious	*p = 0.35*	Undetected	⊕⊕⊕⊕
AET	0		20.82%	77.29%	1.89%	No Serious		0.90 [-0.07; 1.88]	0.90 [-0.07; 1.88]	No serious	Elder people	AET	RC, PLA, AC	GS	No serious	0.90 [-0.07; 1.88]	Serious	*p = 0.35*	Undetected	⊕⊕⊕⊖
MET	15	1069	16.32%	81.59%	2.09%	No Serious	0.26 [-0.04; 0.56]	0.52 [0.12; 0.92]	0.34 [0.10; 0.58]	No serious	Elder people	MET	RC, PLA, AC	GS	No serious	0.34 [0.10; 0.58]	No serious	*p = 0.35*	Undetected	⊕⊕⊕⊕
(b) Protein supplementation alone																				
Casein	1	76	25.98%	73.31%	0.71%	No Serious	0.29 [-0.85; 1.42]	0.47 [-0.59; 1.53]	0.37 [-0.40; 1.15]	No serious	Elder people	PS	RC, PLA, AC	GS	No serious	0.37 [-0.40; 1.15]	Serious	*p = 0.35*	Undetected	⊕⊕⊕⊖
Collagen	1	78	4.59%	34.20%	61.21%	No Serious	0.04 [-1.10; 1.17]	1.30 [-0.32; 2.93]	0.45 [-0.48; 1.38]	No serious	Elder people	PS	RC, PLA, AC	GS	No serious	0.45 [-0.48; 1.38]	Serious	*p = 0.35*	Undetected	⊕⊕⊕⊖
DP	7	401	39.32%	60.32%	0.36%	No Serious	0.21 [-0.24; 0.67]	-0.01 [-0.50; 0.47]	0.11 [-0.22; 0.44]	No serious	Elder people	PS	RC, PLA, AC	GS	No serious	0.11 [-0.22; 0.44]	Serious	*p = 0.35*	Undetected	⊕⊕⊕⊖
Meat	1	17	14.55%	84.79%	0.66%	No Serious	-0.08 [-1.50; 1.34]	0.82 [-1.23; 2.87]	0.21 [-0.96; 1.37]	No serious	Elder people	PS	RC, PLA, AC	GS	No serious	0.21 [-0.96; 1.37]	Serious	*p = 0.35*	Undetected	⊕⊕⊕⊖
MP	5	479	17.45%	71.80%	10.75%	No Serious	0.33 [-0.17; 0.83]	0.44 [-0.12; 0.99]	0.37 [0.00; 0.75]	No serious	Elder people	PS	RC, PLA, AC	GS	No serious	0.37 [0.00; 0.75]	No serious	*p = 0.35*	Undetected	⊕⊕⊕⊕
SP	5	194	56.80%	42.94%	0.26%	Serious	0.54 [-0.03; 1.10]	-0.14 [-1.08; 0.80]	0.36 [-0.13; 0.84]	No serious	Elder people	PS	RC, PLA, AC	GS	No serious	0.36 [-0.13; 0.84]	Serious	*p = 0.35*	Undetected	⊕⊕⊖⊖
WP	10	329	6.91%	87.88%	5.21%	No Serious	0.54 [0.14; 0.95]	0.63 [0.16; 1.10]	0.58 [0.27; 0.89]	No serious	Elder people	PS	RC, PLA, AC	GS	No serious	0.58 [0.27; 0.89]	No serious	*p = 0.35*	Undetected	⊕⊕⊕⊕

[a]Values present the number of studies which provided direct comparisons for each indicated treatment arm versus the regular control.
[b]Performed by Begg–Mazumdar rank correlation test
[c]Certainty of evidence is graded as follows: High: ⊕⊕⊕⊕; Moderate: ⊕⊕⊕⊖; Low: ⊕⊕⊖⊖; Very low: ⊕⊖⊖⊖.
AC, active comparater; AET, aerobic exercise training; 95% CI, 95% confidence interval; DP, dietary protein; GRADE, Grading of Recommendations, Assessment, Development and Evaluations; GS, gait speed; MET, multicomponent exercise training; MP, milk protein; PLA, placebo supplementation; PS, protein supplementation; RC, regular care; RET, resistance exercise training ; SMD, standardized mean difference; SP, soy protein; WP, whey protein.

Supplementary table S17. GRADE certainty rating of treatment efficacy for chair rise outcome.

Treatment (Common comparator: RC)	Involved studies for direct evidence (n)[a]	Participants (n)	Study limitation: Contribution of risks of bias of direct estimates to network estimates			Study limitation	Inconsistency (incoherence): Treatment effect, SMD (95%CI)			Inconsistency (incoherence)	Indirection (transitivity): PICO assessment				Indirection (transitivity)	Imprecision		Publication bias		Certainty of evidence[c]
			High	Mod	Low	Judgment	Direct estimate	Indirect estimate	Network estimate	Judgment	Participant (P)	Intervention (I)	Comparison (C)	Outcome (O)	Judgment	Network estimate	Judgment	Statistical test[b]	Judgment	
A. Combined therapy																				
(a) Protein supplementation plus RET																				
Casein+RET	0		2.08%	94.62%	3.30%	No Serious		0.83 [-0.24; 1.90]	0.83 [-0.24; 1.90]	No serious	Elder people	PS+RET	RC, PLA, AC	STS	No serious	0.83 [-0.24; 1.90]	Serious	*p = 0.31*	Undetected	⊕⊕⊕⊖
Collagen+RET	0		2.08%	94.62%	3.30%	No Serious		0.70 [-0.50; 1.91]	0.70 [-0.50; 1.91]	No serious	Elder people	PS+RET	RC, PLA, AC	STS	No serious	0.70 [-0.50; 1.91]	Serious	*p = 0.31*	Undetected	⊕⊕⊕⊖
DP+RET	7	539	9.11%	90.83%	0.06%	No Serious	0.43 [0.03; 0.83]	0.64 [-0.43; 1.72]	0.45 [0.08; 0.83]	No serious	Elder people	PS+RET	RC, PLA, AC	STS	No serious	0.45 [0.08; 0.83]	No serious	*p = 0.31*	Undetected	⊕⊕⊕⊕
Meat+RET	0		3.31%	92.19%	4.50%	No Serious		0.11 [-1.48; 1.70]	0.11 [-1.48; 1.70]	No serious	Elder people	PS+RET	RC, PLA, AC	STS	No serious	0.11 [-1.48; 1.70]	Serious	*p = 0.31*	Undetected	⊕⊕⊕⊖
MP+RET	2	106	8.00%	84.86%	7.14%	No Serious	0.51 [-0.29; 1.32]	0.62 [0.23; 1.01]	0.60 [0.25; 0.95]	No serious	Elder people	PS+RET	RC, PLA, AC	STS	No serious	0.60 [0.25; 0.95]	No serious	*p = 0.31*	Undetected	⊕⊕⊕⊕
Rice+RET	0		4.21%	89.87%	5.92%	No Serious		0.46 [-0.74; 1.67]	0.46 [-0.74; 1.67]	No serious	Elder people	PS+RET	RC, PLA, AC	STS	No serious	0.46 [-0.74; 1.67]	Serious	*p = 0.31*	Undetected	⊕⊕⊕⊖
SP+RET	1	80	2.85%	87.23%	9.92%	No Serious	0.30 [-0.74; 1.34]	0.75 [-0.04; 1.54]	0.58 [-0.05; 1.21]	No serious	Elder people	PS+RET	RC, PLA, AC	STS	No serious	0.58 [-0.05; 1.21]	Serious	*p = 0.31*	Undetected	⊕⊕⊕⊖
WP+RET	9	512	2.57%	92.62%	4.81%	No Serious	0.82 [0.44; 1.20]	0.45 [-0.02; 0.93]	0.67 [0.38; 0.97]	No serious	Elder people	PS+RET	RC, PLA, AC	STS	No serious	0.67 [0.38; 0.97]	No serious	*p = 0.31*	Undetected	⊕⊕⊕⊕
(b) Protein supplementation plus AET																				
MP+AET	0		0.00%	44.39%	55.61%	No Serious		0.84 [-0.66; 2.35]	0.84 [-0.66; 2.35]	No serious	Elder people	PS+AET	RC, PLA, AC	STS	No serious	0.84 [-0.66; 2.35]	Serious	*p = 0.31*	Undetected	⊕⊕⊕⊖
WP+AET	1	46	0.00%	0.00%	100.00%	No Serious	0.81 [-0.31; 1.92]		0.81 [-0.31; 1.92]	No serious	Elder people	PS+AET	RC, PLA, AC	STS	No serious	0.81 [-0.31; 1.92]	Serious	*p = 0.31*	Undetected	⊕⊕⊕⊖
(c) Protein supplementation plus MET																				
Casein+MET	1	79	16.63%	80.64%	2.73%	No Serious	0.39 [-0.65; 1.43]	0.57 [-0.39; 1.54]	0.49 [-0.22; 1.20]	No serious	Elder people	PS+MET	RC, PLA, AC	STS	No serious	0.49 [-0.22; 1.20]	Serious	*p = 0.31*	Undetected	⊕⊕⊕⊖
DP+MET	5	491	73.25%	25.54%	1.21%	Serious	0.84 [0.37; 1.30]	0.33 [-0.51; 1.16]	0.71 [0.31; 1.12]	No serious	Elder people	PS+MET	RC, PLA, AC	STS	No serious	0.71 [0.31; 1.12]	No serious	*p = 0.31*	Undetected	⊕⊕⊕⊖
Meat+MET	0		81.02%	18.01%	0.97%	Serious		0.71 [-0.36; 1.79]	0.71 [-0.36; 1.79]	No serious	Elder people	PS+MET	RC, PLA, AC	STS	No serious	0.71 [-0.36; 1.79]	Serious	*p = 0.31*	Undetected	⊕⊕⊖⊖
MP+MET	3	125	15.57%	75.83%	8.60%	No Serious	0.62 [-0.05; 1.30]	0.91 [0.15; 1.67]	0.75 [0.24; 1.25]	No serious	Elder people	PS+MET	RC, PLA, AC	STS	No serious	0.75 [0.24; 1.25]	No serious	*p = 0.31*	Undetected	⊕⊕⊕⊕
Rice+MET	0		11.23%	82.47%	6.30%	No Serious		0.55 [-0.55; 1.65]	0.55 [-0.55; 1.65]	No serious	Elder people	PS+MET	RC, PLA, AC	STS	No serious	0.55 [-0.55; 1.65]	Serious	*p = 0.31*	Undetected	⊕⊕⊕⊖
SP+MET	3	198	5.99%	92.92%	1.09%	No Serious	0.33 [-0.30; 0.97]	0.52 [-0.56; 1.60]	0.38 [-0.16; 0.93]	No serious	Elder people	PS+MET	RC, PLA, AC	STS	No serious	0.38 [-0.16; 0.93]	Serious	*p = 0.31*	Undetected	⊕⊕⊕⊖
WP+MET	2	90	12.90%	77.47%	9.63%	No Serious	0.56 [-0.24; 1.35]	0.90 [0.38; 1.43]	0.80 [0.36; 1.24]	No serious	Elder people	PS+MET	RC, PLA, AC	STS	No serious	0.80 [0.36; 1.24]	No serious	*p = 0.31*	Undetected	⊕⊕⊕⊕
B. Monotherapy																				
(a) Exercise training alone																				
RET	9	361	4.33%	94.97%	0.70%	No Serious	0.77 [0.38; 1.17]	0.19 [-0.24; 0.61]	0.50 [0.21; 0.79]	No serious	Elder people	RET	RC, PLA, AC	STS	No serious	0.50 [0.21; 0.79]	No serious	*p = 0.31*	Undetected	⊕⊕⊕⊕
AET	1	46	0.00%	0.00%	100.00%	No Serious	0.79 [-0.32; 1.91]		0.79 [-0.32; 1.91]	No serious	Elder people	AET	RC, PLA, AC	STS	No serious	0.79 [-0.32; 1.91]	Serious	*p = 0.31*	Undetected	⊕⊕⊕⊖
MET	8	487	19.61%	76.92%	3.47%	No Serious	0.39 [0.00; 0.78]	-0.11 [-0.74; 0.53]	0.26 [-0.07; 0.59]	No serious	Elder people	MET	RC, PLA, AC	STS	No serious	0.26 [-0.07; 0.59]	Serious	*p = 0.31*	Undetected	⊕⊕⊕⊖
(b) Protein supplementation alone																				
Casein	1	76	9.49%	89.14%	1.37%	No Serious	0.14 [-0.90; 1.18]	0.28 [-1.49; 2.04]	0.18 [-0.72; 1.08]	No serious	Elder people	PS	RC, PLA, AC	STS	No serious	0.18 [-0.72; 1.08]	Serious	*p = 0.31*	Undetected	⊕⊕⊕⊖
DP	4	272	58.21%	41.42%	0.37%	Serious	0.05 [-0.48; 0.58]	0.29 [-0.51; 1.09]	0.12 [-0.32; 0.57]	No serious	Elder people	PS	RC, PLA, AC	STS	No serious	0.12 [-0.32; 0.57]	Serious	*p = 0.31*	Undetected	⊕⊕⊖⊖
MP	1	77	24.39%	73.81%	1.80%	No Serious	-0.27 [-1.32; 0.77]	0.24 [-0.82; 1.31]	-0.02 [-0.77; 0.73]	No serious	Elder people	PS	RC, PLA, AC	STS	No serious	-0.02 [-0.77; 0.73]	Serious	*p = 0.31*	Undetected	⊕⊕⊕⊖
SP	1	26	6.14%	92.85%	1.01%	No Serious	-0.41 [-1.63; 0.81]	0.49 [-1.37; 2.36]	-0.14 [-1.16; 0.88]	No serious	Elder people	PS	RC, PLA, AC	STS	No serious	-0.14 [-1.16; 0.88]	Serious	*p = 0.31*	Undetected	⊕⊕⊕⊖
WP	7	193	4.61%	89.90%	5.49%	No Serious	-0.04 [-0.51; 0.42]	0.22 [-0.55; 0.99]	0.03 [-0.37; 0.42]	No serious	Elder people	PS	RC, PLA, AC	STS	No serious	0.03 [-0.37; 0.42]	Serious	*p = 0.31*	Undetected	⊕⊕⊕⊖

[a]Values present the number of studies which provided direct comparisons for each indicated treatment arm versus the regular control.
[b]Performed by Begg–Mazumdar rank correlation test
[c]Certainty of evidence is graded as follows: High: ⊕⊕⊕⊕; Moderate: ⊕⊕⊕⊖; Low: ⊕⊕⊖⊖; Very low: ⊕⊖⊖⊖.
AC, active comparater; AET, aerobic exercise training; 95% CI, 95% confidence interval; DP, dietary protein; GRADE, Grading of Recommendations, Assessment, Development and Evaluations; MET, multicomponent exercise training; MP, milk protein; PLA, placebo supplementation; PS, protein supplementation; RC, regular care; RET, resistance exercise training ; SMD, standardized mean difference; SP, soy protein; STS, sit to stand; WP, whey protein.

Supplementary table S18. GRADE certainty rating of treatment efficacy for timed up-and-go outcome.

Treatment (Common comparator: RC)	Involved studies for direct evidence (n)[a]	Participants (n)	Study limitation				Inconsistency (incoherence)				Indirection (transitivity)					Imprecision		Publication bias		Certainty of evidence[c]
			Contribution of risks of bias of direct estimates to network estimates			Judgment	Treatment effect, SMD (95%CI)			Judgment	PICO assessment				Judgment	Network estimate	Judgment	Statistical test[b]	Judgment	
			High	Mod	Low		Direct estimate	Indirect estimate	Network estimate		Participant (P)	Intervention (I)	Comparison (C)	Outcome (O)						
A. Combined therapy																				
(a) Protein supplementation plus RET																				
DP+RET	6	563	13.69%	86.17%	0.14%	No Serious	0.27 [-0.08; 0.61]	0.57 [-0.12; 1.27]	0.33 [0.02; 0.64]	No serious	Elder people	PS+RET	RC, PLA, AC	TUG	No serious	0.33 [0.02; 0.64]	No serious	*p* = 0.07	Undetected	⊕⊕⊕⊕
Meat+RET	0		4.48%	88.06%	7.46%	No Serious		-0.10 [-1.11; 0.92]	-0.10 [-1.11; 0.92]	No serious	Elder people	PS+RET	RC, PLA, AC	TUG	No serious	-0.10 [-1.11; 0.92]	Serious	*p* = 0.07	Undetected	⊕⊕⊕⊝
MP+RET	1	65	29.94%	67.69%	2.37%	No Serious	0.33 [-0.55; 1.20]	-0.03 [-0.55; 0.50]	0.07 [-0.38; 0.52]	No serious	Elder people	PS+RET	RC, PLA, AC	TUG	No serious	0.07 [-0.38; 0.52]	Serious	*p* = 0.07	Undetected	⊕⊕⊕⊝
Rice+RET	0		6.17%	84.05%	9.78%	No Serious		0.00 [-0.57; 0.57]	0.00 [-0.57; 0.57]	No serious	Elder people	PS+RET	RC, PLA, AC	TUG	No serious	0.00 [-0.57; 0.57]	Serious	*p* = 0.07	Undetected	⊕⊕⊕⊝
SP+RET	1	80	6.81%	82.75%	10.44%	No Serious	-0.03 [-0.88; 0.81]	0.43 [-0.26; 1.13]	0.25 [-0.29; 0.78]	No serious	Elder people	PS+RET	RC, PLA, AC	TUG	No serious	0.25 [-0.29; 0.78]	Serious	*p* = 0.07	Undetected	⊕⊕⊕⊝
WP+RET	5	375	4.80%	92.00%	3.20%	No Serious	0.19 [-0.21; 0.60]	0.41 [-0.09; 0.90]	0.28 [-0.03; 0.59]	No serious	Elder people	PS+RET	RC, PLA, AC	TUG	No serious	0.28 [-0.03; 0.59]	Serious	*p* = 0.07	Undetected	⊕⊕⊕⊝
(b) Protein supplementation plus AET																				
MP+AET	0		12.43%	86.28%	1.29%	No Serious		-0.11 [-0.92; 0.71]	-0.11 [-0.92; 0.71]	No serious	Elder people	PS+AET	RC, PLA, AC	TUG	No serious	-0.11 [-0.92; 0.71]	Serious	*p* = 0.07	Undetected	⊕⊕⊕⊝
(c) Protein supplementation plus MET																				
Casein+MET	0		5.06%	86.75%	8.19%	No Serious		0.46 [-0.43; 1.35]	0.46 [-0.43; 1.35]	No serious	Elder people	PS+MET	RC, PLA, AC	TUG	No serious	0.46 [-0.43; 1.35]	Serious	*p* = 0.07	Undetected	⊕⊕⊕⊝
DP+MET	4	477	67.24%	31.49%	1.27%	**Serious**	0.10 [-0.31; 0.51]	0.72 [0.04; 1.40]	0.27 [-0.09; 0.62]	No serious	Elder people	PS+MET	RC, PLA, AC	TUG	No serious	0.27 [-0.09; 0.62]	Serious	*p* = 0.07	Undetected	⊕⊕⊝⊝
Meat+MET	0		76.78%	22.23%	0.99%	**Serious**		0.37 [-0.50; 1.23]	0.37 [-0.50; 1.23]	No serious	Elder people	PS+MET	RC, PLA, AC	TUG	No serious	0.37 [-0.50; 1.23]	Serious	*p* = 0.07	Undetected	⊕⊕⊝⊝
MP+MET	2	96	4.41%	66.06%	29.53%	No Serious	0.81 [0.14; 1.48]	0.15 [-0.37; 0.68]	0.40 [-0.01; 0.82]	No serious	Elder people	PS+MET	RC, PLA, AC	TUG	No serious	0.40 [-0.01; 0.82]	Serious	*p* = 0.07	Undetected	⊕⊕⊕⊝
SP+MET	1	102	0.00%	100.00%	0.00%	No Serious	0.04 [-0.78; 0.86]		0.04 [-0.78; 0.86]	No serious	Elder people	PS+MET	RC, PLA, AC	TUG	No serious	0.04 [-0.78; 0.86]	Serious	*p* = 0.07	Undetected	⊕⊕⊕⊝
WP+MET	3	101	4.43%	83.53%	12.04%	No Serious	0.07 [-0.55; 0.69]	0.78 [0.29; 1.27]	0.51 [0.12; 0.89]	No serious	Elder people	PS+MET	RC, PLA, AC	TUG	No serious	0.51 [0.12; 0.89]	No serious	*p* = 0.07	Undetected	⊕⊕⊕⊕
B. Monotherapy																				
(a) Exercise training alone																				
RET	3	152	11.00%	87.69%	1.31%	No Serious	0.09 [-0.44; 0.63]	0.15 [-0.26; 0.56]	0.13 [-0.19; 0.46]	No serious	Elder people	RET	RC, PLA, AC	TUG	No serious	0.13 [-0.19; 0.46]	Serious	*p* = 0.07	Undetected	⊕⊕⊕⊝
AET	0		12.43%	86.28%	1.29%	No Serious		-0.05 [-0.87; 0.76]	-0.05 [-0.87; 0.76]	No serious	Elder people	AET	RC, PLA, AC	TUG	No serious	-0.05 [-0.87; 0.76]	Serious	*p* = 0.07	Undetected	⊕⊕⊕⊝
MET	5	328	8.86%	79.85%	11.29%	No Serious	0.51 [0.10; 0.91]	-0.46 [-1.04; 0.12]	0.19 [-0.14; 0.52]	**Serious**	Elder people	MET	RC, PLA, AC	TUG	No serious	0.19 [-0.14; 0.52]	Serious	*p* = 0.07	Undetected	⊕⊕⊝⊝
(b) Protein supplementation alone																				
DP	2	128	34.11%	64.55%	1.34%	No Serious	0.19 [-0.44; 0.81]	-0.73 [-1.27; -0.18]	-0.33 [-0.74; 0.08]	**Serious**	Elder people	PS	RC, PLA, AC	TUG	No serious	-0.33 [-0.74; 0.08]	Serious	*p* = 0.07	Undetected	⊕⊕⊝⊝
MP	2	142	8.14%	78.21%	13.65%	No Serious	0.17 [-0.44; 0.78]	-0.87 [-1.74; 0.01]	-0.17 [-0.67; 0.33]	No serious	Elder people	PS	RC, PLA, AC	TUG	No serious	-0.17 [-0.67; 0.33]	Serious	*p* = 0.07	Undetected	⊕⊕⊕⊝
WP	2	72	3.97%	79.93%	16.10%	No Serious	0.06 [-0.63; 0.76]	-0.17 [-1.24; 0.89]	-0.01 [-0.59; 0.57]	No serious	Elder people	PS	RC, PLA, AC	TUG	No serious	-0.01 [-0.59; 0.57]	Serious	*p* = 0.07	Undetected	⊕⊕⊕⊝

[a]Values present the number of studies which provided direct comparisons for each indicated treatment arm versus the regular control.
[b]Performed by Begg–Mazumdar rank correlation test
[c]Certainty of evidence is graded as follows: High: ⊕⊕⊕⊕; Moderate: ⊕⊕⊕⊝; Low: ⊕⊕⊝⊝; Very low: ⊕⊝⊝⊝.
AC, active comparater; AET, aerobic exercise training; 95% CI, 95% confidence interval; DP, dietary protein; GRADE, Grading of Recommendations, Assessment, Development and Evaluations; TUG, timed up and go; MET, multicomponent exercise training; MP, milk protein; PLA, placebo supplementation; PS, protein supplementation; RC, regular care; RET, resistance exercise training ; SMD, standardized mean difference; SP, soy protein; WP, whey protein.

Supplementary table S19. GRADE certainty rating of treatment efficacy for global mobility (SPPB) outcome.

Treatment (Common comparator: RC)	Involved studies for direct evidence (n)[a]	Participants (n)	Study limitation				Inconsistency (incoherence)				Indirection (transitivity)					Imprecision		Publication bias		Certainty of evidence[c]
			Contribution of risks of bias of direct estimates to network estimates			Judgment	Treatment effect, SMD (95%CI)			Judgment	PICO assessment				Judgment	Network estimate	Judgment	Statistical test[b]	Judgment	
			High	Mod	Low		Direct estimate	Indirect estimate	Network estimate		Participant (P)	Intervention (I)	Comparison (C)	Outcome (O)						
A. Combined therapy																				
(a) Protein supplementation plus RET																				
Casein+RET	1	56	0.00%	100.00%	0.00%	No Serious	0.52 [-0.62; 1.66]		0.52 [-0.62; 1.66]	No serious	Elder people	PS+RET	RC, PLA, AC	SPPB	No serious	0.52 [-0.62; 1.66]	Serious	*p* = 0.10	Undetected	⊕⊕⊕⊖
Collagen+RET	0		28.62%	71.07%	0.31%	No Serious		0.73 [-0.57; 2.03]	0.73 [-0.57; 2.03]	No serious	Elder people	PS+RET	RC, PLA, AC	SPPB	No serious	0.73 [-0.57; 2.03]	Serious	*p* = 0.10	Undetected	⊕⊕⊕⊖
DP+RET	4	392	80.93%	18.20%	0.87%	Serious	0.43 [-0.11; 0.98]	0.82 [-0.22;1.86]	0.52 [0.03; 1.00]	No serious	Elder people	PS+RET	RC, PLA, AC	SPPB	No serious	0.52 [0.03; 1.00]	No serious	*p* = 0.10	Undetected	⊕⊕⊕⊖
MP+RET	2	93	54.26%	45.54%	0.20%	Serious	-0.02 [-0.86; 0.82]	0.67 [-0.06; 1.41]	0.37 [-0.18; 0.92]	No serious	Elder people	PS+RET	RC, PLA, AC	SPPB	No serious	0.37 [-0.18; 0.92]	Serious	*p* = 0.10	Undetected	⊕⊕⊖⊖
WP+RET	4	414	53.13%	46.46%	0.41%	Serious	0.71 [0.13; 1.29]	0.77 [0.10; 1.43]	0.73 [0.30; 1.17]	No serious	Elder people	PS+RET	RC, PLA, AC	SPPB	No serious	0.73 [0.30; 1.17]	No serious	*p* = 0.10	Undetected	⊕⊕⊕⊖
(b) Protein supplementation plus AET																				
DP+AET	1	29	28.00%	71.10%	0.90%	No Serious	0.83 [-0.43; 2.08]	-0.45 [-1.20; 0.29]	-0.12 [-0.76; 0.52]	No serious	Elder people	PS+AET	RC, PLA, AC	SPPB	No serious	-0.12 [-0.76; 0.52]	Serious	*p* = 0.10	Undetected	⊕⊕⊕⊖
MP+AET	0		19.66%	79.60%	0.74%	No Serious		-0.62 [-1.87; 0.63]	-0.62 [-1.87; 0.63]	No serious	Elder people	PS+AET	RC, PLA, AC	SPPB	No serious	-0.62 [-1.87; 0.63]	Serious	*p* = 0.10	Undetected	⊕⊕⊕⊖
WP+AET	0		48.63%	50.71%	0.66%	No Serious		0.87 [-0.27; 2.01]	0.87 [-0.27; 2.01]	No serious	Elder people	PS+AET	RC, PLA, AC	SPPB	No serious	0.87 [-0.27; 2.01]	Serious	*p* = 0.10	Undetected	⊕⊕⊕⊖
(c) Protein supplementation plus MET																				
Casein+MET	0		50.68%	43.68%	5.64%	Serious		0.57 [-0.57; 1.72]	0.57 [-0.57; 1.72]	No serious	Elder people	PS+MET	RC, PLA, AC	SPPB	No serious	0.57 [-0.57; 1.72]	Serious	*p* = 0.10	Undetected	⊕⊕⊖⊖
DP+MET	6	2020	12.29%	79.67%	8.04%	No Serious	0.39 [-0.05; 0.83]	0.30 [-0.44; 1.05]	0.37 [-0.01; 0.74]	No serious	Elder people	PS+MET	RC, PLA, AC	SPPB	No serious	0.37 [-0.01; 0.74]	Serious	*p* = 0.10	Undetected	⊕⊕⊕⊖
MP+MET	2	473	3.80%	77.99%	18.21%	No Serious	0.61 [-0.14; 1.35]	0.58 [-0.43; 1.60]	0.60 [0.00; 1.20]	No serious	Elder people	PS+MET	RC, PLA, AC	SPPB	No serious	0.60 [0.00; 1.20]	No serious	*p* = 0.10	Undetected	⊕⊕⊕⊕
Rice+MET	0		25.18%	59.15%	15.67%	No Serious		0.45 [-0.82; 1.72]	0.45 [-0.82; 1.72]	No serious	Elder people	PS+MET	RC, PLA, AC	SPPB	No serious	0.45 [-0.82; 1.72]	Serious	*p* = 0.10	Undetected	⊕⊕⊕⊖
SP+MET	0		11.52%	62.77%	25.71%	No Serious		0.54 [-0.89; 1.97]	0.54 [-0.89; 1.97]	No serious	Elder people	PS+MET	RC, PLA, AC	SPPB	No serious	0.54 [-0.89; 1.97]	Serious	*p* = 0.10	Undetected	⊕⊕⊕⊖
WP+MET	2	102	49.23%	29.49%	21.28%	No Serious	1.04 [0.21; 1.87]	0.56 [-0.22; 1.35]	0.79 [0.22; 1.36]	No serious	Elder people	PS+MET	RC, PLA, AC	SPPB	No serious	0.79 [0.22; 1.36]	No serious	*p* = 0.10	Undetected	⊕⊕⊕⊕
B. Monotherapy																				
(a) Exercise training alone																				
RET	2	48	33.14%	66.57%	0.29%	No Serious	0.17 [-0.75; 1.09]	0.43 [-0.11; 0.96]	0.36 [-0.10; 0.82]	No serious	Elder people	RET	RC, PLA, AC	SPPB	No serious	0.36 [-0.10; 0.82]	Serious	*p* = 0.10	Undetected	⊕⊕⊕⊖
MET	1	137	16.81%	45.10%	38.09%	No Serious	-0.08 [-1.14; 0.99]	0.61 [-0.06; 1.29]	0.42 [-0.15; 0.99]	No serious	Elder people	MET	RC, PLA, AC	SPPB	No serious	0.42 [-0.15; 0.99]	Serious	*p* = 0.10	Undetected	⊕⊕⊕⊖
(b) Protein supplementation alone																				
DP	1	27	13.81%	83.69%	2.50%	No Serious	-0.33 [-1.59; 0.93]	-1.15 [-1.91; -0.40]	-0.94 [-1.59; -0.29]	No serious	Elder people	PS	RC, PLA, AC	SPPB	No serious	-0.94 [-1.59; -0.29]	No serious	*p* = 0.10	Undetected	⊕⊕⊕⊕
MP	0		29.83%	70.01%	0.16%	No Serious		0.26 [-1.25; 1.77]	0.26 [-1.25; 1.77]	No serious	Elder people	PS	RC, PLA, AC	SPPB	No serious	0.26 [-1.25; 1.77]	Serious	*p* = 0.10	Undetected	⊕⊕⊕⊖
WP	3	66	20.04%	79.34%	0.62%	No Serious	0.28 [-0.48; 1.04]	-0.09 [-1.02; 0.84]	0.13 [-0.46; 0.72]	No serious	Elder people	PS	RC, PLA, AC	SPPB	No serious	0.13 [-0.46; 0.72]	Serious	*p* = 0.10	Undetected	⊕⊕⊕⊖

[a]Values present the number of studies which provided direct comparisons for each indicated treatment arm versus the regular control.
[b]Performed by Begg–Mazumdar rank correlation test
[c]Certainty of evidence is graded as follows: High: ⊕⊕⊕⊕; Moderate: ⊕⊕⊕⊖; Low: ⊕⊕⊖⊖; Very low: ⊕⊖⊖⊖.
AC, active comparater; 95% CI, 95% confidence interval; DP, dietary protein; GRADE, Grading of Recommendations, Assessment, Development and Evaluations; MET, multicomponent exercise training; MP, milk protein; PLA, placebo supplementation; PS, protein supplementation; RC, regular care; RET, resistance exercise training ; SMD, standardized mean difference; SPPB, short physical performance battery; SP, soy protein; WP, whey protein.
